# Developing Heterogeneous Porous 3D-Printed SiO_2_-Pd-K_2_SiO_3_ Monolithic Catalyst via Surface MOF Growth and Pyrolysis for the Synthesis of Antitumoral Isatins

**DOI:** 10.3390/pharmaceutics17040505

**Published:** 2025-04-11

**Authors:** Alexandrina Druta, Rania Bouhmala, Teqwa Ragdi, Mariangel Luna, Manuel Bañobre-López, Christian F. Masaguer, Manuel Amorín, Silvia Barbosa, Pablo Taboada, Alberto Coelho

**Affiliations:** 1 Department of Organic Chemistry, Faculty of Pharmacy, University of Santiago de Compostela, 15782 Santiago de Compostela, Spain; alexandrina.druta@usc.es (A.D.); rania.bouhmala@rai.usc.gal (R.B.); teqwa.ragdi@rai.usc.gal (T.R.); christianf.masaguer@usc.es (C.F.M.); manuel.amorin@usc.es (M.A.); 2 Colloids and Polymers Physics Group, Department of Physics of Particles, Faculty of Physics, University of Santiago de Compostela, 15782 Santiago de Compostela, Spain; mariagel.luna@rai.usc.es (M.L.); silvia.barbosa@usc.es (S.B.); 3 Institute of Materials-USC (IMATUS), University of Santiago de Compostela, 15782 Santiago de Compostela, Spain; 4 International Iberian Nanotechnology Laboratory, Avenida Mestre José Veiga s/n, 4715-330 Braga, Portugal; manuel.banobre@inl.int

**Keywords:** heterogeneous catalysis, monolithic catalysts, 3D printing, isatins, HeLa, MCF-7, MDA-MD231, antiproliferative, ZIF-8

## Abstract

**Background/Objectives**: The isatin nucleus is a privileged scaffold in drug discovery, particularly due to its proven relevance in anticancer research. Developing reusable heterogeneous 3D catalysts for drug synthesis represents a critical challenge in both industrial and academic contexts. This multi and interdisciplinary work aimed to design and synthesize a novel 3D-printed silica-based porous catalyst functionalized with palladium, evaluate its catalytic performance in isatin drug synthesis, and assess the antiproliferative activity of the resulting compounds against tumor cell lines such as HeLa, MCF-7, and MDA-MB231. **Methods**: The novel multifaceted approach to synthesizing this heterogeneous catalyst involved the surface growth of a metal–organic framework (ZIF-8) on 3D-printed silica support, followed by potassium silicate coating and pyrolysis. **Results**: After detailed physicochemical characterization, the catalyst was tested in challenging “double” palladium-catalyzed cross-coupling reactions (Suzuki, Stille, and Heck), demonstrating robustness, reusability, and high efficiency in producing bis-1,5-aryl, alkynyl, and alkenyl-isatin derivatives. Importantly, no leaching of palladium species was detected during the catalytic cycles, further underscoring the stability of the system. These isatin-based compounds exhibited remarkable cytotoxicity, with selective molecules achieving nanomolar potency against MCF-7 cells, surpassing reference drugs such as doxorubicin and sunitinib. **Conclusions**: This study not only introduces a novel strategy for fabricating porous heterogeneous catalysts from sintered surfaces but also highlights new biomolecules with promising applications in cancer research.

## 1. Introduction

The efficient and sustainable synthesis of biomolecules plays a crucial role in cancer research, where the development of innovative therapies relies on a steady and reliable supply of bioactive compounds. The growing demand for specific molecules to combat tumoral cells and tissues highlights in many cases the need for rapid, selective, and reproducible catalytic processes. In this context, heterogeneous catalysts stand out as versatile and sustainable tools, offering the advantage of reusability without significant loss of activity, thereby reducing costs and minimizing environmental impact. These advancements not only accelerate the discovery of new therapeutic strategies and compounds but also promote more accessible and responsible research in the fight against cancer.

The development of new compounds targeting cancer cells focuses on achieving high selectivity for malignant cells while also aiming for broader applicability across multiple cell lines. Initial verification of this selectivity and efficacy is carried out in vitro through drug screening processes, often with HeLa, MCF-7, and MDA-MB-231 cell lines serving as key models in oncology research. HeLa, a cervical cancer model, is valuable for targeting metabolic pathways like glycolysis [1]. MCF-7, an estrogenic receptor-positive (ER+) breast cancer line, provides insights into hormone-sensitive therapies [2], while MDA-MB-231, a triple-negative breast cancer model, is crucial for addressing aggressive and drug-resistant tumors [3]. Finding drugs specific to each tumor cell line enables the development of personalized treatments, enhancing clinical efficacy by targeting the unique characteristics of each patient. Additionally, studying resistant lines like MDA-MB-231 provides valuable insights into overcoming therapeutic resistance [4,5]. Finally, specific drugs minimize side effects by reducing toxicity to healthy tissues.

**Chart 1:**
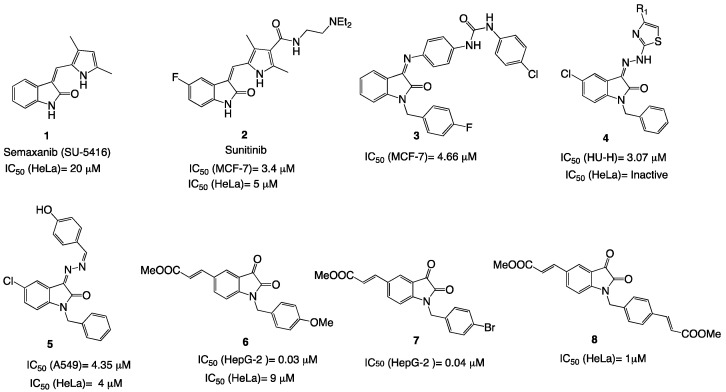
Some of the main isatin-based biomolecules active in different tumor cell lines.

In the field of cancer research, the isatin (1H-indol-2,3-dione) core has garnered considerable interest over the last two decades as a notable potential therapeutic framework [6]. Chart 1 highlights some of the key isatin compounds discovered in recent cancer research [7,8,9,10]. Semaxanib (SU5416) **1**, a selective VEGFR inhibitor, exhibits antiproliferative effects on HeLa cells with an IC_50_ of 20 μM after a 4-day proliferation assay [11]. This molecule has been widely studied for its ability to block VEGFR signaling and its potential antiangiogenic effects in various cellular models. Sunitinib **2** was approved by the FDA in 2006 for the treatment of advanced renal cell carcinoma and gastrointestinal stromal tumors. Its effects on the HeLa cell line were also studied, showing increased apoptosis, reduced mitotic index, and a slower cellular proliferation rate when treated with 5 μM of the drug for 72 h [12].

In recent years, new prototypes derived from the *N*-benzyl-isatin structure have emerged. In 2016, Eldehna et al. reported the potent cytotoxic of biphenylurea derivatives containing indolin-2-one moieties like **3**, showing activity against breast cancer MCF-7 cells (IC_50_ = 4.6 μM). This kind of compound was found to be more active than the reference antineoplastic drug doxorubicin (IC_50_ = 7.3 μM) [13]. In addition, the in vitro anticancer evaluation of type **4** isatin hydrazones was conducted on three cancer cell lines: HeLa, MCF-7, and HuH-7 (liver carcinoma, IC_50_ = 3 μM). However, none of the compounds in this series displayed noteworthy cytotoxic effects on HeLa or MCF-7 cells, indicating that these molecules may lack efficacy against these types of cancer [14]. The hydrazone derivative compound **5**, on the other hand, demonstrated antiproliferative activity against HeLa cells (IC_50_ = 4 μM) that was twice as effective as the reference tyrosine kinase inhibitor gefitinib [15]. Compound **6**, a derivative of 5-(2-carboxyethenyl)isatin, has previously been identified as a highly potent anticancer agent by Chang et al. [16]. These authors further investigated its impact on angiogenesis. Remarkably, **6** demonstrated selective cytotoxicity toward HepG-2 liver hepatocellular carcinoma cells, with an IC_50_ of 30 nM. Additionally, **6** strongly promoted apoptosis, induced G2/M phase cell cycle arrest, and suppressed the migration of HepG-2 cells. These findings indicate that **6** holds promise as an inhibitor of tumor angiogenesis by disrupting the autophosphorylation of AKT, mTOR, and ERK [16]. This compound was also shown to have significant cytotoxic activity with, an IC_50_ value of 30 nM against human T lymphocyte Jurkat cells [17]. Compound **7**, containing a bromine atom in the *para* position of the benzene ring, demonstrated similar therapeutic activity in HepG-2 liver hepatocellular carcinoma cells with IC_50_ = 40 nM [18]. However, its activity in HeLa, MCF-7, or MDA-MB-231 cells was not reported. Collectively, these active drug prototypes and their bioactivities in various tumoral cell lines show a preference for substitution at the 5-position, with benzyl groups on the nitrogen of the oxindole ring. Based on these findings, which highlight the significance of this heterocycle in the antiproliferative activity, we began our own investigation. In 2023, we reported molecule **8**, a bis-(2-carboxyethenyl)isatin containing 2-carboxyethenyl groups at the benzyl and 5-position of the isatin ring [19]. This exhibited significant anticancer activity in HeLa cells (IC_50_ = 1 μM)—nine times more potent than **6**, with minimal cytotoxicity in normal murine Balb-3T3 cells (IC_50_ > 10 μM). However, methyl esters are prone to enzymatic hydrolysis by esterases in the plasma, gastrointestinal tract, and liver, reducing their metabolic stability, especially when administered orally. To improve stability, they can be replaced with more robust functional groups. Then, these results underline the need for new prototypes to explore structure–activity relationships.

Pharmacomodulation of prototype **8** is desirable by altering the structure at the 5-position of the isatin ring and the para-position of the benzyl ring to optimize the steric and electronic properties that may influence the pharmacological activity in these series, aiming to enhance therapeutic potency in HeLa and other cell lines.

Constructing new drug candidate prototypes like **6**, **7**, and **8**, represented in Chart 1, requires palladium catalytic processes. Palladium-catalyzed cross-coupling reactions (PCCCRs) serve as a fundamental approach in C–C bond formation [20,21,22,23,24,25], yet the cost of palladium makes its fixation on stable, robust supports highly desirable for cost savings [26].

In this sense, the development of robust and efficient heterogeneous monolithic catalysts is essential for industrial and pharmaceutical applications [27,28,29,30,31,32,33]. Monolithic catalysts provide significant advantages, including ease of separation during the work up and recycling, both of which are crucial for sustainable and cost-effective manufacturing.

Within the pharmaceutical industry, the stability of metal catalysts on solid supports is paramount. Monolithic catalysts functionalized with metal species must be designed to prevent leaching—unwanted metal dissolution into the reaction mixture—which can compromise the purity of pharmaceutical products with metal residues [34]. This is particularly critical when synthesizing sensitive compounds like pharmaceuticals, where even trace metal contamination can affect drug efficacy and safety. Ensuring that metal species are firmly anchored on the monolith surface is thus essential to maintaining catalyst performance and product integrity.

Traditional monolithic catalysts face challenges related to limited porosity, which can restrict catalytic performance and capacity. There are some examples of 3D-printed monolithic heterogeneous catalysts applied to palladium-catalyzed coupling reactions [35,36,37]. However, in some cases, the materials used are not economically viable at an industrial level (e.g., carbon nanotubes) [36]. Recently, Bulatov et al. reported a 3D-printed monolith prepared via Selective Laser Sintering (SLS) using polypropylene as the base material and palladium nanoparticles on silica. However, the formation of soluble species during the reaction could also result in leaching and hinder catalyst recovery. Furthermore, reliance on SLS technology may limit the scalability and accessibility to industrialize the production process [37]. In other cases, reliance on advanced technologies such as light-assisted 3D printing and Atomic Layer Deposition (ALD) requires specialized and expensive reagents and equipment [35]. For ceramics, manufacturing these devices via robocasting often requires sintering, which excessively compacts the material [38]. In particular, the final sintering process reduces porosity—especially macro- and mesoporosity—which may limit catalytic activity, regardless of the degree of ceramic surface functionalization with the metal. Recently, Wang et al. reported a method of preparing a Pd/C catalytic reactor using coaxial 3D printing that combines multimaterial 3D printing, integrated molding, and complex biomimetic structure fabrication [39]. However, this intriguing method required the use of specialized coaxial 3D printers. Some of our previous works with monolithic catalysts involved silica-based materials also synthesized through 3D printing via robocasting and sintering processes [40,41,42]. This step was followed by surface functionalization to bind metal species, either through silanization and coordination with metal species [40] or via direct impregnation of the ceramic surface using strong electrostatic adsorption (SEA) [41]. Another alternative is custom fabrication of palladium/ceramic *cermets* [42], in which the metal component is uniformly distributed within and across the material’s surface, although sintering similarly reduces external porosity. While these methods laid a strong foundation, they yielded monoliths with limited porosity due to restrictions in the silanization step, which constrained the surface area available for catalytic reactions and reduced catalyst efficiency.

Recent advances have highlighted the potential of Metal–Organic Frameworks (MOFs) as precursors or templates for fabricating nanostructured catalysts [43,44,45]. ZIF-8 (Zeolitic Imidazolate Framework-8), a well-studied MOF with a Zn-based structure, has attracted attention for its high surface area and uniform pore distribution [46,47,48]. However, the inherent instability of MOFs under certain catalytic conditions, particularly under thermal or solvent exposure, limits their direct application in heterogeneous catalysis [49,50,51,52]. To address these limitations, some strategies that combine MOFs with ceramic supports followed by post-synthetic modifications, like hydrothermal treatment or the double-solvent strategy, have been explored [53,54,55]. However, these methods have not been tested on processed sintered silica.

Alkali silicates, such as sodium silicate (Na_2_SiO_3_) and potassium silicate (K_2_SiO_3_), are key cementing agents in ceramics due to their ability to bond particles by forming chemically stable three-dimensional structures. They also provide hydrophobic properties, protecting ceramics from moisture. Sodium silicate is more common and cost-effective, whereas potassium silicate offers greater water resistance and chemical stability. These compounds are widely used in refractories, coatings, and advanced ceramic compositions, enhancing thermal and chemical durability under extreme conditions. Potassium silicate can behave as an effective inorganic binder in ceramic/MOF composites because, upon mixing and thermal treatment, it melts and consolidates the structure, promoting adhesion between the MOF and ceramic [56,57]. During pyrolysis, the silicate may form a SiO_2_ matrix that stabilizes the system and removes MOF carbon content without fully compromising its porous structure. This treatment can potentially yield a robust, chemically resistant composite that retains part of the MOF’s original porosity, a valuable feature for catalytic or adsorption applications where access to active sites is essential.

In this study, a novel approach for fabricating a nanoporous monolithic catalyst is firstly presented. The heterogeneous catalyst was obtained by functionalizing a sintered silica monolith through a multi-step process that includes impregnation with zinc nitrate, hetero-growth of ZIF-8 on the monolith surface via a layer-by-layer method, introduction of palladium species to generate Pd(0) species within the nanostructured framework, and a final consolidation layer of potassium silicate as cement, followed by pyrolysis. Secondly, we present the synthesis of diversely substituted isatins prepared via Heck, Suzuki, and Stille reactions, evaluating the catalytic activity and reusability of the monolithic catalyst. Finally, we report the antiproliferative activity and preliminary structure–activity relationships of the isatins synthesized in these series in HeLa, MCF-7, and MDA-MB-231 cells used as model tumoral cell lines.

## 2. Materials and Methods

Kimble^®^ vials in a PLS (6 Å~4) organic synthesizer (Activotec Ltd., Cambridge, UK) were used to perform the surface MOF-growing, metalation, impregnation with potassium silicate, and catalytic evaluation of the SiO_2_-monoliths. All reagents and solvents were purchased from Sigma Aldrich (St. Louis, MO, USA), including Amberlyst-15, potassium silicate anhydrous, 5-iodo-isatin, and palladium acetate, alkenes, organostannanes, and boronic acids.

### 2.1. Synthesis of the Monolithic 3D-SiO_2_@Zn/Pd@K_2_SiO_3_ Catalyst

#### 2.1.1. 3D-Printed SiO_2_ Monolith Support Fabrication

The SiO_2_ colloidal ink was prepared as previously reported [40] by mixing 50 g of SiO_2_ powder (average particle size 6.3 μm), 12.08 g of poly(vinyl butyral-*co*-vinyl alcohol-*co*-vinyl acetate) (80% vinyl butyral), and 3.99 g of polyethylene glycol (Mw = 600) with 32.55 mL of 2-propanol (≥99.5%). This mixture was homogenized using a planetary centrifugal mixer (ARE-250, Thinky Corp., Tokyo, Japan) at 2000 rpm for 5 cycles of 2 min each. The ink was loaded into a 3 mL syringe (Nordson EFD) fitted with a 410 μm cylindrical nozzle.

Extrusion was performed using an air-pressure system (Performus VII with HP7x) attached to a robotic deposition apparatus (model A3200, Aerotech Inc., Pittsburgh, PA, USA). The printed structures were designed as cylinders with a 10 mm diameter and 40 layers, featuring a body-centered tetragonal (bct) symmetry, rod diameters of 410 μm, and 1 mm spacing between rods. After fabrication, the samples were dried and subjected to debinding at 400 °C for 1 h (2 °C/min heating rate) followed by sintering at 1500 °C for 3 h (5 °C/min heating rate).

#### 2.1.2. Surface Activation and Impregnation

Surface Activation: A sintered SiO_2_ monolith (1 cm tall and 0.8 cm in diameter) was immersed in 3 mL of a 30% H_2_O_2_ solution and heated under reflux at 150 °C for 30 min. Subsequently, the monolith was taken out of the flask and subjected to a washing step with distilled water at 100 °C for 30 min. Finally, it was vacuum-dried for 1 h. The surface impregnation with zinc species was performed by submerging the previously activated monolith in a Kimble^®^ vial containing a zinc nitrate aqueous solution (0.03 M) and heating it at 90 °C for 48 h. The monolith was then removed from the vial, washed with distilled water, vacuum-dried for 2 h, and subsequently placed in an oven at 100 °C for 12 h.

#### 2.1.3. Surface MOF Growth and Metalation

Surface MOF Growth: The Zn-activated monolith was treated with an aqueous solution of 2-methylimidazole (2-IMI) (5 mg in 2 mL of distilled water) at 80 °C, stirring in PLS for 12 h. The monolith was then washed with distilled water. Subsequently, it was impregnated again with zinc nitrate (0.03 M, 10 mg in 2 mL of distilled water) for 12 h at 80 °C, then washed again with distilled water. These two steps followed a sequence of 3 cycles of alternating baths of the metal precursor and the organic ligand (12 h each one) to obtain the ZIF-8 growth. After a final washing step with distilled water and methanol (MeOH), the monolith was vacuum-dried at room temperature. The obtained monolith (3D-SiO_2_@ZIF-8) was analyzed using EDX and SEM before proceeding with the next step.

Metalation: In a Kimble^®^ vial, the 3D-SiO_2_@ZIF-8 monoliths obtained in the previous step were treated with a solution of palladium acetate (10 mg) in 4 mL of ethanol (EtOH) at room temperature with orbital stirring for 24 h under argon to yield 3D-SiO_2_@ZIF-8@Pd. The resulting black-colored 3D-SiO_2_@ZIF-8@Pd monolithic catalyst was washed sequentially with distilled water, methanol, dichloromethane, diethyl ether, and finally dried under reduced pressure for 24 h.

#### 2.1.4. Impregnation with Potassium Silicate

The dry monolith, with MOF and palladium species on its surface, was immersed in an aqueous solution of potassium silicate (30% *w*/*w*). The monolith was left undisturbed at room temperature for 10 min. Then, the monolith was immersed once again, removed from the solution, and placed at room temperature for 5 min and directly vacuum-dried for 5 h, followed by drying in an oven at 100 °C for 12 h to give 3D-SiO_2_@ZIF-8@Pd@K_2_SiO_3_.

#### 2.1.5. Pyrolysis

The tubular furnace used was a Nabertherm GmbH model R-252-2AN (Lilienthal, Germany) with a temperature range of 1100 to 1800 °C. The resulting monolith loaded with ZIF-8, palladium, and silicate on its surface was treated in the furnace at 400 °C with gradual heating for 2 h, operating under an argon atmosphere, to give 3D-SiO_2_@Pd@K_2_SiO_3_.

### 2.2. Techniques and Equipment for Catalyst Characterization

After each chemical treatment of the monolith, it was characterized at every stage to ensure a properly functionalized substrate at each step, primarily confirmed using Scanning Electron Microscopy (SEM) and Energy-Dispersive X-ray Spectroscopy (EDX). X-Ray Diffraction (XRD) analysis was conducted using a Bruker D8 Advance diffractometer. The surface topography and microstructural features of the samples were examined using scanning electron microscopy (SEM, JEOL 6400, JEOL Corporation, Akishima, Japan) and a stereomicroscope (Olympus SZX12, Olympus, Tokyo, Japan). The elemental composition of the sintered samples was analyzed using an energy-dispersive X-ray spectrometer (EDS, AZTEC/Xact, Oxford, UK). For surface analysis, X-ray Photoelectron Spectroscopy (XPS) experiments were carried out in ultra-high vacuum multi-chamber multi-technique system ESCALAB250Xi from Thermo Scientific with a base pressure below 5 × 10^−10^ mbar. The XPS spectra were generated using a monochromated Al *Kα* source (1486.68 eV, X-ray spot size on the surface 650 μm). The XPS spectra were acquired using the hemispherical electron energy analyzer at the normal to the surface. Survey and high-resolution spectra were acquired using a 100 eV and 30 eV pass-energy with an energy step of 1 eV and 100 meV, respectively. The spectra were fitted using the Lorentzian–Gaussian curve shapes and Shirley-like background. Charge compensation was applied using electron and Ar ion flood guns. The porosity properties of the samples were measured using mercury intrusion porosimetry using an Autopore IV 9500 equipment (Micromeritics, Norcross, GA, USA) with a 3 mL penetrometer at pressures of 0.07–1724 bar.

### 2.3. Chemistry

All catalytic reactions were carried out in Kimble^®^ vials using a PLS Organic Synthesizer (4 × 6), Activotec Ltd., Cambridge, UK. The reactions were monitored using TLC on 2.5 mm Merck silica gel GF 254 strips, where the purified products consistently displayed a single spot. Detection was achieved using UV light and/or iodine vapor. Purification of the isolated products was performed using flash chromatography. Characterization of the synthesized compounds was conducted using spectroscopic and analytical techniques. NMR spectra were recorded using Bruker AM 500 MHz (^1^H) and 125 MHz (^13^C), as well as XM500 spectrometers. Chemical shifts (δ) were reported relative to tetramethylsilane as an internal standard, with coupling constants (*J*) provided in Hertz. Proton and carbon NMR spectra (^1^H,^13^C) were obtained in CDCl_3_. Melting points were measured using a Gallenkamp apparatus and were uncorrected. Mass spectra were recorded using a Varian MAT-711 mass spectrometer (Varian MAT, Bremen, Germany).

#### 2.3.1. Synthesis of Precursors **10** and **11**

Precursor **10** was prepared from 5-iodoisatin **9** following the procedure previously reported [19]. Compound **11** was synthesized as follows: In a Kimble^®^ vial, 3 mmol of *N*-benzyl-isatin **10** were dissolved in an excess of ethylene glycol (6 mL) and 1.5 mmol of Amberlyst-15 resin (loading: 1.5 mmol/g), and 50 mg of molecular sieves (3 Å) were added. The mixture was heated to 120 °C in the PLS Organic Synthesizer (4 × 6) for 24 h. Once the reaction was complete, as monitored using TLC, the mixture was vacuum filtered using a Buchner funnel and washed with ethanol. The filtrate was evaporated to dryness, and the resulting solid was purified using column chromatography using a 1:7 mixture of AcOEt/Hexane (1:5) to give a yellowish solid. The yield was 80%.

#### 2.3.2. General Procedure for Double Heck Reactions (Compounds **8**, **15**, **16**, **17**, **25**, **26**, **27**)

A solution of 1.5 mmol of the starting material, iodobenzylisatin **10**, 6 mmol of triethylamine, and 3.5 mmol of the corresponding alkene (styrene, acrylonitrile, or methyl/ethyl-acrylates) was prepared in 4 mL of dimethylformamide (DMF). Subsequently, the catalyst 3D-SiO_2_@Pd@K_2_SiO_3_ was added to the mixture, which was stirred at 85 °C under an inert argon atmosphere. The reaction progress was monitored using thin-layer chromatography (TLC) until the complete consumption of the starting material. For the TLC analysis, a mixture of AcOEt and hexane was used as the mobile phase. During the reaction (after 3 h), the monosubstituted product was detected using TLC (the corresponding intermediates **12** and **13** could be isolated, including **14**, which did not give the disubstituted isatin), which, over a longer reaction time (12 h), led to the formation of the disubstituted products **8**, **15**, **16**, or **17**. Finally, the catalyst was extracted from the vial, washed with EtOH and diethyl ether, and dried for reuse. The reaction mixture was evaporated to dryness and the resulting residue was purified using preparative TLC, employing AcOEt/hexane as the mobile phase. Compounds **25**, **26**, and **27** were prepared as above, but using **11** as the starting product. The final disubstituted compounds were obtained via heating at 85 °C under an inert argon atmosphere for 12 h.

#### 2.3.3. General Procedure for Double Stille Reactions (Synthesis of Compounds **23**, **24**, **29**, **30**, **31**, and **32**)

The compounds were synthesized using the Stille reaction. In a Kimble^®^ vial coated with a suitable material, iodoisatin (**10**, for compound **23**, **24**) or **11** (for the synthesis of **29**, **30**, **31**, **32**) (0.5 mmol) and the corresponding organostannane (0.6 mmol) were dissolved in 5 mL of toluene. The 3D-SiO_2_@Pd@K_2_SiO_3_ catalyst (total Pd content on monolith: 5.2 mg, 3% mol Pd) was then added to the solution. The mixture was heated to 80 °C with orbital stirring for 12 h until the starting material was fully consumed. Afterwards, the mixture was allowed to cool, and the catalyst was separated, washed, and recovered for future use as described above. To remove tin byproducts, the mixture was washed with 10 mL of a 1 M KF solution (3 Å). The organic phase was extracted using ethyl acetate (AcOEt) and dried with anhydrous Na_2_SO_4_. The solvent was evaporated under reduced pressure, and the resulting solid was purified using flash chromatography (dichloromethane/MeOH) and recrystallization (iPrOH) to yield compounds **23**, **30**, **31**, and **32**.

The synthesis of compounds **24** and **29** via double Stille was performed using **11** as the starting product as above, but after consumption of the starting material and detecting the coupling product (not isolated), HCl 3N was added to the mixture, and the reaction was heated at 70 °C during 3 h to give compound **29**. For compound **24**, HCl 6N was used, heating for 24 h. The work-up process was performed as described above.

#### 2.3.4. General Procedures for Suzuki Reactions

Procedure A (for the synthesis of compounds **18**, **19**, **20**, **21**, and **22**): In a Kimble^®^ vial, 1.5 mmol of the starting material (**10**), 6 mmol of TEA, and 3.5 mmol of the corresponding boronic acid were dissolved in 5 mL of DMF. The catalyst 3D-SiO_2_@Pd@ K_2_SiO_3_ was added to the mixture, and the reaction was stirred at 75 °C under an argon atmosphere for 24 h. During the reaction, adduct **18**, **19**, or **20** was detected first and eventually isolated. Upon completion of the reaction (consumption of the starting material and the corresponding adduct), as confirmed via TLC monitoring, the catalyst was removed using tweezers, washed, and stored for reuse. The product was extracted using dichloromethane as the solvent. The filtrate was evaporated to dryness using a rotary evaporator, and the resulting residue was purified via preparative TLC using a mobile phase of AcOEt (1)/Hex (5) to give the final disubstituted isatin (**21**, **22**). For compound **20**, phenylboronic acid was used. For isolation of compounds **18** and **21**, 4-methoxicarbonyl-phenyl-boronic acid was used. For isolation of compounds **19** and **22**, boronic acid-4-carbonitrile was used.

Procedure B for the synthesis of compounds **28**, **33**, **34**, and **35**: In a Kimble^®^ vial, 1.5 mmol of the starting material (**10**), 6 mmol of K_2_CO_3_, and the corresponding boronic acid (3.5 mmol) were dissolved in a mixture of dimethoxyethane (DME) (6 mL)/H_2_O (1 mL). The catalyst 3D-SiO_2_@Pd@K_2_SiO_3_ was added, and the reaction was stirred in a PLS organic synthesizer at 95 °C for 6–8 h. Upon completion of the reaction, confirmed via TLC monitoring, the mixture was washed with distilled water and extracted with AcOEt. The organic phase was dried over anhydrous sodium sulfate (Na_2_SO_4_), followed by evaporation to dryness using a rotary evaporator. The resulting semi-solid residue was purified via preparative TLC using a mobile phase of AcOEt/Hex. The final products were characterized using NMR spectroscopy and mass spectrometry. The spectroscopic data of the related compounds are available in the Supporting Information.

#### 2.3.5. Hot Filtration Test and Recyclability

The Suzuki reaction was performed using 3D-SiO_2_@Pd@K_2_SiO_3_ as the catalyst. A reaction mixture was prepared in a Kimble^®^ vial by combining 1 mmol of precursor **11**, 1.2 mmol of phenylboronic acid, 3 mmol of potassium carbonate, and 5 mL of a DME/water solution (1:1 *v*/*v*). The catalytic monolith was added to the mixture, which was heated to 95 °C under constant stirring for 30 min. Without allowing the mixture to cool, the catalyst was removed via hot filtration using an appropriate funnel, ensuring no solids remained in the filtrate. The filtrate was kept at 95 °C under continuous stirring for 6 h, and the reaction progress was monitored using TLC. No formation of the final product **34** was detected in the absence of the catalyst. Additionally, samples of the filtrate were analyzed using ICP-MS and no palladium was detected, confirming the catalyst’s stability and the absence of leaching. The halted conversion and the absence of the final product in the filtrate confirmed the heterogeneous nature of the catalytic process.

The recyclability was evaluated throughout the study. The catalyst showed no apparent surface damage and could be reused at least 10 times without any loss of catalytic activity in any of the three synthesis methods.

### 2.4. Anticancer Activity In Vitro

#### 2.4.1. Cell Lines and Cell Culture Conditions

HeLa and MDA-MB-231 cells were obtained from cell BioLabs (San Diego, CA, USA), whereas MCF-7 cells were kindly donated by Prof. J. A. Costoya (University of Santiago de Compostela, Spain). HeLa and MDA-MB-231 cells were cultured in DMEM, supplemented with 10% (*v*/*v*) FBS and 1% (*v*/*v*) of penicillin/streptomycin, sodium pyruvate, and nonessential amino acids (NEAAs). MCF-7 were culture in DMEM, supplemented with 10% (*v*/*v*) FBS, insulin (10 μg/mL), and 1% (*v*/*v*) of glutamine, penicillin/streptomycin, sodium pyruvate, and nonessential amino acids (NEAAs). All cell cultures were maintained under standard cell culture conditions (37 °C, 5% CO_2_, and 90% humidity).

#### 2.4.2. Cell Viability

The cytotoxicity of the synthesized compounds was tested in vitro using the CCK-8 cytotoxicity assay. HeLa, MDA-MB-231, and MCF-7 cells were seeded into 96-well plates (1.0 × 10^4^ cells/well) and grown for 24 h at an optical confluence of 80–90% under standard culture conditions in 100 μL of growth medium. Bare cells were used as the negative control. After 24 h of incubation, the cells were treated with the drug (**8**–**35**) at a concentration equivalent to 10 μM for 24 and 48 h. After incubation, the cells were washed with PBS (1×, pH 7.4) and 100 μL of culture medium with 10% (*v*/*v*) of the CCK-8 reagent added to each well and incubated for 1 h. After 2 h, the absorption at 450 nm of the cell samples was measured using a UV–vis microplate absorbance reader (Bio-Rad model 689, Hercules, CA, USA). Cell viability (SR, survival rate) was calculated as follows:

(1) $$SR=\frac{{Abs}_{sample}}{{Abs}_{blank}}\times 100$$

where Abs_sample_ is the absorbance at 450 nm for cell samples and Abs_blank_ is the absorbance corresponding to the sample controls without the drug.

For the determination of the half-maximal inhibitory concentration (IC_50_), a dose–response curve between the (**8**–**35**) concentrations and percent cell viability was plotted and fitted using a nonlinear least-squares fitting method (Microcal Origin 2021) to a four-parameter logistic equation:

(2) $$Y=min+\left(\frac{\left(max-min\right)}{1+10^{\left(\left(log{IC}_{50}-X\right)xp\right)}}\right)$$

where the original, %control, or %survival data are represented by Y along their minimal (min) and maximal (max) values; the isatin derivative concentration is represented by X; IC_50_ is the concentration at 50% maximal value; and p is the slope factor. The pharmacological toxicity tests were performed in triplicate.

## 3. Results and Discussion

### 3.1. Design of the Chemical Library

**Scheme 1:**
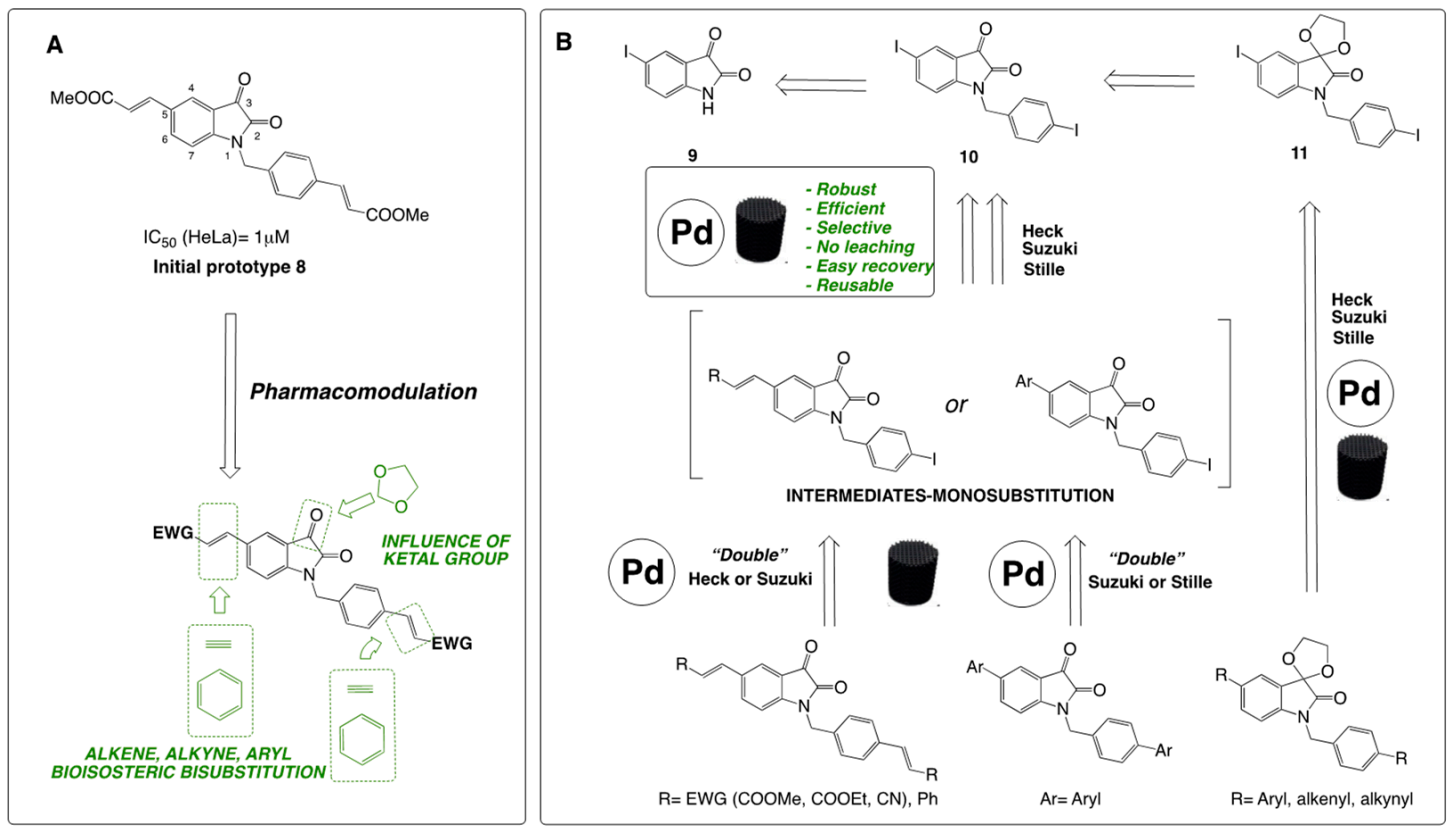
(**A**) Design of new bis-aryl, bis-alkenyl, and bis-alkynyl isatins; (**B**) retrosynthetic scheme of the compounds prepared in this work using the 3D-printed catalyst.

The synthetic strategy followed in this work is depicted in Scheme 1, illustrating the synthesis of new target isatin prototypes. This approach builds on previously active molecules in key cell lines and extends our earlier studies [19], which highlighted that the incorporation of two alkenyl groups at the benzylic group in 1 and the position 5 of the isatin ring, having electron-withdrawing groups (EWG), is optimal to enhance the cytotoxic activity against HeLa cells. Additionally, brominated derivatives at the *para* position of the phenyl ring showed promising activity, as corroborated by Han et al. [18]. The modifications planned in this work aim to explore the electronic and steric features of substituted isatins at positions 1 and 5 and the influence of their biological activity. The substitution of an alkene with an alkyne or a benzene ring as a linker between functional groups in biomolecules represents a rational pharmacomodulation approach (Scheme 1A). These modifications ensure electronic equivalence while providing tunable physical and chemical characteristics, aligning with the principles of bioisosterism to optimize pharmacological profiles. Therefore, the main objective here was replacing alkenes with alkyne or benzene groups, maintaining similar electronic properties while altering steric parameters, lipophilicity, or substituent orientation, favoring EWGs like -CN, -CHO, and -COOR. Interestingly, the antiproliferative activity of iodinated derivatives at the *para* position of the benzyl ring in these series remained unexplored. With careful control of reaction conditions, it is possible to access mono- or bis-substituted products from precursors **10** and **11**, obtained from isatin **9** (5-iodoindoline-2,3-dione). The simplified retrosynthetic strategy using a heterogeneous Pd-catalyst is depicted in Scheme 1B. These target structures can be obtained through Heck reactions (for alkenylation), Suzuki reactions (preferably for arylation), or Stille reactions (for the introduction of alkynes, aryl groups, and ketones) (Scheme 1B).

Finally, the reactivity of isatins bearing a cyclic ketal group at position 3 was additionally explored, along with its influence on biological activity, to understand the overall importance of the carbonyl group at C-3 in cytotoxicity across the three tumor cell lines. In all these transformations, the availability of a robust, efficient, and reusable heterogeneous catalyst was highly demanded in this work (Scheme 1B).

### 3.2. Design and Synthesis of the 3D-SiO_2_@Pd@K_2_SiO_3_ Catalyst

#### 3.2.1. Design of the 3D-Printed Catalytic System

**Figure 1:**
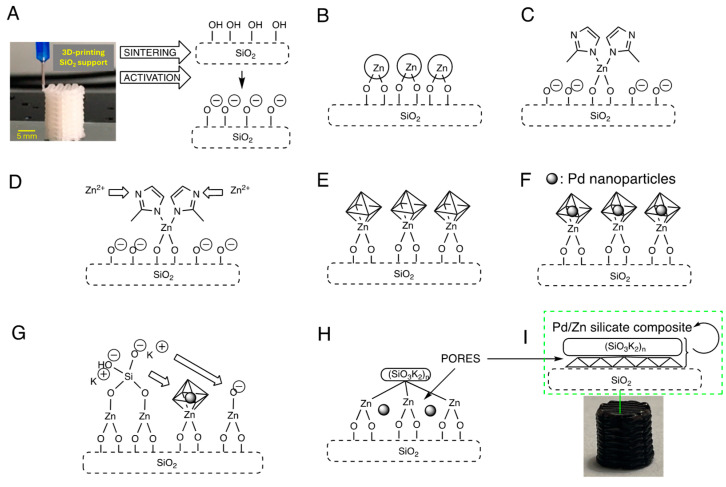
(**A**) 3D-printing, sintering, and surface activation of the silica monolithic support. (**B**) Impregnation of the monolith with an aqueous zinc nitrate solution, 90 °C, 48 h. (**C**–**E**) Growth of ZIF-8 on the monolith surface: cyclic treatment with 2-MIM and zinc nitrate, 90 °C, 24 h per stage (3 cycles). (**F**) Treatment with palladium acetate in EtOH, 40 °C, 24 h. (**G**) Treatment with potassium silicate, room temperature, 10 min. (**H**,**I**) 3D-SiO_2_@Pd@K_2_SiO_3_ after pyrolysis (400 °C) with the presence of Pd nanoparticles entrapped in the final composite surface.

The catalyst manufacturing process is depicted in Figure 1. The support monolith, 3D-printed in the first step, features a lattice of bars that provide specific surface area to the final structure after sintering. However, this thermal process reduces the material’s porosity. Therefore, the main goal was to create a system of interconnected pores on the monolith’s surface, built during the previous growth of a metal–organic framework (ZIF-8) on the 3D-printed ceramic substrate. MOF crystals with smaller sizes are recognized for their superior catalytic performance due to enhanced exposure of active sites. Nonetheless, synthesizing small-sized MOFs remains a significant challenge, as conventional methods typically favor the growth of larger crystals. The layer-by-layer approach followed in this work led to the growth of small ZIF-8 crystals and compensated for the loss of surface porosity caused by prior sintering of the ceramic support after the 3D-printing step. The MOF framework is an excellent medium for trapping palladium species via impregnation. Further treatment with potassium silicate acts as effective cement via infiltration in the ZIF-8 pores. Finally, the ZIF-8 is removed via pyrolysis (400 °C) to yield a catalytic system that retains some surface porosity and houses palladium active species within a composite protected by a sodium silicate layer. A final thermal treatment compacts the system and eliminates carbon, resulting in a stable and robust catalyst capable of operating under diverse catalytic conditions, surpassing the stability of surface-bound MOF systems. 

Although zinc remains in the final system as a “spectator metal” in the proposed catalysis, its presence could have beneficial effects. ZnO can act as a support or co-support that stabilizes active Pd species, particularly in low oxidation states such as Pd(0) and Pd(II) [58]. This stabilization can prevent the agglomeration or sintering of Pd during reactions, thereby enhancing the catalyst’s durability and reusability. Pd–Zn interactions can influence the regeneration of Pd(0) from PdO(0) or other oxidized species, thus improving the catalyst’s lifespan.

#### 3.2.2. Synthesis of the Catalyst

First, the monolithic support was obtained via 3D printing, using the direct ink writing technique and subsequent sintering in an oven at 1600 °C [40]. Then, the monolith surface was activated with a piranha solution (Figure 1A), increasing the density of hydroxyl groups to facilitate the subsequent adsorption of metal ions. The strong oxidative environment may also create negatively charged oxygen species (O^−^) on the monolith surface (Figure 1A). Subsequently, the monolith was impregnated with zinc nitrate via Strong Electrostatic Adsorption (SEA) (Figure 1B). The activated monolith was immersed in an aqueous solution of Zn(NO_3_)_2_. This led to the effective deposition of Zn species due to the Z-potential difference between the surface and the metallic species. The monolith became impregnated within 48 h under heating at 80 °C (Figure 1B). SEA interactions occur due to opposing electric charges on the support surface and the metal species, being influenced by factors like pH and the zeta potential [59]. While not covalent, these forces are stronger than van der Waals or physisorption, ensuring stable and uniform metal deposition. On silica, interactions often involve surface silanol groups (Si-OH/Si-OH), making SEA an effective method for achieving controlled metal dispersion, enhancing the catalytic performance of the final material. The next step involved the growth of ZIF-8 using a layer-by-layer method with alternating treatments of the monolith with zinc metal precursor and the organic ligand (Figure 1C–E). The growth of ZIF-8 from Zn(NO_3_)_2_ crystallites on silica is an effective approach to control the nucleation and distribution of the MOF. In this process, we deposited zinc crystals on the surface of the silica, which act as anchoring points where zinc coordinates with 2-methylimidazole (2-MIM), promoting uniform and stable hetero-growth of ZIF-8. This method allows for the adjustment of the MOF size and distribution by optimizing the Zn(NO_3_)_2_ concentration, pH, and Zn:2-MIM molar ratio. Subsequent cyclic treatments with 2-MIM and Zn nitrate (three cycles, 90 °C, 12 h) enabled the observation using SEM of ZIF-8 crystal growth on the ceramic surface. Palladium acetate was added in a further step to generate Pd(0) nanoparticles within the nanostructure, taking advantage of ZIF-8 as a template. Treatment with a solution of palladium acetate in ethanol—which is a natural reductant solvent for palladium—at room temperature for 24 h resulted in a darkened monolith, confirming the formation of Pd(0) species on the surface (1F).

To improve the stability of the catalyst, the ZIF-8-functionalized monolith was treated with a potassium silicate solution, which acted as a cementing agent, ensuring the structural integrity of the MOF and facilitating the creation of a stable composite (Figure 1G). As previously mentioned, this silicate enhances the structural integrity of MOF composites, improving stability during thermal treatments by encapsulating active metal sites and preserving catalytic functions.

Careful optimization of binder concentration is critical to maintain porosity, as an excess amount can compromise the structure. Overall, the use of potassium silicate provides a robust method for creating durable, thermally stable materials. Finally, the applied thermal treatment enhances the stability of the catalytic systems by eliminating organic residues such as ligands and creating a more compact and cohesive material. It strengthens the interaction between the support and the deposited metal, reducing metal leaching, and optimizes the thermal and mechanical resistance of the support. This process results in a more robust and durable catalyst that is suitable for demanding catalytic conditions. Therefore, the final pyrolysis at 400 °C removed the carbon-based ZIF-8 organic ligands, resulting in a carbon-free nanoporous composite, with Zn, O, Si, K, and Pd as the remaining elements on the surface (Figure 1H,I).

### 3.3. Characterization of the Catalyst

**Figure 2:**
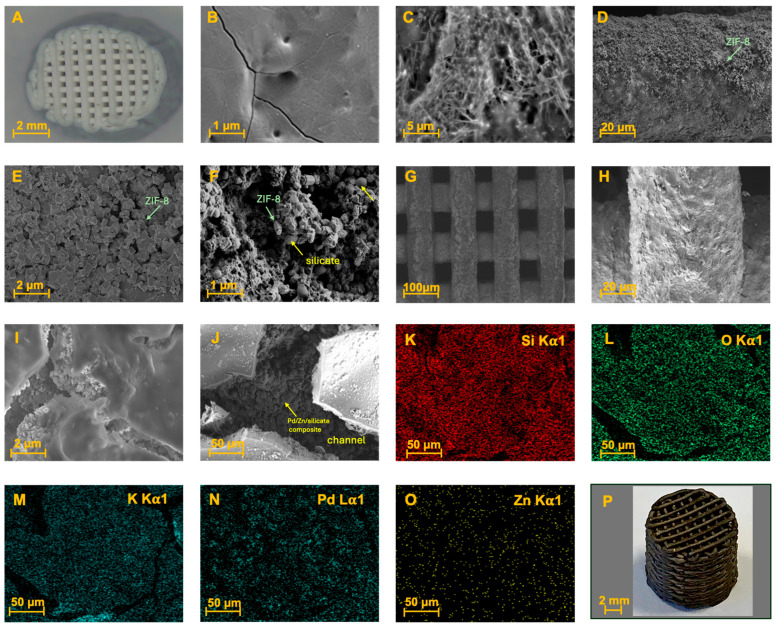
(**A**) Silica monolithic support obtained through 3D printing and sintering; (**B**) SEM: Smooth surface of the initial SiO_2_ sintered monolith; (**C**) SEM: Surface impregnation with Zn_2_(NO_3_)_2_ by SEA; (**D**,**E**) SEM of ZIF-8 growth on the monolith surface; (**F**) SEM: Impregnation with potassium silicate; (**G**,**H**) SEM: Detail of the monolith bars with the final composite after pyrolysis; (**I**,**J**) SEM: Details of the nanopores generated on the surface composite within the channels; (**K**–**O**) EDX mapping of the final composite; (**P**) Appearance of the final monolithic catalyst 3D-SiO_2_@Pd@K_2_SiO_3_.

**Figure 3:**
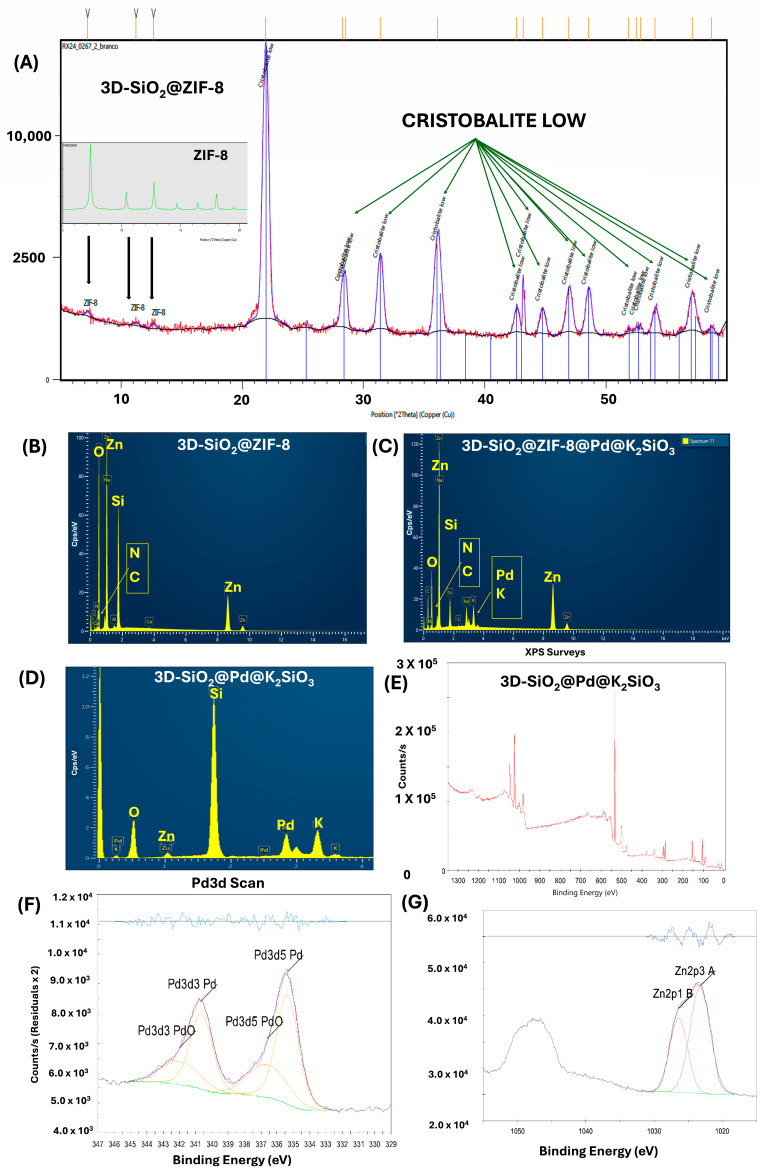
(**A**) XRD of 3D-SiO_2_@ZIF-8 and the ZIF-8 pattern; (**B**) EDX: elemental composition of the surface of the monolith functionalized with ZIF-8, 3D-SiO_2_@ZIF-8; (**C**) EDX of the surface of the monolith functionalized with ZIF-8, palladium, and potassium silicate, 3D-SiO_2_@ZIF-8@Pd@K_2_SiO_3_; (**D**) EDX of the surface of the final monolith 3D-SiO_2_@Pd@K_2_SiO_3_; (**E**) XPS full spectra of 3D-SiO_2_@Pd@K_2_SiO_3_; (**F**) XPS showing the Pd species in 3D-SiO_2_@Pd@K_2_SiO_3_; (**G**) XPS showing the Zn species in 3D-SiO_2_@Pd@K_2_SiO_3_.

The primary characterization process was conducted step-by-step after each chemical treatment applied on the starting silica monolith (8 mm diameter, 10 mm height, Figure 2A) using SEM, XRD, EDX and mapping techniques, as represented in Figure 2 and Figure 3.

The final system was also analyzed using XPS. Initially, the silica support surface (Figure 2A) appeared smooth, with a few superficial nanopores, and treatment with piranha solution did not produce significant differences, as observed using SEM (Figure 2B). The deposited crystals were likely hydrated Zn(NO_3_)_2_, as the acidic aqueous medium favors the stability of this salt in solution. Local supersaturation during the process could have led to crystallization on the silica surface. Alternatively, if the local pH had slightly changed (due to interactions with the silanol groups of silica), small, basic zinc phases such as Zn(OH)_2_ might have formed, although this is less common in acidic media. Furthermore, the detection of a small proportion of nitrogen using EDX (Figure 3B) confirms the presence of nitrate residues or related phases, highlighting the influence of salts in the MOF formation process (Figure 2C).

The formation of ZIF-8 crystals is clearly visible in the SEM images (Figure 2D,E). As can be observed in Figure 2E, the deposited ZIF-8 crystals have an average size of approx. 250 nm. Immersing the monolith in a potassium silicate solution generates a composite in which the ZIF-8 is encapsulated and interconnected by a uniform and compact layer of silicate. This cementing agent surrounds and interposes itself between the structure of the deposited MOF (Figure 2F). Subsequent thermal treatment at 400 °C produces micrometric-sized pores, as observed in Figure 2G–J.

EDX mapping analysis revealed the presence of the expected surface elements (Si, O, K, Zn, and Pd), shown in Figure 2K–O and Figure 3D. These elements are uniformly distributed, albeit at different concentrations. The final 3D-SiO_2_@Pd@K_2_SiO_3_ monolith exhibits an intense black color, resulting from the presence of palladium (0) species on its surface (Figure 2P). XRD analysis of the intermediate state of the monolith with surface-deposited ZIF-8 (corresponding to the states in Figure 2D,E) showed a diffractogram (Figure 3A) with the characteristic signals of the cristobalite support and other small peaks at low angles and a pair of intense peaks, which correspond to signals from ZIF-8. Given the monolithic structure and the surface-specific analysis, identifying the MOF’s crystalline structure proved challenging. Furthermore, the presence of ZIF-8 is confirmed by the detection of elements such as zinc, carbon, and nitrogen in the EDX spectrum (Figure 3B).

After drying, an aqueous solution of potassium silicate (30% *w*/*w*) was applied, generating a composite with silicate interposed within the ZIF-8 previously deposited on the monolith surface (Figure 2F), as confirmed using EDX (Figure 3C). Pyrolysis at 400 °C under nitrogen yielded a robust system with encapsulated Pd nanoparticles. The final surface comprised a potassium silicate layer with an underlying porous structure of zinc, silicon, oxygen, and palladium (Figure 2I–K). The absence of carbon in the final monolithic catalyst (EDX-Figure 3D) confirmed the removal of the organic ligand, potentially enhancing reactant flow through the catalytic system. EDX mapping showed a uniform distribution of Si, O, Zn, Pd, and K in the final composite.

The XPS analysis of the calcined monolith (Figure 3E–G) revealed that the sample primarily consisted of SiO_2_, as indicated by the high contributions of silicon (21.59%) and oxygen (43.87%). The peaks in the binding energy region of 102.6 eV (Si2p3) and 103.25 eV (Si2p1) corresponded to Si in the SiO_2_ matrix, confirming its dominant presence on the surface. The O1s peak at 532.43 eV is associated with oxides and organic components (SiO_2_), while the peak at 530.8 eV represents metal oxides. There were other elements like potassium (K), palladium (Pd), zinc (Zn). Palladium was present predominantly in its metallic state, Pd(0) (0.41%) (Pd3d5 at 335.37 eV), with a smaller fraction as oxidized Pd(II) (0.21%) (PdO at 336.59 eV), showing a Pd(0)/Pd(II) ratio of approximately 2:1, suggesting slight surface oxidation (Figure 3E,F) with a relatively low atomic contribution (0.41% and 0.21%, respectively). This suggests that Pd is well dispersed on the surface. The Zn2p3 and Zn2p1 peaks (1023.26 eV and 1026.41 eV) confirmed the incorporation of zinc on the surface. Zinc appeared as Zn(II) (8.48%), likely in the form of ZnO. Additionally, potassium (3.82%) was detected in small quantities. The K2p3 peak (293.59 eV) demonstrates the incorporation of potassium, detected in small quantities (3.82%), originating from the potassium silicate treatment (Figure 3G).

**Figure 4:**
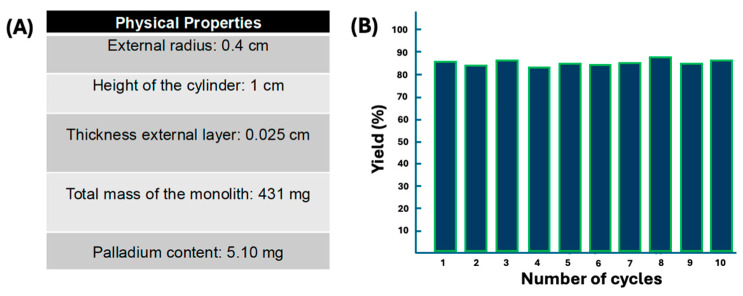
(**A**) Physical properties of the 3D-SiO_2_@Pd@K_2_SiO_3_ catalyst and (**B**) recyclability using the synthesis of **34** as a model reaction in ten reactions.

The elemental composition of 3D-SiO_2_@Pd@K_2_SiO_3_ was determined using EDX: O: 38.22%; Si: 21.72%; K: 4.87%; Zn: 35.07%; Pd: 10.88%. The content of the catalytically active species, Pd, was calculated based on the palladium surface percentage (10%) measured at different points on the catalyst surface via EDX. Considering that the thickness of the monolith’s surface layer is 100 μm and the total weight of the monolith 430 mg, the final content of palladium on the surface was estimated to be 5.2 mg (Figure 4A).

The overall porosity generated was determined using the mercury porosimetry technique, and the results are summarized in Table S1. The 3D-SiO_2_@Pd@K_2_SiO_3_ monolith, derived from the initial monolith through controlled surface growth of a MOF–silicate composite, exhibits significant enhancement in its porous properties despite undergoing a subsequent calcination process. While this pyrolysis step may have caused a slight loss of porosity, the final monolithic catalyst resulted in a remarkable increase in the porosity percentage with respect to the initial support, from 1.37% (initial 3D-silica support) to 9.12% (3D-SiO_2_@Pd@K_2_SiO_3_), along with substantial growth in the average pore diameter (from 0.28 μm to 9.84 μm).

This hierarchical porous network, now more open and accessible, improves the diffusion of reactants and products while maintaining the structural stability of the monolith. These features make the catalyst particularly effective for heterogeneous reactions with small volumes, performed in batch mode, where its small size and enhanced accessibility to active sites are crucial.

### 3.4. Results of Catalytic Activity

**Scheme 2:**
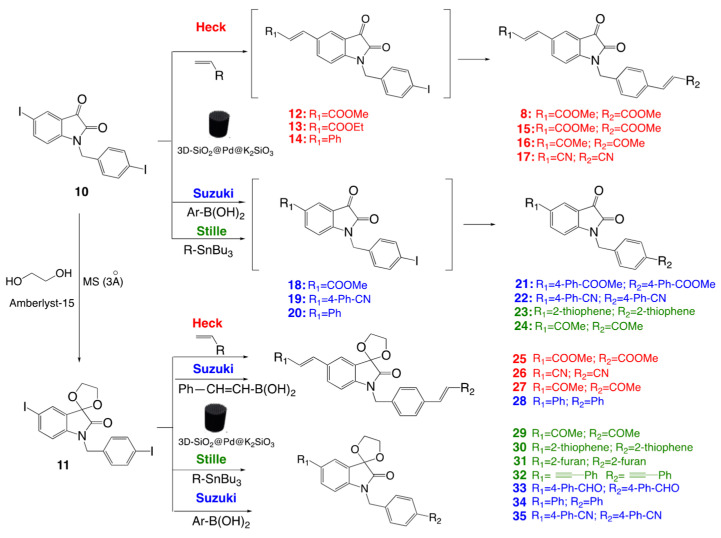
Synthetic route for the preparation of the compounds developed in this work using 3D-SiO_2_@Pd@K_2_SiO_3_ catalyst.

The synthetic route depicted in Scheme 2 relies on the use of two precursors and three Pd-methodologies (Heck, Stille, Suzuki). The Heck reaction was the main method for obtaining bis-alkenyl-isatins, while the Suzuki reaction was preferably used for the synthesis of bis-aryl-isatins. The Stille reaction was also tested for the synthesis of other diverse compounds, such as acetyl, alkynyl, and thienyl or furyl-isatins, the latter of which could also be prepared via the Suzuki reaction. Precursor **10**, previously prepared via benzylation of commercially available 5-iodoisatin **9** [19], was further processed by protecting its C-3 with a cyclic ketal to yield the ketal compound **11**, serving as the starting point for other final analogues. Initial attempts to conduct the reaction of **10** with conventional homogeneous catalysts, such as palladium acetate or dichlorobis(triphenylphosphine)-Pd(II), at various temperatures resulted in low yields (see Table S2) and the formation of significant by-products, likely metalates. These by-products arose due to the affinity of homogeneous palladium species for coordination with the isatin’s carbonyl groups at C-2 and C-3 [60]. This issue was exacerbated at higher temperatures (90–100 °C), reducing the overall yields in the Heck and Suzuki reactions. Therefore, the heterogeneous 3D-SiO_2_@Pd@K_2_SiO_3_ catalyst, featuring immobilized palladium species, was used.

The catalyst demonstrated efficiency and reusability over multiple reaction cycles and confirmed the superior reactivity of precursor **11** over **10** in the PCCCRs explored. The Heck reaction of **10** with different alkenes yielded monosubstituted products **12**, **13**, and **14**, with styrene producing monosubstituted compound **14**, in good yields (see Table 1). Under controlled reaction conditions, using triethylamine as the base and dimethylformamide as the solvent (75 °C), these iodinated intermediates (adducts) could be isolated through careful optimization of the temperature and reaction time.

Using an excess of alkene and extended reaction times, bi-substituted isatins **8**, **15**, **16** and **17** were obtained—without isolation of intermediates—in good yields (see Table 1). Suzuki reactions produced arylated products **18**–**20** and bis arylated derivatives **21** and **22** using special conditions (Method A, using TEA and anhydrous DMF) to prevent methyl ester or cyano hydrolysis.

The Stille reaction from **10** provided compounds **23** and **24** (in toluene), the latter obtained by treating an intermediate bis-vinyl ether (not isolated) with 3N HCl for 24 h. For isatin **11**, containing a cyclic acetal at position 3, rapid formation of disubstituted products was observed. Although monosubstituted intermediates could be detected via TLC, they were quickly converted to the disubstituted products. Consequently, the Heck reactions yielded compounds **25**–**27**, and the Stille ones yielded compounds **29**–**32** under analogous conditions used for the 3-carbonyl series. The Suzuki reactions produced **28**, **33**, **34**, and **35** via Method B, using potassium carbonate as a base in dimethoxyethane/water in good yields (see Table 1).

Collectively, the 3D-SiO_2_@Pd@K_2_SiO_3_ catalyst addresses a chemical challenge, as all attempts to perform these transformations with the previously described homogeneous reagents (palladium acetate, dichloro(bistriphenylphosphine)-palladium (II), Pd-C) were less than satisfactory, yielding significant impurities in the final mixtures (see Table S2). However, using 3D-SiO_2_@Pd@K_2_SiO_3_, the reactions proceeded much more cleanly, indicating a certain selectivity for C–C coupling rather than palladium interacting with the bi-carbonyl system. In addition, the reusability was also checked. The 3D-SiO_2_@Pd@K_2_SiO_3_ catalyst was reused up to 10 times without any apparent loss of activity (Figure 4B). Furthermore, preliminary *hot filtration tests* revealed the absence of metal species in the solution, a result that was confirmed via ICP analysis of extracts of the reactions, which did not detect any trace of palladium in the final reaction mixtures.

### 3.5. Cytotoxic Activity of Substituted Isatins in HeLa, MDA-MB-231, and MCF-7 Cell Lines

**Figure 5:**
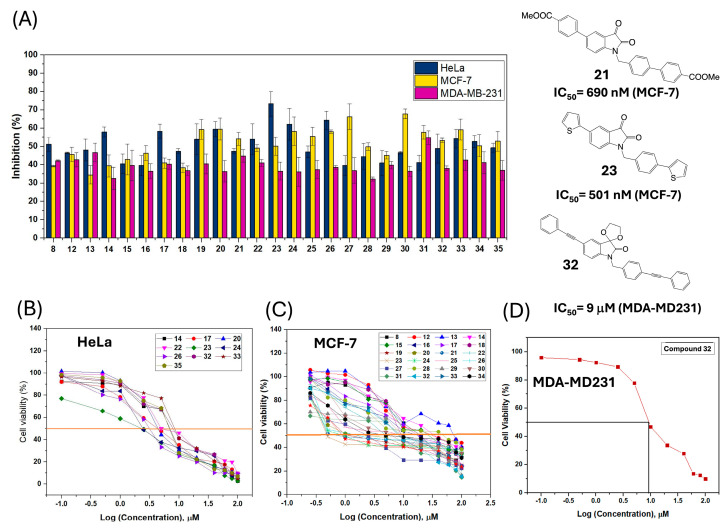
Cytotoxicity of isatins **8**–**35**: (**A**) Inhibition (**%**) at 10 μM in HeLA, MCF-7, MDA-MB231 and best prototypes; (**B**) Inhibition dose/response curves in HeLa; (**C**) in MCF-7; (**D**) in MDA-MB231 for compound **32**.

Figure 5A illustrates the percentage of cell growth inhibition in the three tumor cell lines studied after 24 h of incubation. Many of the synthesized compounds showed cytotoxicity above 50% at the initial screening drug concentration of 10 mM in both the HeLa and MCF-7 lines. However, for the triple-negative breast cancer MDA-MB-231 cell line, only compound **32** showed significant cytotoxic activity (IC_50_ = 9.1 μM). Figure 5B–D display the inhibition curves for each compound in each cell line. Table 2 presents the IC_50_ values for each compound for each cell line.

Doxorubicin and sunitinib were used as the standard drug for comparison in the HeLa, MCF-7, and MDA-MB231 cells. From the inhibitory activity data obtained for this series of compounds in the HeLa cells, the results of compounds **23** (IC_50_ = 2.4 μM) and **24** (IC_50_ = 2.3 μM) stand out, respectively. Interestingly, although they do not surpass our previous methyl diester prototype (**8**) in this cell line (IC_50_ =1 μM), it was confirmed that the presence of EWGs on alkenes or directly on the isatin and benzyl ring (**24**) generally enhanced cytotoxic activity, preferably keeping the carbonyl at position 3 intact. However, this is not essential, as the presence of a cyclic acetal can also result in cytotoxicity (**26**) (IC_50_ = 3.1 μM). Additionally, the presence of aromatic rings appears to be suitable for HeLa survival, with thiophene **23** demonstrating interesting activity (IC_50_ = 2.4 μM); the same activity of the reference drug doxorubicin (IC_50_ = 2.4 μM).

In the MCF-7 cell line, compounds featuring cyano groups—**19**, **22**, **26**, and **35**—exhibited remarkable cytotoxic activity, with IC_50_ values ranging from 1 to 2 μM. Notably, two of these compounds (**26** and **35**) also incorporated a cyclic ketal moiety at C-3, highlighting a potential structural feature contributing to their potency. These results could be related to those obtained by Stanton regarding the cytotoxicity of 2-phenylacrylonitriles in the same cell line [61]. On the other hand, the bis-alkenyl-isatins did not exhibit potent cytotoxic activity in the MCF-7 cell line. However, to our satisfaction, the bis-aryl derivatives, particularly the diester **21** (IC_50_ = 0.69 μM) and especially the bis-thienyl **23** (IC_50_ = 0.50 μM), which is fourteen times more potent than doxorubicin (IC_50_ = 7.3 μM) and sunitinib (IC_50_ = 10.7 μM), as seen in Table 2, did show outstanding therapeutic activity. This confirms that the EWGs in C-5 and the benzyl group are not strictly necessary in the positions studied. Collectively, these data reinforce the idea that the presence of one or two aromatic rings in the proposed positions is preferred for cytotoxic activity in MCF-7. Interestingly, the diketone **24** also showed potent activity in this line (IC_50_ = 0.88 μM). These three derivatives (**21**, **23**, **24**) share an isatin structure with a carbonyl group at C-3. Compounds **23** and **24** are promising new hits, as they also show significant cytotoxicity in HeLa cells. Finally, compound **32** proved to be the only one of interest in the triple-resistant line MDA-MB-231 (IC_50_ = 9.1 μM), notably containing an alkyne group and a cyclic acetal in its structure. Its structure, containing a triple bond, could be related to drugs like ponatinib (IC_50_= 1.41 μM for MDA-MB-231 and 4.59 μM for MCF-7) [62] and its mechanism.

**Table 2:** Results of cytotoxic activity of the diversely substituted 1-benzylindolin-2-ones.

Compound	HeLa	MCF-7	MDA-MB231
	IC_50_ (μM) ^a^		
**8**	1.0 [19] ^b^	19.9	17.8
**12**	10.8 [19] ^b^	13.6	15.7
**13**	20.0	33.1	19.8
**14**	6.9	28.1	34.3
**15**	12.4	26.3	14.3
**16**	11.2	13.8	17.8
**17**	4.3	16.5	16.9
**18**	9.3	9.2	23.8
**19**	9.9	1.1	20.7
**20**	4.3	1.8	14.9
**21**	12.3	0.6 (690 nM)	12.3
**22**	5.0	1.6	11.7
**23**	2.4	0.5 (501 nM)	15.8
**24**	2.3	0.8 (880 nM)	14.8
**25**	11.9	8.2	12.3
**26**	3.1	1.6	13.9
**27**	15.5	2.4	12.7
**28**	13.7	12.6	20.8
**29**	17.5	9.9	16.8
**30**	14.7	6.3	17.9
**31**	13.5	8.5	11.9
**32**	7.8	8.1	9.1
**33**	8.4	9.1	11.9
**34**	9.7	4.7	12.7
**35**	7.8	1.2	14.9
**Doxorubicin**	2.4 [63] ^b^	7.3 [13] ^b^	9.6 [64] ^b^
**Sunitinib**	2.4 [63] ^b^	10.7 [65] ^b^	10 (57%) [66] ^b,c^

^a^ Results after 24 h. ^b^ Results previously reported. ^c^ (% inhibition) at 10 mM.

## 4. Conclusions

This interdisciplinary study introduces a novel approach for stabilizing and functionalizing MOFs on 3D-printed ceramic supports, broadening the toolkit for heterogeneous catalyst fabrication and the synthesis of biologically relevant compounds. The surface growth of ZIF-8 on a silica sintered monolith, followed by potassium silicate treatment and pyrolysis, resulted in a porous and robust system that remained effective and stable under various catalytic conditions.

The catalyst exhibited excellent performance in the parallel synthesis of small biomolecules with potential anticancer activity. Notably, it outperformed both homogeneous and heterogeneous counterparts, demonstrating superior efficiency in diverse reaction conditions (Heck, Suzuki, and Stille reactions). Additionally, the catalyst maintained its activity and structural integrity over at least 10 cycles, with no apparent loss of performance or leaching.

Several of the compound prototypes synthesized in this study displayed remarkable cytotoxicity against HeLa and MDA-MB-231 cell lines, with particularly potent antiproliferative effects against MCF-7 cells. Compounds **21**, **23**, and **24** exhibited exceptional inhibition of cell growth, with nanomolar-range activity in MCF-7 cells, while also demonstrating superior effectiveness in HeLa cells compared to previous isatin-based reference ligands, as well as standard anticancer drugs such as doxorubicin and sunitinib. Notably, compound **25** emerged as the most potent against MDA-MB231 cells.

In summary, this work presents a novel approach for synthesizing porous heterogeneous catalysts from sintered surfaces. Additionally, the bi-substituted *N*-benzylisatins obtained in this study show promise as potential anticancer therapeutics. Ongoing studies aim to elucidate both the selectivity and the precise mechanisms underlying the antiproliferative activity of these compounds.

## Figures and Tables

**Table 1:** Structure of the diversely substituted 1-benzylindolin-2-ones and 1-benzylspiro[indoline-3,2′-[1,3]dioxolan]-2-ones obtained using 3D-SiO_2_@Pd@K_2_SiO_3_ as a catalyst.

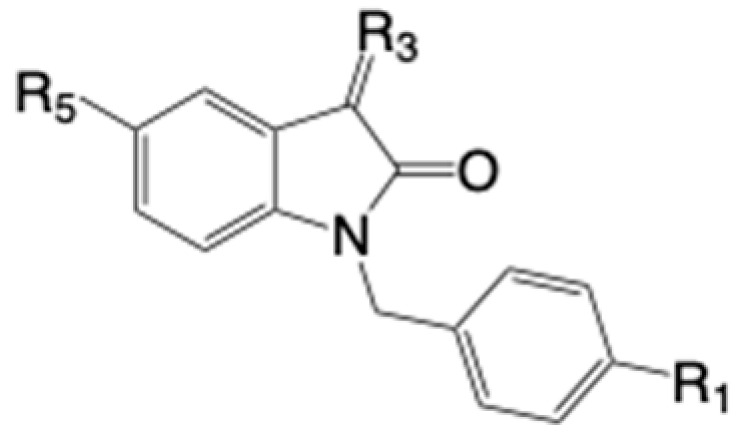					
Compound	R_1_	R_3_	R_5_	Method	Yield (%)
**8**	CH=CH-COOMe	O	CH=CH-COOMe	HECK ^1^	65
**12**	I	O	CH=CH-COOMe	HECK ^1,2^	50
**13**	I	O	CH=CH-COOEt	HECK ^1,2^	40
**14**	I	O	CH=CH-Ph	HECK ^1^	75
**15**	CH=CH-COOEt	O	CH=CH-COOEt	HECK ^1^	78
**16**	CH=CH-COMe	O	CH=CH-COMe	HECK ^1^	67
**17**	CH=CH-CN	O	CH=CH-CN	HECK ^1^	66
**18**	I	O	4-Ph-COOMe	SUZUKI ^1,2,3^	55
**19**	I	O	4-Ph-CN	SUZUKI ^1,2,3^	58
**20**	I	O	Ph	SUZUKI ^1,2,3^	59
**21**	4-Ph-COOMe	O	4-Ph-COOMe	SUZUKI ^1,3^	78
**22**	4-Ph-CN	O	4-Ph-CN	SUZUKI ^1,3^	35
**23**	2-thiophene	O	2-thiophene	STILLE ^1^	72
**24**	COCH_3_	O	COCH_3_	STILLE ^1^	76
**25**	CH=CH-COOMe	O-CH_2_CH_2_O	CH=CH-COOMe	HECK ^4^	80
**26**	CH=CH-CN	O-CH_2_CH_2_O	CH=CH-CN	HECK ^4^	85
**27**	CH=CH-COMe	O-CH_2_CH_2_O	CH=CH-COMe	HECK ^4^	87
**28**	CH=CH-Ph	O-CH_2_CH_2_O	CH=CH-Ph	SUZUKI ^4,5^	85
**29**	COCH_3_	O-CH_2_CH_2_O	COCH_3_	STILLE ^4^	85
**30**	2-thiophene	O-CH_2_CH_2_O	2-thiophene	STILLE ^4^	84
**31**	2-furan	O-CH_2_CH_2_O	2-furan	STILLE ^4^	78
**32**	C≡C-Ph	O-CH_2_CH_2_O	CH≡C-Ph	STILLE ^4^	79
**33**	4-Ph-CHO	O-CH_2_CH_2_O	4-Ph-CHO	SUZUKI ^4,5^	66
**34**	Ph	O-CH_2_CH_2_O	Ph	SUZUKI ^4,5^	88
**35**	4-Ph-CN	O-CH_2_CH_2_O	4-Ph-CN	SUZUKI ^4,5^	70

^1^ Compound **10** was used as the starting product. ^2^ Concomitant bi-substituted partner was also formed. ^3^ Method A was used. ^4^ Compound **11** was used as the starting product. ^5^ Method B was used.

## Data Availability

The data supporting this article have been included as part of the Supplementary Information.

## Supplementary Materials

The following supporting information can be downloaded at: https://www.mdpi.com/article/10.3390/pharmaceutics17040505/s1, ^1^H NMR, ^13^C NMR, mass spectra and high-resolution mass spectrometry (HRMS) data for all final compounds described in this work. Table S1: Porosimetry of initial support and final monolithic catalyst; Table S2: Comparative performance of 3D-SiO_2_@Pd@K_2_SiO_3_ with other catalysts.
