# Supplementary material for: Developing Heterogeneous Porous 3D-Printed SiO2-Pd-K2SiO3 Monolithic Catalyst via Surface MOF Growth and Pyrolysis for the Synthesis of Antitumoral Isatins
Source: Pharmaceutics. 2025 Apr 11;17(4):505. doi: 10.3390/pharmaceutics17040505 (PMC12030608; doi:10.3390/pharmaceutics17040505)
Supplement: Supplementary file 1 [file pharmaceutics-17-00505-s001.zip › pharmaceutics-3564260-supplementary.pdf]

Supplementary Information

Developing Heterogeneous Porous 3D-Printed SiO_2-Pd-K_2SiO_3 Monolithic Catalyst via Surface MOF Growth and Pyrolysis for the Synthesis of Antitumoral Isatins

Alexandrina Druta [1,2], Rania Bouhmala [1], Teqwa Ragdi [1], Mariangel Luna [2,3], Manuel Bañobre-López [4], Christian F. Masaguer [1], Manuel Amorín [1], Silvia Barbosa [2,3], Pablo Taboada [2,3,*] and Alberto Coelho [1,2,3,*]

[1] Department of Organic Chemistry, Faculty of Pharmacy, University of Santiago de Compostela, 15782 Santiago de Compostela, Spain

[2] Colloids and Polymers Physics Group, Department of Physics of Particles, Faculty of Physics, University of Santiago de Compostela, 15782 Santiago de Compostela, Spain

[3] Institute of Materials-USC (IMATUS), University of Santiago de Compostela, 15782 Santiago de Compostela, Spain

[4] International Iberian Nanotechnology Laboratory. Avenida Mestre José Veiga s/n., 4715-330 Braga, Portugal.

* Author to whom correspondence should be addressed

Table of Contents:

1. SPECTROSCOPIC DATA OF THE RELATED COMPOUNDS..........................S1
2. ^{1}H-NMR, ^{13}C-NMR, MASAS, AND HIGH-RESOLUTION MASAS SPECTRA* OF THE RELATED COMPOUNDS..S9
3. TABLE S1. POROSIMETRY OF INITIAL SUPPORT AND FINAL MONOLITHIC CATALYST...S86
4. TABLE S2. COMPARATIVE PERFORMANCE OF 3D-SIO_2@Pd@K_2SIO_3 WITH OTHER CATALYSTS..S86

*: HIGH-RESOLUTION MASAS SPECTRA (HRMS): In the list of exact masses, the measurement error of the peak of interest is shown, along with the average error for the analyte cluster as a whole and a statistical value, "mSigma," which assesses the reliability of the measurement. This evaluation goes beyond the error in ppm by also considering the height and distribution of the peaks compared to the theoretical profile. A lower mSigma value indicates a better fit to the theoretical profile, with optimal values typically being below 50.

1. SPECTROSCOPIC DATA OF THE RELATED COMPOUNDS

5-iodo-1-(4-iodobenzyl)indoline-2,3-dione, (10): Orange solid. M.P.= 200–201°C. ^{1}H-NMR ($CDCl_3$, 500 MHz): 7.88 (s, 1H), 7.80 (m, 1H), 7.65 (d *J* = 8.2 Hz, 2H), 7.05 (d *J* = 8.2 Hz, 2H), 6.55 (d *J* = 8.2 Hz, 1H), 4.85 (s, 2H). ^{13}C NMR ($CDCl_3$, 125 MHz): 181.7, 157.2, 149.6, 146.4, 138.3, 134.0, 133.7, 129.2, 119.2, 112.9, 93.9, 86.4, 43.6. MS (ESI) [M + H]: 489. [ref. 19]

5-iodo-1-(4-iodobenzyl)spiro[indoline-3,2'-[1,3]dioxolan]-2-one (11): Yellow solid. M.P.: 147-148°C. ^{1}H-NMR ($CDCl_3$, 75 MHz) δ: 7.93 (d, *J* = 8.4 Hz, 3H), 7.84 (dd, *J* = 8.2, 1.8 Hz, 1H), 7.27 (d, *J* = 8.4 Hz, 2H), 6.66 (d, *J* = 8.3 Hz, 1H), 5.00 (s, 2H), 4.87 (m, 2H), 4.61 (m, 2H). ^{13}C NMR ($CDCl_3$, 125 MHz): 172.7, 143.3, 140.4, 138.1, 134.6, 134.0, 129.1, 126.3, 111.6, 101.7, 93.5, 86.0, 66.2, 43.1. MS-APCI [M+H]: 533.

methyl (*E*)-3-(1-(4-iodobenzyl)-2,3-dioxoindolin-5-yl)acrylate (12): Orange solid. M.P.: 136-138°C. ^{1}H-NMR ($CDCl_3$, 500 MHz) δ: 7.79 (d, *J* = 1.7 Hz, 1H), 7.69 (d, *J* = 8.3 Hz, 1H), 7.69-7.60 (m, 2H), 7.58 (d, *J* = 16.0 Hz, 1H), 7.11 (m, 1H), 7.08 (d, *J* = 8.4 Hz, 1H), 6.78 (d, *J* = 8.2 Hz, 1H), 6.36 (d, *J* = 16.0 Hz, 1H). , 4.89 (s, 2H), 3.80 (s, 3H). ^{13}C NMR ($CDCl_3$, 125 MHz): 182.3, 166.9, 158.1, 151.7, 142.3, 138.3, 138.0, 133.8, 130.8, 129.3, 124.2, 118.4, 118.1, 111.2, 93.9, 51.9, 43.8. MS-APCI [M+H]: 448. HRMS (APCI) m/z: $[M+H]^+$ calculated for $C_{19}H_{15}INO_4$, 448.0040; found, 448.0042.

ethyl (*E*)-3-(1-(4-iodobenzyl)-2,3-dioxoindolin-5-yl)acrylate (13): Orange solid. M.P.: 125-126 ^{1}H-NMR ($CDCl_3$, 500 MHz) δ: 7.75-7.79 (m, 5H), 7.08 (d, *J* = 8.1 Hz, 2H), 6.77 (d, *J* = 8.2 Hz, 1H), 6.35 (d, *J* = 16.0 Hz, 1H), 4.88 (s, 2H), 4.25 (q, *J* = 7.1 Hz, 2H), 1.32 (t, *J* = 7.1 Hz, 3H). ^{13}C NMR ($CDCl_3$, 125 MHz): 186.0, 166.5, 157.8, 151.1, 142.1, 138.4, 138.0, 134.0, 131.0,129.4, 124.3, 119.0, 118.1, 111.3, 94.9, 60.9, 43.9,

14.4. MS-APCI [M+H]: 462. HRMS (APCI) m/z: $[M+H]^+$ calculated for $C_{20}H_{17}INO_4$, 462.0197; found, 462.0197.

(*E*)-1-(4-iodobenzyl)-5-styrylindoline-2,3-dione (14): Orange oil. ^1H-NMR ($CDCl_3$, 500 MHz) δ: 7.1-7.70 (m, 12H), 7.80 (s, 1H), 6.73 (d, *J* = 8.2 Hz, 1H), 4.88 (s, 2H). ^{13}C NMR ($CDCl_3$, 125 MHz): 183.2, 158.3, 149.3, 147.1, 138.6, 137.6, 136.8, 135.6, 134.2, 129.5, 129.3, 128.3, 126.5, 124.2, 118.3, 112.2, 111.2, 93.9, 43.4. MS-APCI [M+H]: 466. HRMS (APCI) m/z: $[M+H]^+$ calculated for $C_{23}H_{17}INO_2$, 466.0299; found, 466.0300.

methyl (*E*)-3-(4-((5-((*E*)-3-methoxy-3-oxoprop-1-en-1-yl)-2,3-dioxoindolin-1-yl)-methyl)-phenyl)acrylate (8): Orange solid. M.P.: 188–190 ºC. ^1H-NMR ($CDCl_3$, 500 MHz) δ: 7.80-7.50 (m, 6H), 7.40 (br, 1H), 7.35 (d, *J* = 8.0 Hz, 1H), 6.79 (d, *J* = 8.2 Hz, 1H), 6.44-6.33 (m, 2H), 4.96 (s, 2H), 3.80 (s, 6H). ^{13}C NMR ($CDCl_3$, 125 MHz): 182.4, 167.2, 166.9, 158.1, 151.2, 143.7, 142.3, 138.0, 136.2, 134.6, 130.8, 128.8, 127.9, 124.3, 118.6, 118.4, 118.1, 111.2, 51.9, 51.8, 43.9. MS-APCI [M+H]: 406.0. HRMS (APCI) m/z: $[M+H]^+$ calculated for $C_{23}H_{20}NO_6$, 406.1285; found, 406.1288.

ethyl(*E*)-3-(4-((5-((*E*)-3-ethoxy-3-oxoprop-1-en-1-yl)-2,3-dioxoindolin-1-yl) me-thyl) phenyl)-acrylate (15): Orange solid. M.P.: 140-142°C ^1H-NMR ($CDCl_3$, 500 MHz) δ: 7.80 (s, 1H), 7.50-7.70 (m, 5H), 7.36 (m, 2H), 6.79 (d, *J* = 8.2 Hz, 1H), 6.38 (dd, *J* = 8.2, 16.0 Hz, 2H), 4.96 (s, 2H), 4.48-4.13 (m, 4H),1.32 (m, 6H). ^{13}C NMR ($CDCl_3$, 125 MHz): 182.5, 166.8, 166.5, 158.2, 151.3, 143.5, 142.1, 138.1, 136.3, 134.8, 131.0, 128.8, 127.0, 124.3, 119.2, 118.2, 111.3, 60.9, 44.1, 13.5. MS-APCI [M+H]: 434. HRMS (APCI) m/z: $[M+H]^+$ calculated for $C_{25}H_{24}NO_6$, 434.1598; found, 434.1603.

5-((*E*)-3-oxobut-1-en-1-yl)-1-(4-((*E*)-3-oxobut-1-en-1-yl)benzyl)indoline-2,3-dione (16): Orange oil. ^{1}H-NMR ($CDCl_3$, 500 MHz) δ: 7.59 (s, 1H), 7.26-7.49 (m, 7H), 6.56-6.66 (m, 3H), 4.82 (s, 2H), 2.32 (s, 3H), 2.30 (s, 3H). ^{13}C NMR ($CDCl_3$, 125 MHz): 198.1, 197.9, 181.0, 171.1, 144.0, 143.1, 142.5, 140.5, 137.1, 134.5, 131.5, 128.7, 127.6, 127.3, 126.2, 125.1, 124.1, 110.1 52.1, 27.5. MS-APCI [M+H]: 374. HRMS (APCI) m/z: $[M+H]^+$ calculated for $C_{23}H_{20}NO_4$, 374.1387; found: 374.1387.

(*E*)-3-(4-((5-((*E*)-2-cyanovinyl)-2,3-dioxoindolin-1-yl)methyl)phenyl)acrylonitrile (17): White solid. M.P.: 135-137°C ^{1}H-NMR ($CDCl_3$, 500 MHz) δ: 8.31 (s, 1H), 8.07 (d, *J* = 8.2 Hz, 1H), 7.75 (s, 1H), 7.54-7.63 (m, 5H), 6.88 (d, *J* = 8.2 Hz, 1H), 6.02 (dd, *J* = 8.0 Hz, 2H), 5.08 (s, 2H). ^{13}C NMR ($CDCl_3$, 125 MHz): 175.1, 146.6, 150.1, 146.6, 144.2, 137.1, 132.3, 130.7, 128.5, 128.1, 126.9, 123.1, 121.2, 116.9, 108.6, 95.9, 94.1, 92.7, 42.7. MS-APCI [M+H]: 340. HRMS (APCI) m/z: $[M+H]^+$ calculated for $C_{21}H_{14}N_3O_2$, 340.1081; found: 340.1078.

methyl 4-(1-(4-iodobenzyl)-2,3-dioxoindolin-5-yl)benzoate (18): Orange solid. M.P.: 192-194°C ^{1}H-NMR ($CDCl_3$, 500 MHz) δ: 8.10 (d, *J* = 8.4 Hz, 2H), 7.88 (s, 1H), 7.75 (dd, *J* = 8.2, 2.0 Hz, 1H), 7.70 (d, *J* = 8.3 Hz, 2H), 7.56 (d, *J* = 8.3 Hz, 2H), 7.11 (d, *J* = 8.1 Hz, 2H), 6.84 (d, *J* = 8.2 Hz, 1H). 4.90 (s,2H), 3.92 (s,3H). ^{13}C NMR ($CDCl_3$, 125 MHz): 182.9, 166.7, 158.3, 150.1, 143.2, 138.4, 137.0, 136.5, 134.2, 130.5, 129.7, 129.4, 126.6, 124.2, 118.3, 11.4, 94.0, 52.3, 43.8. MS-APCI [M+H]: 498. HRMS (APCI) m/z: $[M+H]^+$ calculated for $C_{23}H_{17}NIO_4$, 498.0197; found: 498.0200.

4-(1-(4-iodobenzyl)-2,3-dioxoindolin-5-yl)benzonitrile (19): Orange solid. M.P.: 290-292°C. ^{1}H-NMR ($CDCl_3$, 500 MHz) δ: 7.88 (s, 1H), 7.75-7.65 (m, 7H), 7.50 (d, *J* = 8.1 Hz, 2H), 6.85 (d, *J* = 8.2 Hz, 1H). 5.05 (s,2H). ^{13}C NMR ($CDCl_3$, 125 MHz): 183.1, 158.2, 151.9, 145.2, 143.9, 139.1, 137.5, 136.1, 135.1, 133.6, 133.5, 128.7, 128.4,

127.6, 124.5, 118.1, 111.5, 43.9. MS-APCI [M+H]: 465. HRMS (APCI) m/z: $[M+H]^+$ calculated for $C_{22}H_{14}IN_2O_2$, 465.0095; found: 465.0098.

1-(4-iodobenzyl)-5-phenylindoline-2,3-dione (20): Orange solid. M.P.: 146-148°C. ^{1}H-NMR ($CDCl_3$, 500 MHz) δ: 8.30 (s, 1H), 8.10-8.20 (m, 2H), 7.75-8.10 (m, 6H), 7.65 (d, *J* = 8.2 Hz, 2H), 7.25 (d, *J* = 8.2 Hz, 1H), 5.30 (s, 2H). ^{13}C NMR ($CDCl_3$, 125 MHz): 183.1, 158.4, 149.4, 138.9, 137.7, 136.9, 135.7, 134.3, 129.4, 129.2, 128.1, 126.6, 124.1, 118.2, 111.2, 93.9, 43.8. MS-APCI [M+H]: 440. HRMS (APCI) m/z: $[M+H]^+$ calculated for $C_{21}H_{15}NIO_2$, 440.0142; found: 440.0144.

methyl 4'-[(5-(4-(methoxycarbonyl)phenyl)-2,3-dioxoindolin-1-yl)-methyl]-[1,1'-biphenyl]-4-carboxylate (21): Orange solid. M.P.: 276-278°C. ^{1}H-NMR ($CDCl_3$, 500 MHz) δ: 8.13 – 7.99 (m, 3H), 7.88 (s,1H), 7.76 (dd, *J* = 8.2, 1.9 Hz, 1H), 7.61 (d, *J* = 8.1 Hz, 6H), 7.54, (d, *J* = 8.0 Hz, 2H), 7.45 (d, *J* = 8.0 Hz, 1H), 6.91 (d, *J* = 8.2 Hz, 1H), 5.02 (s, 2H), 3.92 (s, 6H). ^{13}C NMR ($CDCl_3$, 125 MHz): 207.0, 183.1, 166.9, 166.7, 158.4, 150.3, 144.7, 143.2, 140.1, 137.0, 136.3, 134.4, 130.4, 130.2, 129.6, 129.3, 128.2, 128.1, 127.0, 126.5, 124.1, 111.5, 52.3, 52.2, 44.0. MS-APCI [M+H]: 506. HRMS (APCI) m/z: $[M+H]^+$ calculated for $C_{31}H_{24}NO_6$, 506.1598; found: 506.1599.

4'-((5-(4-cyanophenyl)-2,3-dioxoindolin-1-yl)methyl)-[1,1'-biphenyl]-4-carbonitrile (22): Orange solid. M.P.: 313-315°C. ^{1}H-NMR ($CDCl_3$, 500 MHz) δ: 7.88 (s, 1H), 7.60-7.75 (m, 12H), 7.48 (d, *J* = 8.3 Hz, 1H), 6.94 (d, *J* = 8.3 Hz, 1H), 5.04 (s, 2H). ^{13}C NMR ($CDCl_3$, 125 MHz): 182.8, 158.3, 150.6, 144.7, 143.3, 139.4, 137.0, 135.5, 134.9, 133.0, 132.8, 128.4, 128.1, 127.8, 127.3, 124.2, 118.8, 118.6, 118.4, 118.9, 118.6, 118.5, 44.0. MS-APCI [M+H]: 440. HRMS (APCI) m/z: $[M+H]^+$ calculated for $C_{29}H_{18}N_3O_2$, 440.1394; found: 440.1394.

5-(thiophen-2-yl)-1-(4-(thiophen-2-yl)benzyl)indoline-2,3-dione (23): Orange solid. M.P.: 109-111°C. ^{1}H-NMR ($CDCl_3$, 500 MHz) δ: 7.88 (s, 1H), 7.73-7.75 (m, 1H), 7.62-7.64 (m, 2H), 7.26-7.40 (m, 6H), 7.10 (m, 2H), 6.85 (d, *J* = 8.2 Hz, 1H), 4.99 (s, 2H). ^{13}C NMR ($CDCl_3$, 125 MHz): 183.2, 158.3, 149.4, 143.5, 142.0, 135.4, 134.6, 133.5, 131.0, 128.4, 128.2, 128.1, 126.5, 125.5, 125.3, 123.6, 123.5, 122.6, 118.1, 111.5, 44.0. MS-APCI [M+H]: 402. HRMS (APCI) m/z: $[M+H]^+$ calculated for $C_{23}H_{16}NO_2S_2$, 402.0617; found: 402.0622.

5-acetyl-1-(4-acetylbenzyl)indoline-2,3-dione (24): Orange solid. M.P.: 190-191°C. ^{1}H-NMR ($CDCl_3$, 500 MHz) δ: 8.18 (s, 2H), 7.93 (d, *J* = 8.1 Hz, 2H), 7.42 (d, *J* = 8.1 Hz, 2H), 6.84 (d, *J* = 8.2 Hz, 1H), 5.02 (s, 2H), 2.61 (s, 6H). ^{13}C NMR ($CDCl_3$, 125 MHz): 197.7, 196.0, 179.7, 173.7, 147.6, 140.0, 137.5, 136.5, 133.1, 129.2, 127.2, 125.3, 124.3, 108.9, 101.8, 42.5, 26.3, 26.2. MS-APCI [M+H]: 322. HRMS (APCI) m/z: $[M+H]^+$ calculated for $C_{19}H_{16}NO_4$, 322.1074; found: 322.1071.

Methyl (*E*)-3-(4-((5-((*E*)-3-methoxy-3-oxoprop-1-en-1-yl)-2-oxospiro[indoline-3,2'-[1,3]dioxolan]-1-yl)methyl)phenyl)acrylate (25): White solid. M.P.: 200-202°C. ^{1}H-NMR ($CDCl_3$, 500 MHz) δ: 7.70 (s, 1H), 7.66 (s, 1H), 7.60 (d, *J* = 8.1 Hz, 1H), 7.40-7.50 (m, 2H), 7.31-7.35 (m, 1H), 7.25-7.35 (m, 2H), 6.65 (d, *J* = 8.1 Hz, 1H), 6.37 (dd, *J* = 8.1, 16.0 Hz, 2H), 4.80 (s, 2H), 4.64 (m, 2H), 4.38 (m, 2H), 3.80 (s, 3H), 3.78 (s, 3H). ^{13}C NMR ($CDCl_3$, 125 MHz): 175.1, 168.1, 145.3, 144.1, 143.8, 137.1, 134.2, 132.7, 131.1, 128.7, 127.8, 124.7, 124.1, 118.3, 116.9, 109.9, 101.9, 66.1, 51.8, 43.4. MS-APCI [M+H]: 449. HRMS (APCI) m/z: $[M+H]^+$ calculated for $C_{25}H_{24}NO_7$, 449.1547; found: 449.1548.

(*E*)-3-(4-((5-((*E*)-2-cyanovinyl)-2,3-dioxoindolin-1-yl)methyl)phenyl)acrylonitrile (26): White oil. ^{1}H-NMR ($CDCl_3$, 500 MHz) δ: 8.31 (s, 1H), 8.07 (s, 1H), 7.75 (s, 1H),

7.54-7.63 (m, 5H), 6.88 (d, *J* = 8.2 Hz, 1H), 6.02 (q, *J* = 16.0 Hz, 2H), 5.08 (s, 2H), 4.86 (m, 2H), 4.62 (m, 2H). ^{13}C NMR ($CDCl_3$, 125 MHz): 173.3, 163.5, 149.7, 145.9, 143.1, 138.1, 137.2, 137.1, 134.5, 132.1, 129.6, 129.4, 128.0, 127.7, 123.3, 122.5, 110.0, 102.3, 66.1, 43.4. MS-APCI [M+H]: 384. HRMS (APCI) m/z: $[M+H]^+$ calculated for $C_{23}H_{18}N_3O_3$, 384.1343; found: 384.1340.

5-((*E*)-3-oxobut-1-en-1-yl)-1-(4-((*E*)-3-oxobut-1-en-1-yl)benzyl)spiro[indoline-3,2'-[1,3]dioxolan]-2-one (27): White solid. M.P.: 172-175°C. ^{1}H-NMR ($CDCl_3$, 500 MHz) δ: 7.59 (s, 1H), 7.26-7.49 (m, 7H), 6.56-6.66 (m, 3H), 4.81 (s, 2H), 4.60 (m, 2H), 4.36 (m, 2H) 2.32 (s, 3H), 2.30 (s, 3H). ^{13}C NMR ($CDCl_3$, 125 MHz): 198.2, 197.9, 173.3, 145.7, 142.5, 142.2, 137.3, 134.1, 132.8, 130.1, 128.8, 127.7, 127.4, 126.0, 125.0, 124.1, 109.9, 101.7, 66.0, 43.2, 27.6, 27.5. MS-APCI [M+H]: 418. HRMS (APCI) m/z: $[M+H]^+$ calculated for $C_{25}H_{24}NO_5$, 418.1649; found: 418.1649.

5-((*E*)-styryl)-1-(4-((*E*)-styryl)benzyl)spiro[indoline-3,2'-[1,3]dioxolan]-2-one (28): White solid. M.P.: 199-201°C. ^{1}H-NMR ($CDCl_3$, 500 MHz) δ: 7.66-7.80 (m, 2H), 7.46-56 (m, 6H), 7.26-7.35 (m, 9H), 7.01-7.2 (m, 2H), 6.66 (d, *J* = 8.1 Hz, 1H), 6.60 (d, *J* = 8.1 Hz, 1H), 4.84 (s, 2H), 4.41 (m, 2H), 4.35 (m, 2H). ^{13}C NMR ($CDCl_3$, 125 MHz): 173.5, 143.1, 138.1, 137.2, 137.1, 134.5, 133.3, 130.5, 129.2, 129.1, 128.8, 128.3, 128.1, 128.0, 127.8, 127.8, 127.7, 127.1, 126.6, 126.5, 124.6, 122.5, 110.0, 102.3, 66.1, 43.4. MS-APCI [M+H]: 486. HRMS (APCI) m/z: $[M+H]^+$ calculated for $C_{33}H_{28}NO_3$, 486.2064; found: 486.2059.

5-acetyl-1-(4-acetylbenzyl)spiro[indoline-3,2'-[1,3]dioxolan]-2-one (29): White solid. M.P.: 189-190°C. ^{1}H-NMR ($CDCl_3$, 500 MHz) δ: 7.89 (s, 1H), 7.80 (d, *J* = 8.2 Hz, 3H), 7.25 (d, *J* = 8.2 Hz, 2H), 6.57 (d, *J* = 8.2 Hz, 1H), 4.80 (s, 2H), 4.52 (m, 2H), 4.29 (m, 2H), 2.45 (s, 3H), 2.43 (s, 3H). ^{13}C NMR ($CDCl_3$, 125 MHz): 197.4, 196.1, 173.7,

147.6, 140.0, 136.8, 133.0, 133.0, 129.1, 127.3, 125.3, 124.4, 109.2, 101.6, 66.1, 43.3, 26.8, 26.4. MS-APCI [M+H]: 366. HRMS (APCI) m/z: $[M+H]^+$ calculated for $C_{21}H_{20}NO_5$, 366.1336; found: 366.1337.

5-(thiophen-2-yl)-1-(4-(thiophen-2-yl)benzyl)spiro[indoline-3,2'-[1,3]dioxolan]-2-one (30): Grey solid. M.P.: 152-153°C. ^{1}H-NMR ($CDCl_3$, 500 MHz) δ: 7.75-7.87 (m, 5H), 7.40-7.60 (m, 6H), 7.30-7.20 (m, 1H), 6.89 (d, *J* = 8.2 Hz, 1H), 5.05 (s, 2H), 4.88 (m, 2H), 4.62 (m, 2H). ^{13}C NMR ($CDCl_3$, 125 MHz): δ173.4, 143.7, 143.5, 142.9, 134.3, 134.1, 129.2, 128.1, 128.1, 127.8, 126.4, 126.2, 125.0, 124.8, 124.6, 123.3, 122.9, 122.8, 110.1, 102.2, 63,7, 43.3. MS-APCI [M+H]: 446. HRMS (APCI) m/z: $[M+H]^+$ calculated for $C_{25}H_{20}NO_3S_2$, 446.0879; found: 446.0882.

5-(furan-2-yl)-1-(4-(furan-2-yl)benzyl)spiro[indoline-3,2'-[1,3]dioxolan]-2-one (31): White solid. M.P.: 330-332°C. ^{1}H-NMR ($CDCl_3$, 500 MHz) δ: 7.29-7.70 (m, 8H), 6.43-6.68 (m, 5H), 4.83 (s, 2H), 4.66 (m, 2H), 4.39 (m, 2H). ^{13}C NMR ($CDCl_3$, 125 MHz): 173.4, 153.5, 153.2, 142.7, 142.2, 142.0, 141.9, 134.1, 130.5, 127.6, 127.0, 126.9, 124.6, 124.3, 120.8, 111.7, 110.0, 105.3, 104.4, 102.2, 66.0, 43.4. MS-APCI [M+H]: 414. HRMS (APCI) m/z: $[M+H]^+$ calculated for $C_{25}H_{20}NO_5$, 414.1336; found: 414.1337.

5-(phenylethynyl)-1-(4-(phenylethynyl)benzyl)spiro[indoline-3,2'-[1,3]dioxolan]-2-one (32): Grey solid. M.P.: 199-201°C. ^{1}H-NMR ($CDCl_3$, 500 MHz) δ: 7.25-7.60 (m, 16H), 6.61 (d, *J* = 8.2 Hz, 1H), 4.63 (s, 2H), 4.36 (m, 2H), 4.12 (m, 2H). ^{13}C NMR ($CDCl_3$, 125 MHz): 173.4, 171.2, 143.4, 135.1, 135.1, 132.2, 131.7, 131.5, 128.4, 128.3, 127.2, 124.5, 123.2, 123.1, 123.0, 118.6, 109.7, 101.9, 89.9, 89.4, 88.9, 88.7, 66.1, 60.4, 43.4. MS-APCI $[M+H]^+$: 482. HRMS (APCI) m/z: $[M+H]^+$ calculated for $C_{33}H_{24}NO_3$, 482.1751; found: 482.1752.

4'-((5-(4-formylphenyl)-2-oxospiro[indoline-3,2'-[1,3]dioxolan]-1-yl)methyl)-[1,1'-biphenyl]-4-carbaldehyde (33): White solid. M.P.: 192-194°C. ^{1}H-NMR ($CDCl_3$, 500 MHz) δ: 10.03 (d, *J* = 6.1 Hz, 2H), 7.94-7.90 (m, 4H), 7.50-7.69 (m, 8H), 7.42 (d, *J* = 7.9 Hz, 2H), 6.80 (d, *J* = 8.2 Hz, 1H), 4.92 (s, 2H), 4.68 (m, 2H), 4.41 (m, 2H). ^{13}C NMR ($CDCl_3$, 125 MHz): 191.9, 191.8, 173.5, 146.5, 146.2, 144.1, 139.4, 135.5, 135.4, 135.2, 130.8, 130.4, 130.4, 128.0, 127.7, 127.6, 127.5, 127.2, 125.0, 124.2, 110.2, 102.2, 66.1, 43.3. MS-APCI $[M+H]^+$: 490. HRMS (APCI) m/z: $[M+H]^+$ calculated for $C_{31}H_{24}NO_5$, 490.1649; found: 490.1646.

1-([1,1'-biphenyl]-4-ylmethyl)-5-phenylspiro[indoline-3,2'-[1,3]dioxolan]-2-one (34): White solid. M.P.: 177-178°C. ^{1}H-NMR ($CDCl_3$, 500 MHz) δ: 7.65 (s, 1H), 7.32-7.55 (m, 15H), 6.87 – 6.72 (d, *J* = 8.1 Hz, 1H), 4.92 (s, 2H), 4.69 (m, 2H), 4.40 (m, 2H). ^{13}C NMR ($CDCl_3$, 125 MHz): 173.6, 143.1, 140.8, 140.6, 140.4, 137.0, 135.5, 134.3, 130.4, 128.8, 127.7, 127.7, 127.5, 127.3, 127.1, 126.8, 124.7, 123.9, 110.1, 102.4, 66.1, 43.39. MS-APCI [M+H]: 434. HRMS (APCI) m/z: $[M+H]^+$ calculated for $C_{29}H_{24}NO_3$, 434.1751; found: 434.1750.

4'-((5-(4-cyanophenyl)-2-oxospiro[indoline-3,2'-[1,3]dioxolan]-1-yl)methyl)-[1,1'-biphenyl]-4-carbonitrile (35): White solid. M.P.: 248-250°C. ^{1}H-NMR ($CDCl_3$, 500 MHz) δ: 7.30-7.75 (m, 14H), 6.80 (d, *J* = 8.2 Hz, 1H), 4.92 (s, 2H), 4.67 (m, 2H), 4.41 (m, 2H). ^{13}C NMR ($CDCl_3$, 125 MHz): δ 173.5, 145.0, 144.0, 143.1, 138.9, 135.7, 134.9, 132.7, 132.7, 130.7, 128.2, 127.5, 127.3, 127,1, 126, 1, 125.1, 124.1, 118.9, 118.9, 111.2, 110.9, 110.2, 66.1, 43.3. MS-APCI $[M+H]^+$: 484. HRMS (APCI) m/z: $[M+H]^+$ calculated for $C_{31}H_{22}N_3O_3$, 484.1656; found: 484.1650.

Compound 11

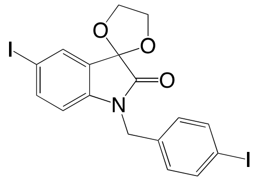

I
O
O
O
N
I

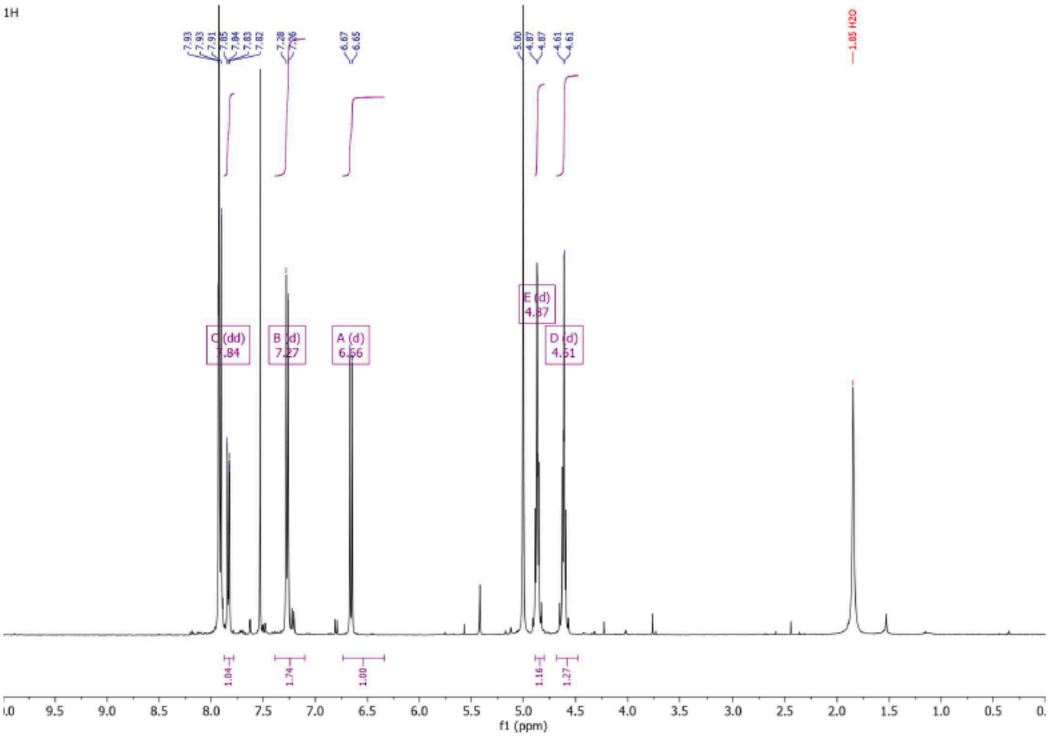

1H
1.85 H2O
C (dd)
7.84
B (d)
7.27
A (d)
6.56
E (d)
4.87
D (d)
4.61
1.04
1.74
1.00
1.16
1.27
f1 (ppm)

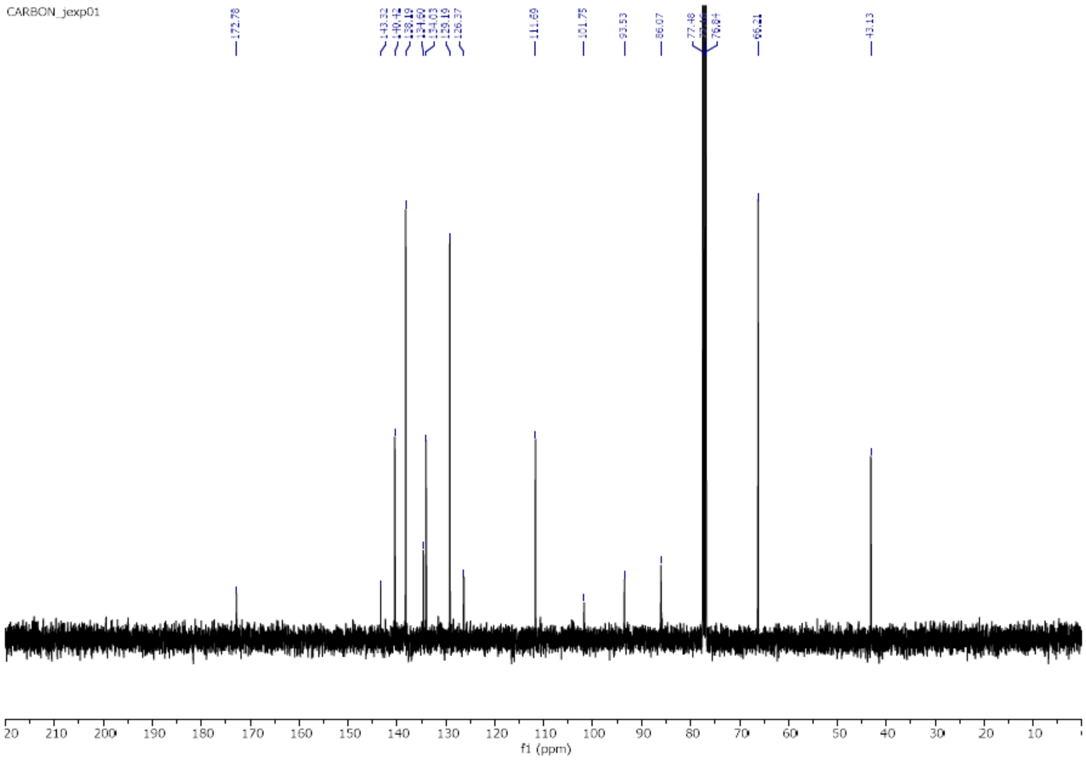

CARBON_jexp01
172.78
111.69
101.75
93.53
86.07
77.48
76.84
66.21
43.13
f1 (ppm)

ESPECTRO APCI-FIA-Ion Trap

Analysis Info

Sample Name	3 APCI MS24-0266-1 533_2_01_2057.d	Acquisition Date	12/04/2024 11:44:58
Method	2057.m	Instrument	amaZon ETD

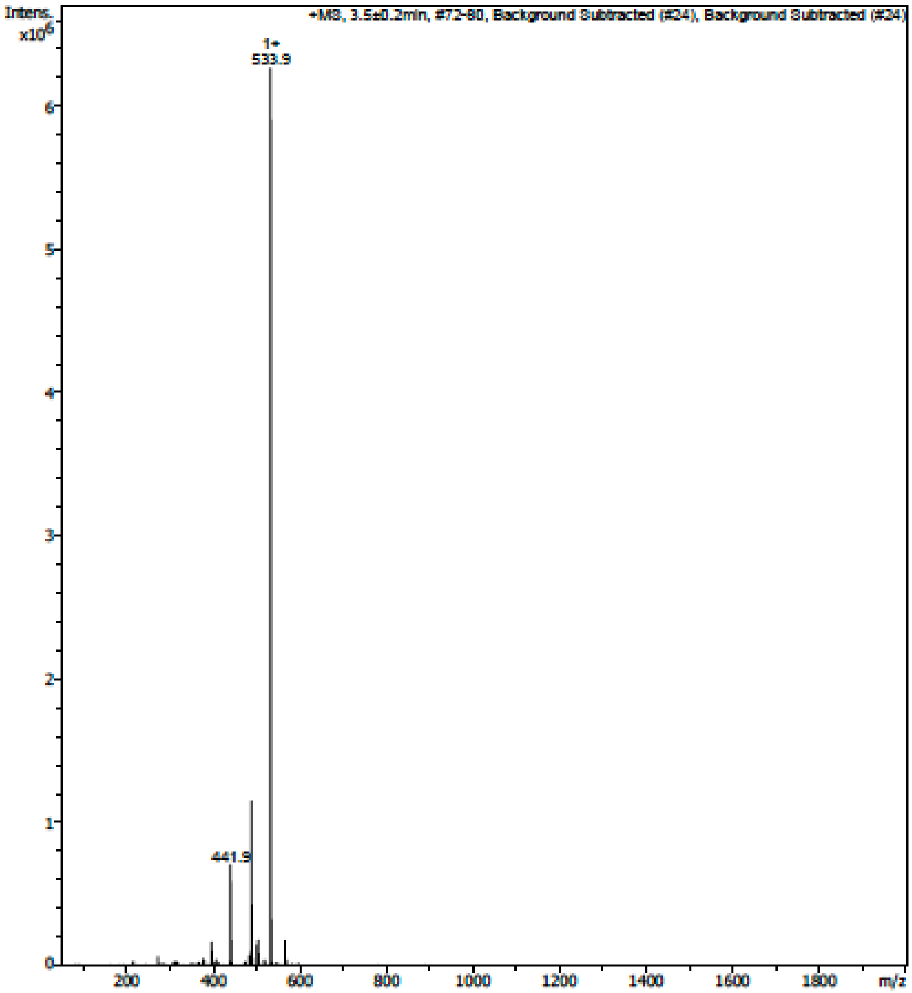

Compound 12:

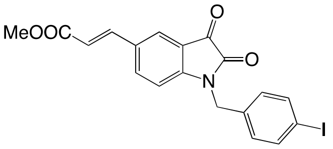
MeOOC
O
O
N
I

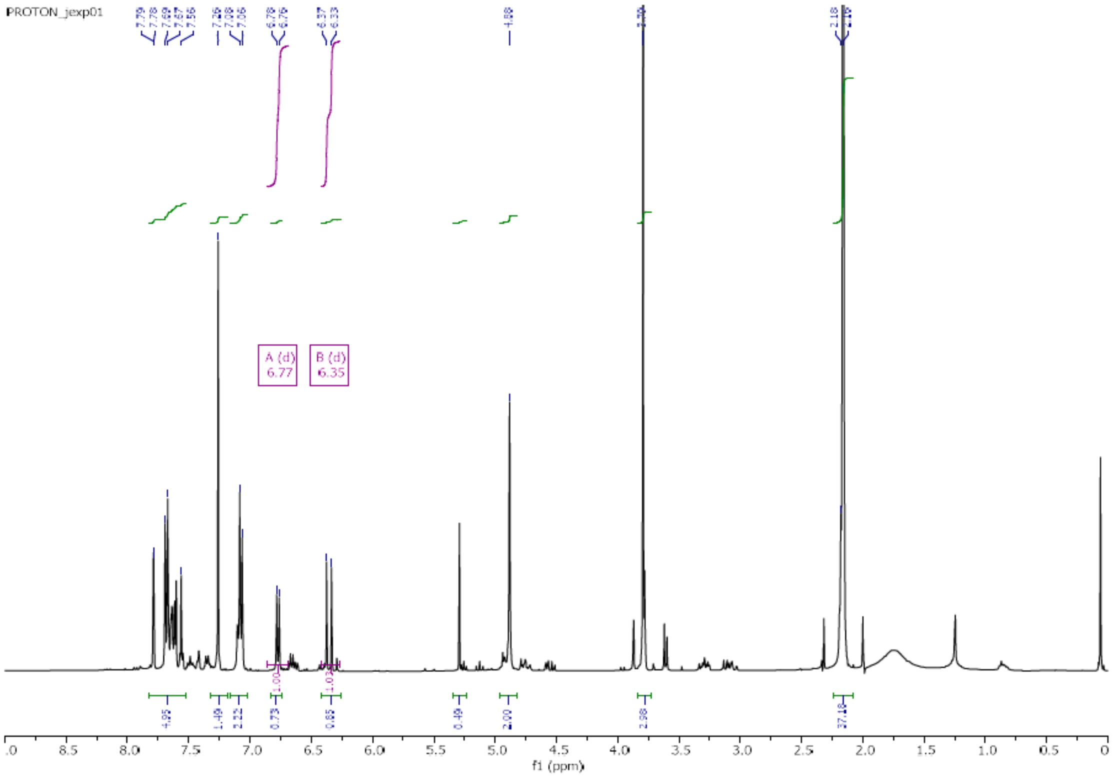
PROTON_jexp01
A (d)
6.77
B (d)
6.35
f1 (ppm)

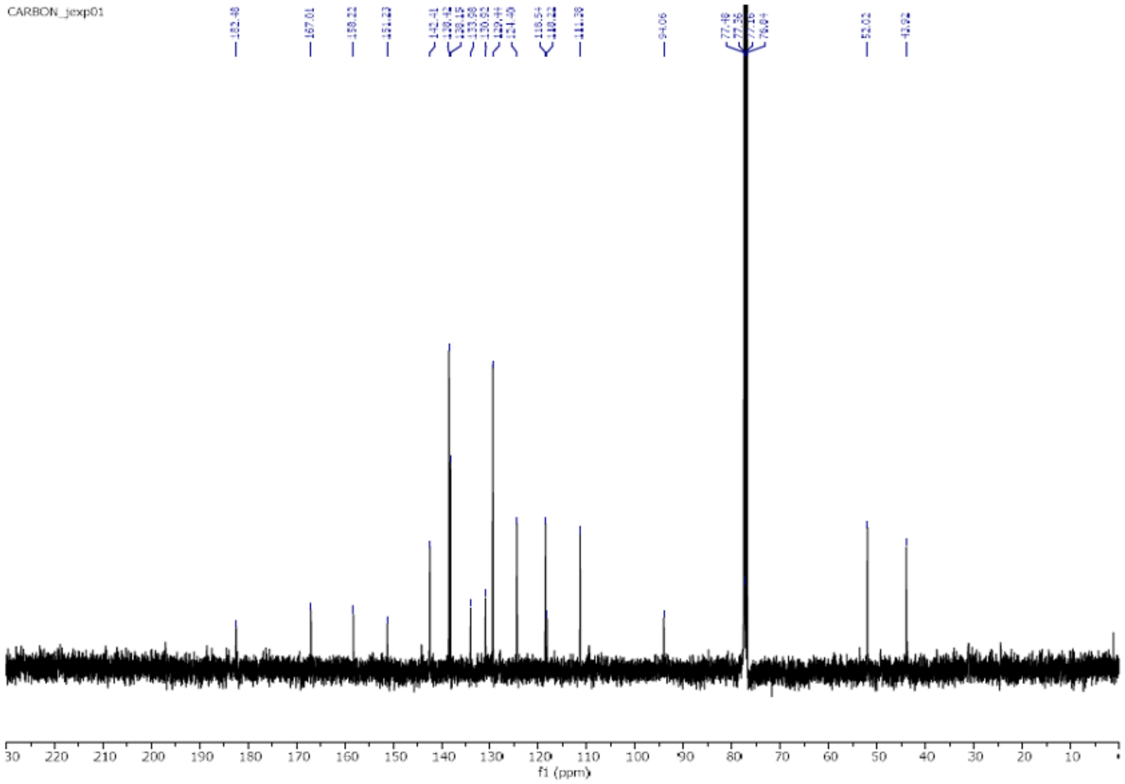
CARBON_jexp01
f1 (ppm)

UNIDADE DE ESPECTROMETRÍA DE MASAS E PROTEÓMICA

AREA DE INFRAESTRUCTURAS DE INVESTIGACION
Edificio CACTUS * Campus Vida
15782 Santiago de Compostela
Tel. 881816242. Ext. 16242
E-mail: sxamasas@usc.es
Web:
https://www.usc.gal/es/servicios/unidades/unidad-espectrometria-masas-proteom

ESPECTRO APCI-FIA-Ion Trap

Analysis Info

Sample Name	15 APCI MS24-0283-1 446_12_01_2091.d	Acquisition Date	16/04/2024 19:58:41
Method	2091.m	Instrument	amaZon ETD

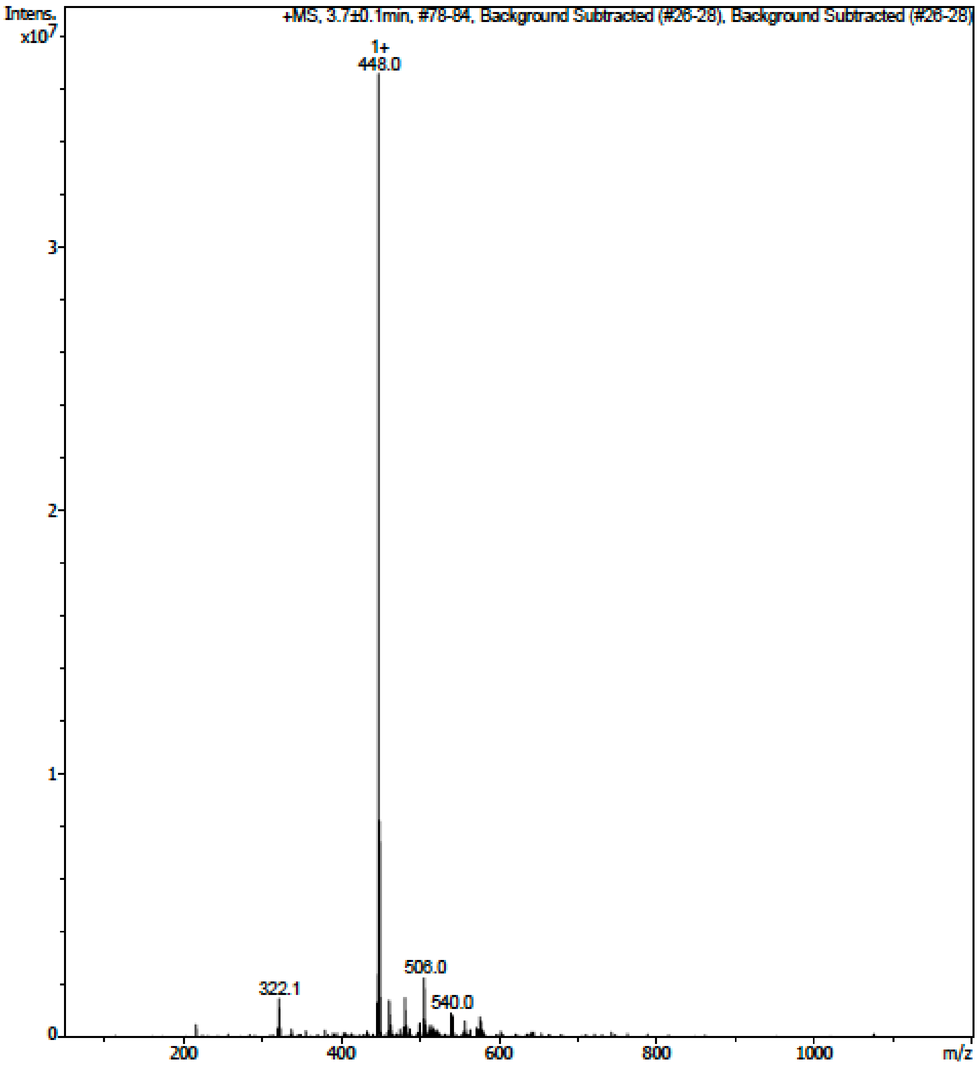

Medida De Masas Exactas

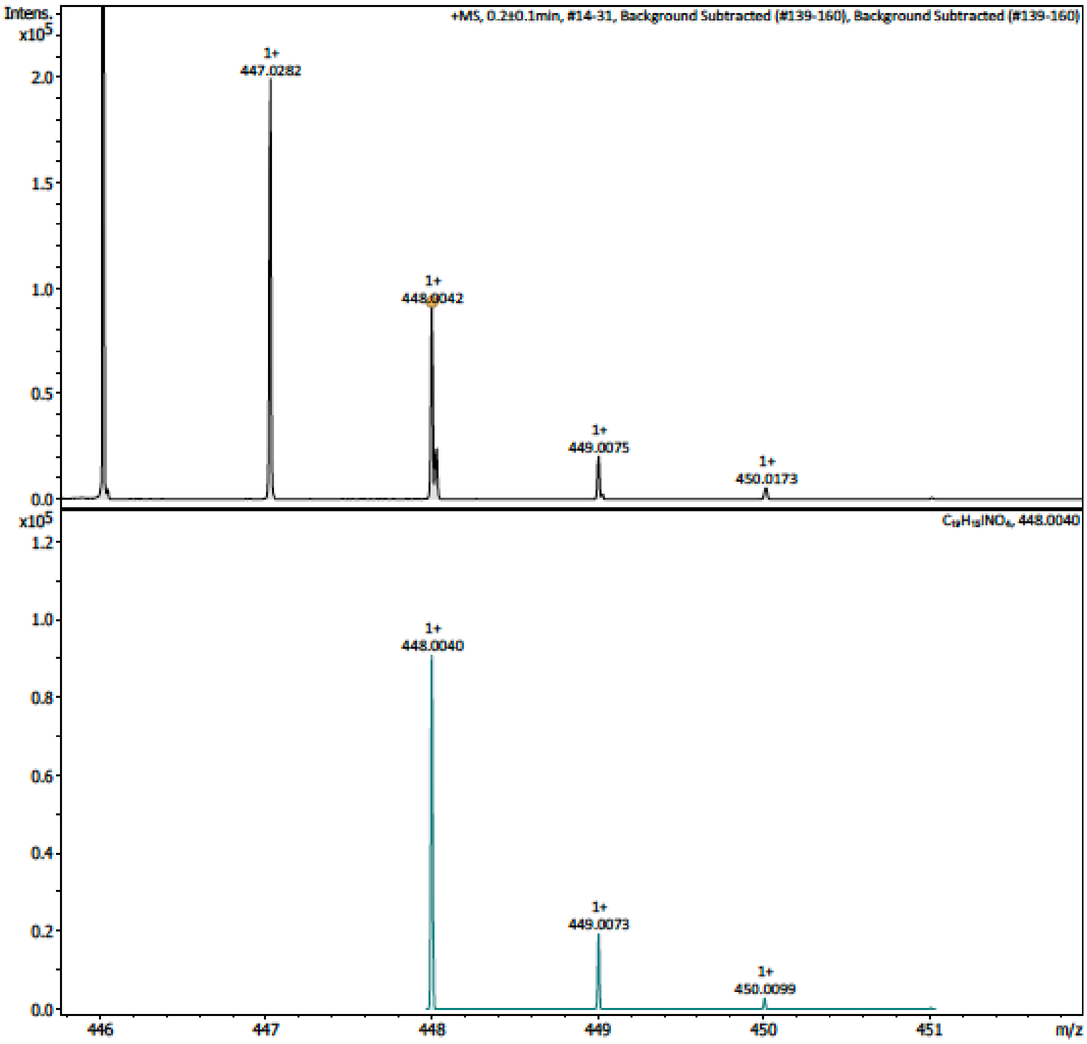

Meas. m/z	#	Ion Formula	Score	m/z	err [mDa]	err [ppm]	mSigma	rdb (neutral)	e⁻ Conf	N-Rule
448.0042	1	C20H6N3O10	100.00	448.0048	0.6	1.3	7.5	20.0	even	ok
448.0042	2	C18H4N6O9	66.11	448.0034	-0.8	-1.7	8.7	20.5	odd	ok
448.0042	3	C19H15INO4	75.60	448.0040	-0.2	-0.4	16.6	16.0	even	ok
448.0042	4	C20H11IN5	43.41	448.0054	1.2	2.6	18.8	21.0	even	ok
448.0042	5	C17H13IN4O3	32.58	448.0027	-1.5	-3.4	21.9	16.5	odd	ok

Compound 13:

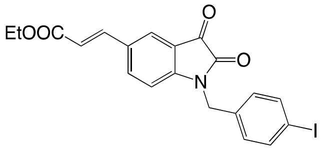
EtOOC
O
O
N
I

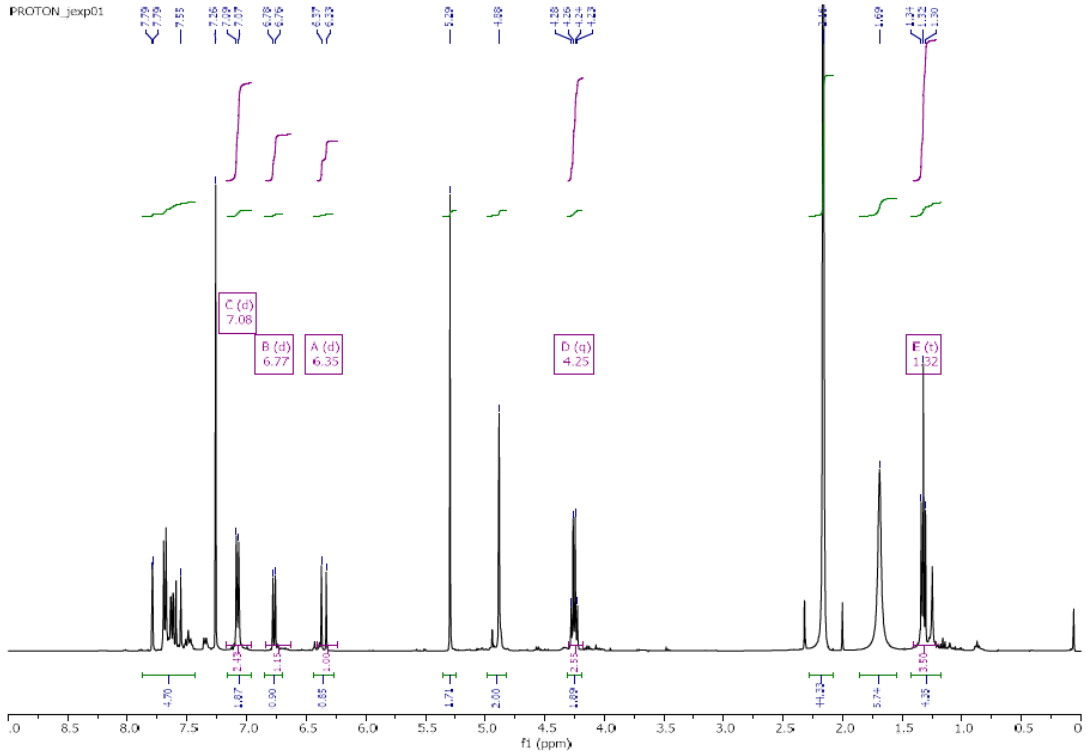
PROTON_jexp01
C (d)
7.08
B (d)
6.77
A (d)
6.35
D (q)
4.25
E (t)
1.32
f1 (ppm)

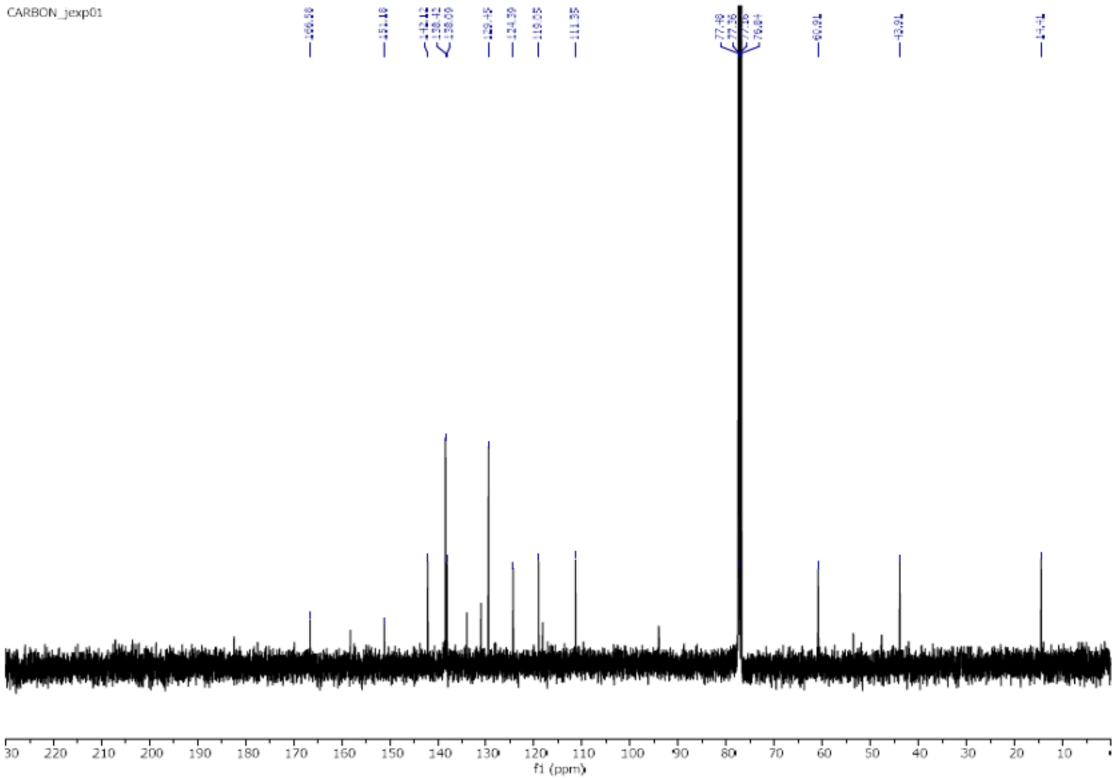
CARBON_jexp01
f1 (ppm)

AREA DE INFRAESTRUCTURAS DE INVESTIGACION
Edificio CACTUS * Campus Vida
15782 Santiago de Compostela
Tel. 881816242. Ext. 16242
E-mail: sxemasas@usc.es
Web:
https://www.usc.gal/es/servicios/unidades/unidad-espectrometria-masas-proteom

ESPECTRO APCI-FIA-Ion Trap

Analysis Info

Sample Name	17 APCI MS24-0283-3 461_14_01_2093.d	Acquisition Date	16/04/2024 20:20:58
Method	2093.m	Instrument	amaZon ETD

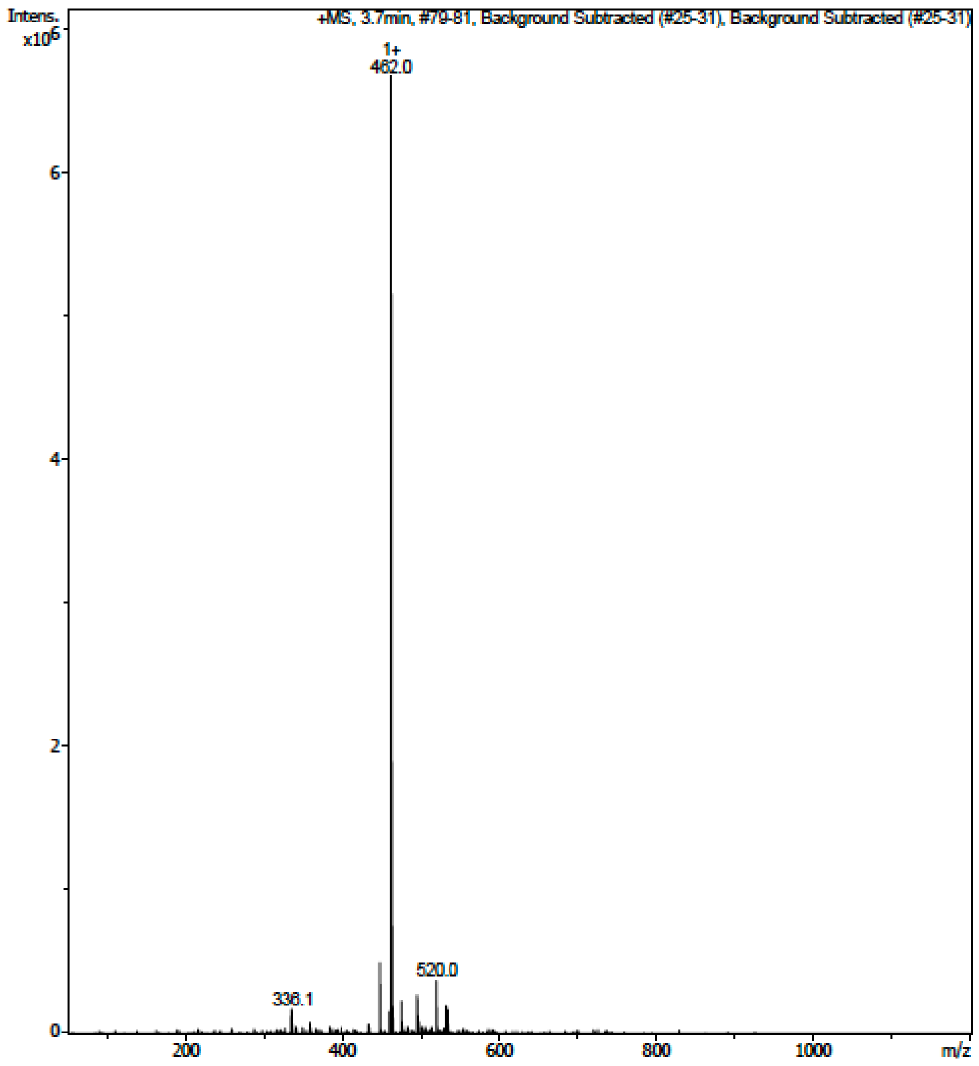

Medida De Masas Exactas

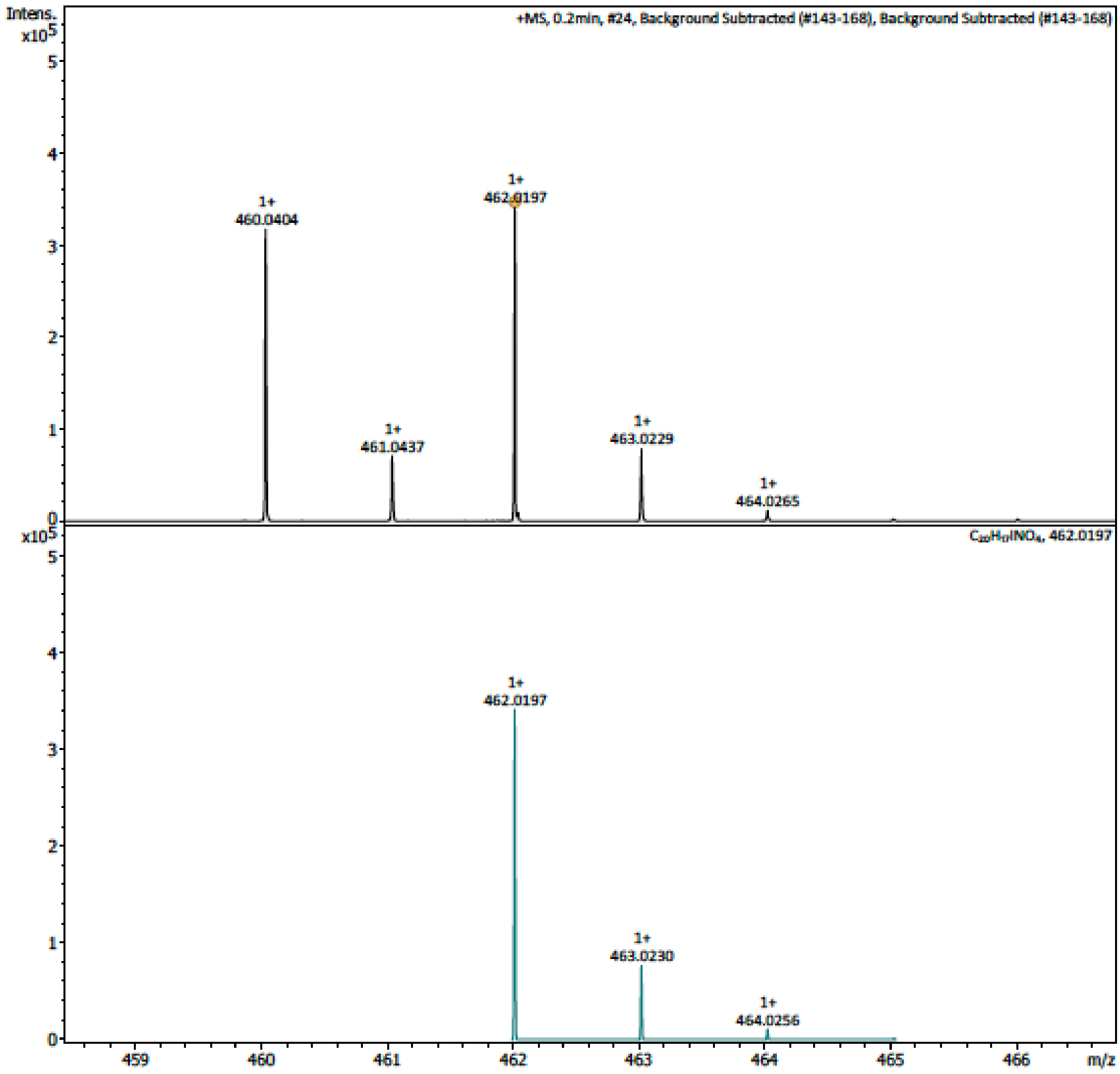

Meas. m/z	#	Ion Formula	Score	m/z	err [mDa]	err [ppm]	mSigma	rdb (neutral)	e⁻ Conf	N-Rule
462.0197	1	C20H17INO4	100.00	462.0197	0.0	0.1	3.1	16.0	even	ok
462.0197	2	C19H6N6O9	98.77	462.0191	-0.6	-1.2	6.3	20.5	odd	ok
462.0197	3	C18H15IN4O3	45.87	462.0183	-1.3	-2.8	9.9	16.5	odd	ok
462.0197	4	C21H13IN5	43.88	462.0210	1.4	3.0	10.5	21.0	even	ok
462.0197	5	C21H8N3O10	82.20	462.0204	0.8	1.7	11.0	20.0	even	ok

Compound 14:

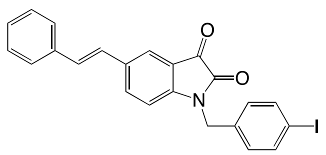

O
O
N
I

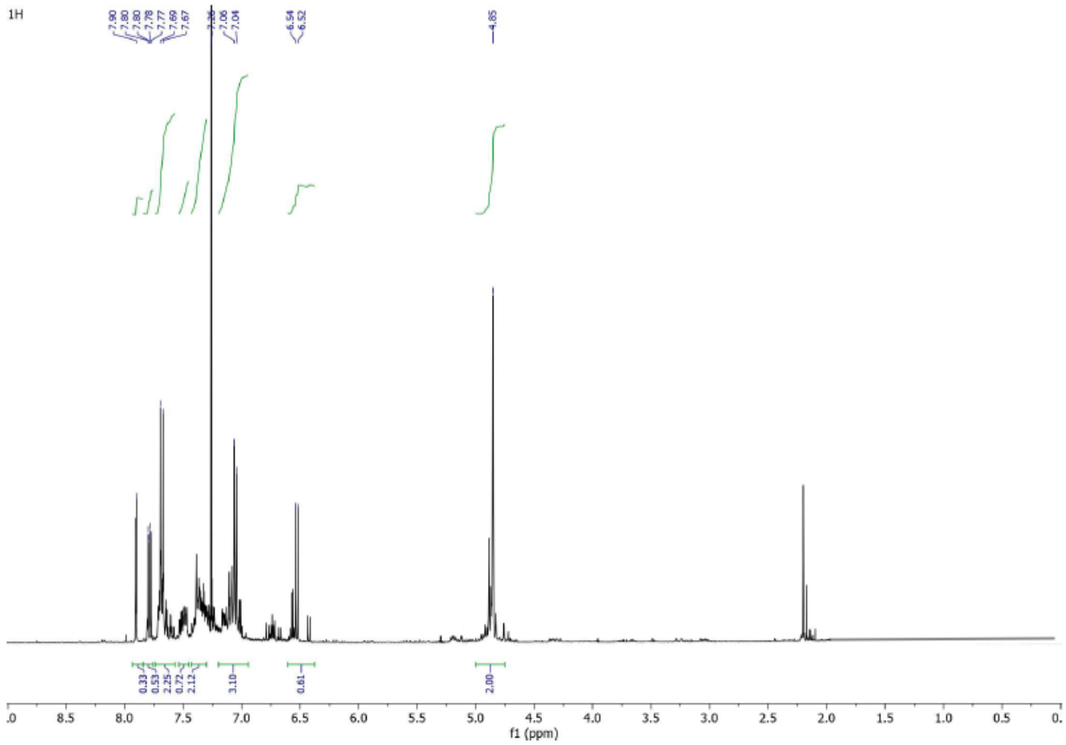

1H
7.90
7.80
7.80
7.78
7.77
7.69
7.67
7.06
7.04
6.54
6.52
4.85
0.33
0.53
2.25
0.72
2.12
3.10
0.61
2.00
f1 (ppm)

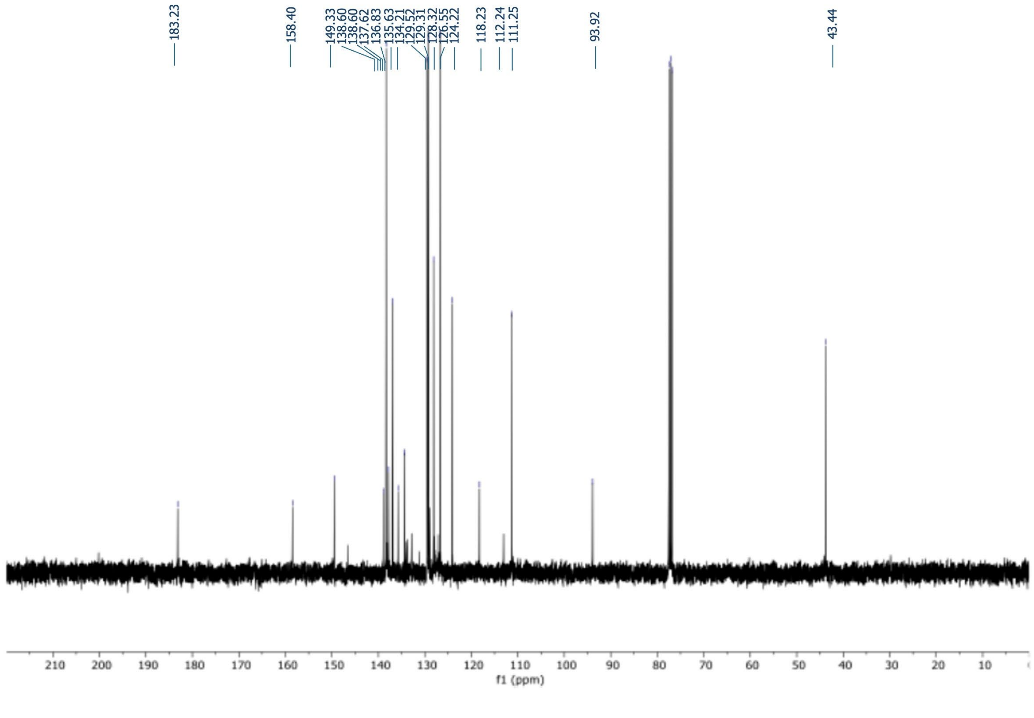

183.23
158.40
149.33
138.60
138.60
137.62
136.83
135.63
134.21
129.52
129.31
128.32
126.55
124.22
118.23
112.24
111.25
93.92
43.44
f1 (ppm)

UNIDADE DE ESPECTROMETRÍA DE MASAS E PROTEÓMICA

AREA DE INFRAESTRUCTURAS DE INVESTIGACION
Edificio CACTUS * Campus Vida
15782 Santiago de Compostela
Tel. 881816242. Ext. 16242
E-mail: sxemasas@usc.es
Web:
https://www.usc.gal/es/servicios/unidades/unidad-espectrometria-masas-proteom

ESPECTRO APCI-FIA-Ion Trap

Analysis Info

Sample Name	11 APCI MS-24-0378-9-tb3 485_10_01_2214.d	Acquisition Date	16/05/2024 12:21:22
Method	2214.m	Instrument	amaZon ETD

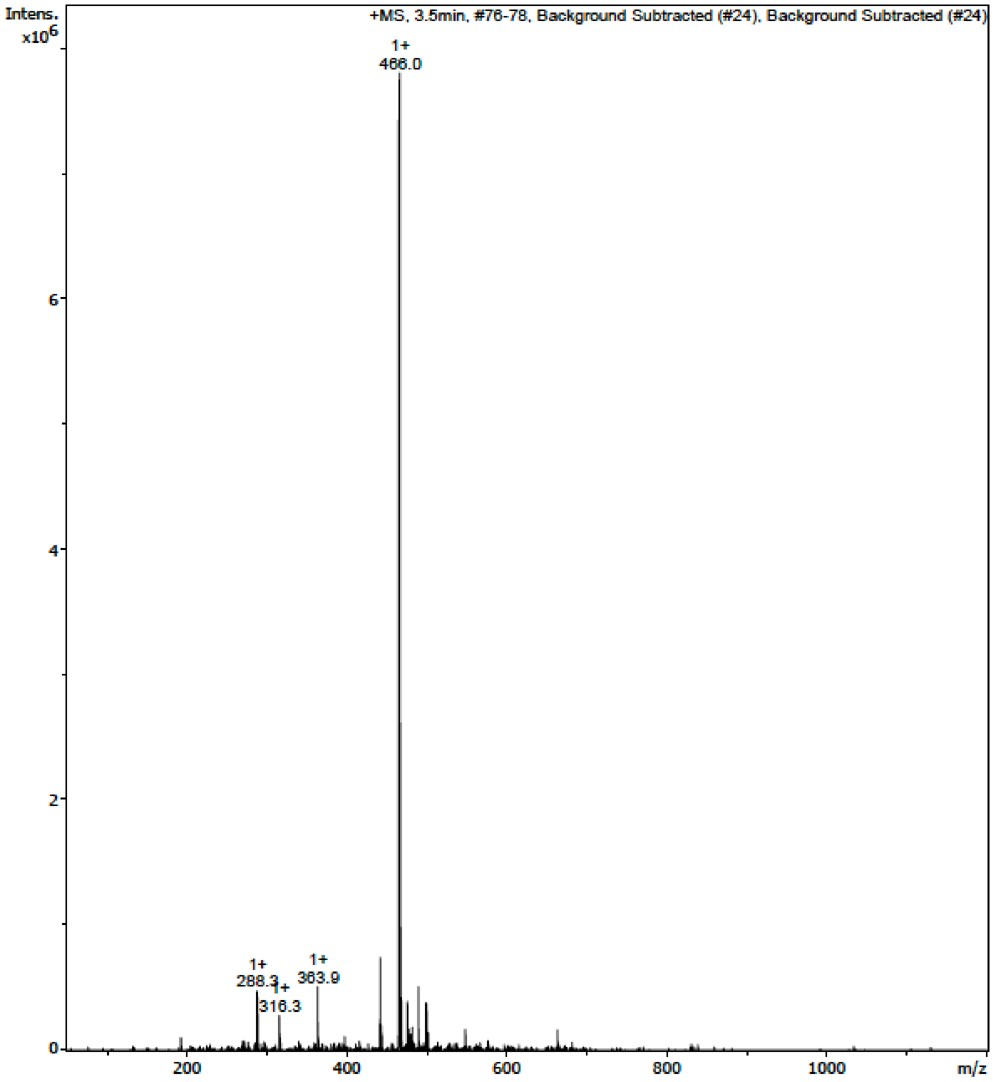

Medida De Masas Exactas

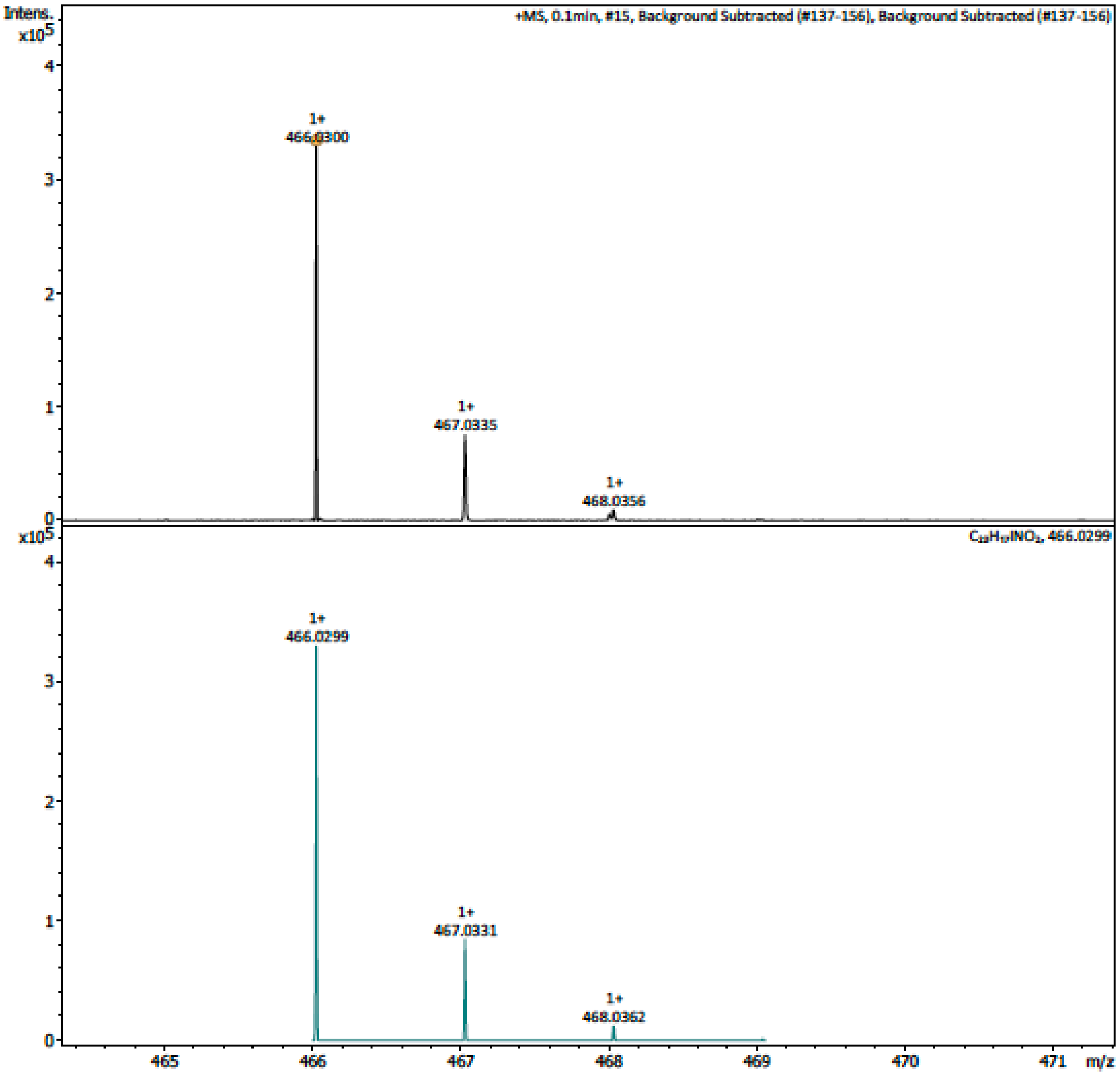

Meas. m/z	#	Ion Formula	Score	m/z	err [mDa]	err [ppm]	mSigma	rdb (neutral)	e⁻ Conf	N-Rule
466.0300	1	C21H15IN4O	55.51	466.0285	-1.5	-3.2	8.6	19.5	odd	ok
466.0300	2	C23H17INO2	100.00	466.0299	-0.1	-0.3	15.6	19.0	even	ok
466.0300	3	C22H6N6O7	91.80	466.0292	-0.7	-1.6	19.8	23.5	odd	ok
466.0300	4	C24H8N3O8	86.30	466.0306	0.6	1.3	26.0	23.0	even	ok
466.0300	5	C23H2N10O3	52.36	466.0306	0.6	1.3	34.8	28.5	odd	ok

Compound 8:

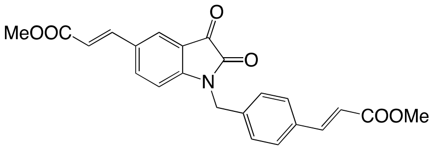

tb31012024_7_ACC-NV04B_20240113_01

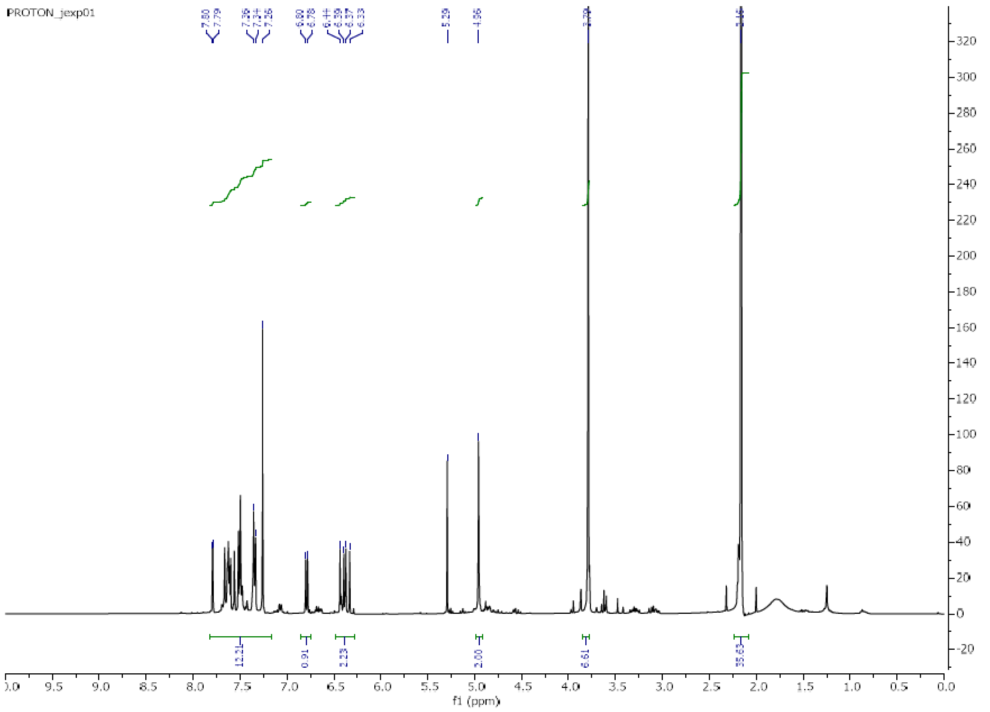

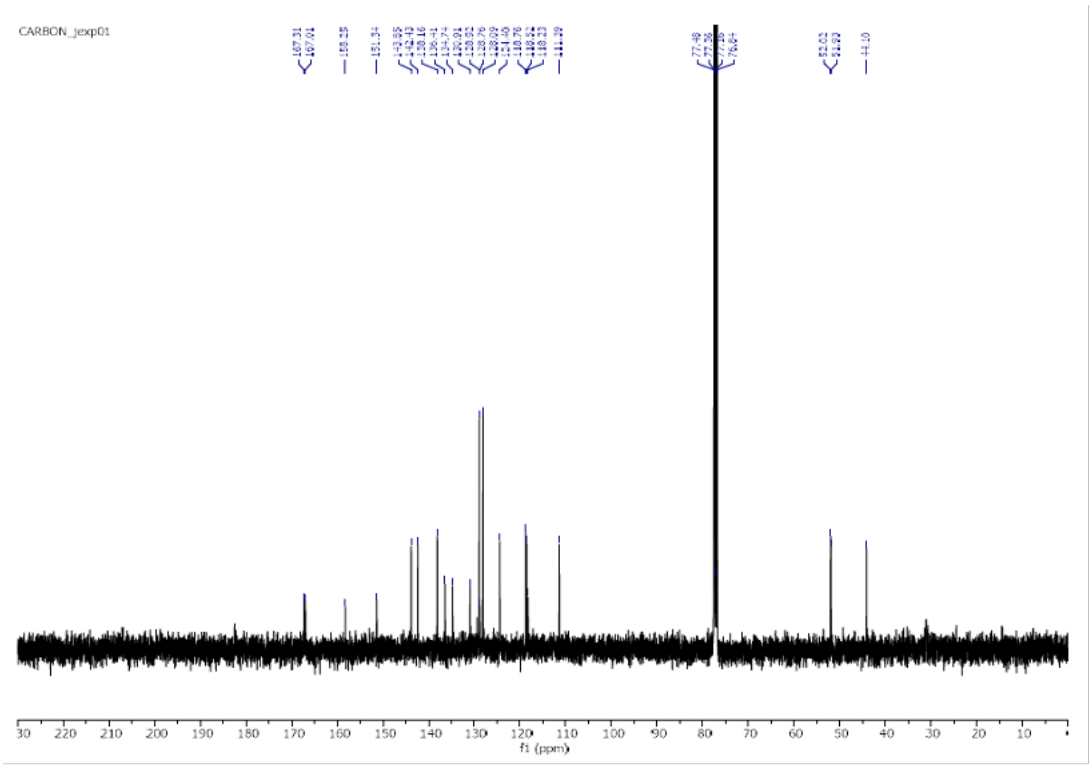

UNIDADE DE ESPECTROMETRÍA DE MASAS E PROTEÓMICA

AREA DE INFRAESTRUCTURAS DE INVESTIGACION
Edificio CACTUS * Campus Vida
15782 Santiago de Compostela
Tel. 881816242. Ext. 16242
E-mail: sxemasas@usc.es
Web:
https://www.usc.gal/es/servicios/unidades/unidad-espectrometria-masas-proteom

ESPECTRO APCI-FIA-Ion Trap

Analysis Info

Sample Name	16 APCI MS24-0283-2 405_13_01_2092.d	Acquisition Date	16/04/2024 20:09:51
Method	2092.m	Instrument	amaZon ETD

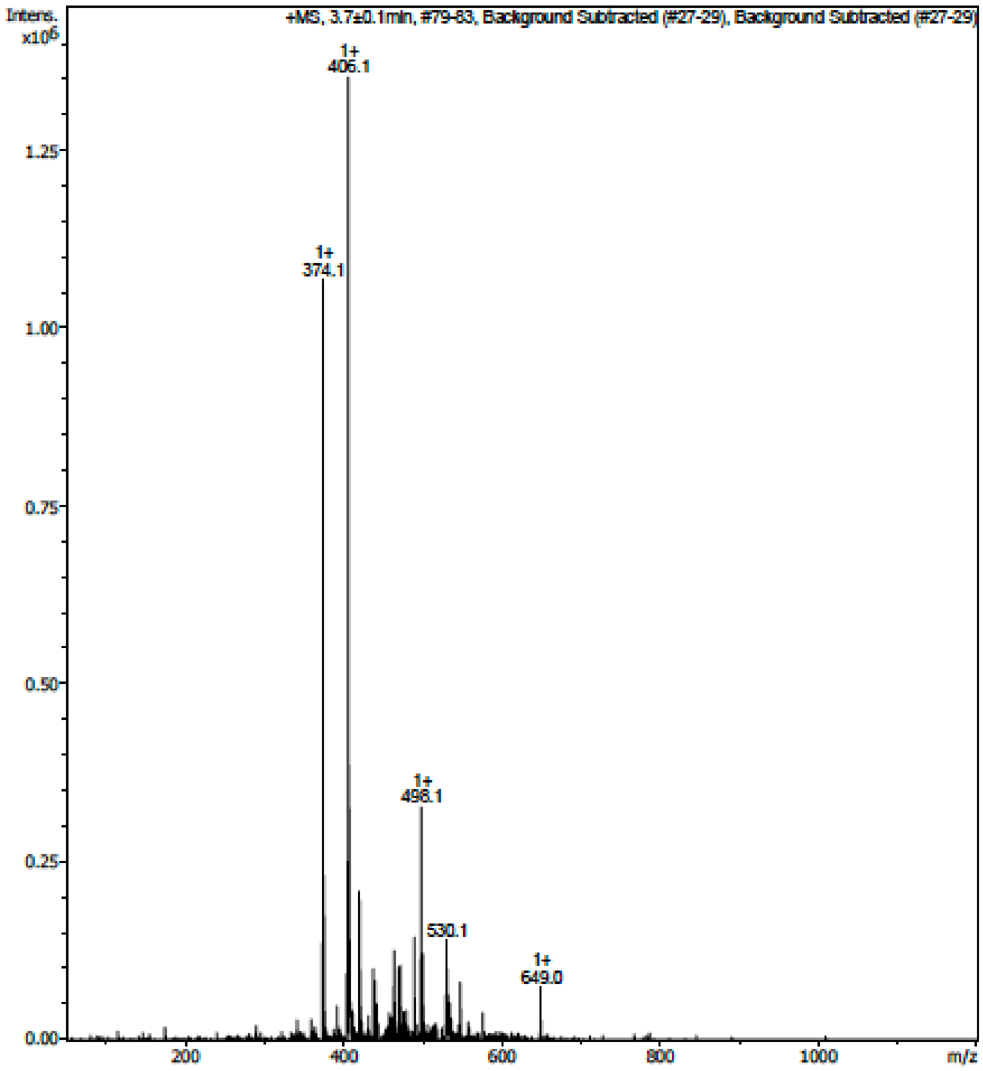

Medida De Masas Exactas

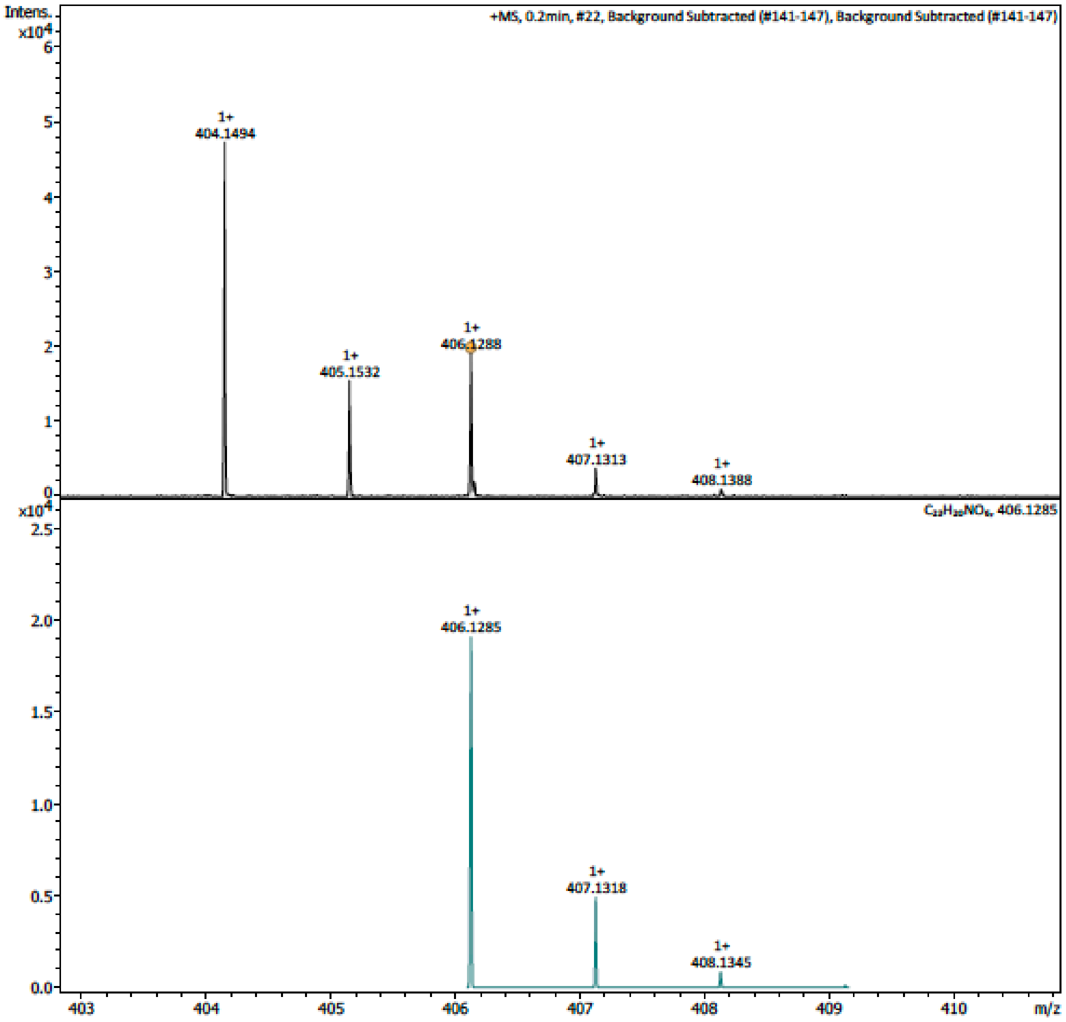

Meas. m/z	#	Ion Formula	Score	m/z	err [mDa]	err [ppm]	mSigma	rdb (neutral)	e⁻ Conf	N-Rule
406.1288	1	C23H20NO6	100.00	406.1285	-0.3	-0.6	35.1	15.0	even	ok

Compound 15:

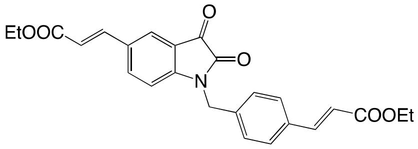
EtOOC
O
O
N
COOEt

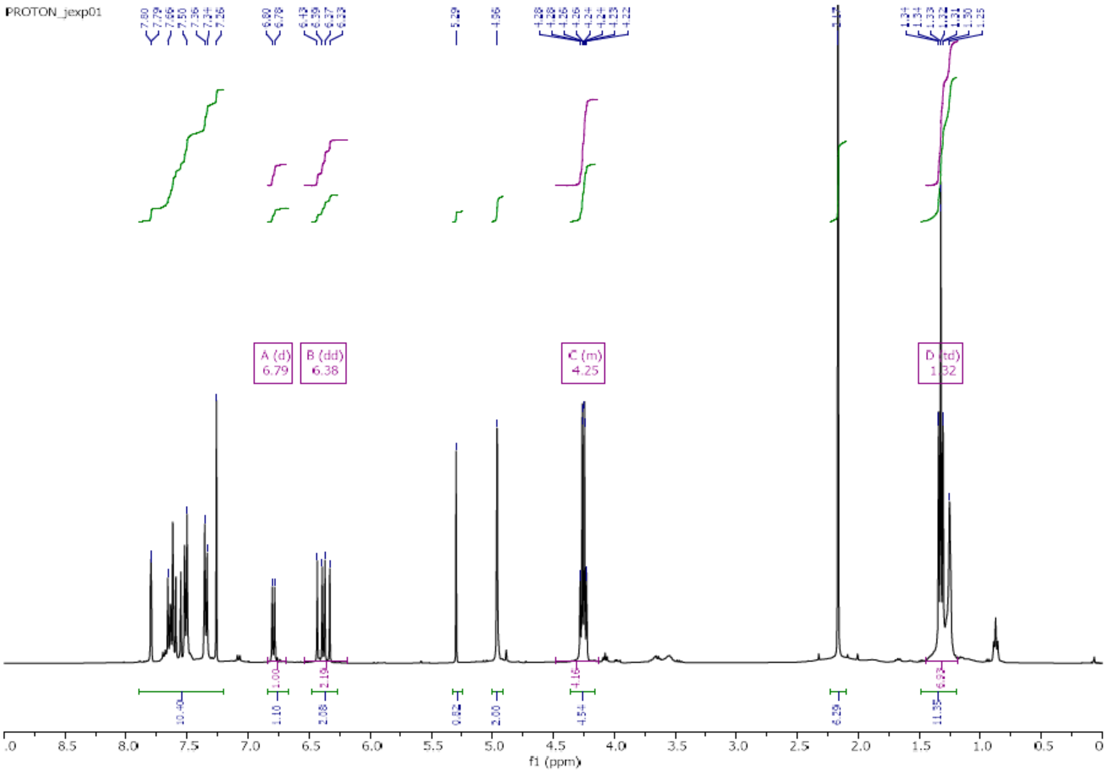
PROTON_jexp01
A (d)
6.79
B (dd)
6.38
C (m)
4.25
f1 (ppm)

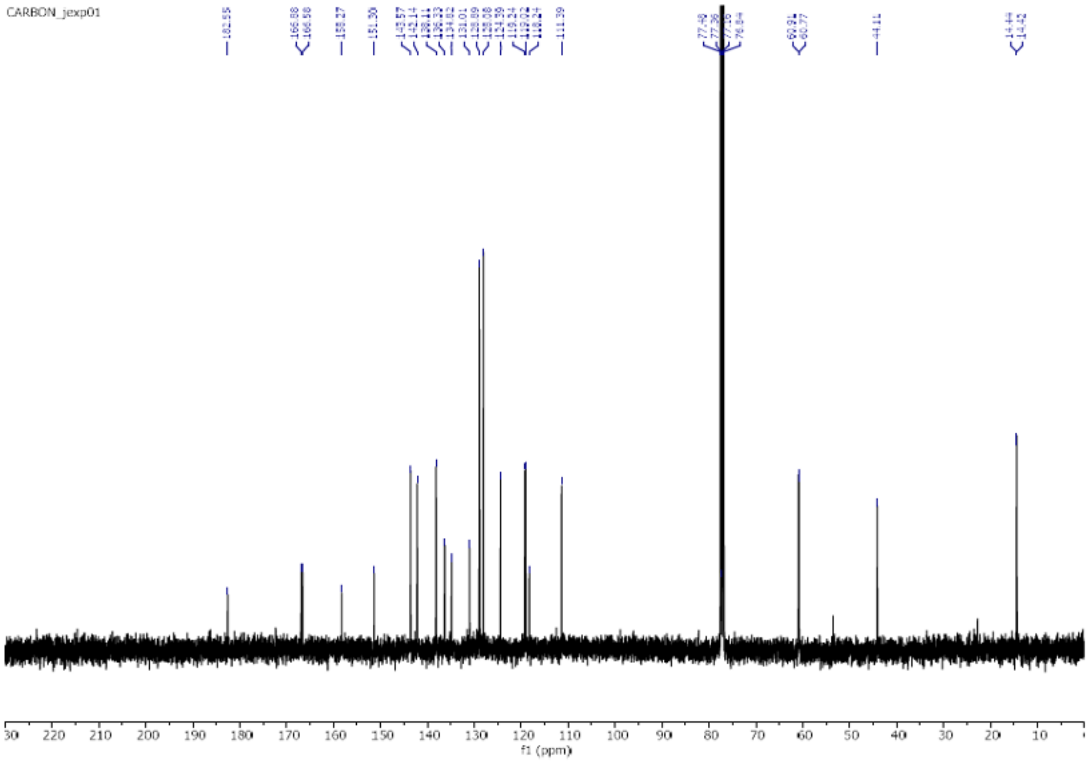
CARBON_jexp01
f1 (ppm)

AREA DE INFRAESTRUCTURAS DE INVESTIGACION
Edificio CACTUS * Campus Vida
15782 Santiago de Compostela
Tel. 881816242. Ext. 16242
E-mail: sxemasas@usc.es
Web:
https://www.usc.gal/es/servicios/unidades/unidad-espectrometria-masas-proteom

UNIDADE DE ESPECTROMETRÍA DE MASAS E PROTEÓMICA

ESPECTRO APCI-FIA-Ion Trap

Analysis Info

Sample Name	19 APCI MS24-0283-4 433_15_01_2095.d	Acquisition Date	16/04/2024 20:42:42
Method	2095.m	Instrument	amaZon ETD

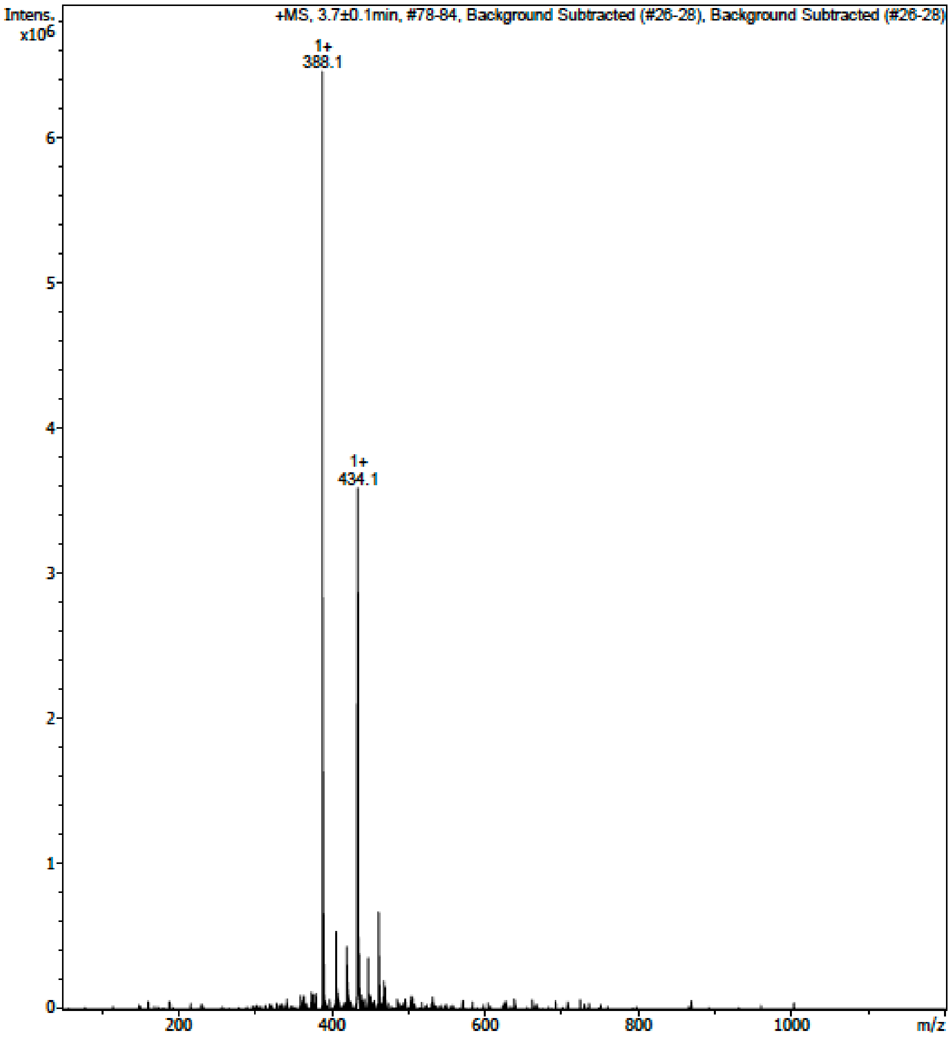

Medida De Masas Exactas

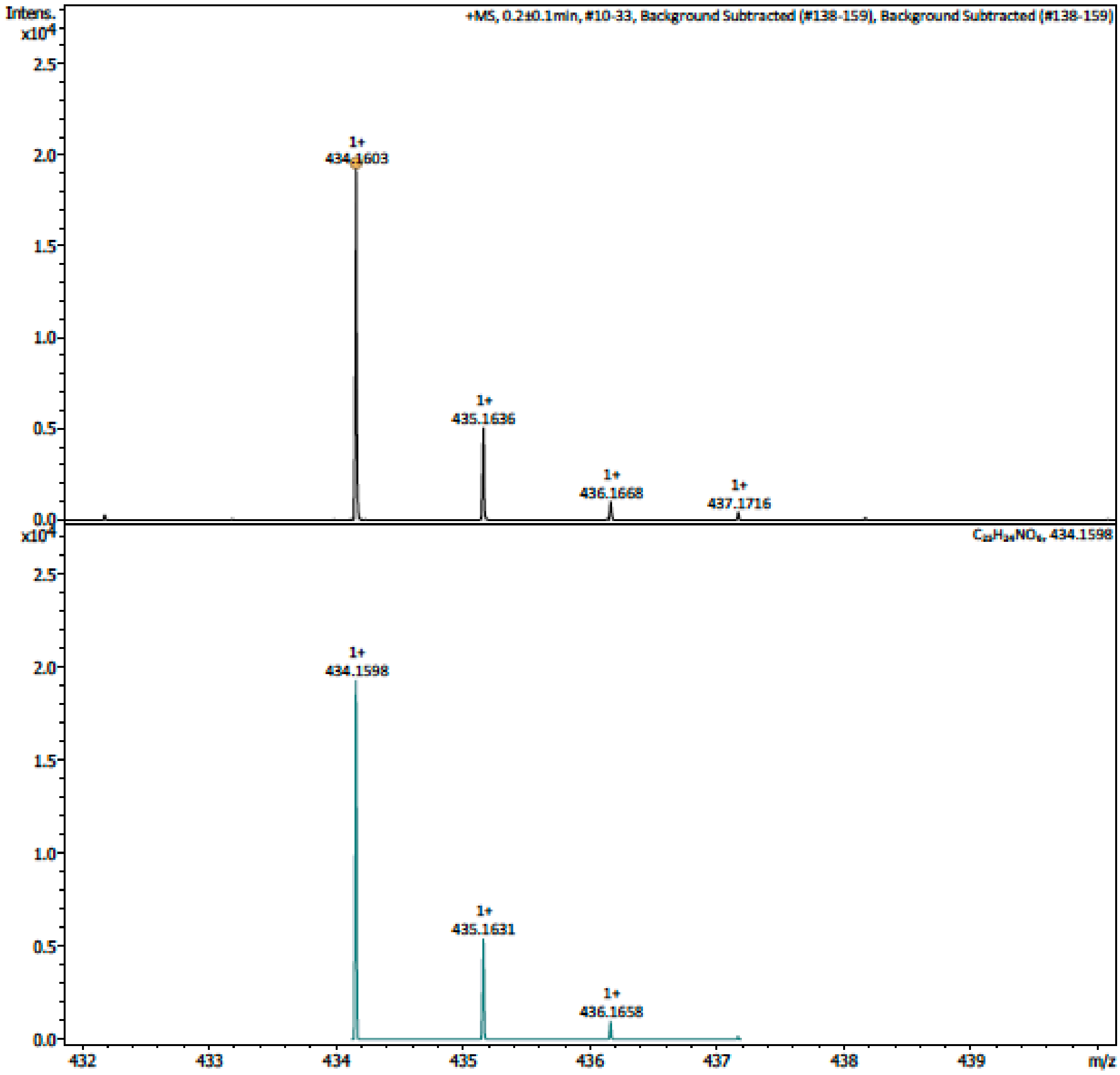

Meas. m/z	#	Ion Formula	Score	m/z	err [mDa]	err [ppm]	mSigma	rdb (neutral)	e⁻ Conf	N-Rule
434.1603	1	C25H24NO6	100.00	434.1598	-0.5	-1.2	11.5	15.0	even	ok
434.1603	2	C24H18N8O	66.31	434.1598	-0.5	-1.2	17.6	20.5	odd	ok
434.1603	3	C26H20N5O2	51.14	434.1612	0.8	1.9	23.4	20.0	even	ok
434.1603	4	C11H16N17O3	19.90	434.1617	1.3	3.0	48.3	13.0	even	ok
434.1603	5	C12H22N10O8	16.83	434.1617	1.3	3.0	53.7	7.5	odd	ok

Compound 16:

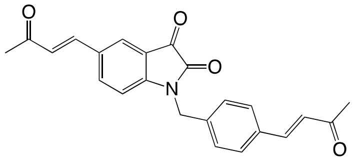

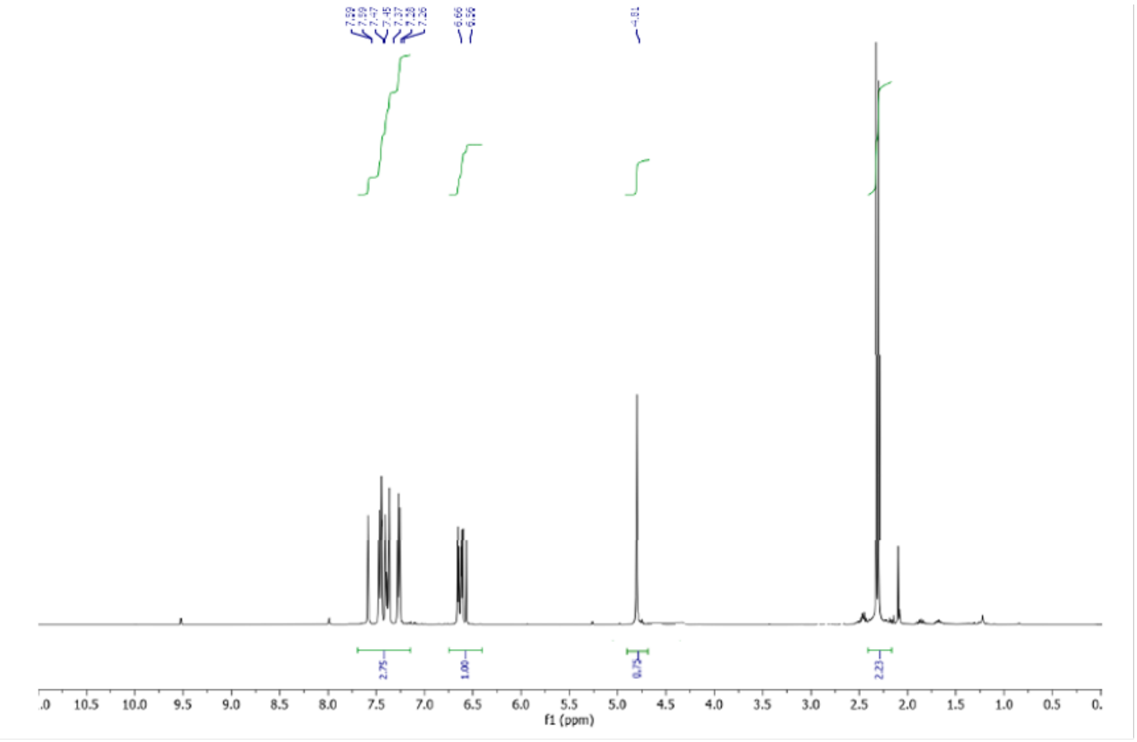
7.45
7.26
6.66
4.81
2.75
1.00
0.75
2.23
f1 (ppm)

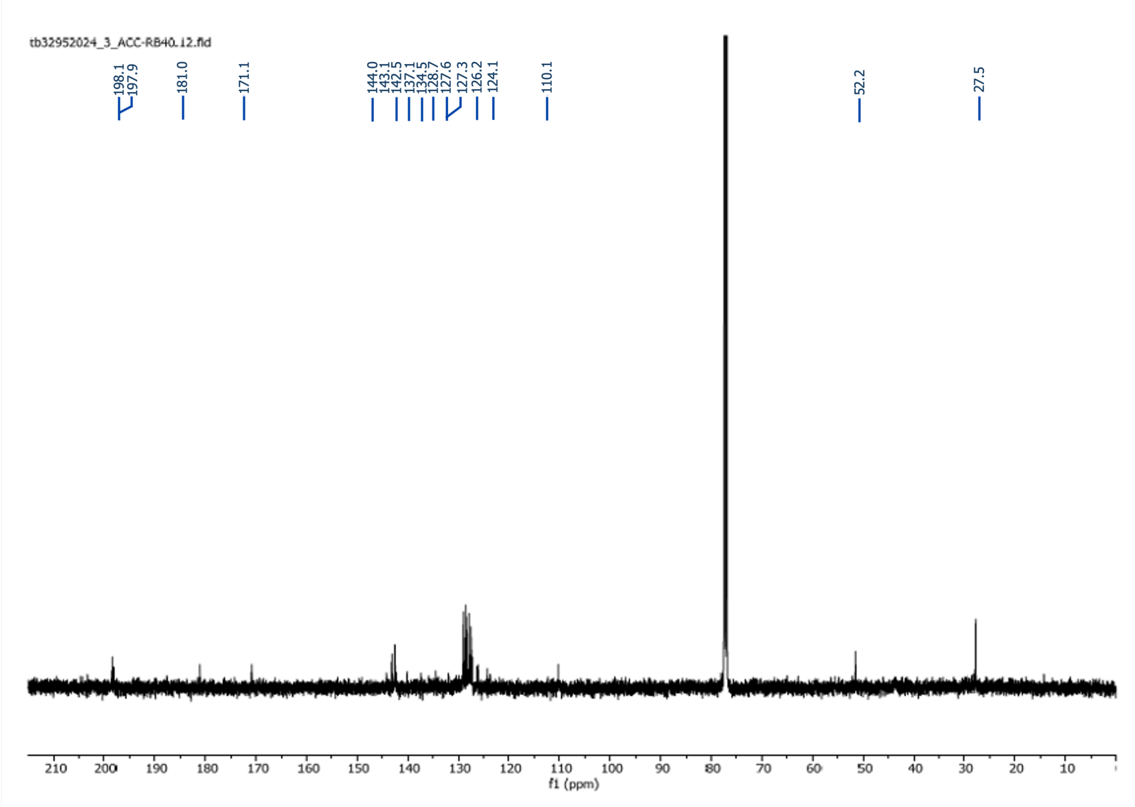
tb32952024_3_ACC-RB40.12.fid
198.1
197.9
181.0
171.1
144.0
143.1
142.5
137.1
134.5
128.7
127.6
127.3
126.2
124.1
110.1
52.2
27.5
f1 (ppm)

UNIDADE DE ESPECTROMETRÍA
DE MASAS E PROTEÓMICA

AREA DE INFRAESTRUCTURAS DE INVESTIGACION
Edificio CACTUS * Campus Vida
15782 Santiago de Compostela
Tel. 881816242. Ext. 16242
E-mail: sxemasas@usc.es
Web:
https://www.usc.gal/es/servicios/unidades/unidad-espectrometria-masas-proteom

ESPECTRO APCI-FIA-Ion Trap

Analysis Info

Sample Name	5 APCI MS-24-0756-3-tb3 373_4_01_2541.d	Acquisition Date	30/09/2024 15:47:21
Method	2541.m	Instrument	amaZon ETD

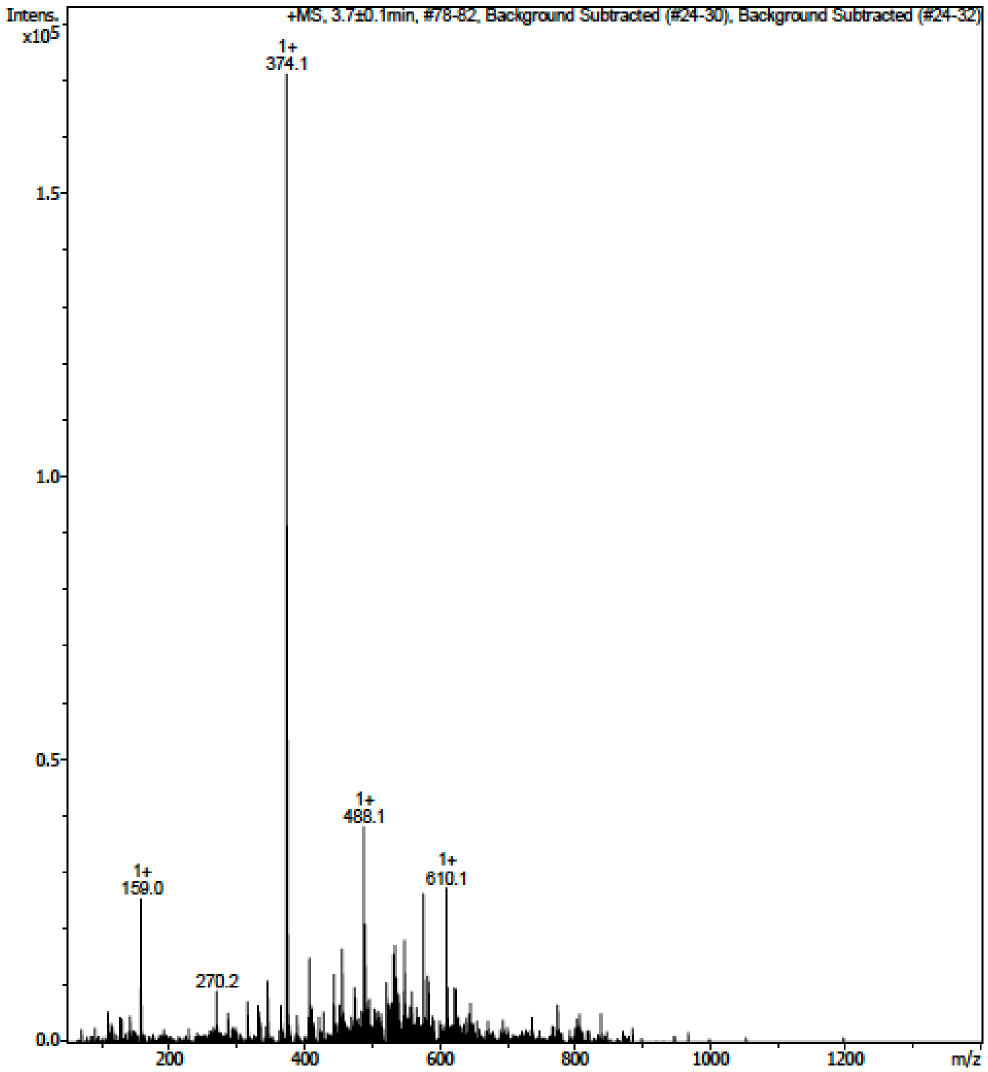

Medida De Masas Exactas

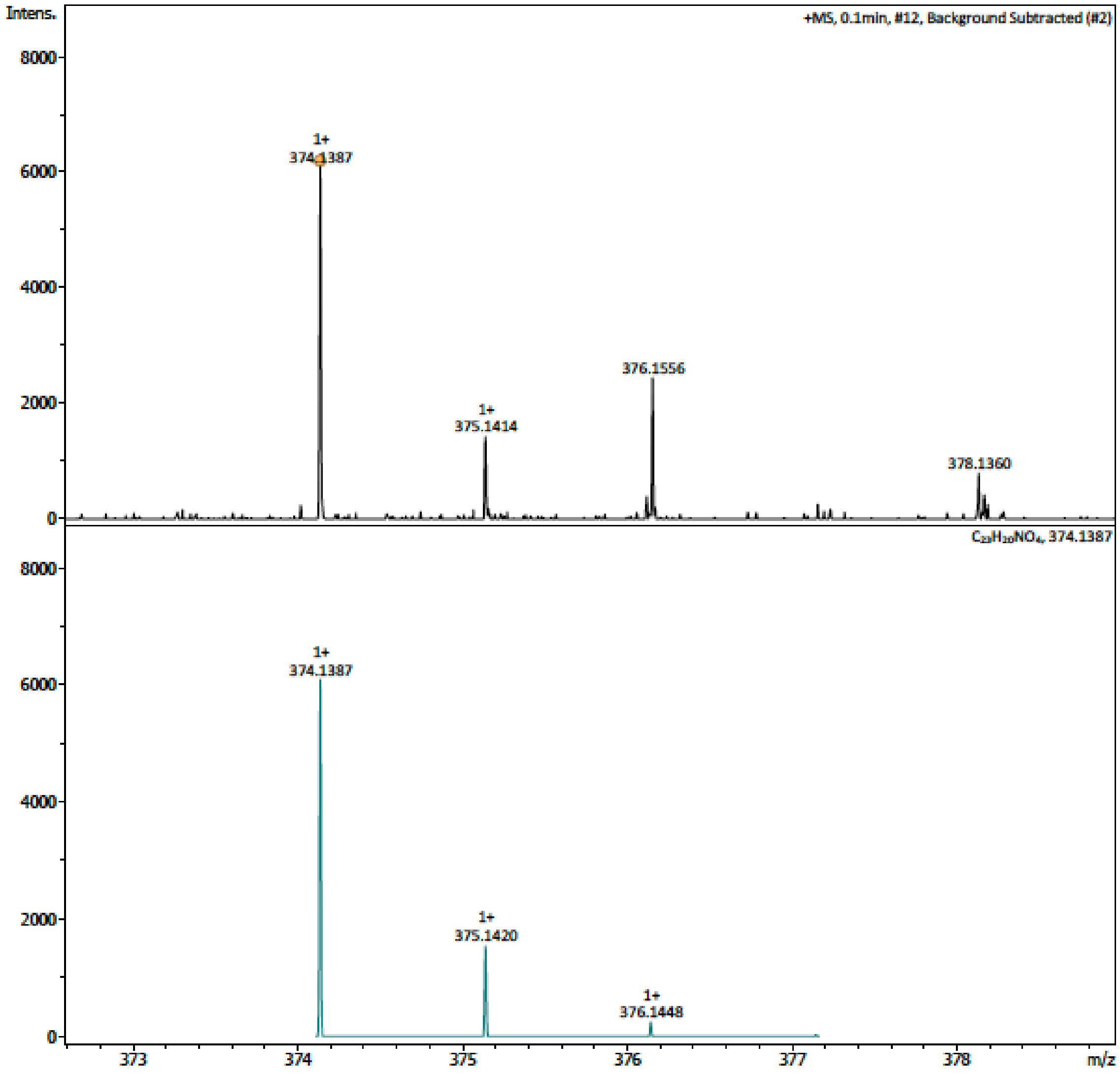

Meas. m/z	#	Ion Formula	Score	m/z	err [mDa]	err [ppm]	mSigma	rdb (neutral)	e⁻ Conf	N-Rule
374.1387	1	C23H20NO4	100.00	374.1387	-0.0	-0.1	26.3	15.0	even	ok

Compound 17:

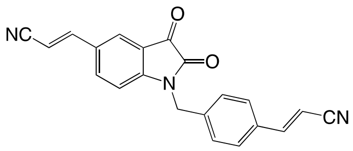

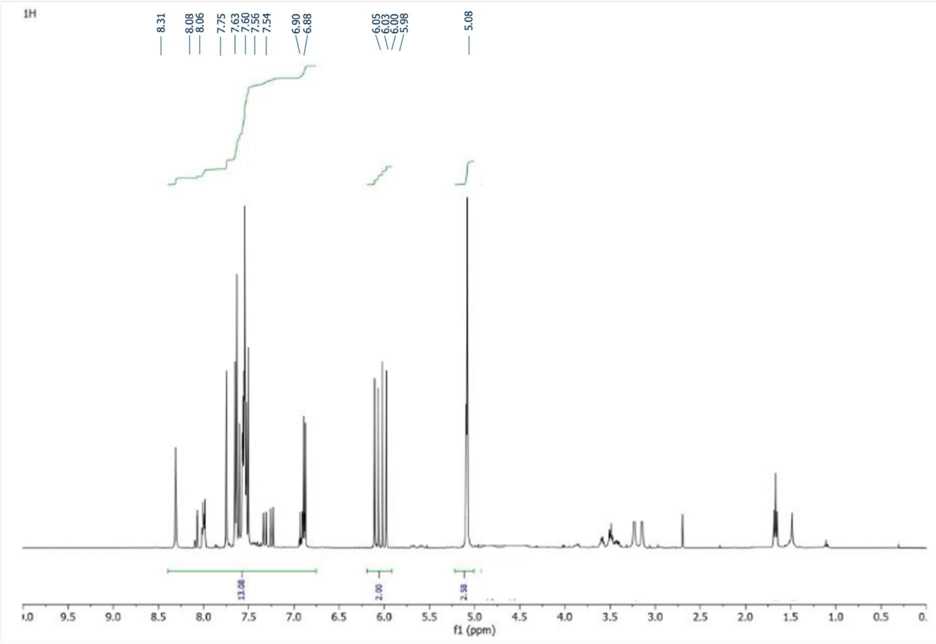

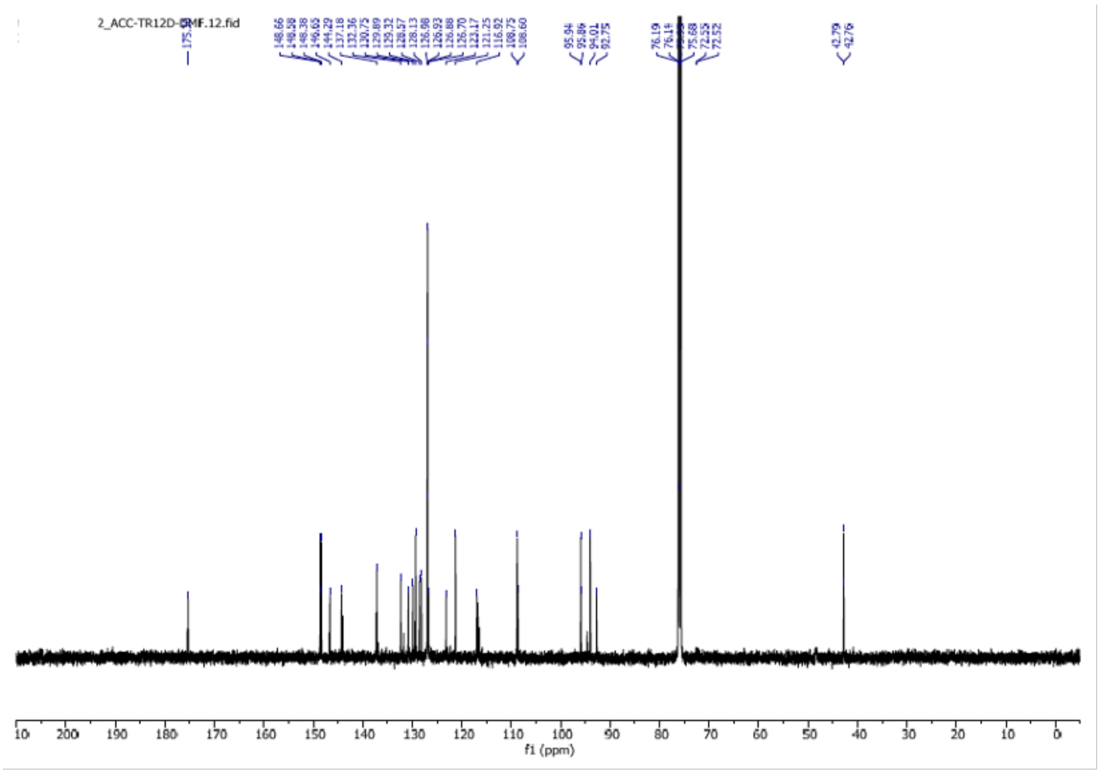

UNIDADE DE ESPECTROMETRÍA DE MASAS E PROTEÓMICA

AREA DE INFRAESTRUCTURAS DE INVESTIGACION
Edificio CACTUS * Campus Vida
15782 Santiago de Compostela
Tel. 881816242. Ext. 16242
E-mail: sxemasas@usc.es
Web:
https://www.usc.gal/es/servicios/unidades/unidad-espectrometria-masas-proteom

ESPECTRO APCI-FIA-Ion Trap

Analysis Info

Sample Name	8 APCI MS-24-0432-2-tb3 339_3_01_2266.d	Acquisition Date	28/05/2024 13:05:54
Method	2266.m	Instrument	amaZon ETD

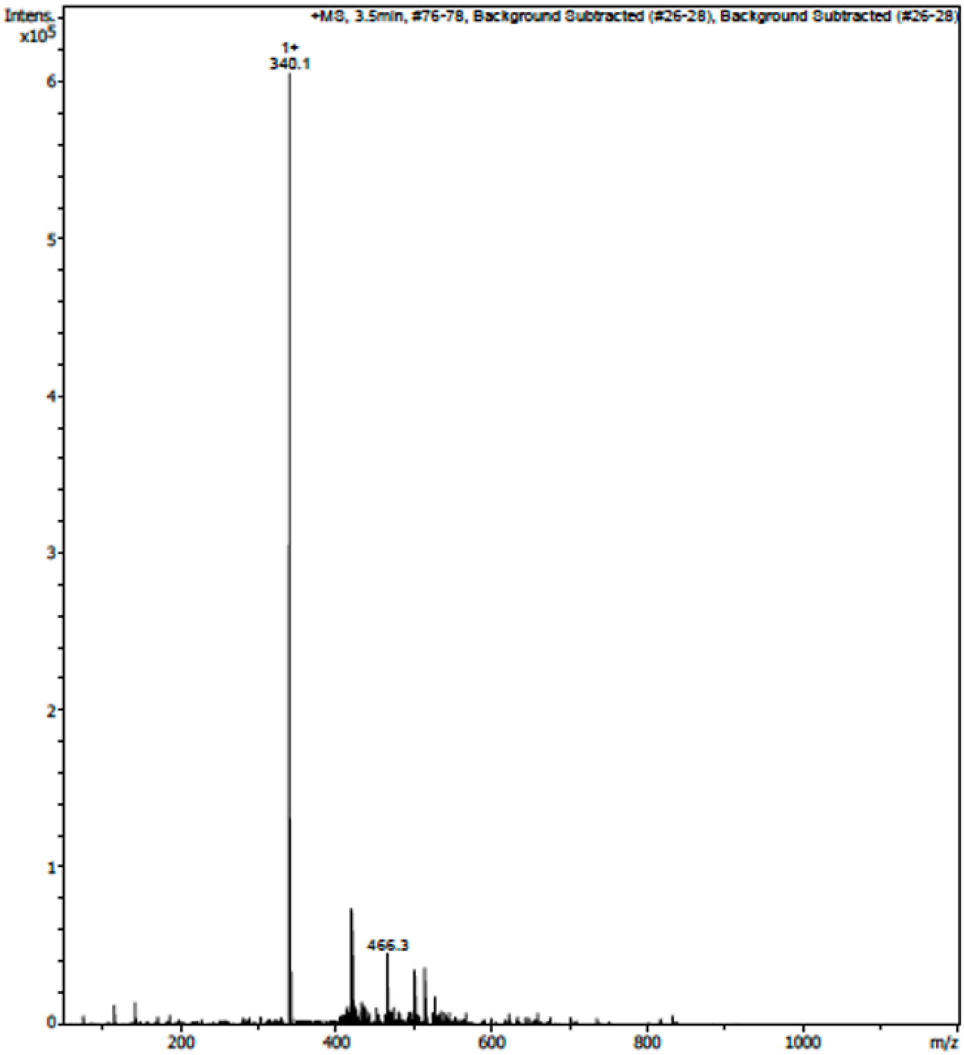

Medida De Masas Exactas

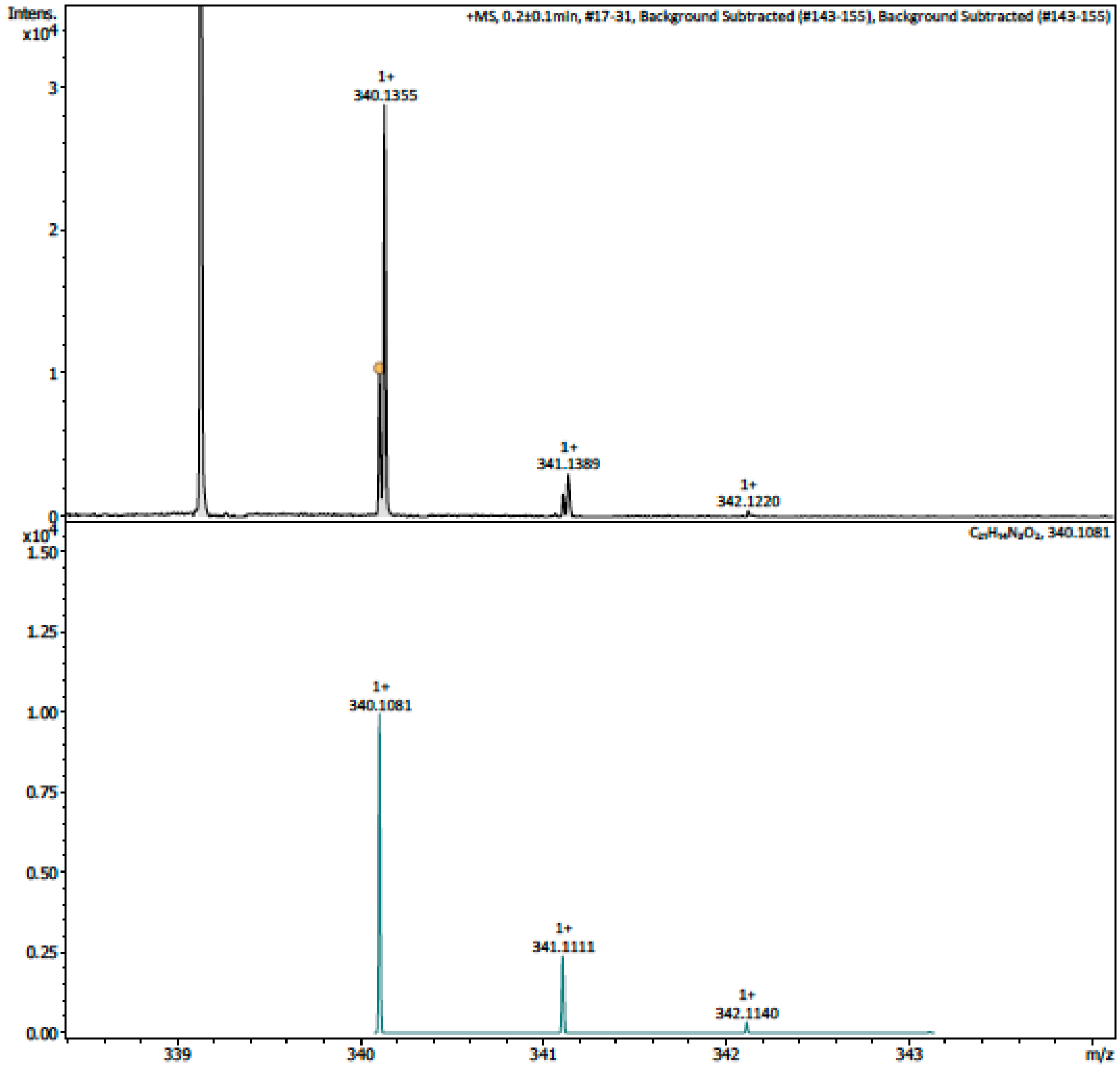

Meas. m/z	#	Ion Formula	Score	m/z	err [mDa]	err [ppm]	mSigma	rdb (neutral)	e⁻ Conf	N-Rule
340.1078	1	C21H14N3O2	100.00	340.1081	0.3	0.8	52.1	17.0	even	ok

Compound 18:

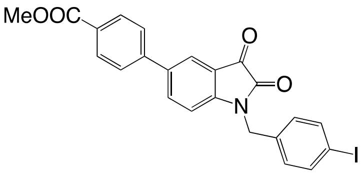
MeOOC
O
O
N
I

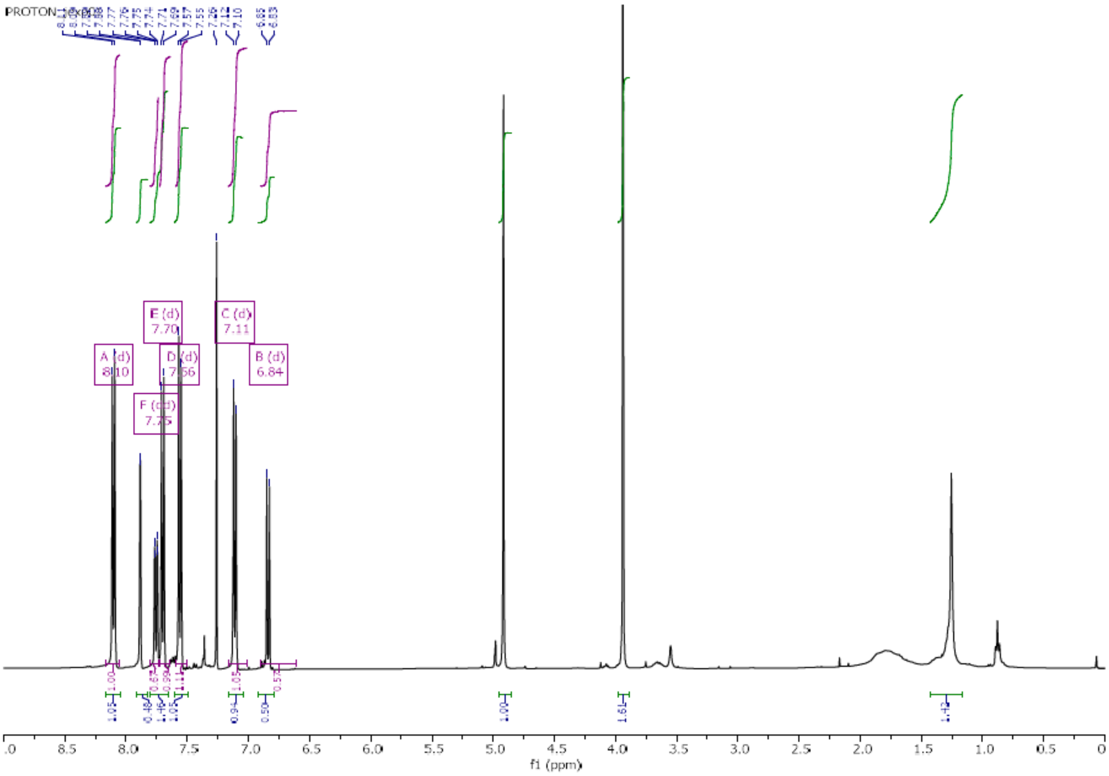
PROTON
A (d) 8.10
B (d) 6.84
C (d) 7.11
D (d) 7.56
E (d) 7.70
F (dd) 7.75
f1 (ppm)

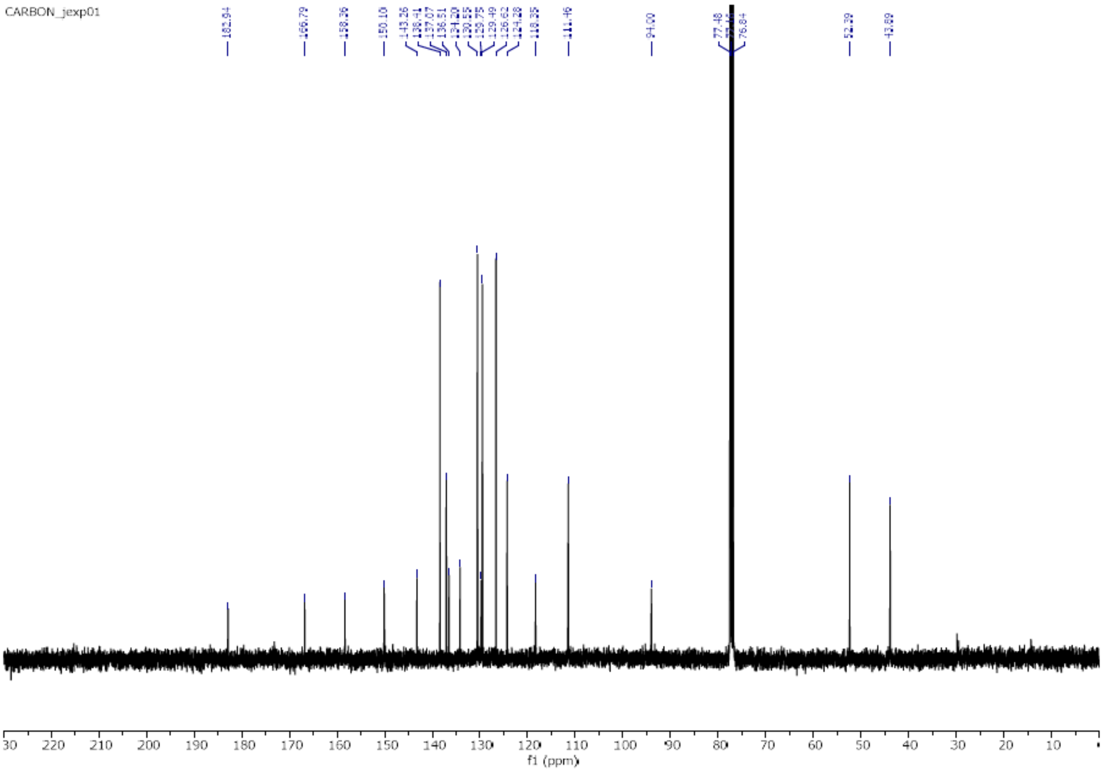
CARBON_jexp01
182.94
166.79
158.36
150.10
111.46
94.00
76.84
52.39
43.89
f1 (ppm)

UNIDADE DE ESPECTROMETRÍA DE MASAS E PROTEÓMICA

AREA DE INFRAESTRUCTURAS DE INVESTIGACION
Edificio CACTUS * Campus Vida
15782 Santiago de Compostela
Tel. 881816242. Ext. 16242
E-mail: sxemasas@usc.es
Web: https://www.usc.gal/es/servicios/unidades/unidad-espectrometria-masas-proteom

ESPECTRO APCI-FIA-Ion Trap

Analysis Info

Sample Name 20 APCI MS24-0283-5 497_16_01_2096.d
Method 2096.m
Acquisition Date 16/04/2024 20:53:49
Instrument amaZon ETD

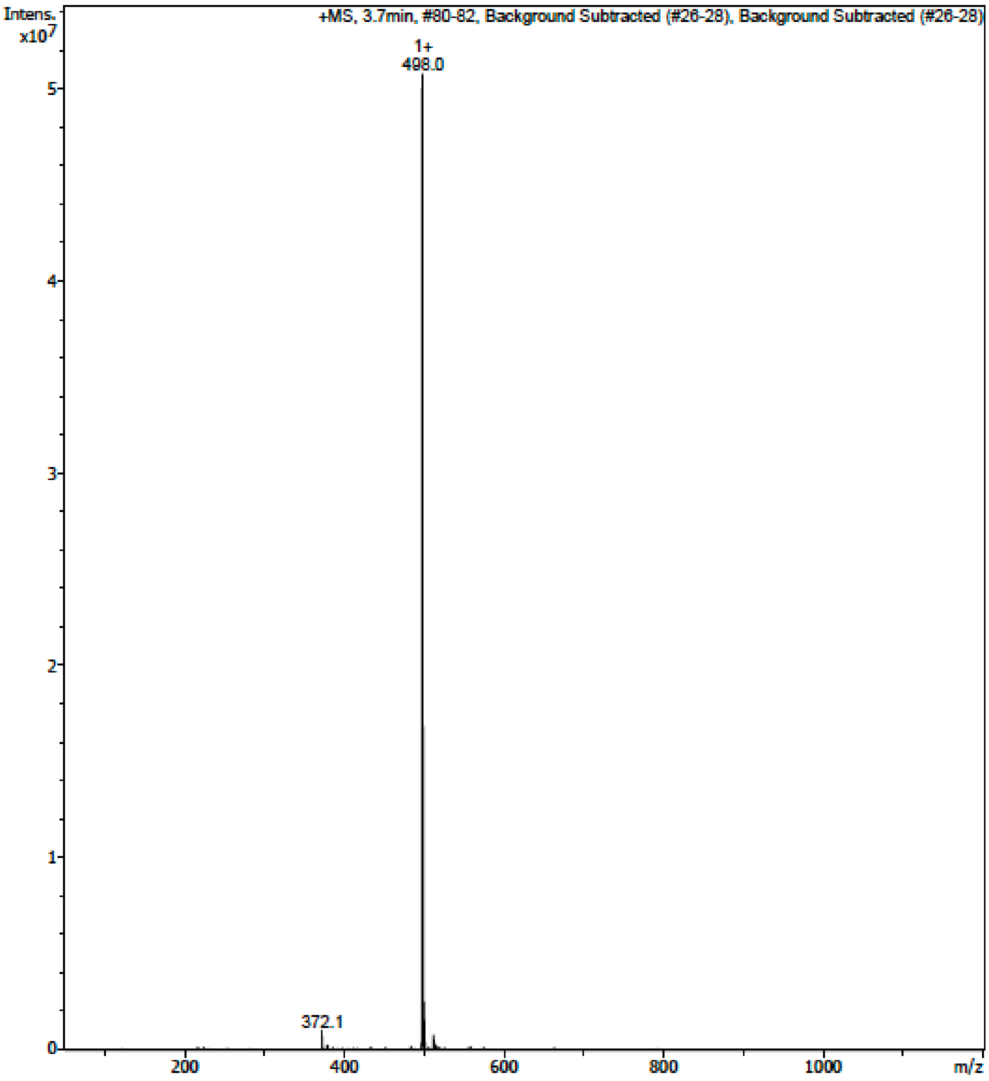

Medida De Masas Exactas

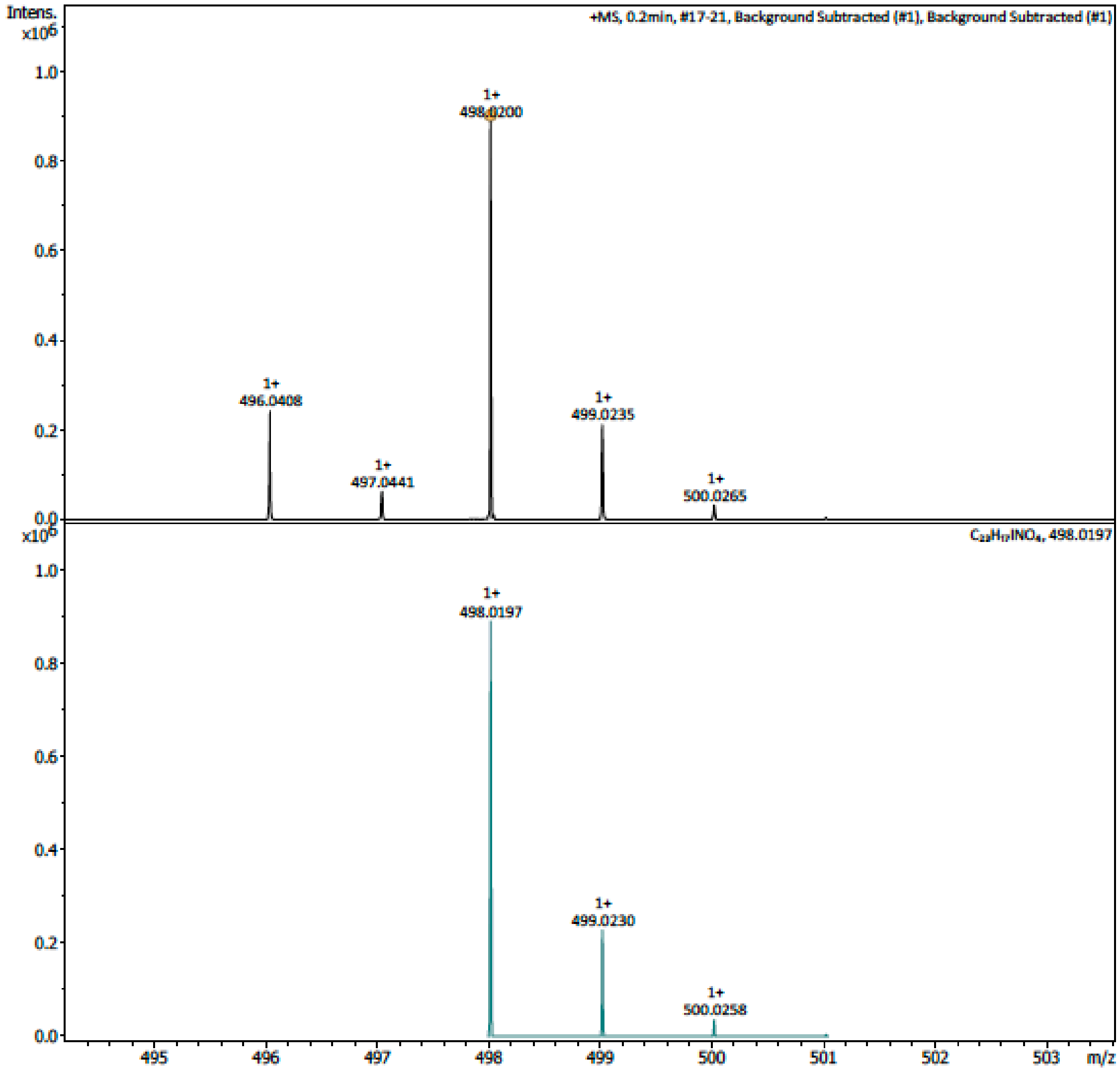

Meas. m/z	#	Ion Formula	Score	m/z	err [mDa]	err [ppm]	mSigma	rdb (neutral)	e^- Conf	N-Rule
498.0200	1	C21H15IN4O3	48.89	498.0183	-1.7	-3.4	4.2	19.5	odd	ok
498.0200	2	C23H17INO4	94.09	498.0197	-0.4	-0.7	11.1	19.0	even	ok
498.0200	3	C22H6N6O9	83.84	498.0191	-1.0	-1.9	15.9	23.5	odd	ok
498.0200	4	C24H8N3O10	100.00	498.0204	0.4	0.7	22.0	23.0	even	ok
498.0200	5	C24H13IN5	52.44	498.0210	1.0	2.0	24.3	24.0	even	ok

Compound 19:

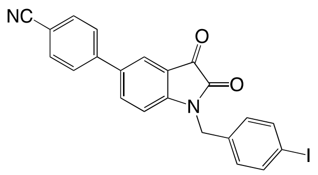

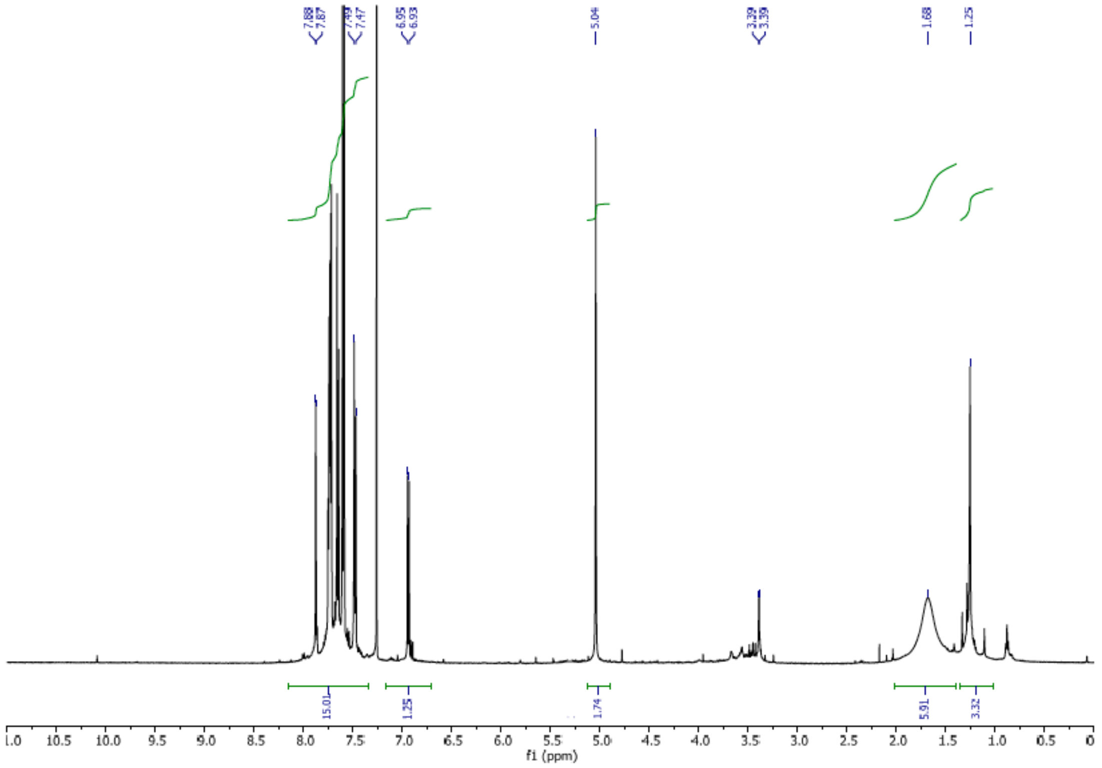

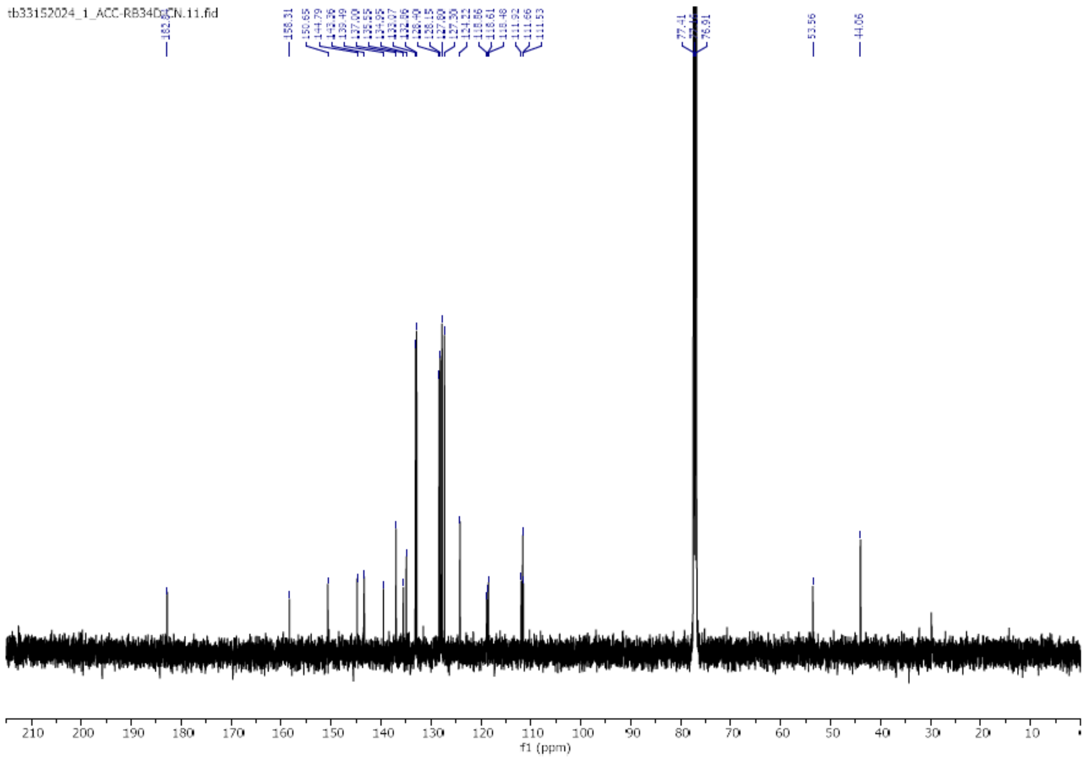

UNIDADE DE ESPECTROMETRÍA
DE MASAS E PROTEÓMICA

AREA DE INFRAESTRUCTURAS DE INVESTIGACION
Edificio CACTUS * Campus Vida
15782 Santiago de Compostela
Tel. 881816242. Ext. 16242
E-mail: sxemasas@usc.es
Web:
https://www.usc.gal/es/servicios/unidades/unidad-espectrometria-masas-proteom

ESPECTRO APCI-FIA-Ion Trap

Analysis Info

Sample Name	5 APCI MS-24-0509-2-tb3 439_3_01_2344.d	Acquisition Date	24/06/2024 11:53:25
Method	2344.m	Instrument	amaZon ETD

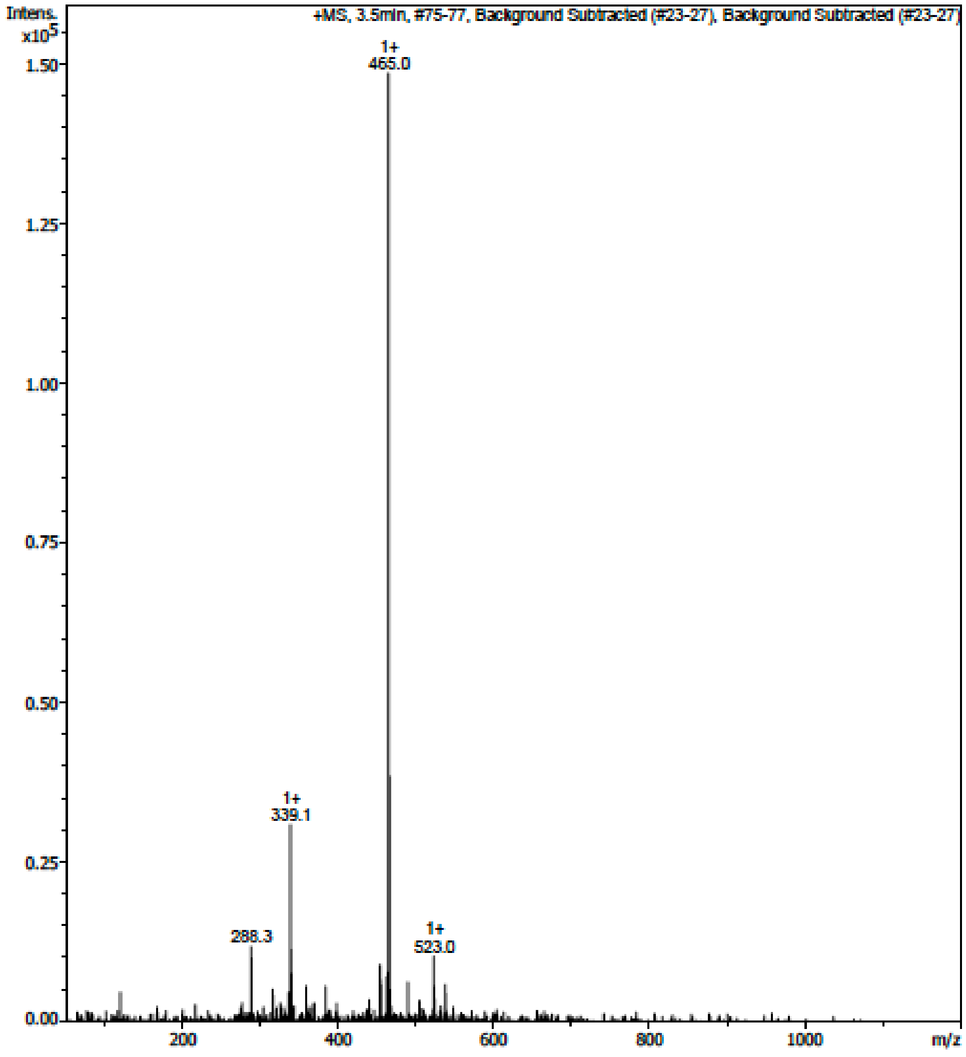

Medida De Masas Exactas

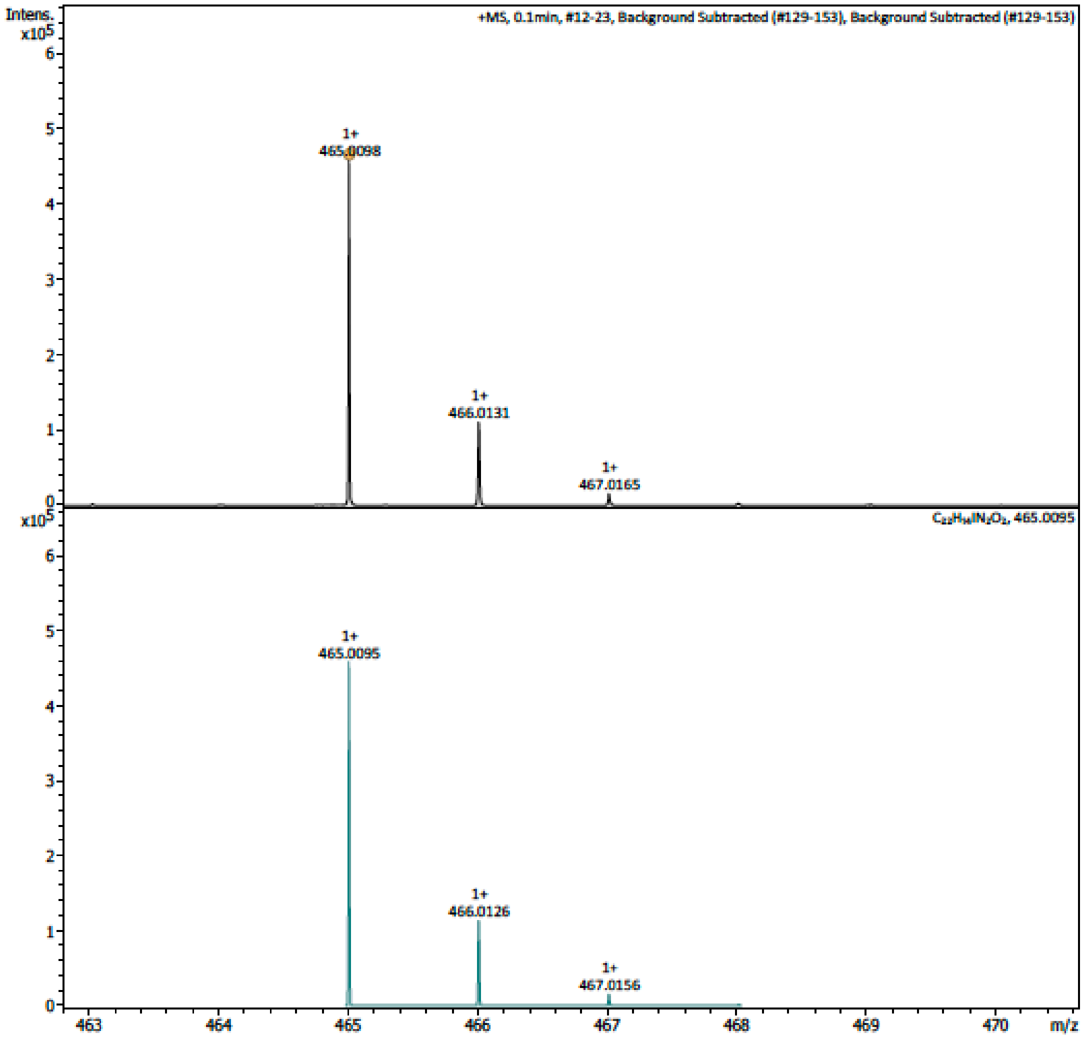

Meas. m/z	#	Ion Formula	Score	m/z	err [mDa]	err [ppm]	mSigma	rdb (neutral)	e⁻ Conf	N-Rule
465.0098	1	C20H12IN5O	42.86	465.0081	-1.7	-3.7	3.2	20.5	odd	ok
465.0098	2	C22H14IN2O2	91.19	465.0095	-0.4	-0.8	4.9	20.0	even	ok
465.0098	3	C22H9O12	81.21	465.0089	-1.0	-2.1	10.2	19.0	even	ok
465.0098	4	C21H3N7O7	80.71	465.0088	-1.0	-2.1	10.3	24.5	odd	ok
465.0098	5	C23H5N4O8	100.00	465.0102	0.3	0.7	16.4	24.0	even	ok

Compound 20:

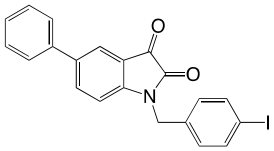
O
O
N
I

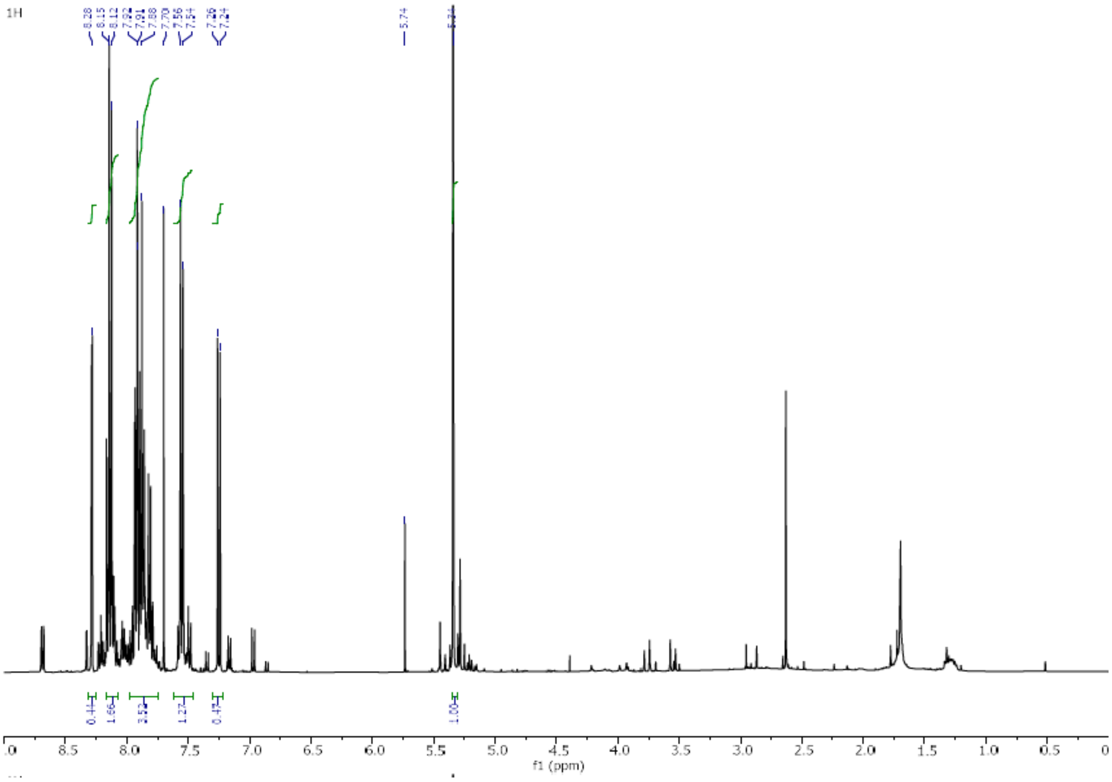
1H
8.28
8.15
8.12
7.92
7.91
7.88
7.70
7.56
7.54
7.26
7.24
5.74
0.44
1.66
3.52
1.27
1.00
8.5
8.0
7.5
7.0
6.5
6.0
5.5
5.0
4.5
4.0
3.5
3.0
2.5
2.0
1.5
1.0
0.5
0
f1 (ppm)

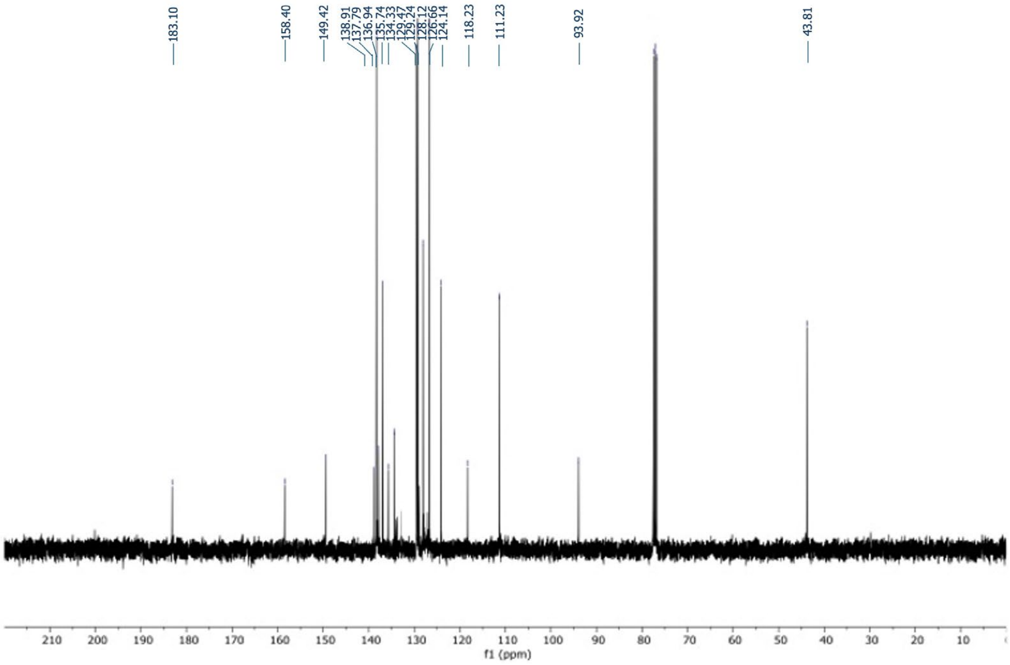
183.10
158.40
149.42
138.91
137.79
136.94
135.74
134.33
129.47
129.24
128.12
126.66
124.14
118.23
111.23
93.92
43.81
210
200
190
180
170
160
150
140
130
120
110
100
90
80
70
60
50
40
30
20
10
f1 (ppm)

USC UNIVERSIDADE DE SANTIAGO DE COMPOSTELA
UNIDADE DE ESPECTROMETRÍA DE MASAS E PROTEÓMICA

AREA DE INFRAESTRUCTURAS DE INVESTIGACION
Edificio CACTUS * Campus Vida
15782 Santiago de Compostela
Tel. 881816242. Ext. 16242
E-mail: sxamasas@usc.es
Web:
https://www.usc.gal/es/servicios/unidades/unidad-espectrometria-masas-proteom

ESPECTRO APCI-FIA-Ion Trap

Analysis Info

Sample Name	13 APCI MS24-0272-10 389_11_01_2089.d	Acquisition Date	16/04/2024 19:37:01
Method	2089.m	Instrument	amaZon ETD

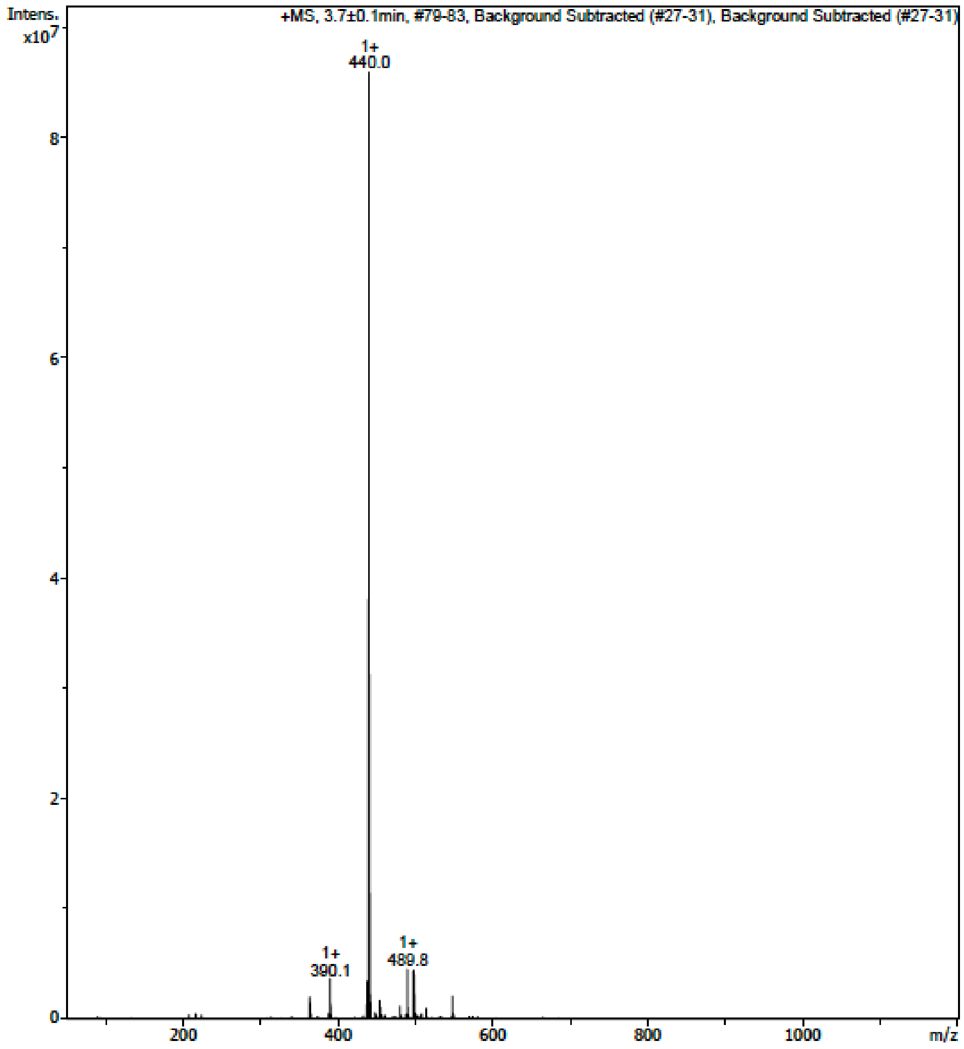

Medida De Masas Exactas

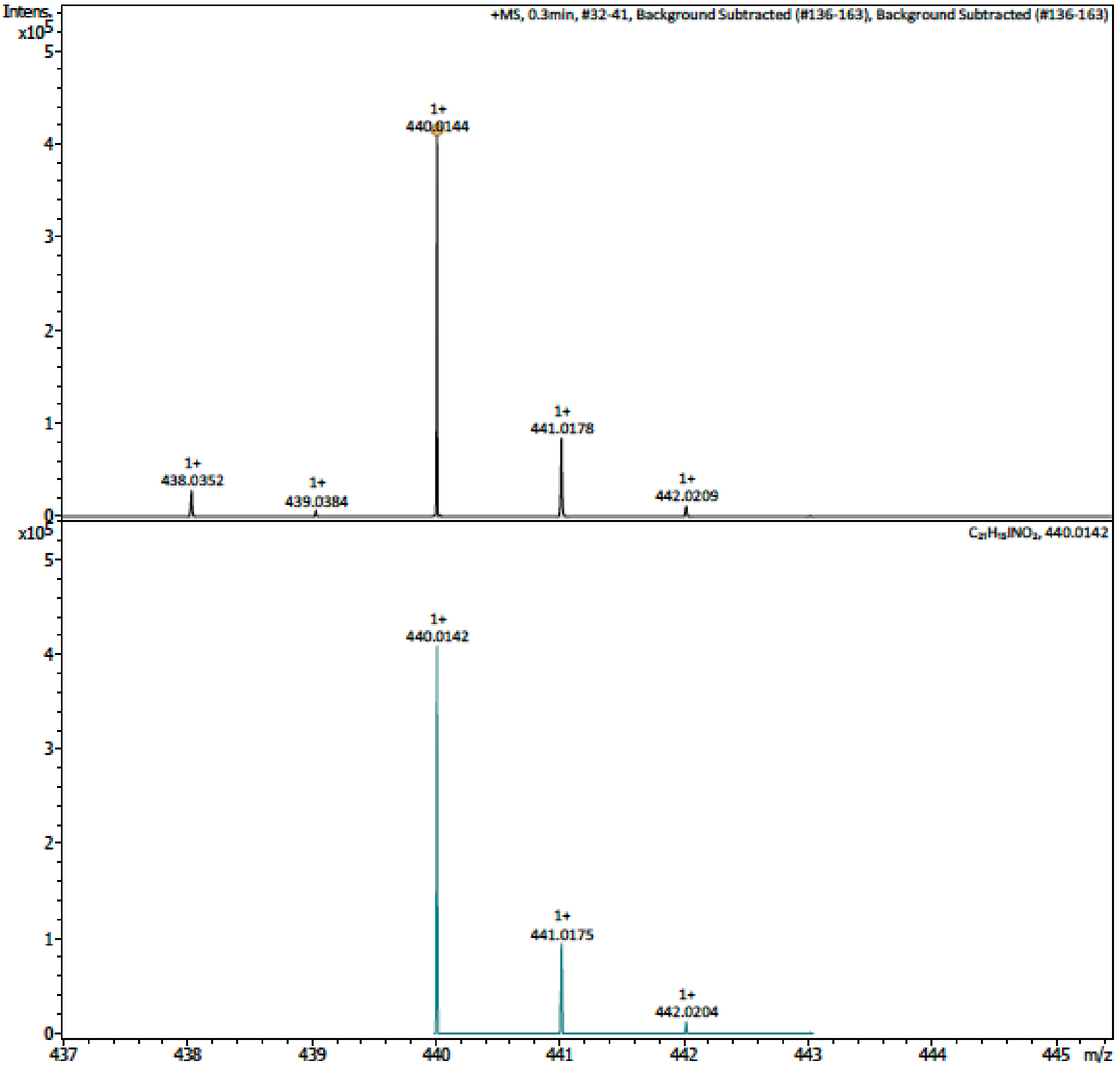

Meas. m/z	#	Ion Formula	Score	m/z	err [mDa]	err [ppm]	mSigma	rdb (neutral)	e⁻ Conf	N-Rule
440.0144	1	C19H13IN4O	54.50	440.0129	-1.5	-3.5	8.7	18.5	odd	ok
440.0144	2	C21H15INO2	100.00	440.0142	-0.2	-0.4	15.2	18.0	even	ok
440.0144	3	C20H4N6O7	65.59	440.0136	-0.8	-1.8	21.5	22.5	odd	ok
440.0144	4	C22H6N3O8	90.87	440.0149	0.6	1.3	24.8	22.0	even	ok
440.0144	5	C7H2N15O9	33.58	440.0154	1.1	2.4	42.1	15.0	even	ok

Compound 21:

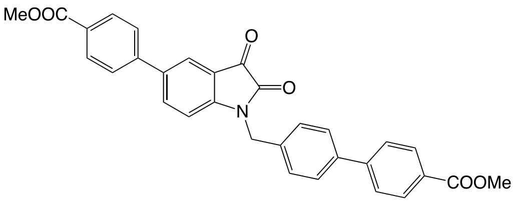

MeOOC
O
O
N
COOMe

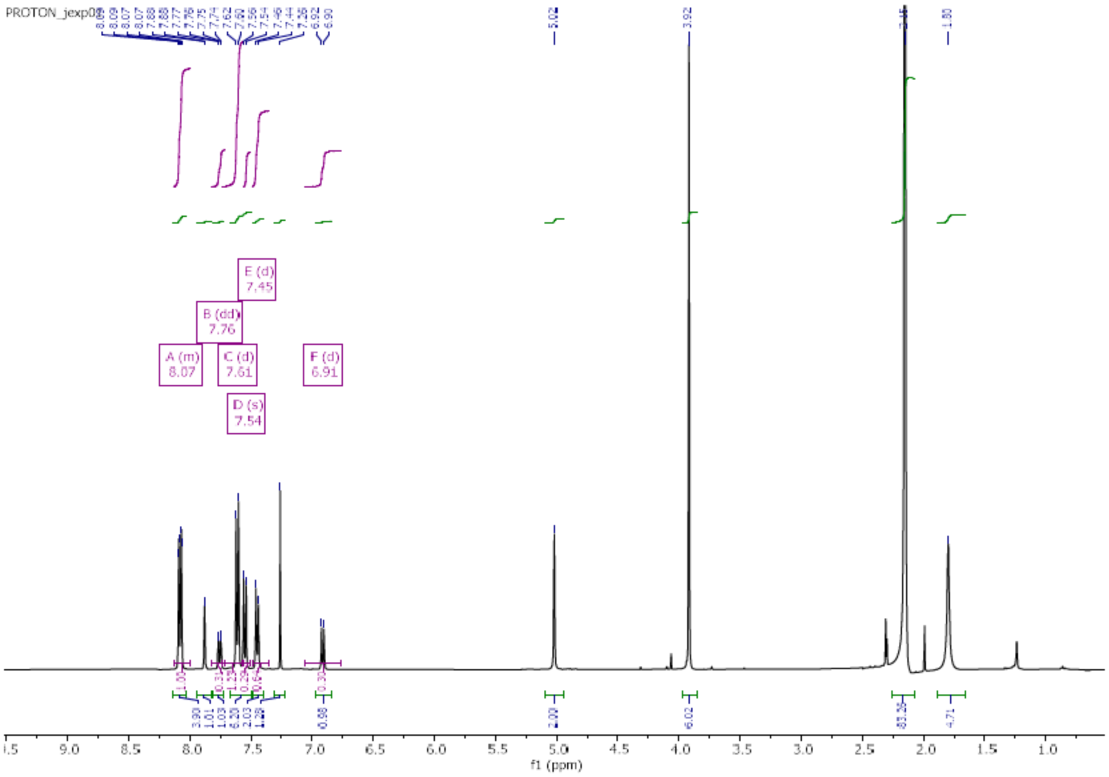

A (m)
8.07
B (dd)
7.76
C (d)
7.61
D (s)
7.54
E (d)
7.45
F (d)
6.91
5.02
3.92
1.80
9.0
8.5
8.0
7.5
7.0
6.5
6.0
5.5
5.0
4.5
4.0
3.5
3.0
2.5
2.0
1.5
1.0
f1 (ppm)

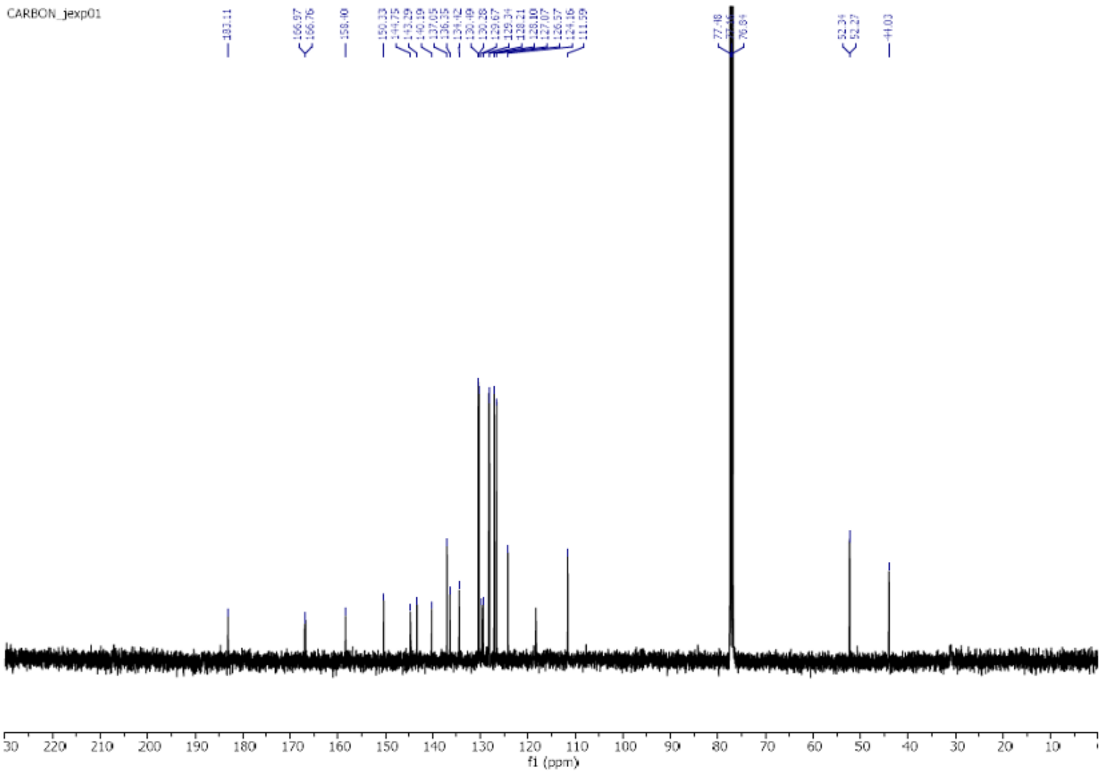

CARBON_jexp01
183.11
166.97
166.76
158.40
77.48
76.84
52.34
52.27
44.03
220
210
200
190
180
170
160
150
140
130
120
110
100
90
80
70
60
50
40
30
20
10
f1 (ppm)

UNIDADE DE ESPECTROMETRÍA
DE MASAS E PROTEÓMICA

AREA DE INFRAESTRUCTURAS DE INVESTIGACION
Edificio CACTUS * Campus Vida
15782 Santiago de Compostela
Tel. 881816242. Ext. 16242
E-mail: sxemasas@usc.es
Web:
https://www.usc.gal/es/servicios/unidades/unidad-espectrometria-masas-proteom

ESPECTRO APCI-FIA-Ion Trap

Analysis Info

Sample Name	21 APCI MS24-0283-6 505_17_01_2097.d	Acquisition Date	16/04/2024 21:04:58
Method	2097.m	Instrument	amaZon ETD

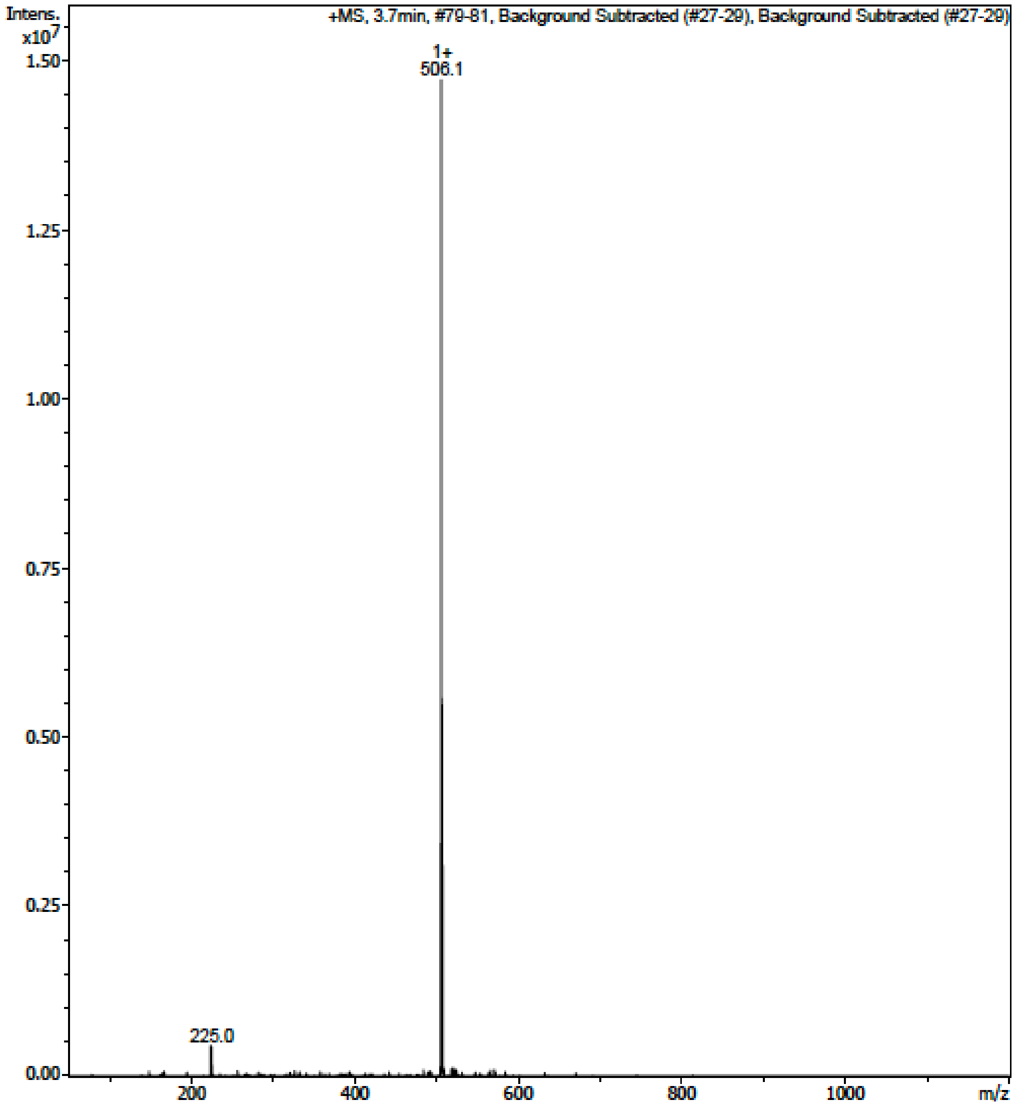

Medida De Masas Exactas

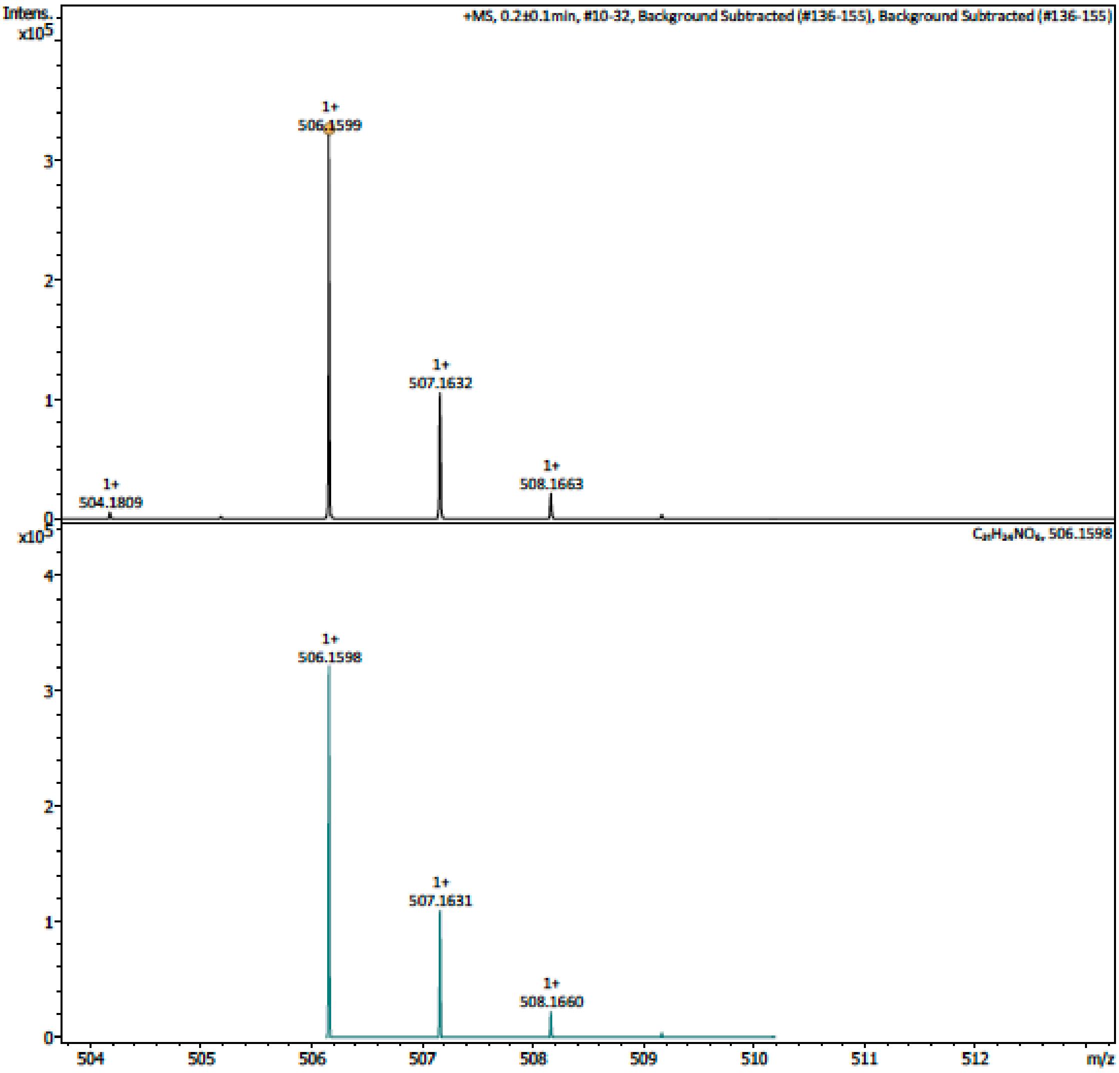

Meas. m/z	#	Ion Formula	Score	m/z	err [mDa]	err [ppm]	mSigma	rdb (neutral)	e⁻ Conf	N-Rule
506.1599	1	C29H22N4O5	54.89	506.1585	-1.4	-2.7	2.4	21.5	odd	ok
506.1599	2	C31H24NO6	100.00	506.1598	-0.0	-0.1	8.1	21.0	even	ok
506.1599	3	C28H16N11	35.50	506.1585	-1.4	-2.7	10.6	27.0	even	ok
506.1599	4	C30H18N8O	89.30	506.1598	-0.0	-0.1	14.0	26.5	odd	ok
506.1599	5	C32H20N5O2	41.94	506.1612	1.3	2.6	19.7	26.0	even	ok

Compound 22:

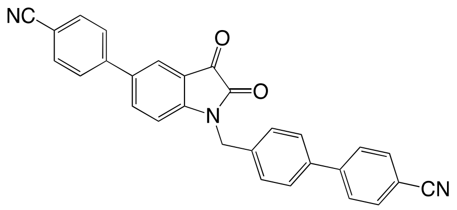
NC
O
O
N
CN

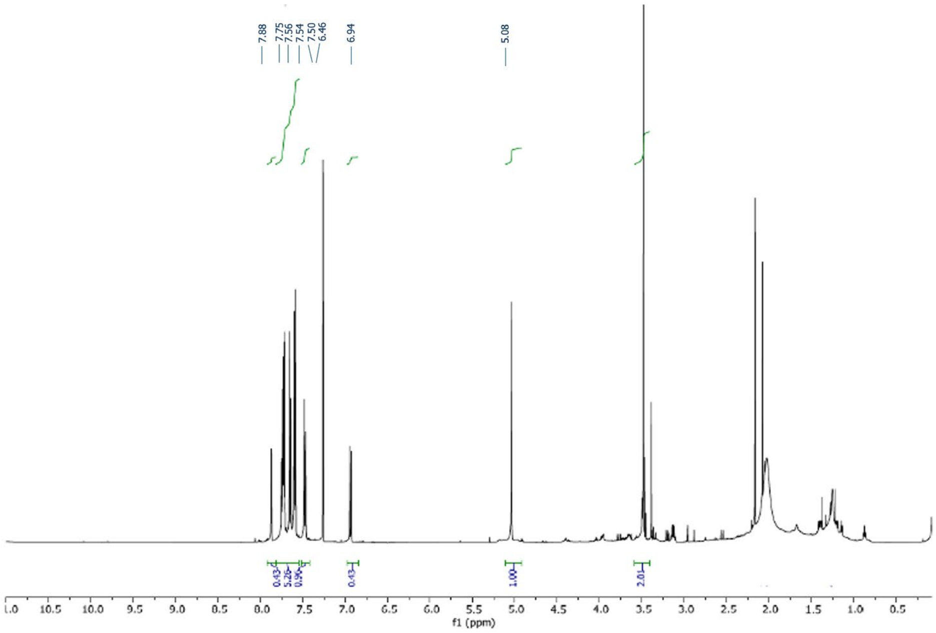
7.88
7.75
7.56
7.54
7.50
6.46
6.94
5.08
0.43
5.26
0.96
0.43
1.00
2.01
f1 (ppm)

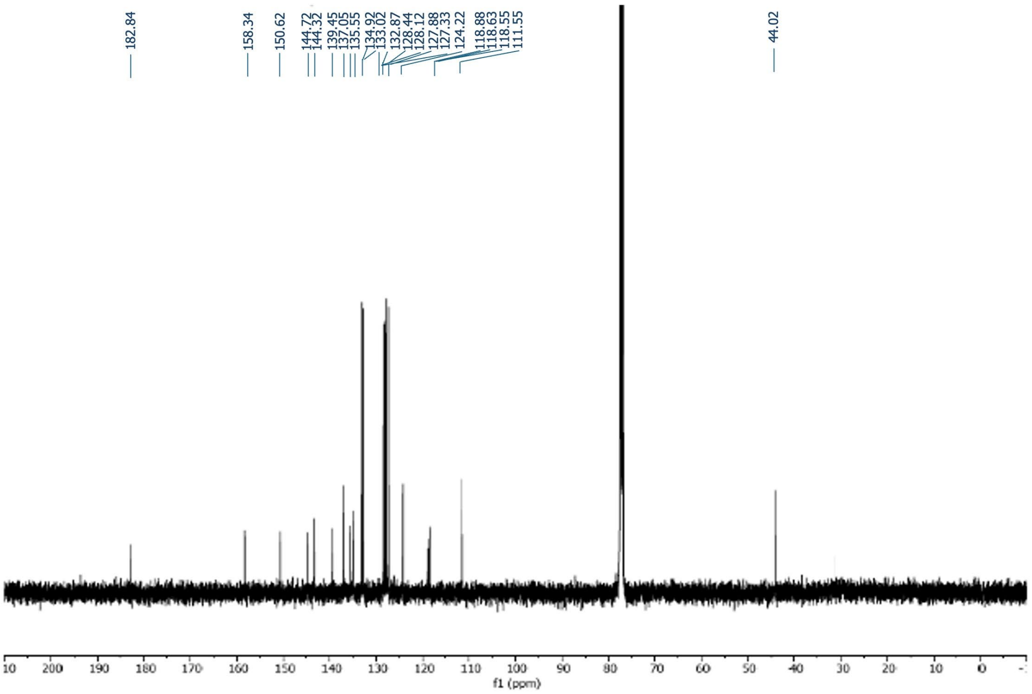
182.84
158.34
150.62
144.72
144.32
139.45
137.05
135.55
134.92
133.02
132.87
128.44
128.12
127.88
127.33
124.22
118.88
118.63
118.55
111.55
44.02
f1 (ppm)

UNIDADE DE ESPECTROMETRÍA DE MASAS E PROTEÓMICA

AREA DE INFRAESTRUCTURAS DE INVESTIGACION
Edificio CACTUS * Campus Vida
15782 Santiago de Compostela
Tel. 881816242. Ext. 16242
E-mail: sxemasas@usc.es
Web:
https://www.usc.gal/es/servicios/unidades/unidad-espectrometria-masas-proteom

ESPECTRO APCI-FIA-Ion Trap

Analysis Info

Sample Name	6 APCI MS-24-0756-4-tb3 439_5_01_2542.d	Acquisition Date	30/09/2024 15:58:28
Method	2542.m	Instrument	amaZon ETD

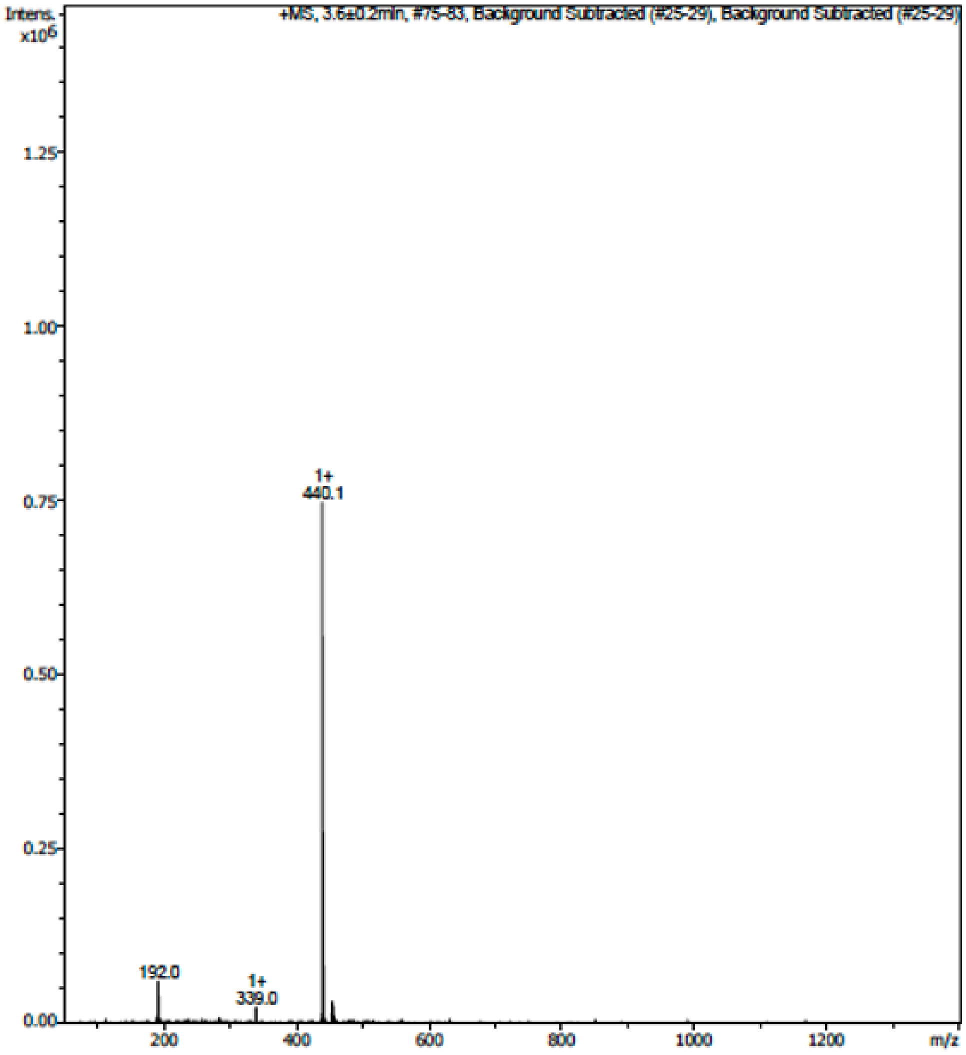

Medida De Masas Exactas

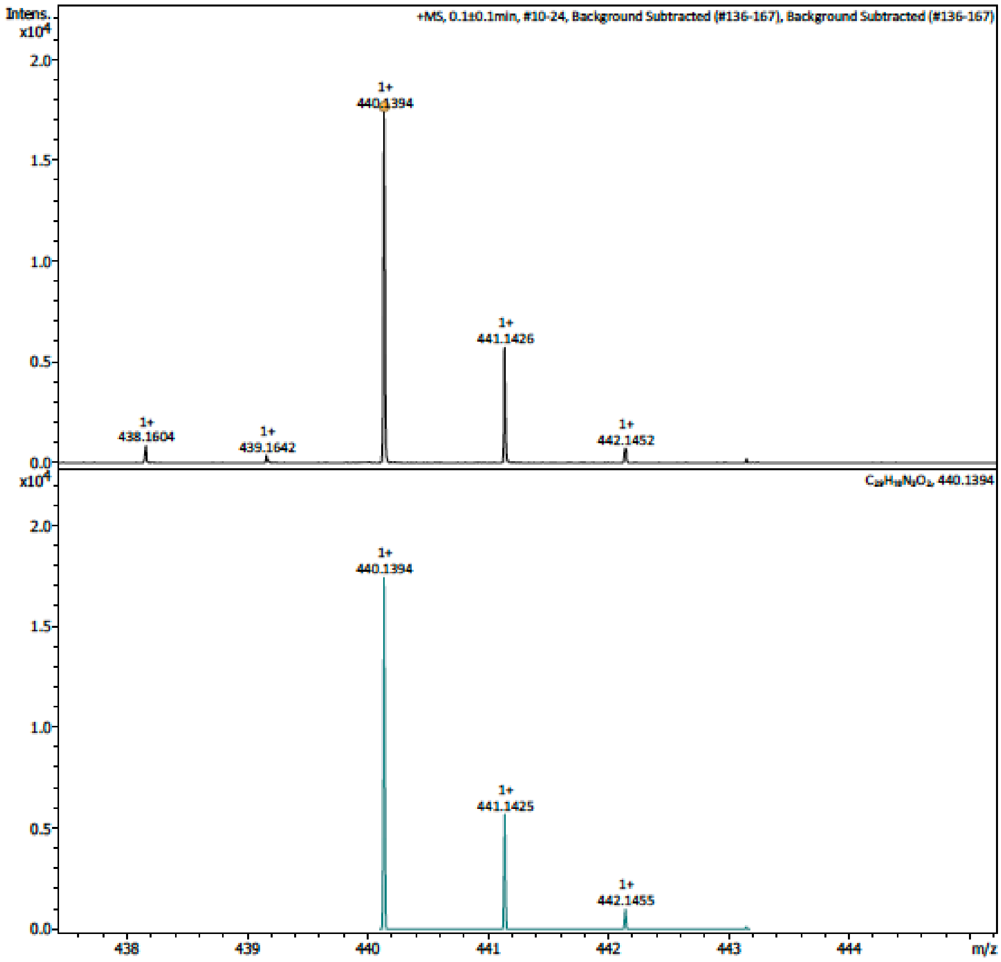

Meas. m/z	#	Ion Formula	Score	m/z	err [mDa]	err [ppm]	mSigma	rdb (neutral)	e⁻ Conf	N-Rule
440.1394	1	C29H18N3O2	100.00	440.1394	-0.1	-0.2	6.0	23.0	even	ok
440.1394	2	C27H16N6O	36.39	440.1380	-1.4	-3.3	8.7	23.5	odd	ok
440.1394	3	C31H20O3	51.12	440.1407	1.3	2.9	11.9	22.5	odd	ok

Compound 23

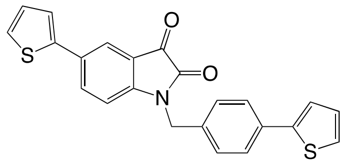

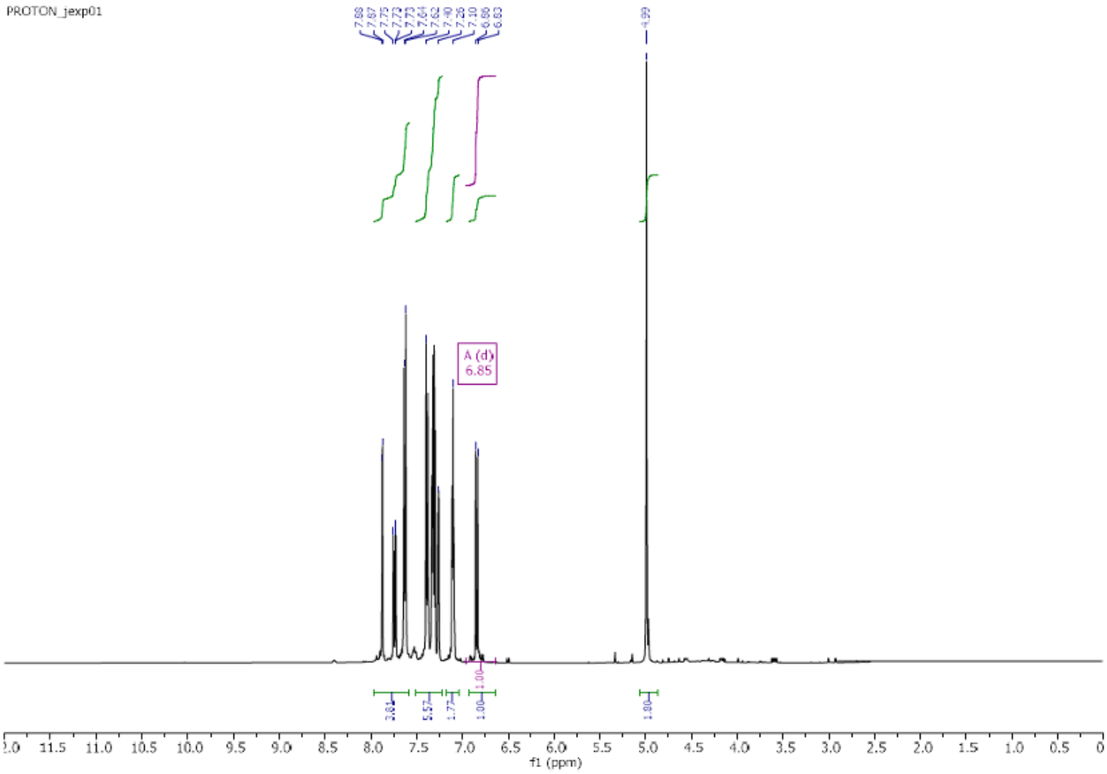

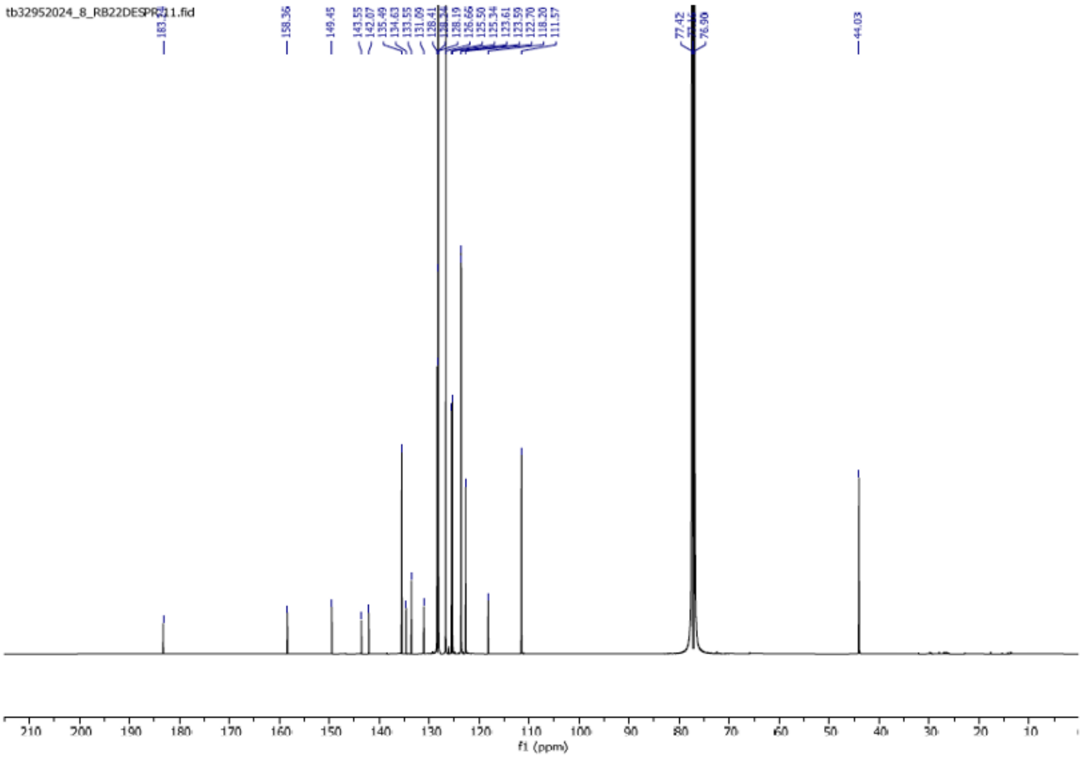

UNIDADE DE ESPECTROMETRÍA DE MASAS E PROTEÓMICA

AREA DE INFRAESTRUCTURAS DE INVESTIGACION
Edificio CACTUS * Campus Vida
15782 Santiago de Compostela
Tel. 881816242. Ext. 16242
E-mail: sxemasas@usc.es
Web:
https://www.usc.gal/es/servicios/unidades/unidad-espectrometria-masas-proteom

ESPECTRO APCI-FIA-Ion Trap

Analysis Info

Sample Name	10 APCI MS-24-0378-8-tb3 401_9_01_2213.d	Acquisition Date	16/05/2024 12:10:18
Method	2213.m	Instrument	amaZon ETD

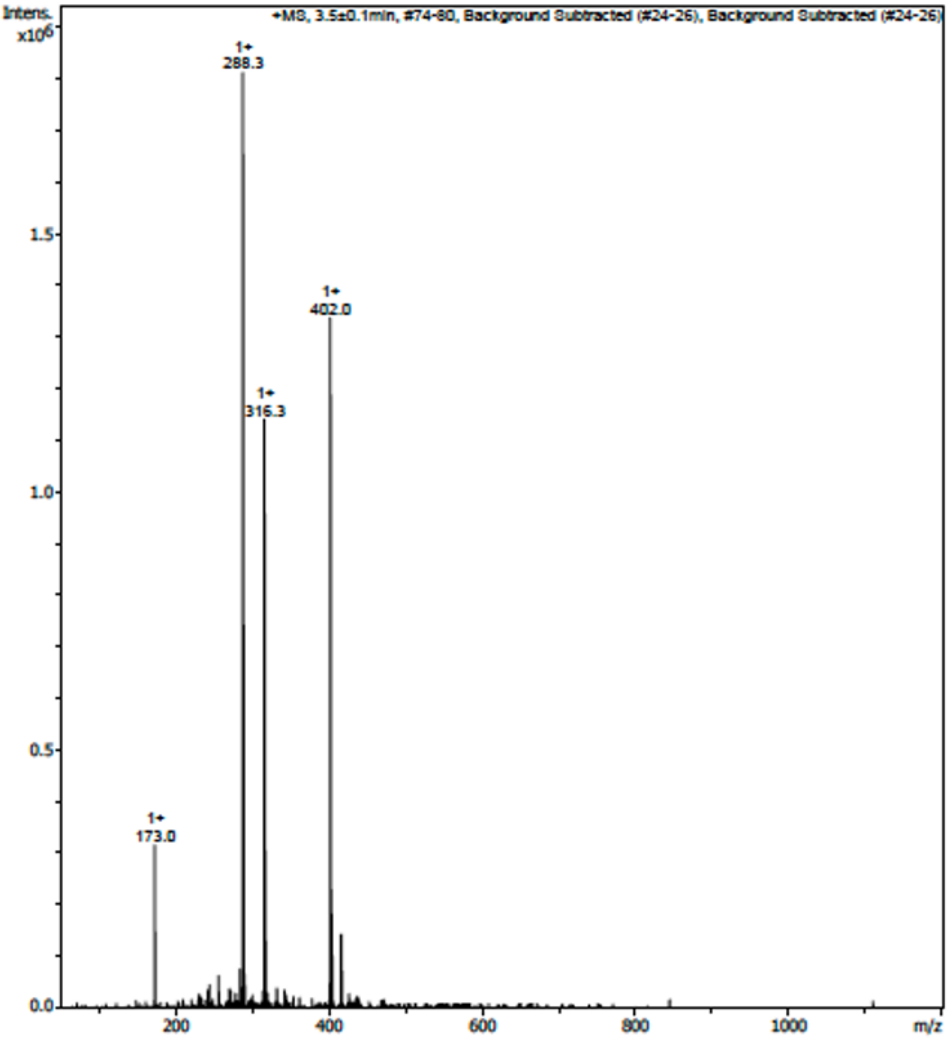

Medida De Masas Exactas

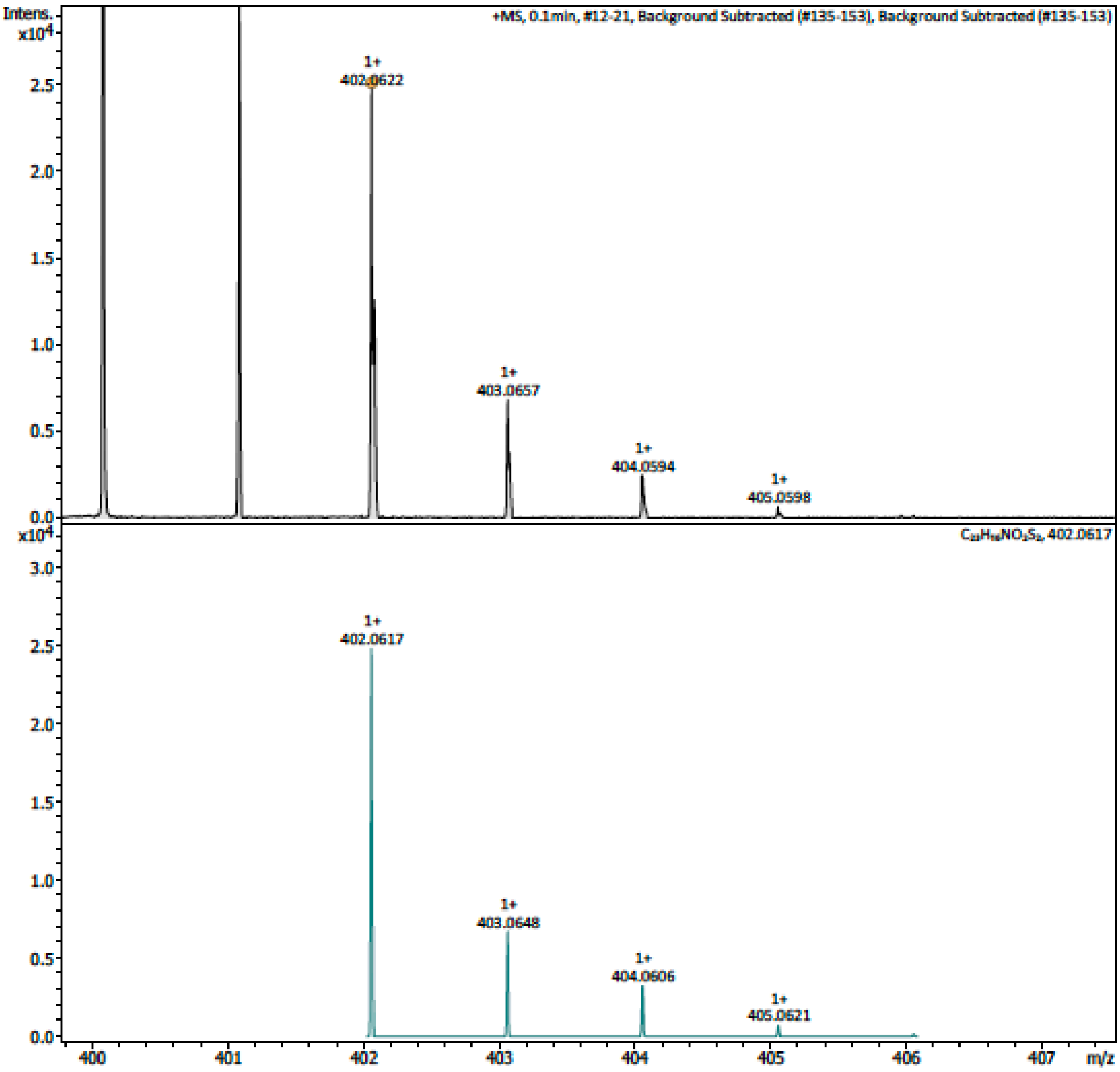

Meas. m/z	#	Ion Formula	Score	m/z	err [mDa]	err [ppm]	mSigma	rdb (neutral)	e⁻ Conf	N-Rule
402.0622	1	C23H16NO2S2	100.00	402.0617	-0.5	-1.3	15.1	21.0	even	ok
402.0622	2	C22H12NO7	28.01	402.0608	-1.4	-3.5	36.5	18.0	even	ok
402.0622	3	C16H18O10S	43.67	402.0615	-0.7	-1.8	45.3	10.5	odd	ok

Compound 24:

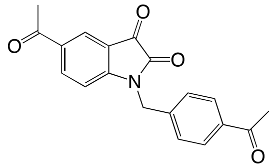

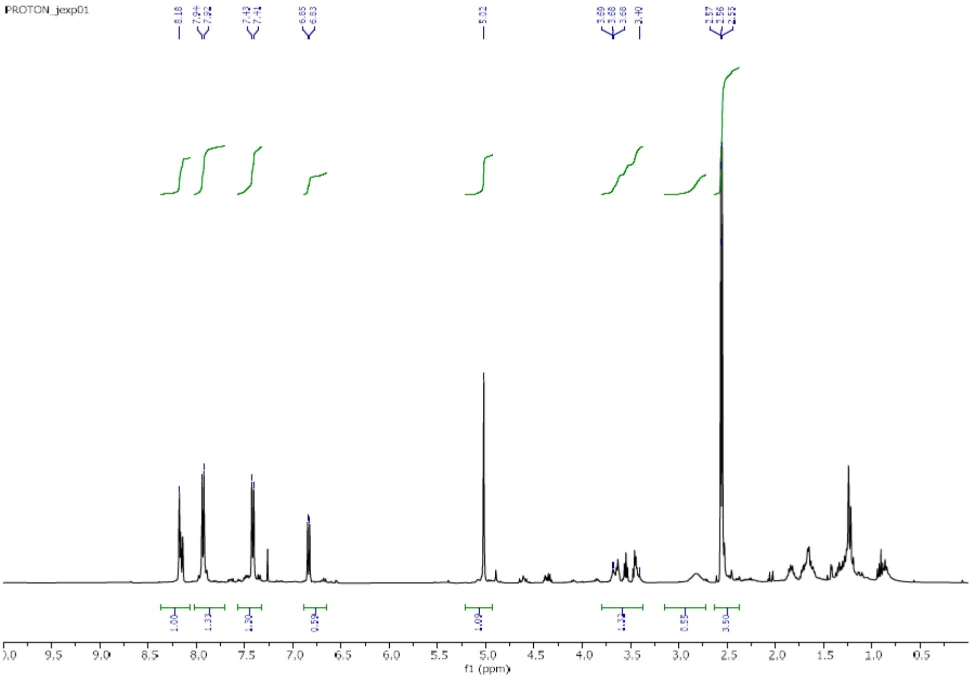

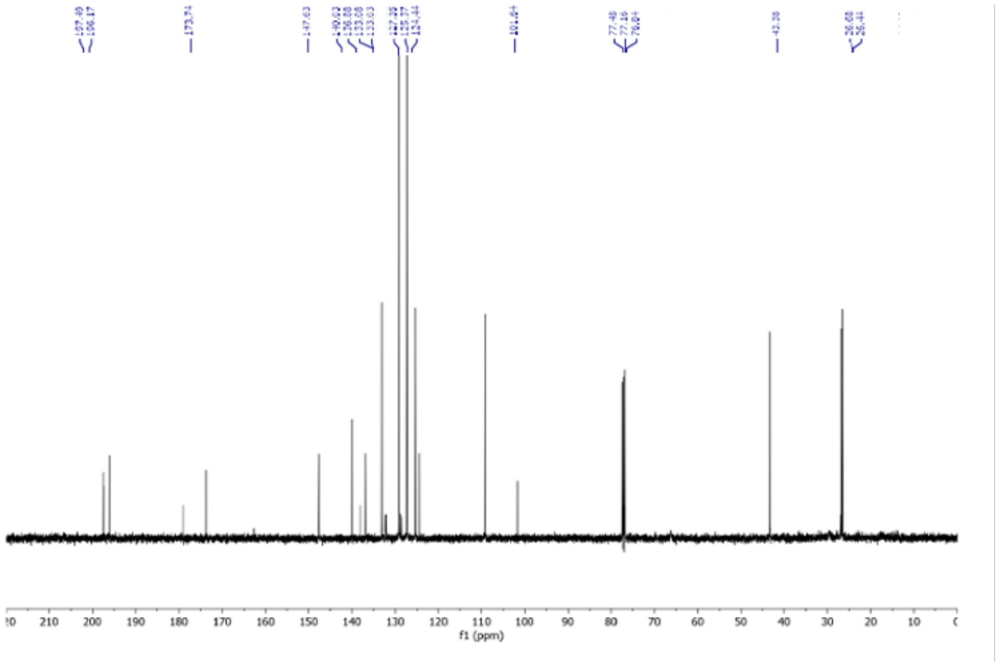

AREA DE INFRAESTRUCTURAS DE INVESTIGACION
UNIDADE DE ESPECTROMETRIA DE MASAS E PROTEÓMICA
Edificio CACTUS * Campus Vida
15782 Santiago de Compostela
Tel. 881816242 Ext. 16242
E-mail: sxemasas@usc.es
https://www.usc.gal/gl/servizos/area/infraestruturas-investigacion/servizos-equipamentos/espectrometria-masas-proteomica

ESPECTRO APCI-FIA-Ion Trap

Analysis Info

Sample Name	9 APCI MS24-0266-7 321_7_01_2068.d	Acquisition Date	15/04/2024 12:15:08
Method	2068.m	Instrument	amaZon ETD

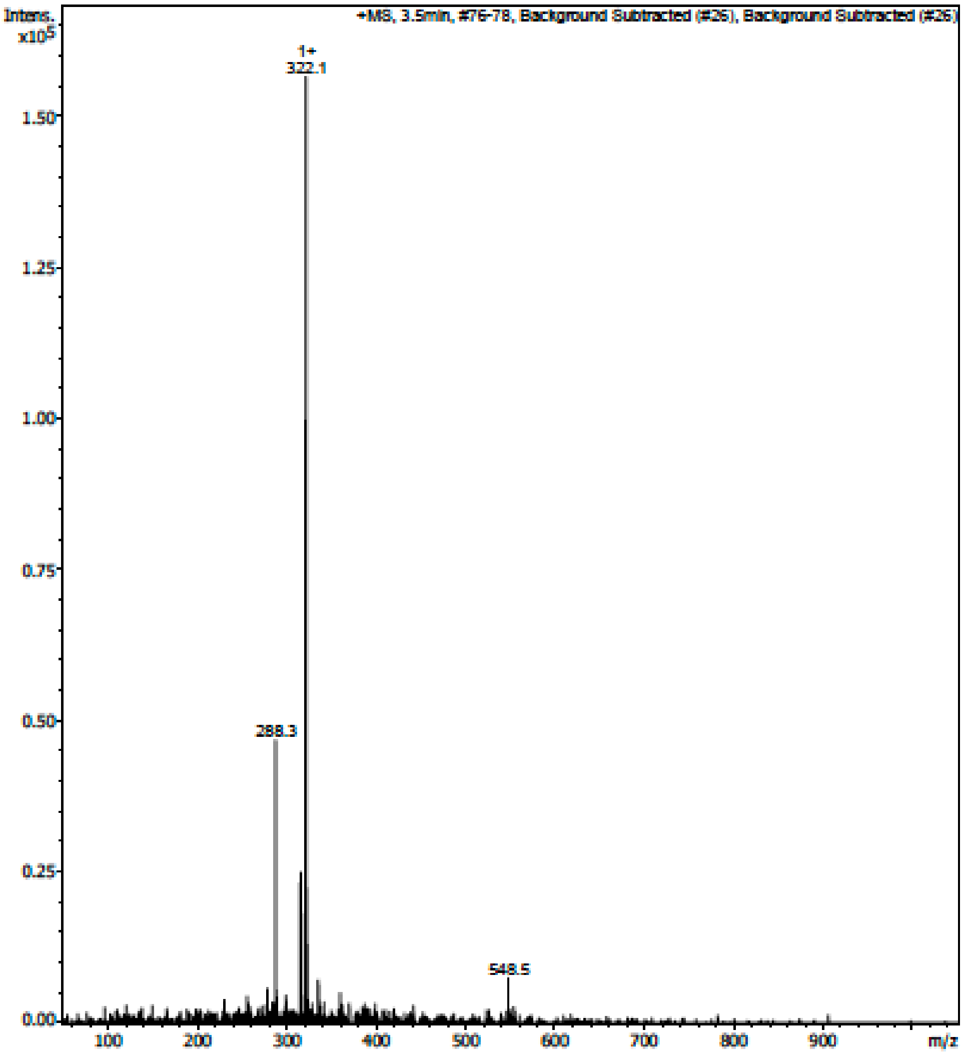

Medida De Masas Exactas

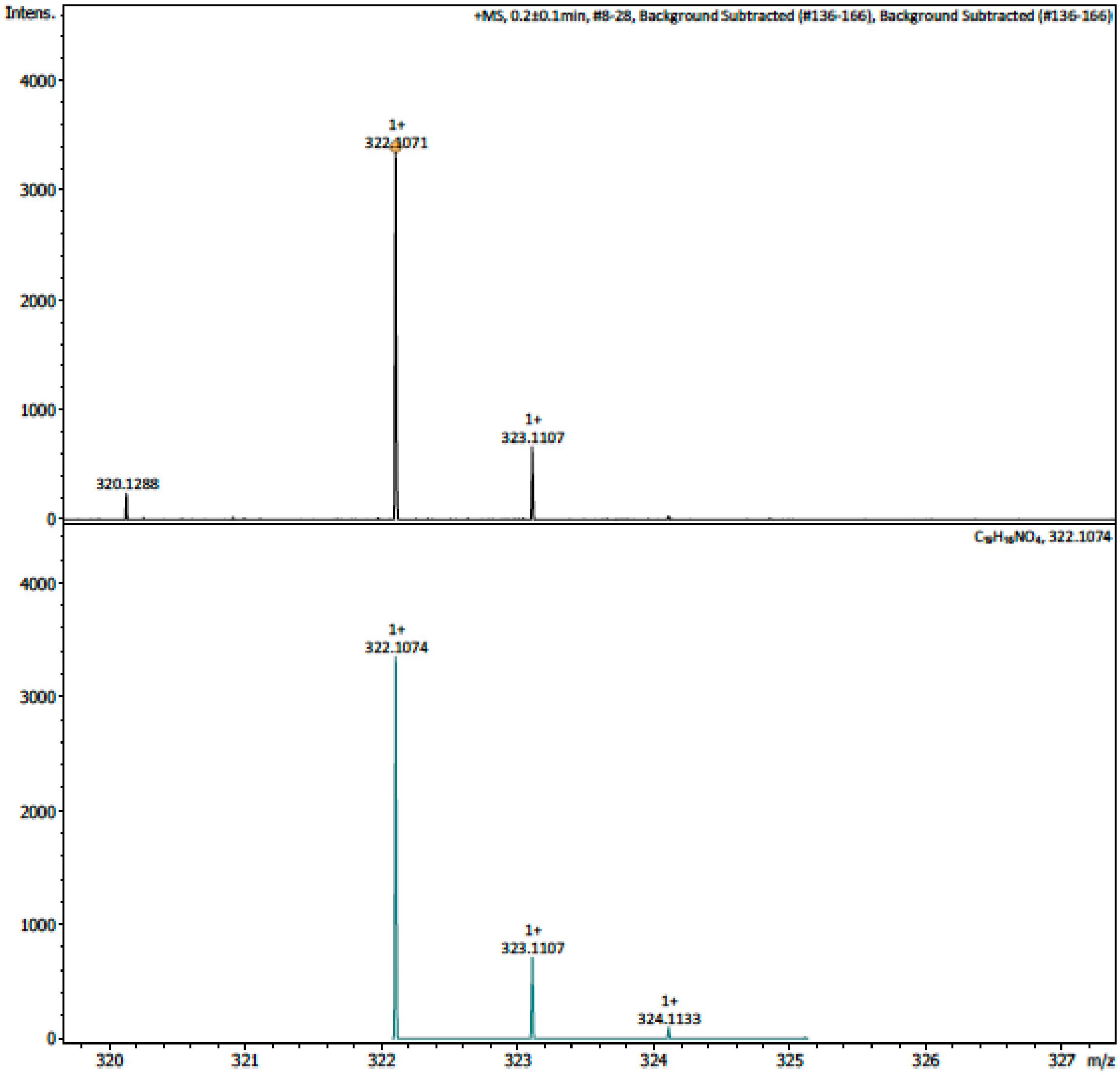

Meas. m/z	#	Ion Formula	Score	m/z	err [mDa]	err [ppm]	mSigma	rdb (neutral)	e¯ Conf	N-Rule
322.1071	1	C17H14N4O3	75.43	322.1060	-1.0	-3.2	8.6	13.5	odd	ok
322.1071	2	C19H16NO4	100.00	322.1074	0.3	0.9	13.5	13.0	even	ok
322.1071	3	C3H6N20	28.25	322.1079	0.8	2.5	53.6	11.5	odd	ok
322.1071	4	C4H12N13O5	22.41	322.1079	0.8	2.5	60.6	6.0	even	ok

Compound 25:

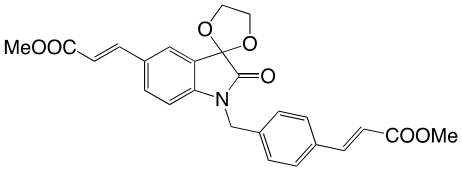

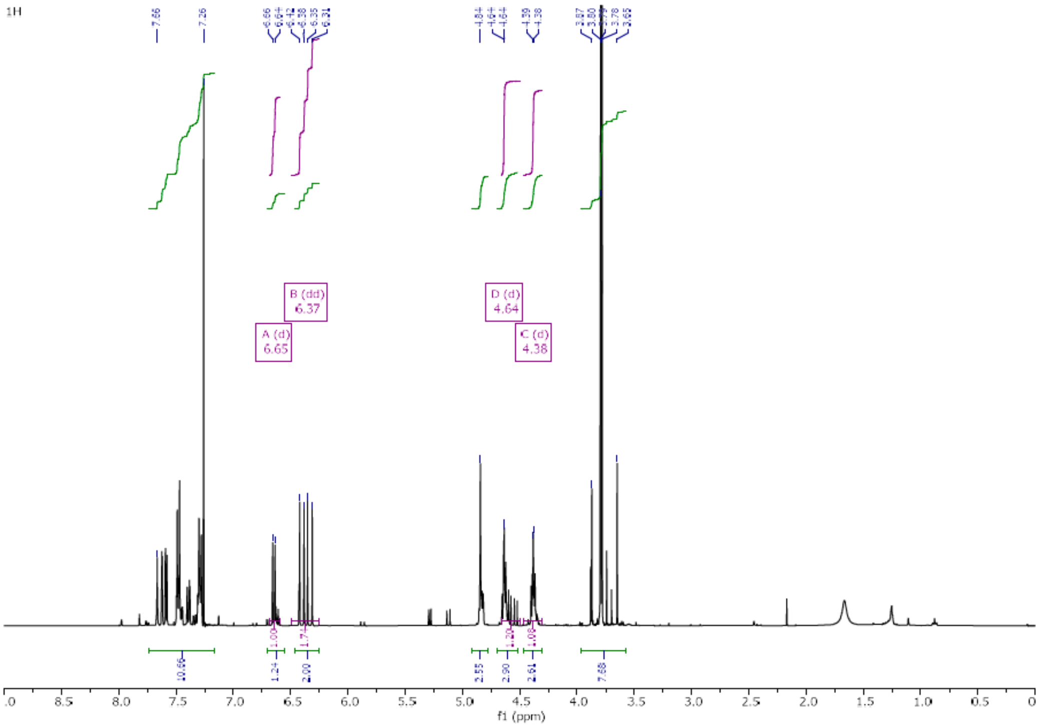

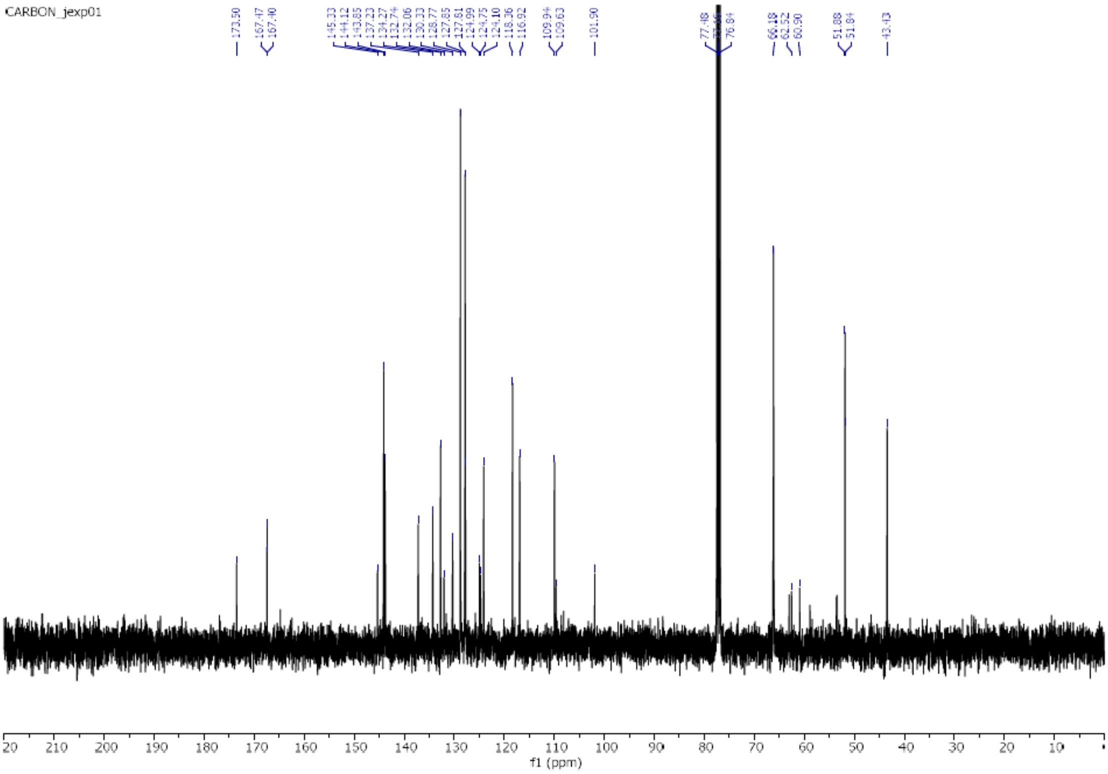

UNIDADE DE ESPECTROMETRÍA DE MASAS E PROTEÓMICA

AREA DE INFRAESTRUCTURAS DE INVESTIGACION
Edificio CACTUS * Campus Vida
15782 Santiago de Compostela
Tel. 881816242. Ext. 16242
E-mail: sxmasas@usc.es
Web
https://www.usc.gal/es/servicios/unidades/unidad-espectrometria-masas-proteom

ESPECTRO APCI-FIA-Ion Trap

Analysis Info

Sample Name	9 APCI MS24-0272-6 449_7_01_2085.d	Acquisition Date	16/04/2024 18:52:30
Method	2085.m	Instrument	amaZon ETD

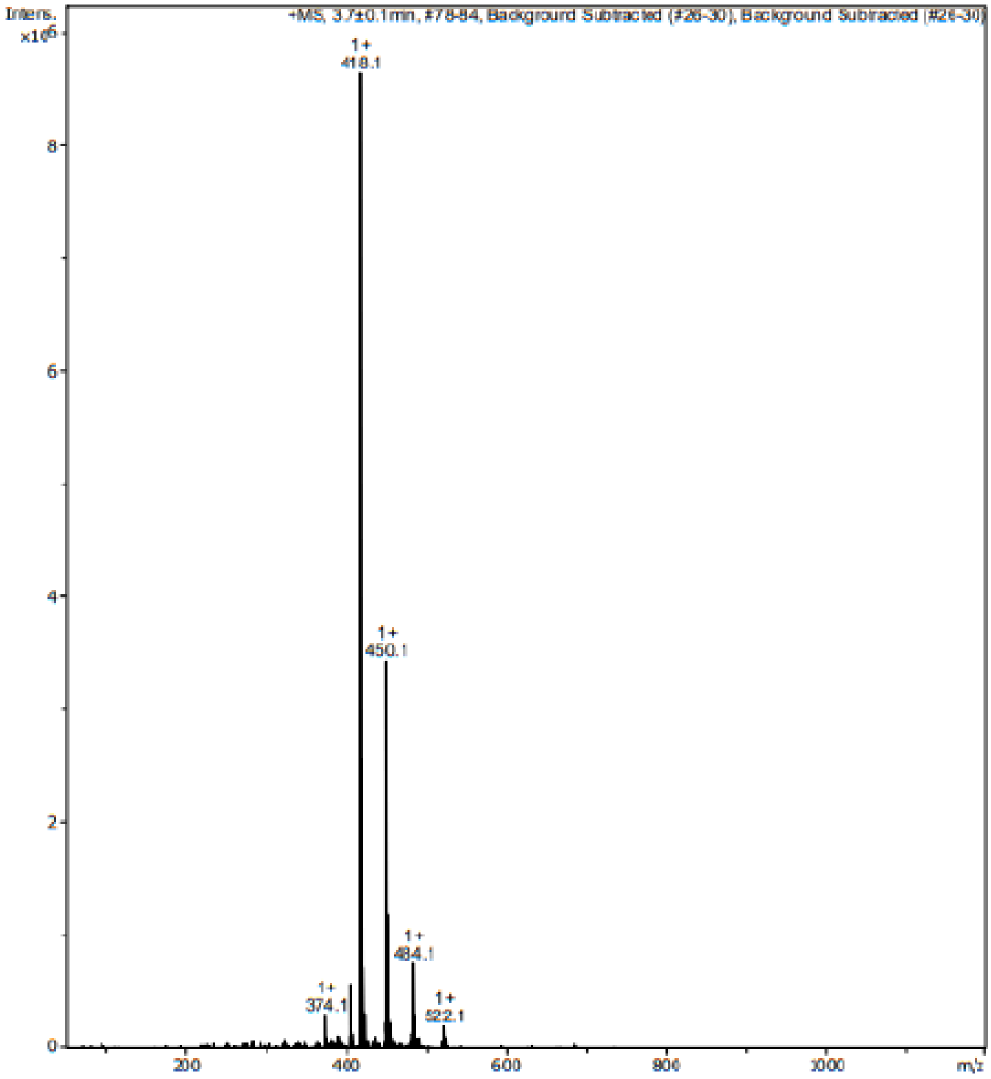

Medida De Masas Exactas

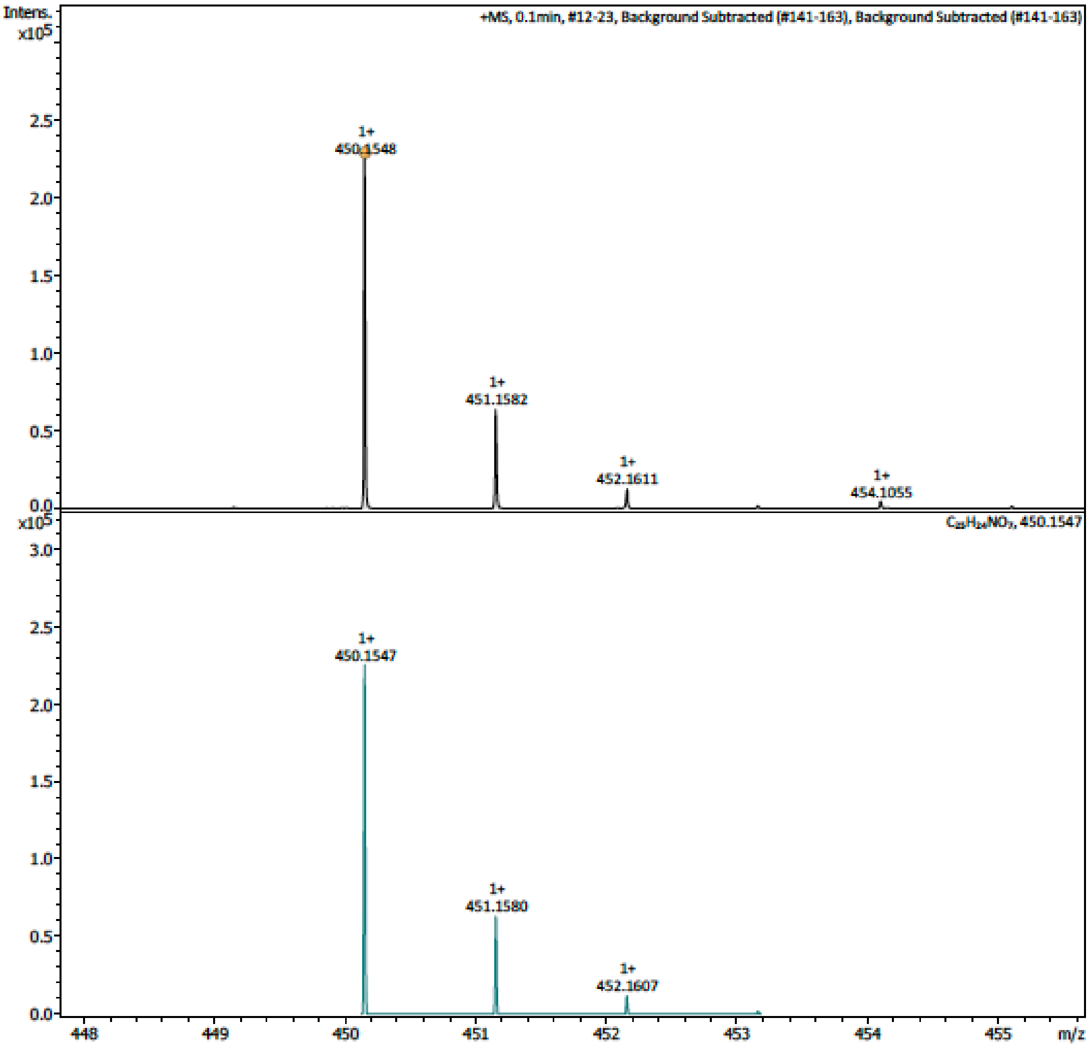

Meas. m/z	#	Ion Formula	Score	m/z	err [mDa]	err [ppm]	mSigma	rdb (neutral)	e⁻ Conf	N-Rule
450.1548	1	C25H24NO7	100.00	450.1547	-0.0	-0.1	1.4	15.0	even	ok
450.1548	2	C23H22N4O6	44.84	450.1534	-1.4	-3.1	7.6	15.5	odd	ok
450.1548	3	C24H18N8O2	67.15	450.1547	-0.0	-0.1	7.8	20.5	odd	ok
450.1548	4	C22H16N11O	33.18	450.1534	-1.4	-3.1	8.1	21.0	even	ok
450.1548	5	C26H20N5O3	32.14	450.1561	1.3	2.9	12.5	20.0	even	ok

Compound 26:

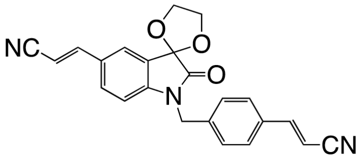

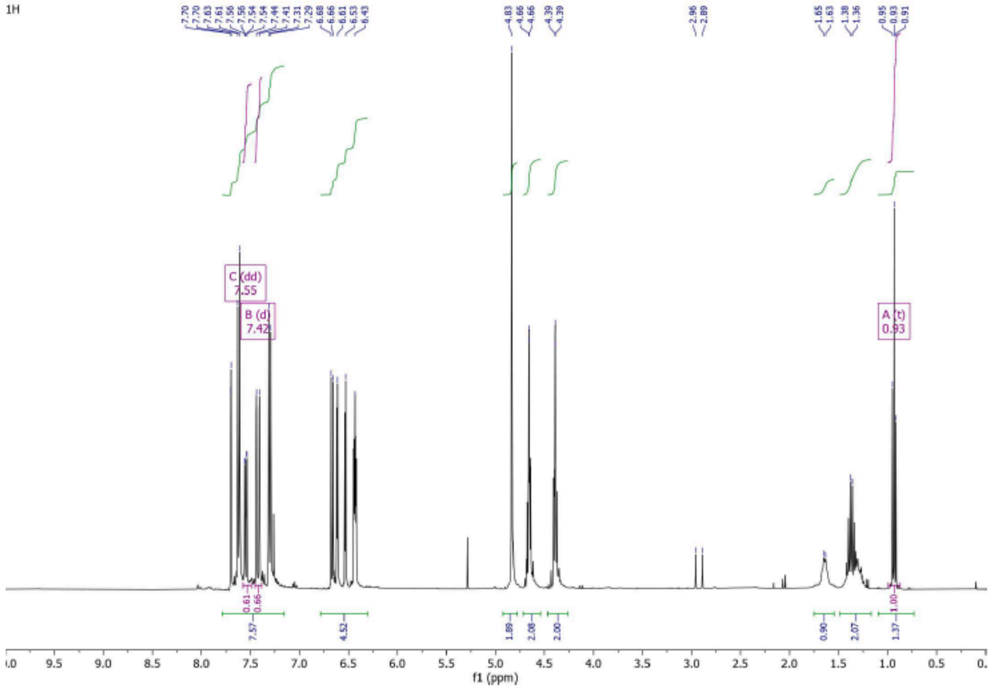

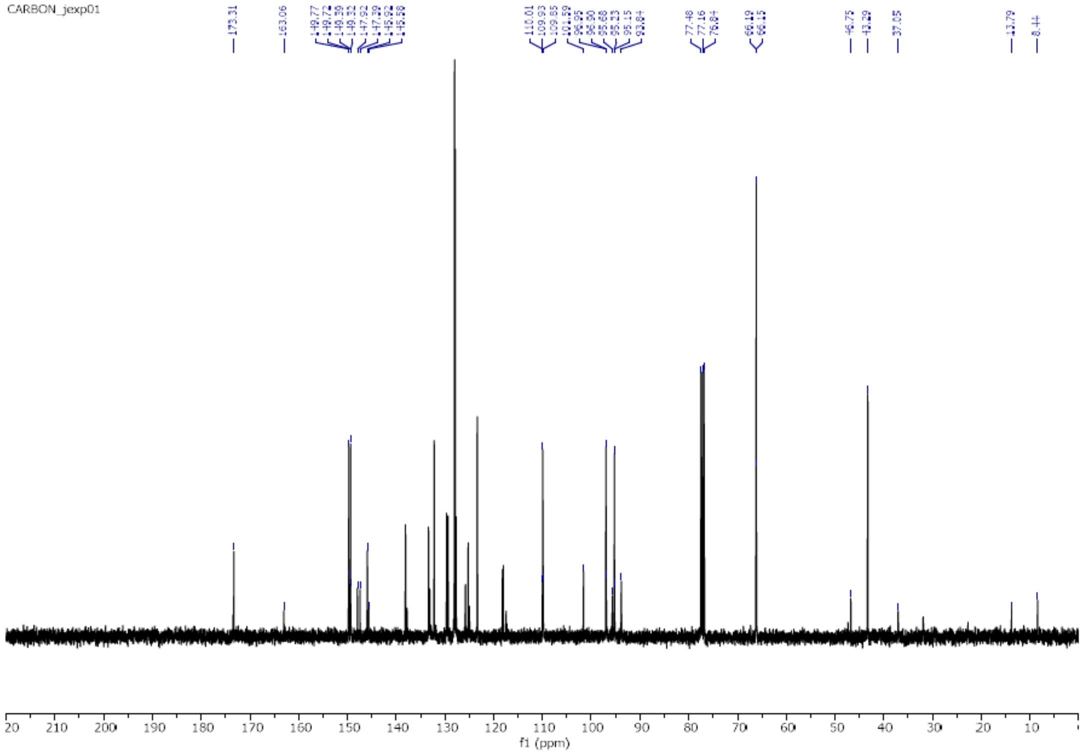

UNIDADE DE ESPECTROMETRÍA DE MASAS E PROTEÓMICA

AREA DE INFRAESTRUCTURAS DE INVESTIGACION
Edificio CACTUS * Campus Vida
15782 Santiago de Compostela
Tel. 881816242. Ext. 16242
E-mail: sxemasas@usc.es
Web:
https://www.usc.gal/es/servicios/unidades/unidad-espectrometria-masas-proteom

ESPECTRO APCI-FIA-Ion Trap

Analysis Info

Sample Name	11 APCI MS24-0272-9 383_10_01_2087.d	Acquisition Date	16/04/2024 19:14:45
Method	2087.m	Instrument	amaZon ETD

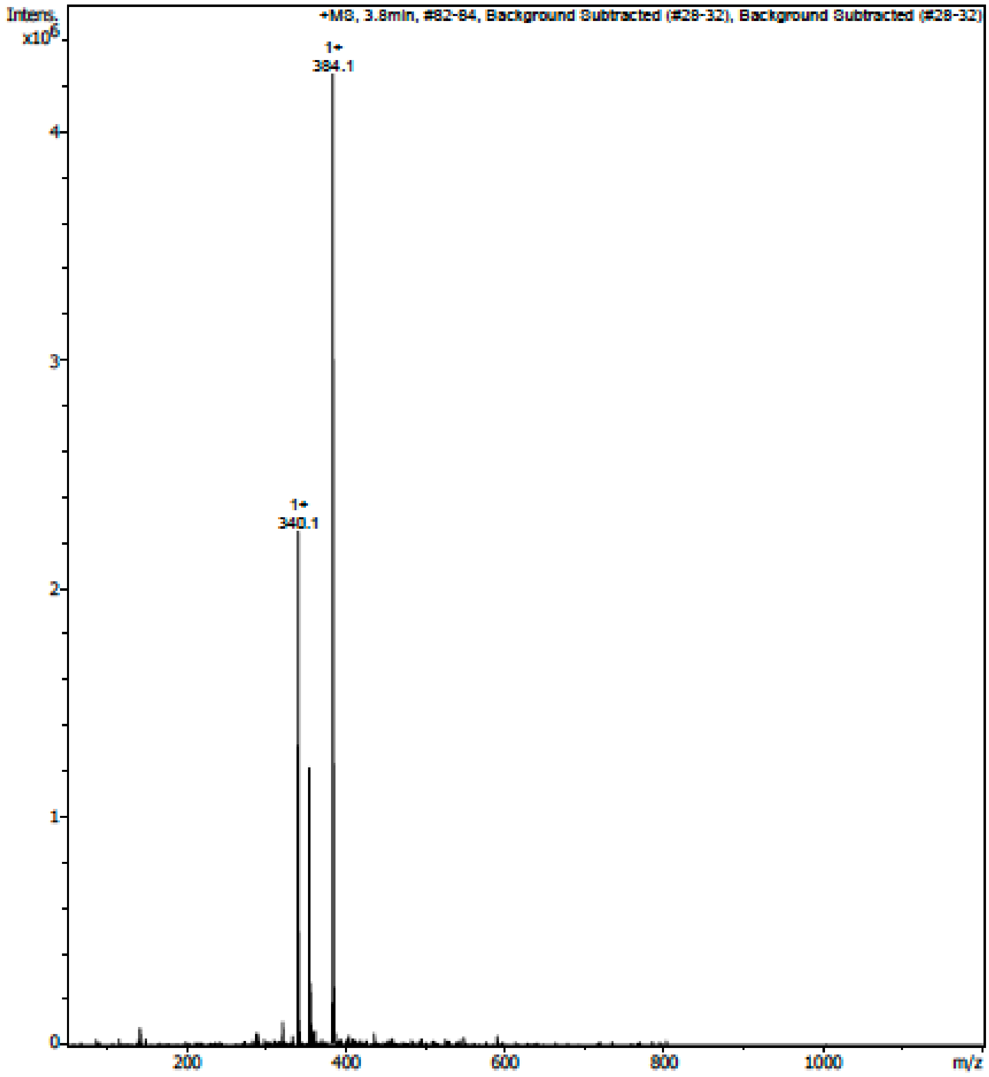

Medida De Masas Exactas

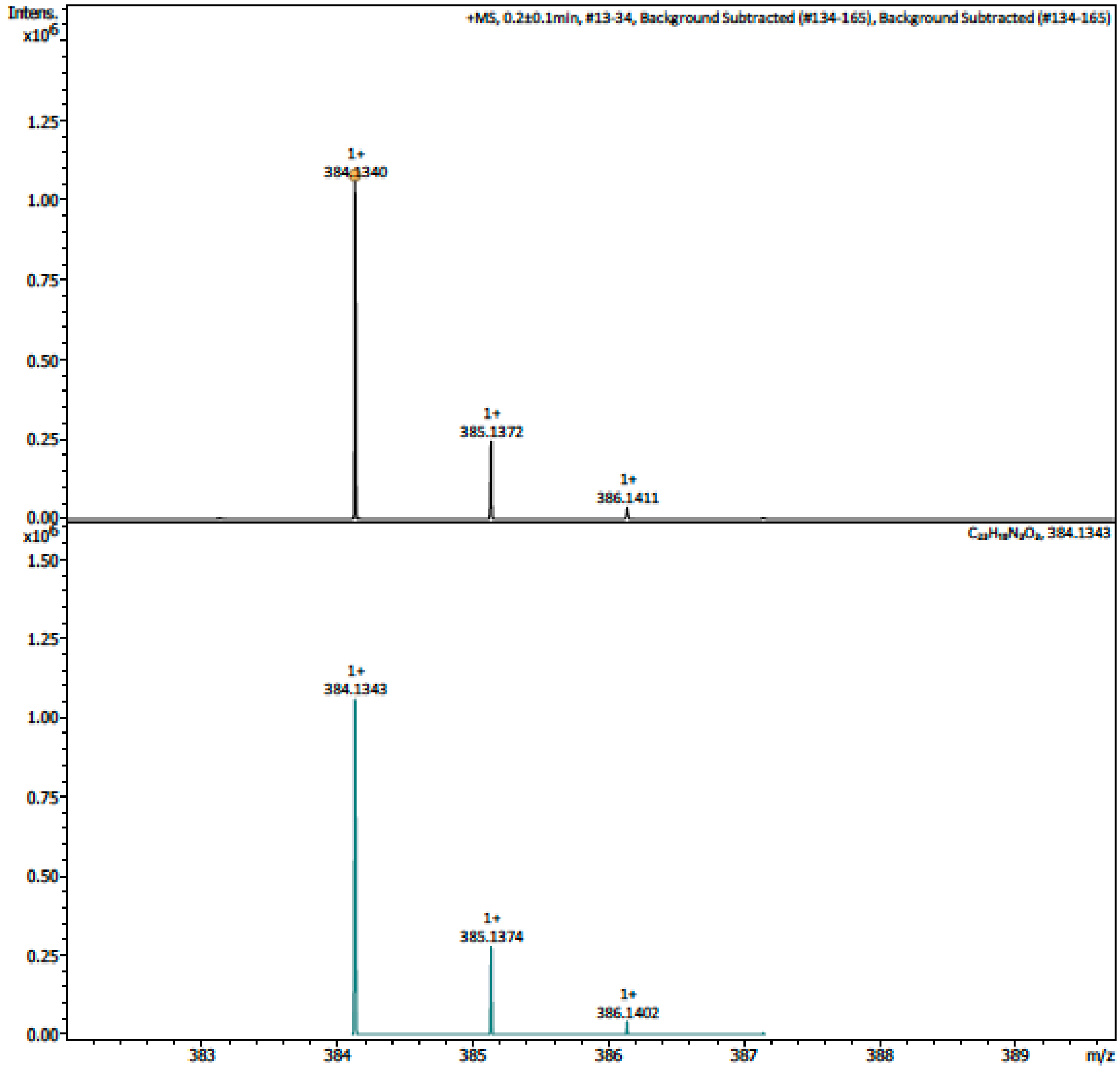

Meas. m/z	#	Ion Formula	Score	m/z	err [mDa]	err [ppm]	mSigma	rdb (neutral)	e⁻ Conf	N-Rule
384.1340	1	C21H16N6O2	79.07	384.1329	-1.0	-2.7	12.2	17.5	odd	ok
384.1340	2	C23H18N3O3	100.00	384.1343	0.3	0.8	18.8	17.0	even	ok

Compound 27:

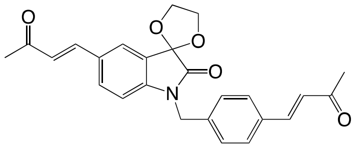

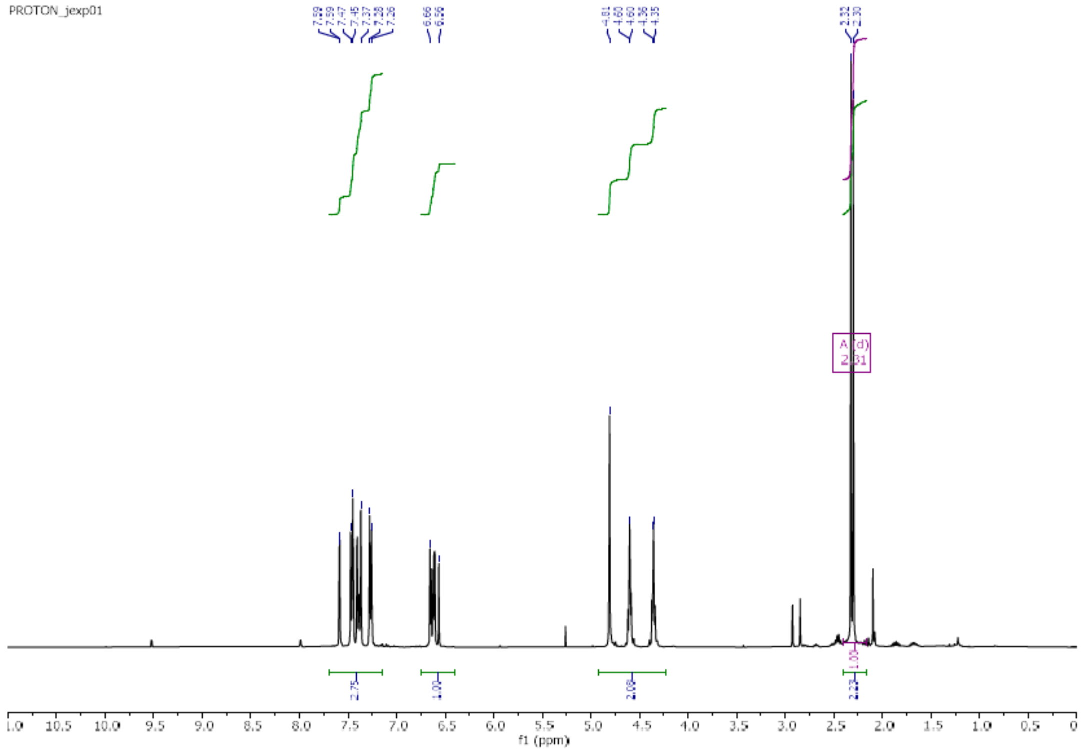

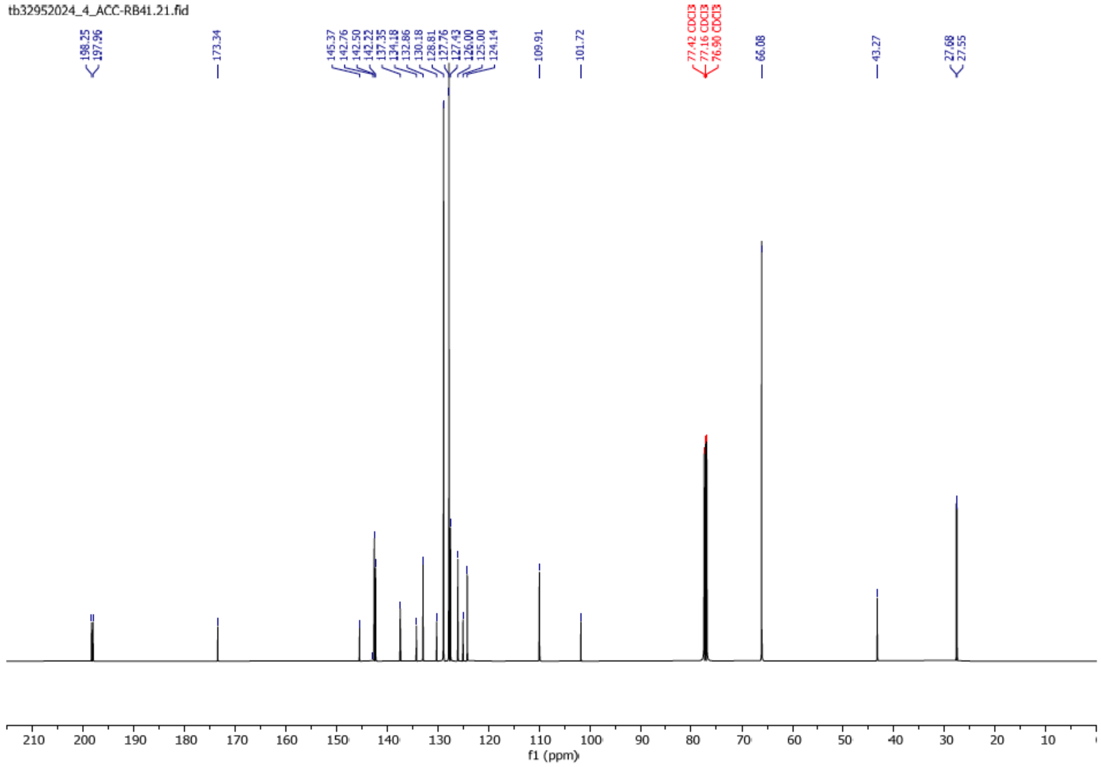

UNIDADE DE ESPECTROMETRÍA
DE MASAS E PROTEÓMICA

AREA DE INFRAESTRUCTURAS DE INVESTIGACION
Edificio CACTUS * Campus Vida
15782 Santiago de Compostela
Tel. 881816242 Ext. 16242
E-mail: sxemasas@usc.es
Web:
https://www.usc.gal/es/servicios/unidades/unidad-espectrometria-masas-proteom

ESPECTRO APCI-FIA-Ion Trap

Analysis Info

Sample Name	4 APCI MS-24-0509-1-fb3 417_2_01_2343.d	Acquisition Date	24/06/2024 11:42:23
Method	2343.m	Instrument	amaZon ETD

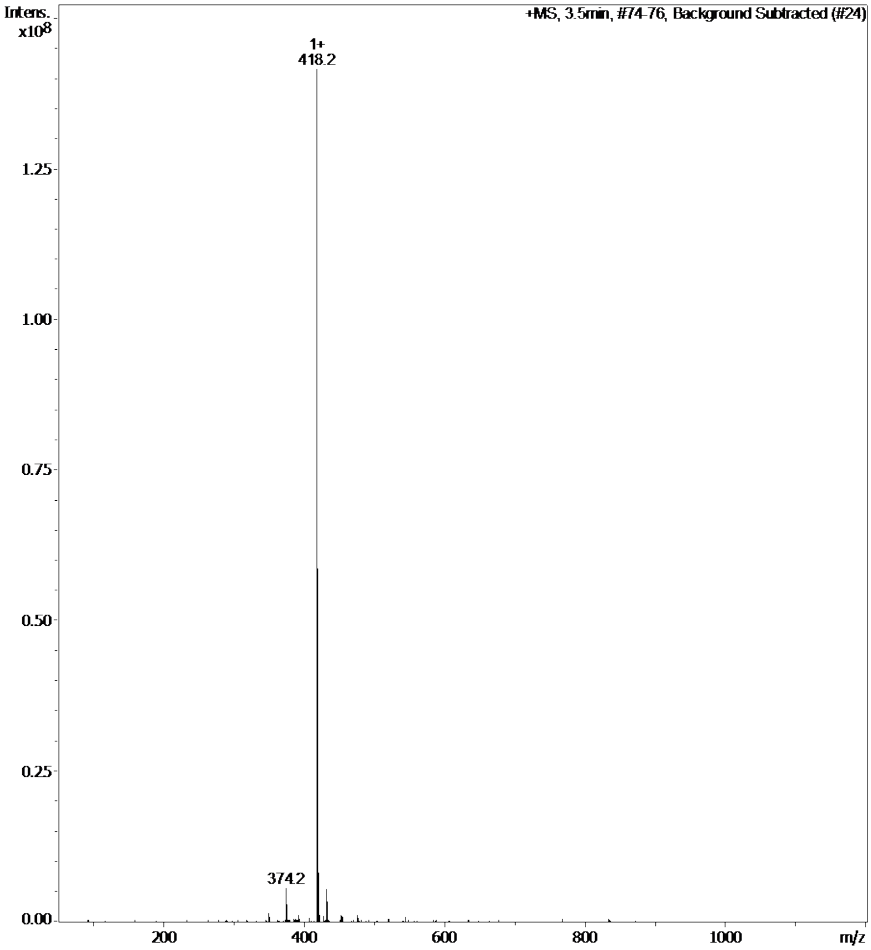

Medida De Masas Exactas

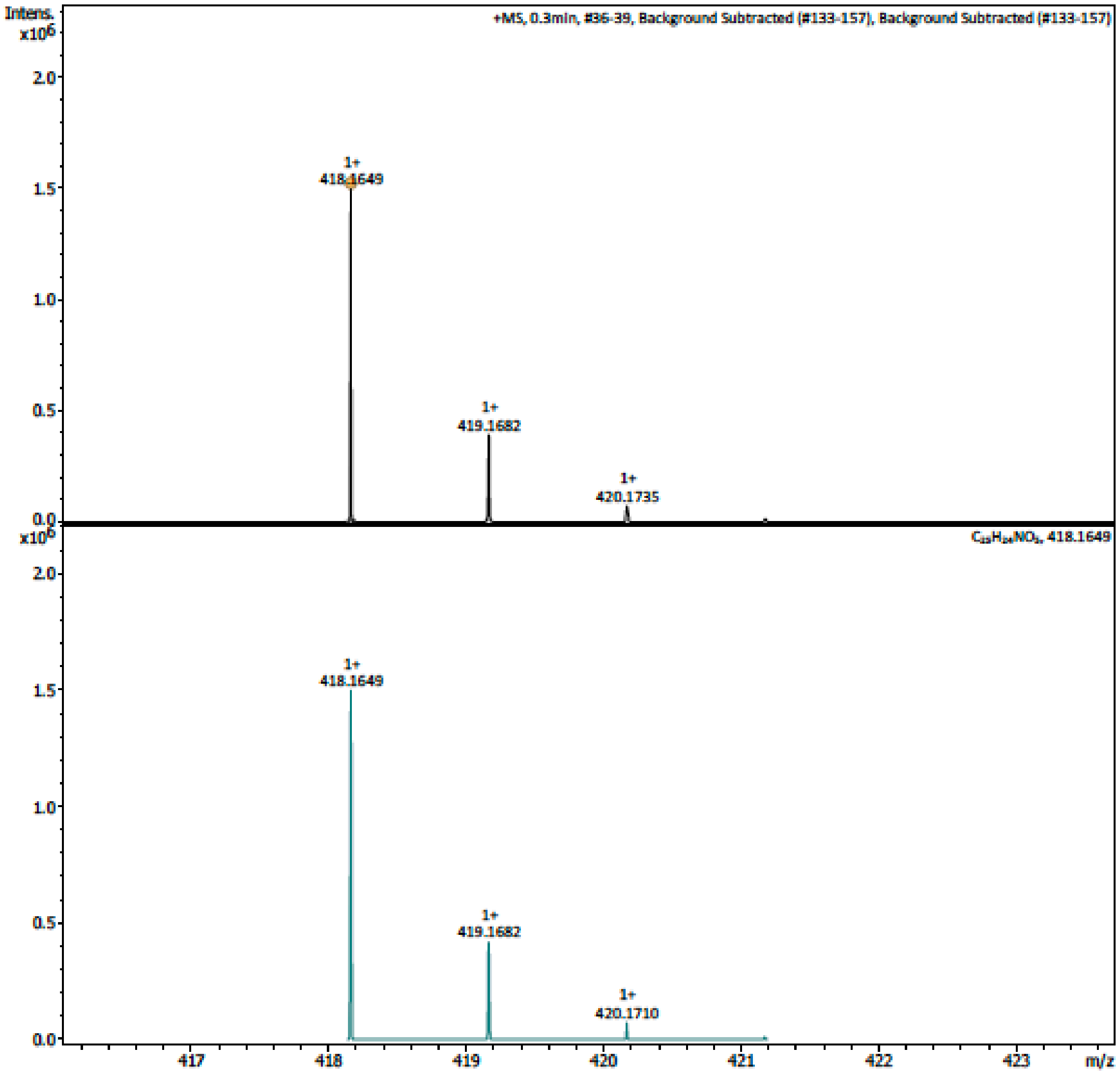

Meas. m/z	#	Ion Formula	Score	m/z	err [mDa]	err [ppm]	mSigma	rdb (neutral)	e⁻ Conf	N-Rule
418.1649	1	C23H22N4O4	40.50	418.1636	-1.4	-3.3	4.3	15.5	odd	ok
418.1649	2	C25H24NO5	100.00	418.1649	-0.0	-0.1	8.8	15.0	even	ok
418.1649	3	C24H18N8	63.30	418.1649	-0.0	-0.1	17.4	20.5	odd	ok
418.1649	4	C26H20N5O	28.87	418.1662	1.3	3.1	23.6	20.0	even	ok
418.1649	5	C9H14N20O	20.04	418.1654	0.5	1.1	54.2	13.5	odd	ok

Compound 28:

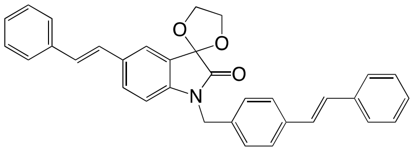

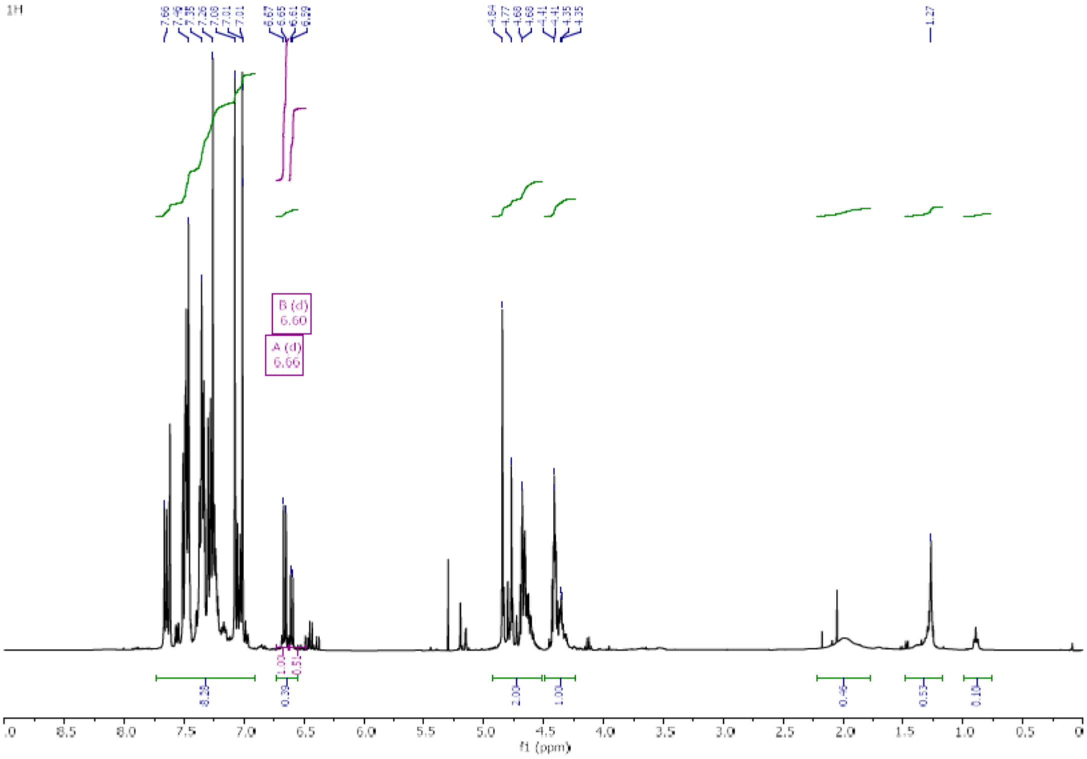

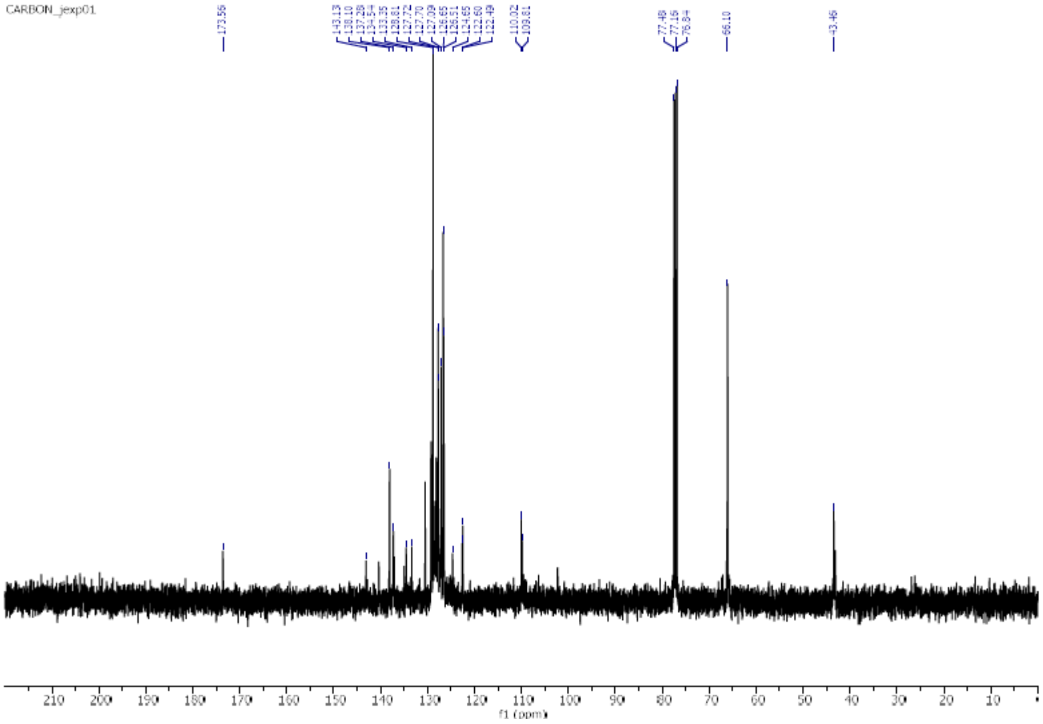

UNIDADE DE ESPECTROMETRÍA DE MASAS E PROTEÓMICA

AREA DE INFRAESTRUCTURAS DE INVESTIGACION
Edificio CACTUS * Campus Vida
15782 Santiago de Compostela
Tel. 881816242. Ext. 16242
E-mail: sxemasas@usc.es
Web:
https://www.usc.gal/es/servicios/unidades/unidad-espectrometria-masas-proteom

ESPECTRO APCI-FIA-Ion Trap

Analysis Info

Sample Name	7 APCI MS24-0272-5 485_6_01_2083.d	Acquisition Date	16/04/2024 18:30:43
Method	2083.m	Instrument	amaZon ETD

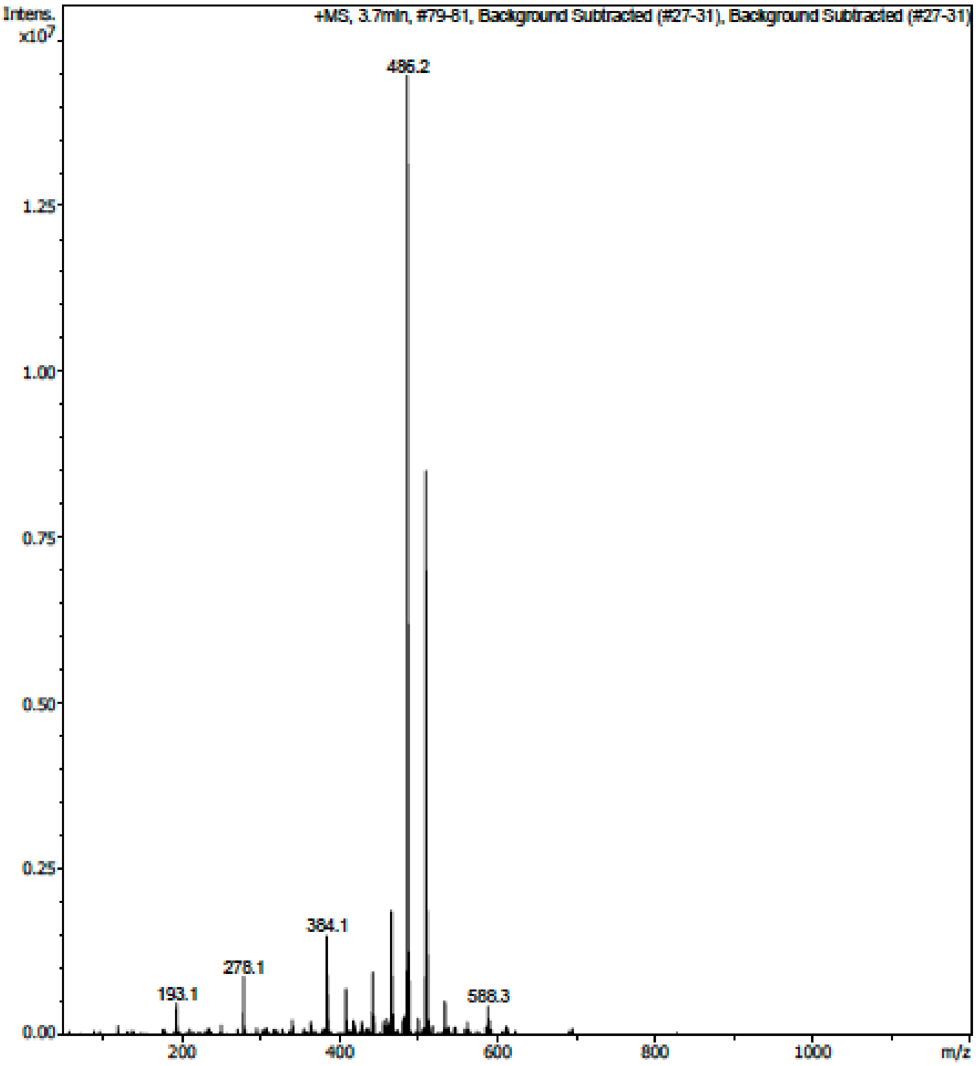

Medida De Masas Exactas

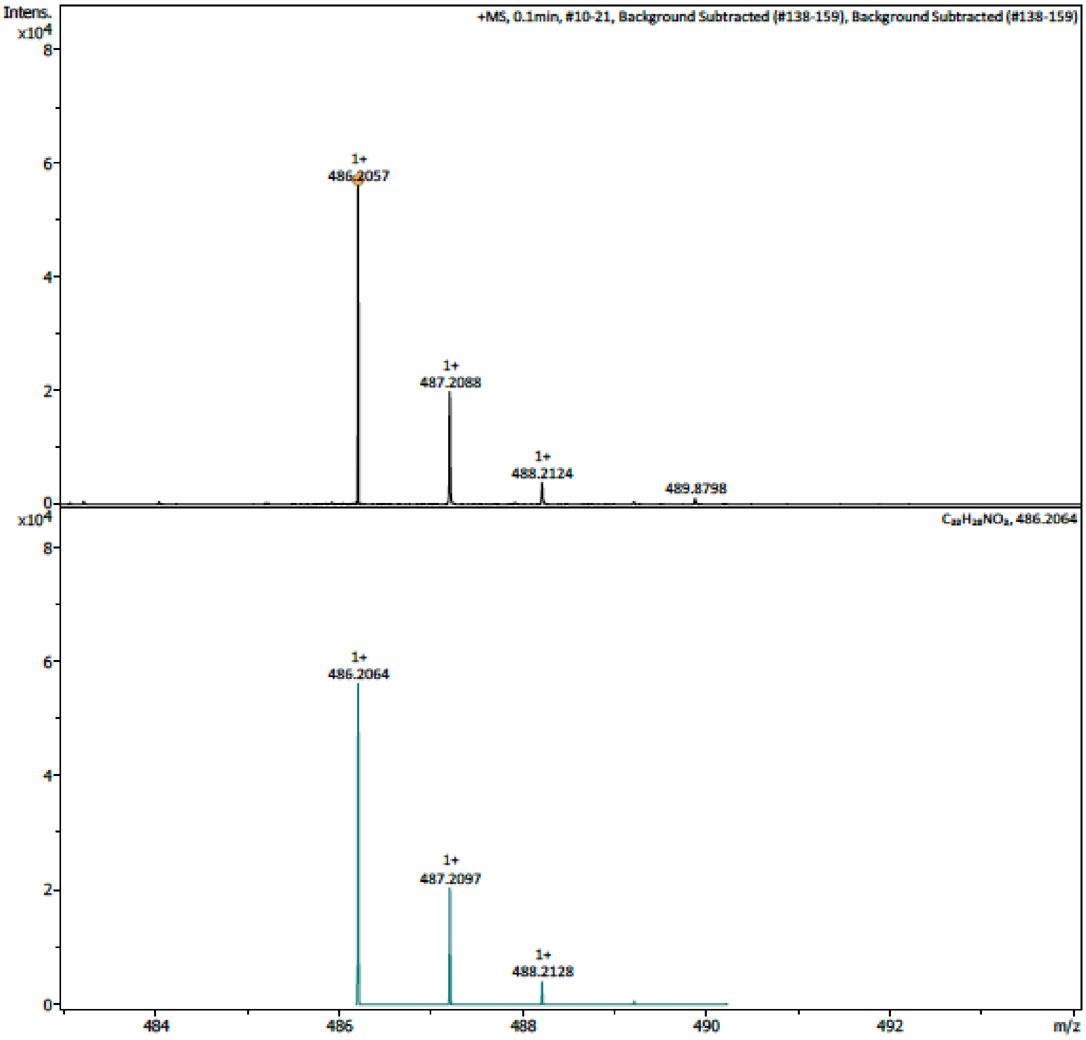

Meas. m/z	#	Ion Formula	Score	m/z	err [mDa]	err [ppm]	mSigma	rdb (neutral)	e¯ Conf	N-Rule
486.2057	1	C31H26N4O2	100.00	486.2050	-0.7	-1.5	1.8	21.5	odd	ok
486.2057	2	C33H28NO3	97.85	486.2064	0.6	1.3	5.9	21.0	even	ok
486.2057	3	C19H30N6O9	18.31	486.2069	1.1	2.3	60.8	8.5	odd	ok
486.2057	4	C18H24N13O4	12.17	486.2069	1.1	2.3	64.4	14.0	even	ok
486.2057	5	C16H22N16O3	15.07	486.2055	-0.2	-0.4	71.4	14.5	odd	ok

Compound 29:

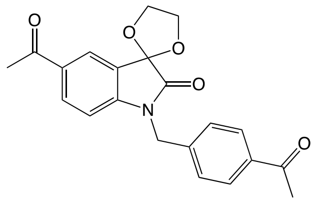

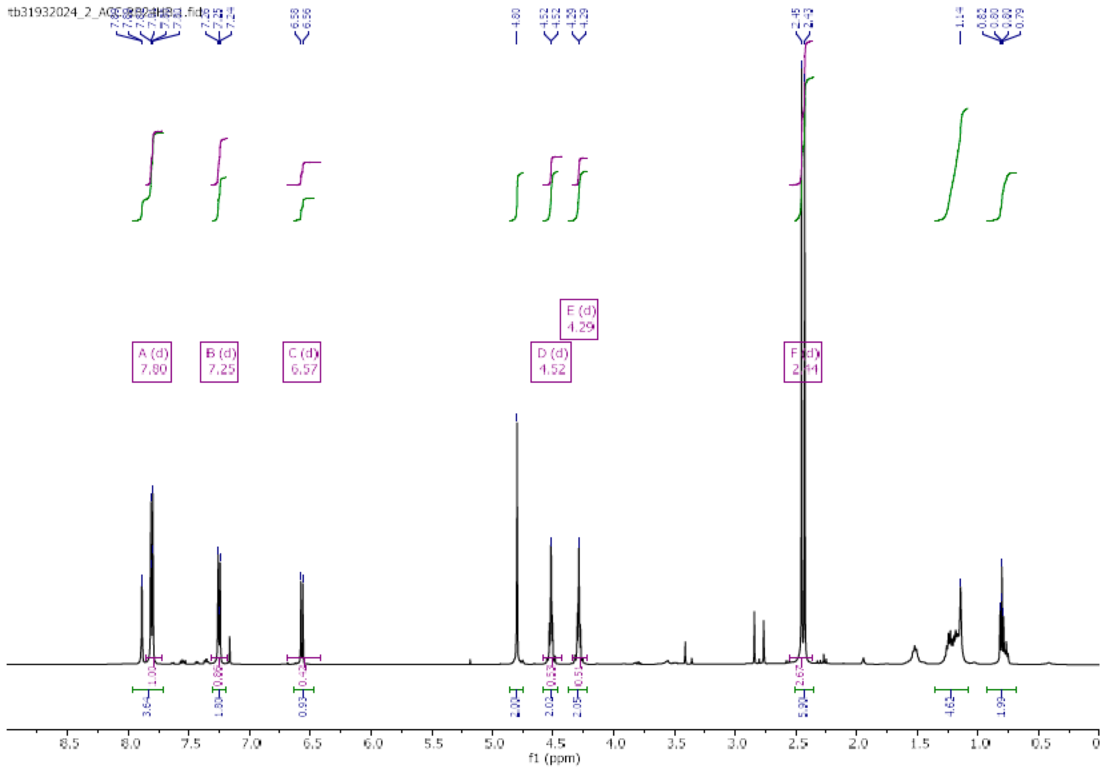

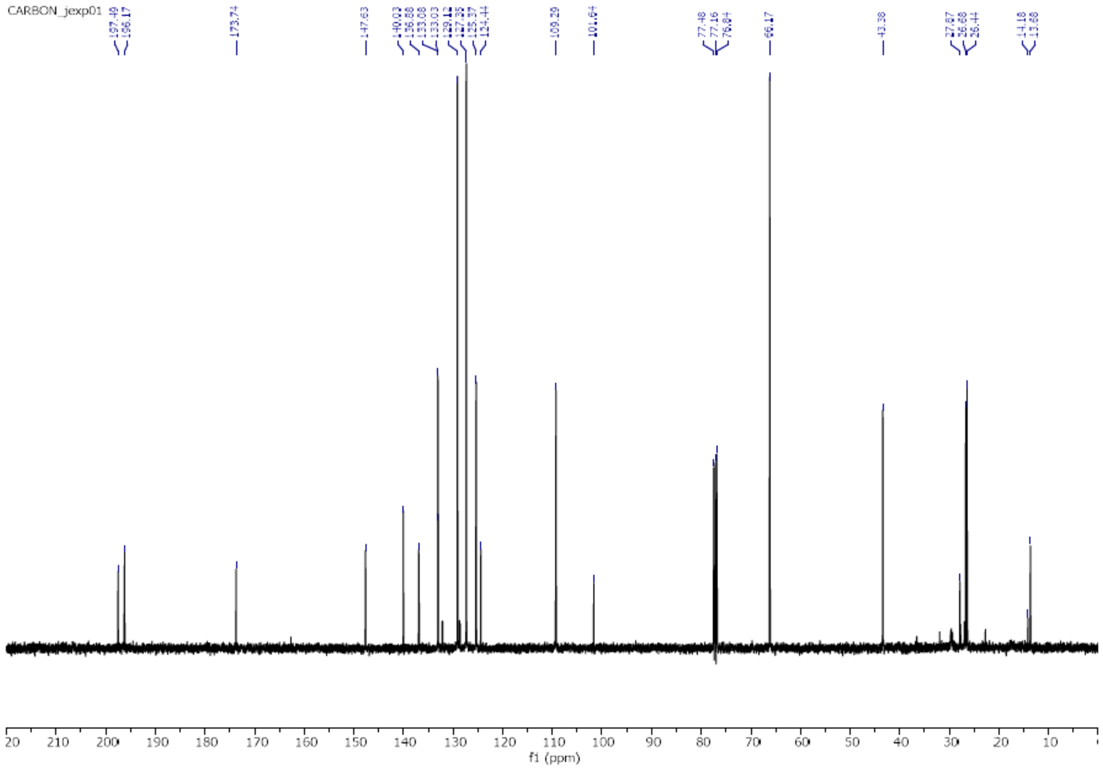

ESPECTRO APCI-FIA-Ion Trap

Analysis Info

Sample Name	8 APCI MS24-0266-6 365_6_01_2067.d	Acquisition Date	15/04/2024 12:04:06
Method	2067.m	Instrument	amaZon ETD

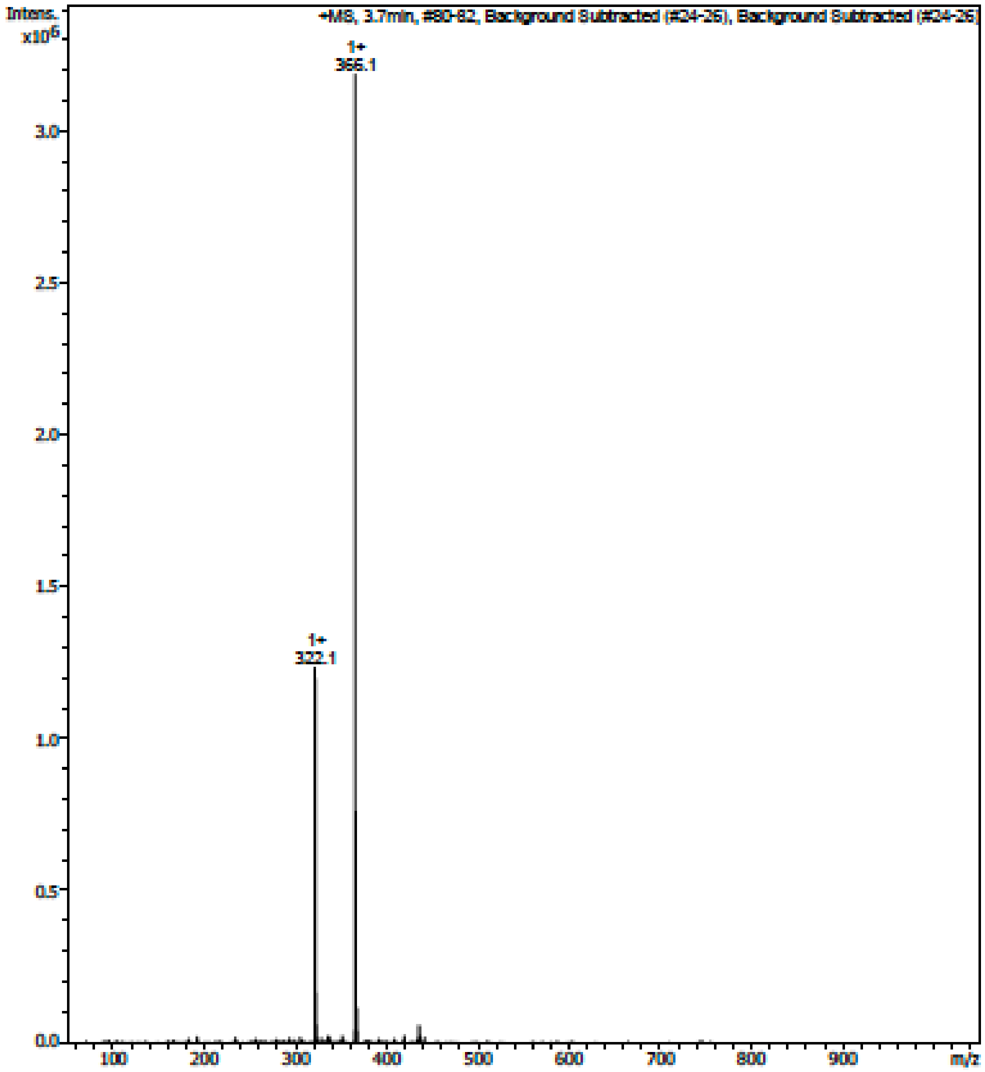

Medida De Masas Exactas

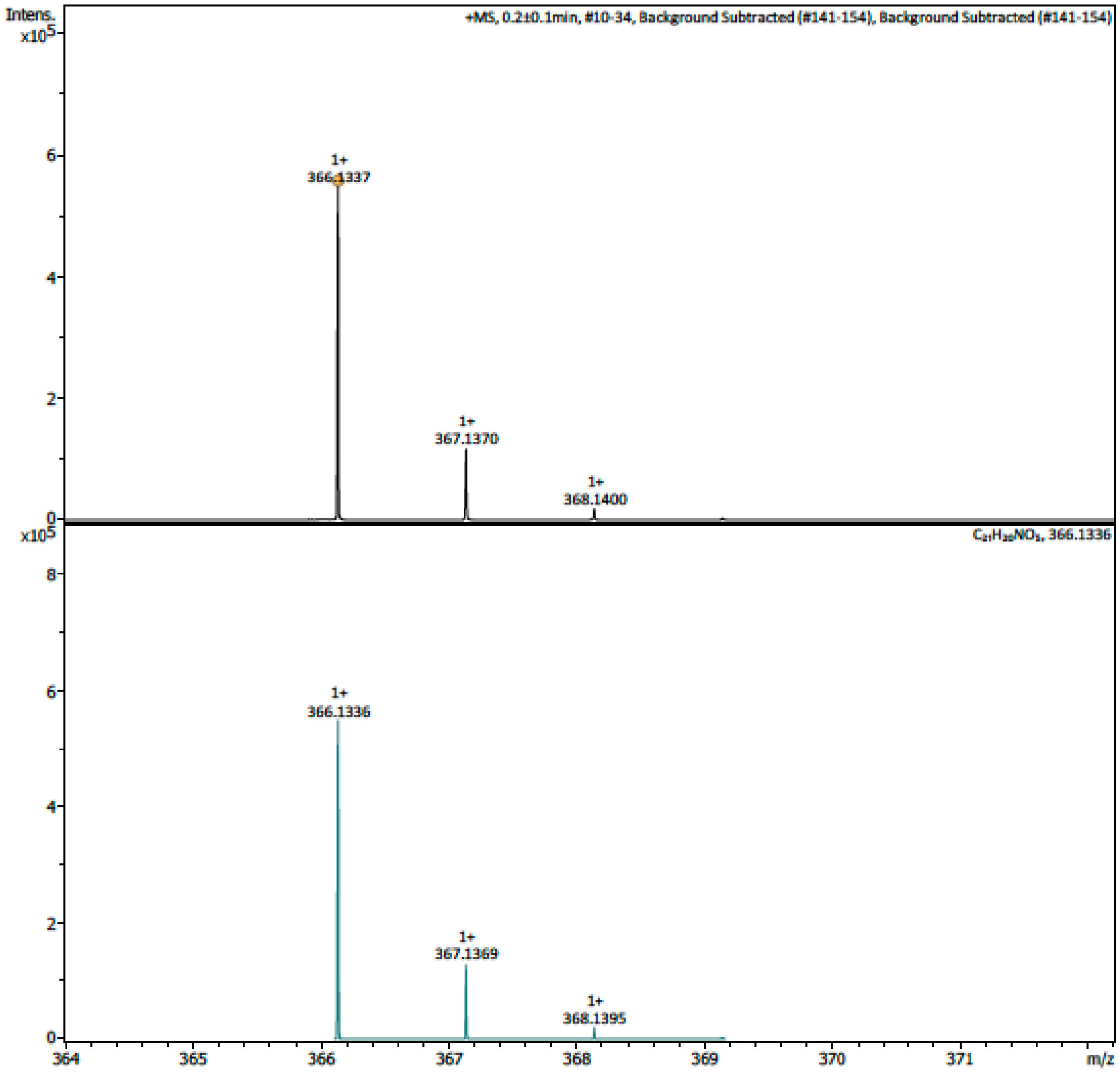

Meas. m/z	#	Ion Formula	Score	m/z	err [mDa]	err [ppm]	mSigma	rdb (neutral)	e⁻ Conf	N-Rule
366.1337	1	$C_{19}H_{18}N_4O_4$	55.27	366.1323	-1.5	-4.0	6.6	13.5	odd	ok
366.1337	2	$C_{21}H_{20}NO_5$	100.00	366.1336	-0.1	-0.4	13.4	13.0	even	ok
366.1337	3	$C_{20}H_{14}N_8$	86.95	366.1336	-0.1	-0.4	20.1	18.5	odd	ok
366.1337	4	$C_{22}H_{16}N_5O$	43.38	366.1349	1.2	3.3	26.7	18.0	even	ok
366.1337	5	$C_5H_{10}N_{20}O$	36.39	366.1341	0.4	1.0	49.9	11.5	odd	ok

Compound 30:

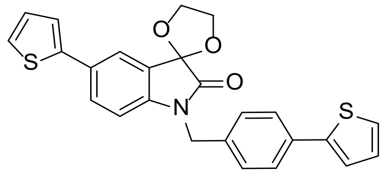

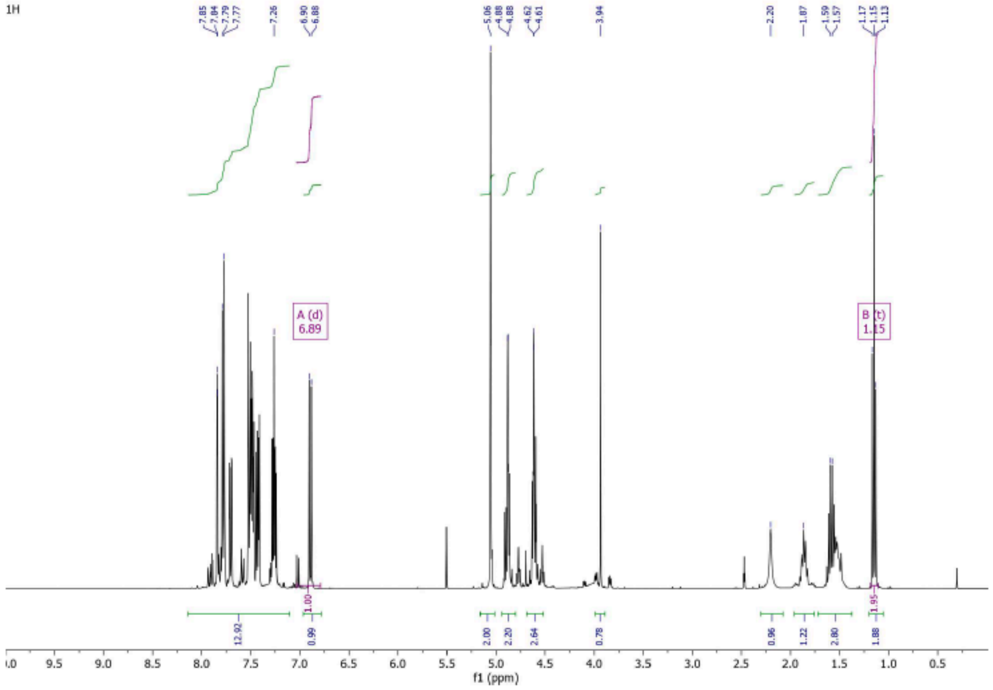

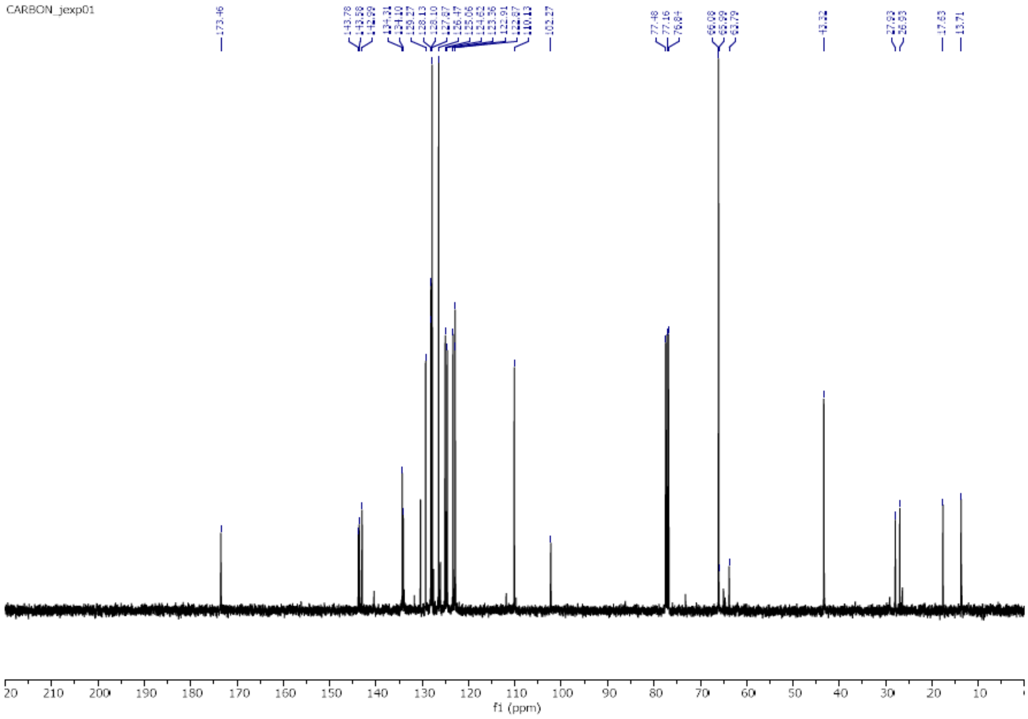

ESPECTRO APCI-FIA-Ion Trap

Analysis Info

Sample Name	5 APCI MS24-0266-4 445_4_01_2064.d	Acquisition Date	15/04/2024 11:31:28
Method	2064.m	Instrument	amaZon ETD

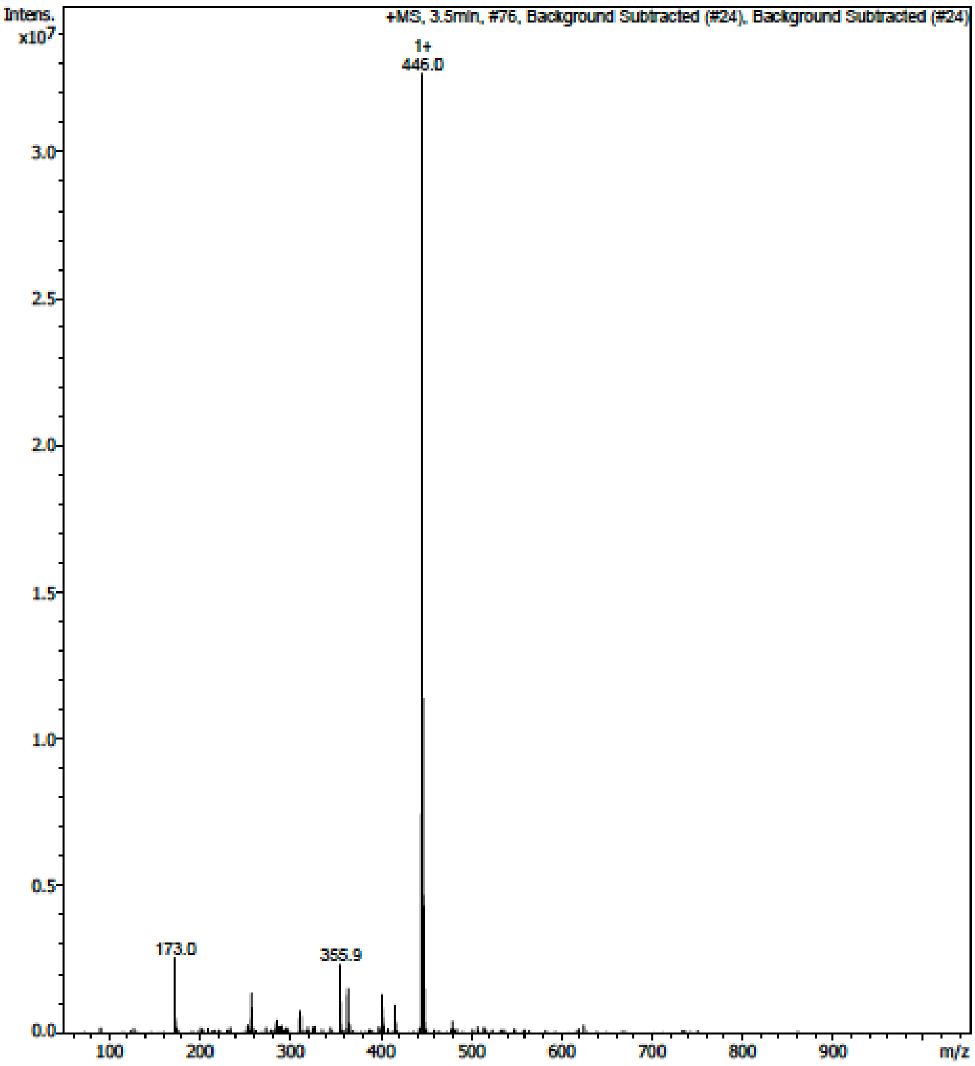

Medida De Masas Exactas

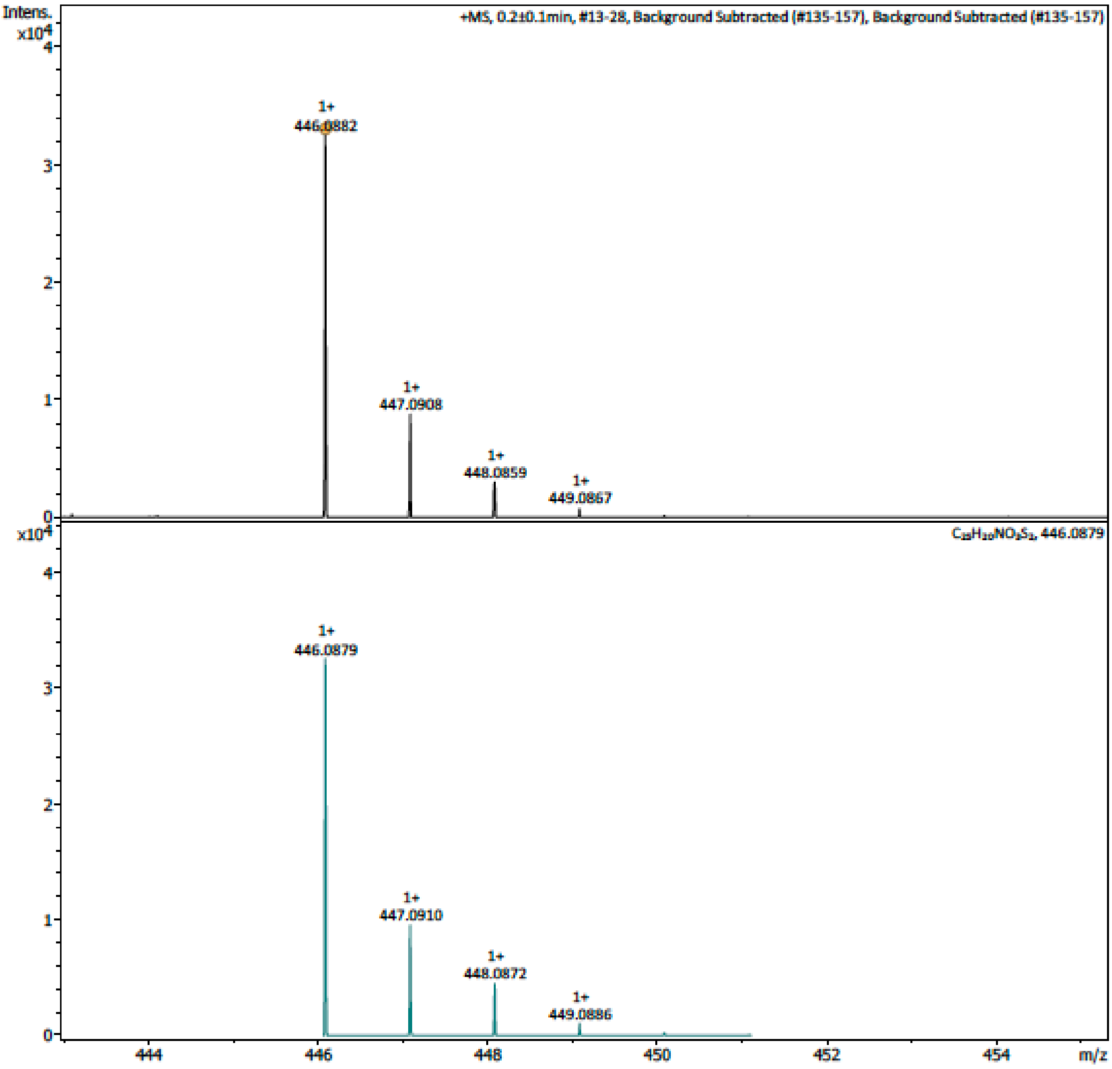

Meas. m/z	#	Ion Formula	Score	m/z	err [mDa]	err [ppm]	mSigma	rdb (neutral)	e⁻ Conf	N-Rule
446.0882	1	C18H12N11O2S	73.30	446.0891	0.8	1.9	15.1	21.0	even	ok
446.0882	2	C19H18N4O7S	67.20	446.0891	0.8	1.9	19.2	15.5	odd	ok
446.0882	3	C24H16NO8	52.88	446.0870	-1.2	-2.7	21.0	18.0	even	ok
446.0882	4	C16H10N14OS	76.34	446.0877	-0.5	-1.1	21.1	21.5	odd	ok
446.0882	5	C25H20NO3S2	100.00	446.0879	-0.3	-0.7	23.0	21.0	even	ok

Compound 31:

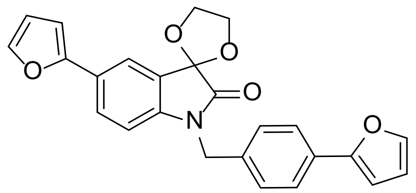

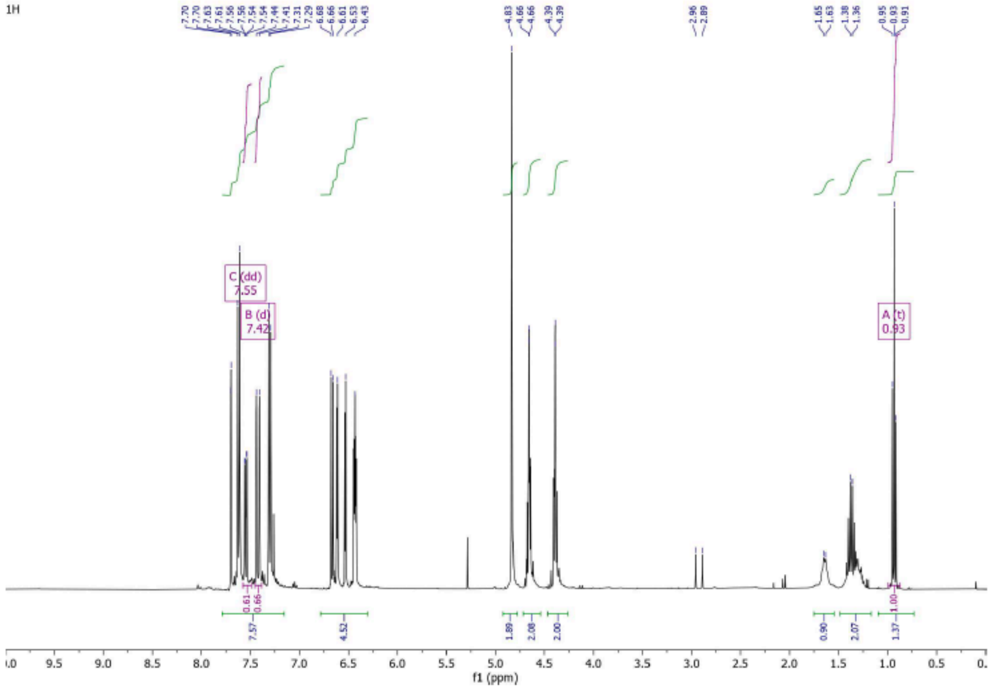

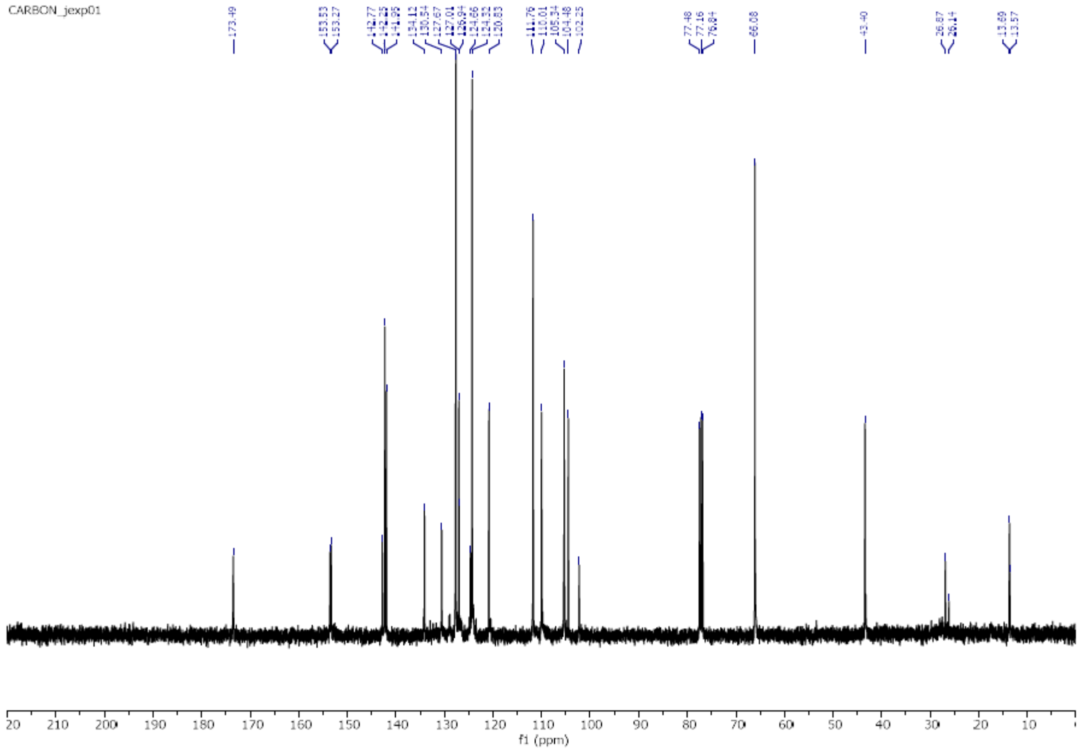

AREA DE INFRAESTRUCTURAS DE INVESTIGACION
UNIDADE DE ESPECTROMETRIA DE MASAS E PROTEÓMICA
Edificio CACTUS * Campus Vida
15782 Santiago de Compostela
Tel. 881816242. Ext. 16242
E-mail: sxemasas@usc.es
https://www.usc.gal/gl/servizos/area/infraestruturas-investigacion/servizos-equipamentos/espectrometria-masas-proteomica

ESPECTRO APCI-FIA-Ion Trap

Analysis Info

Sample Name	7 APCI MS24-0266-5 413_5_01_2066.d	Acquisition Date	15/04/2024 11:53:05
Method	2066.m	Instrument	amaZon ETD

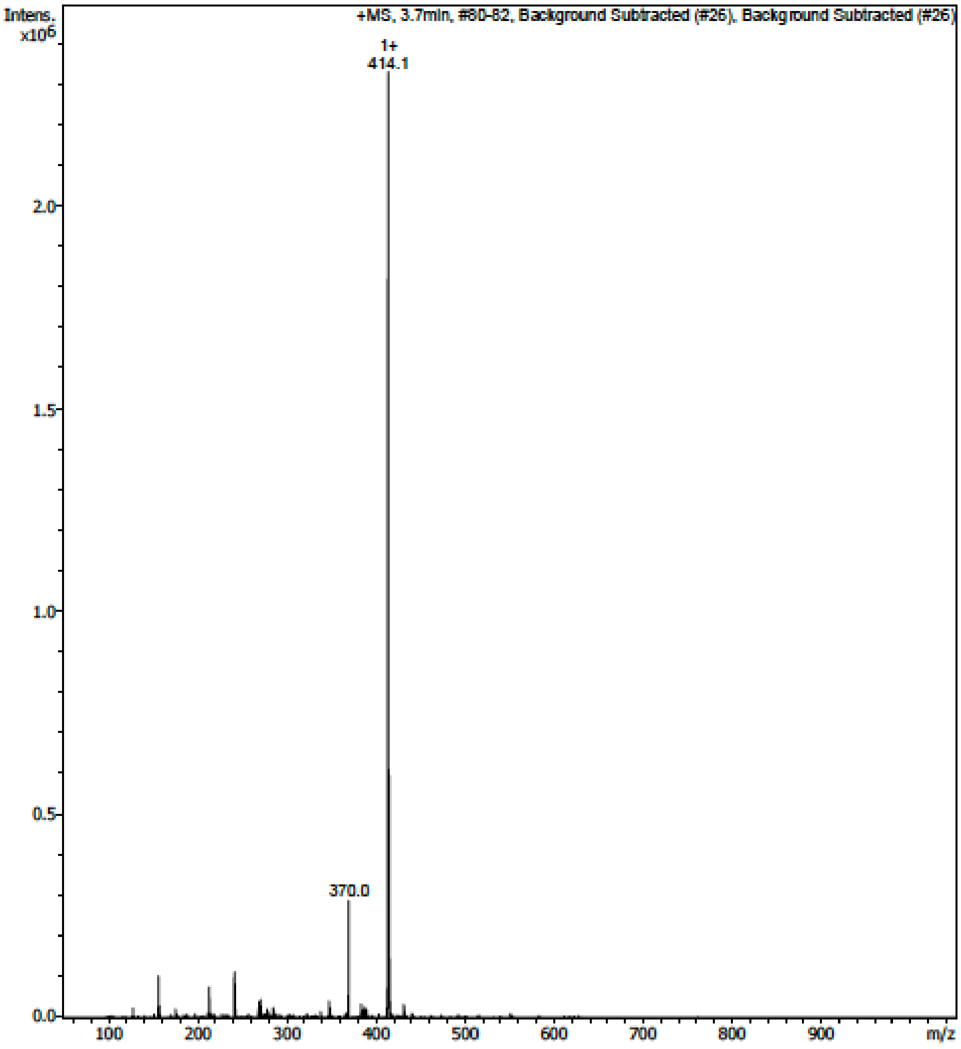

Medida De Masas Exactas

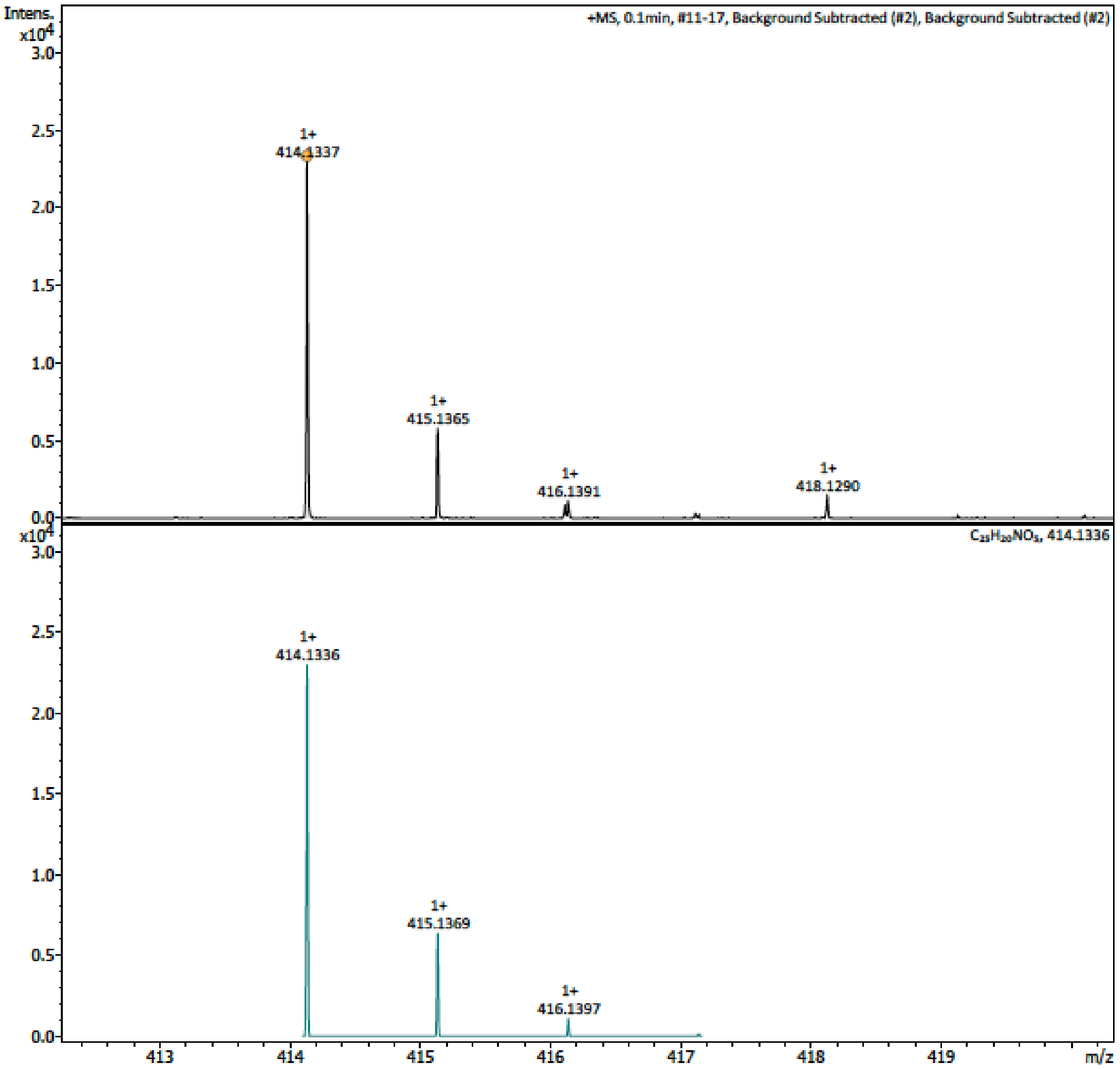

Meas. m/z	#	Ion Formula	Score	m/z	err [mDa]	err [ppm]	mSigma	rdb (neutral)	e⁻ Conf	N-Rule
414.1337	1	C25H20NO5	100.00	414.1336	-0.1	-0.2	13.5	17.0	even	ok

Compound 32:

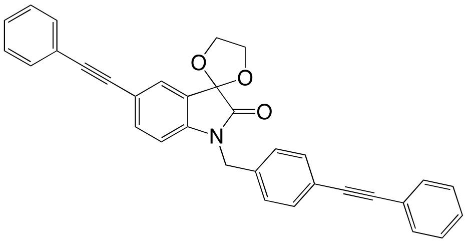

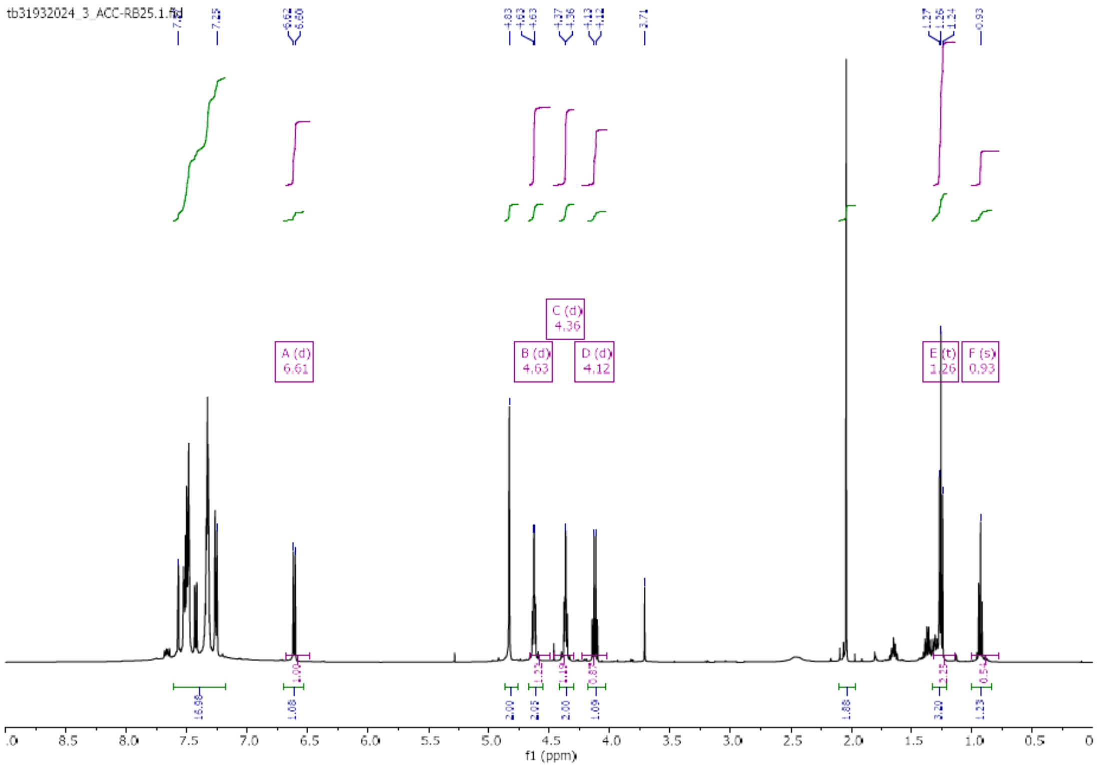

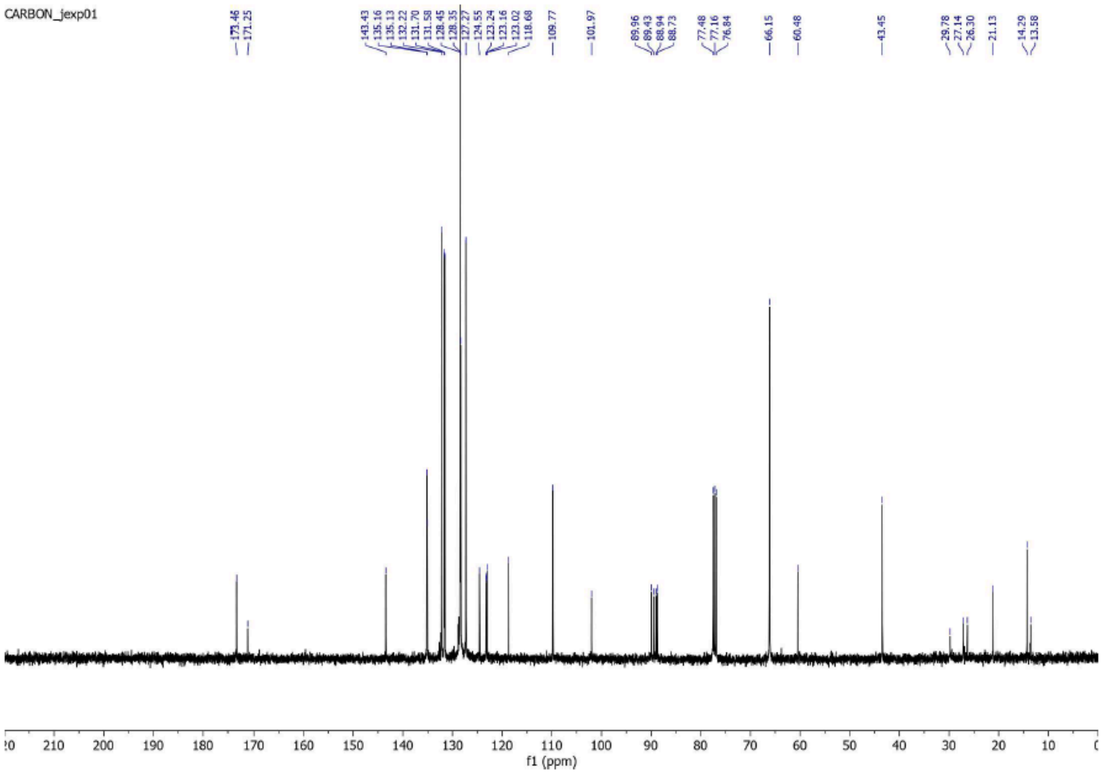

AREA DE INFRAESTRUCTURAS DE INVESTIGACION
UNIDADE DE ESPECTROMETRIA DE MASAS E PROTEÓMICA
Edificio CACTUS * Campus Vida
15782 Santiago de Compostela
Tel. 881816242. Ext. 16242
E-mail: sxemasas@usc.es
https://www.usc.gal/gl/servizos/area/infraestruturas-investigacion/servizos-equipamentos/espectrometria-masas-proteomica

ESPECTRO APCI-FIA-Ion Trap

Analysis Info

Sample Name	4 APCI MS24-0266-8 481_3_01_2058.d	Acquisition Date	12/04/2024 11:56:00
Method	2058.m	Instrument	amaZon ETD

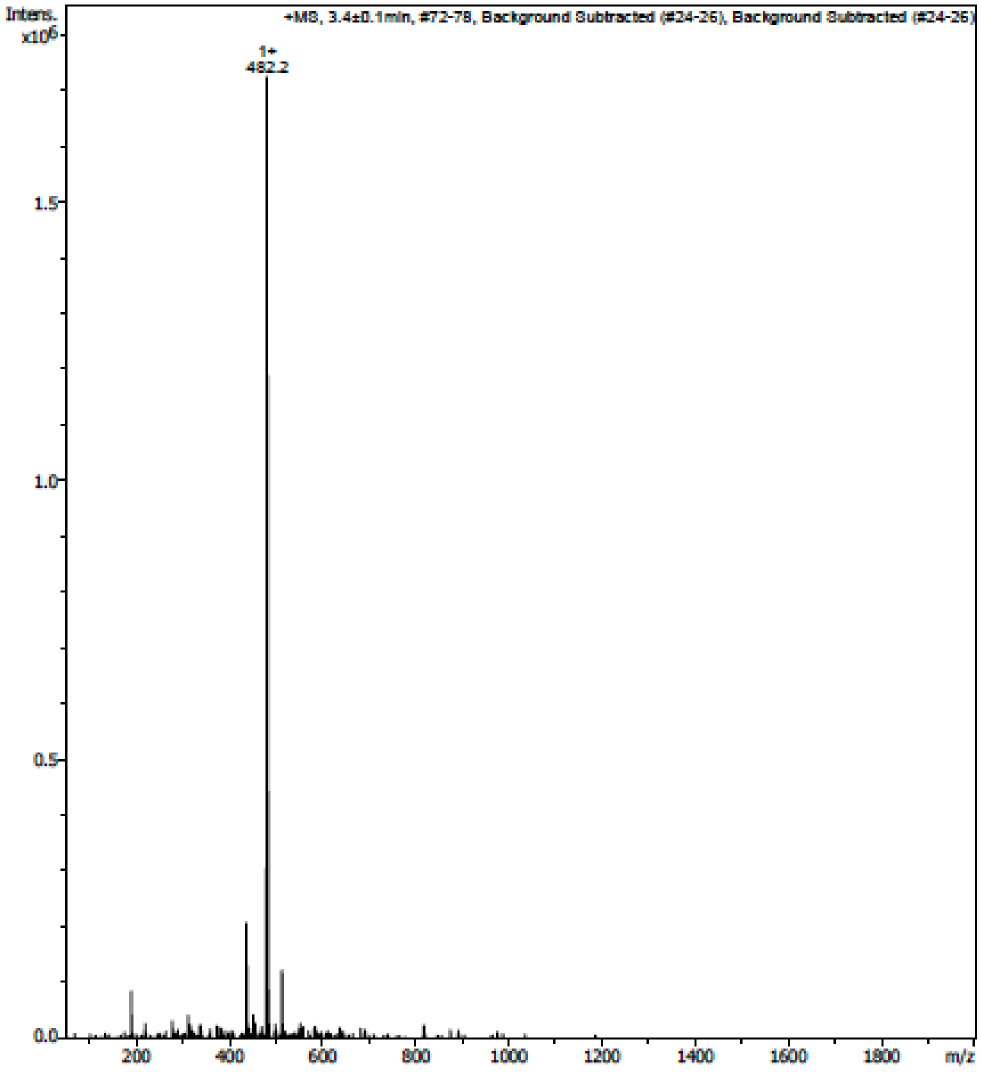

Medida De Masas Exactas

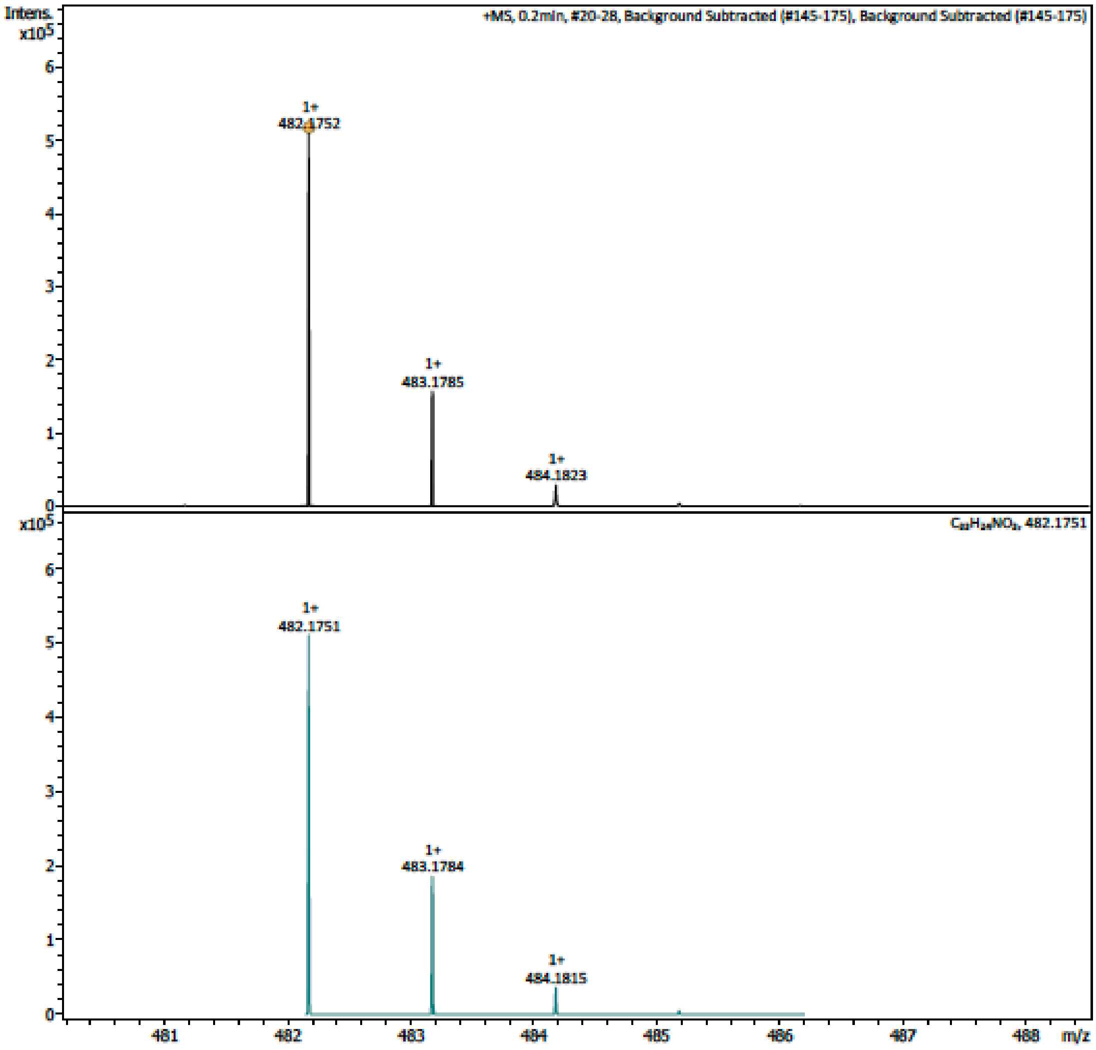

Meas. m/z	#	Ion Formula	Score	m/z	err [mDa]	err [ppm]	mSigma	rdb (neutral)	e¯ Conf	N-Rule
482.1752	1	C31H22N4O2	55.92	482.1737	-1.5	-3.1	22.7	23.5	odd	ok
482.1752	2	C19H16N17	33.36	482.1769	1.7	3.5	26.1	21.0	even	ok
482.1752	3	C33H24NO3	100.00	482.1751	-0.1	-0.3	28.8	23.0	even	ok
482.1752	4	C20H22N10O5	29.64	482.1769	1.7	3.5	30.9	15.5	odd	ok
482.1752	5	C21H28N3O10	38.38	482.1769	1.7	3.6	31.9	10.0	even	ok

Compound 33:

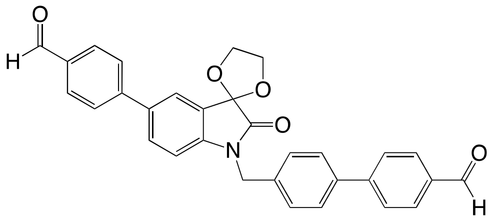

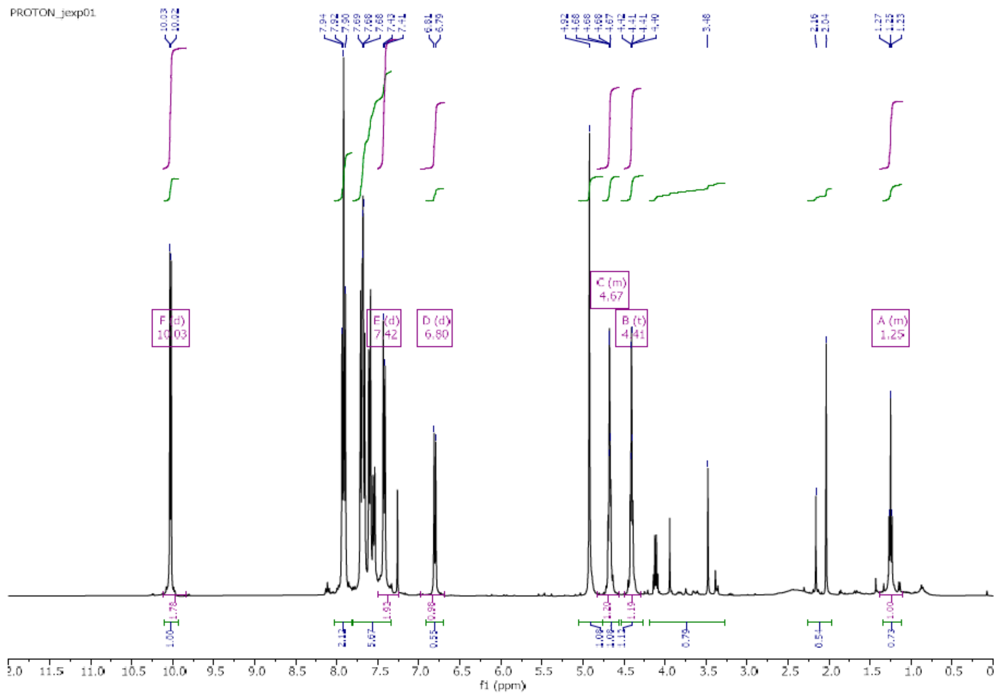

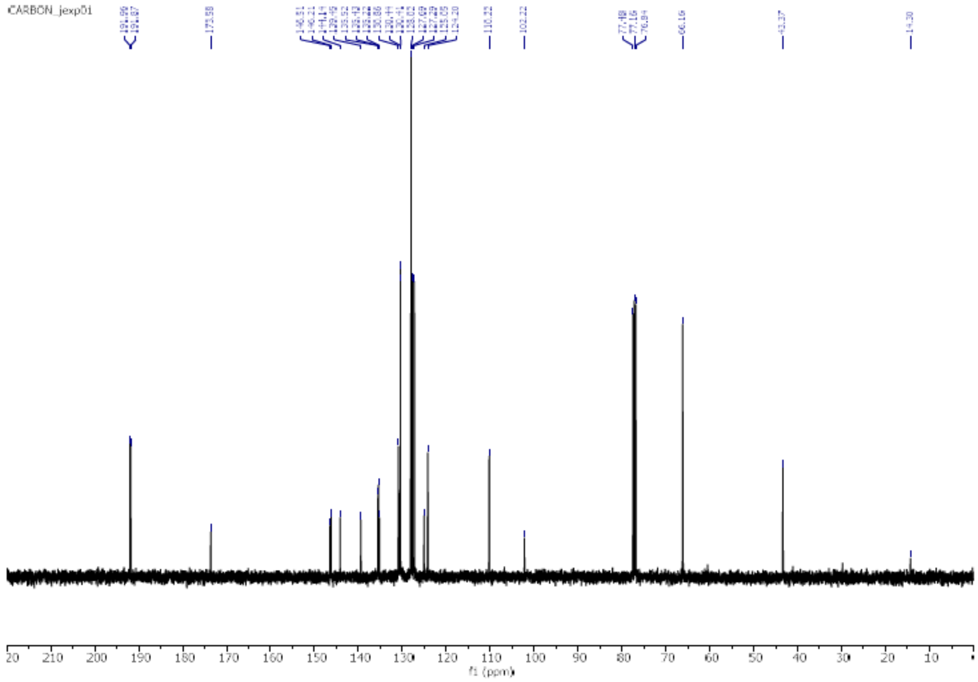

UNIDADE DE ESPECTROMETRÍA
DE MASAS E PROTEÓMICA

AREA DE INFRAESTRUCTURAS DE INVESTIGACION
Edificio CACTUS * Campus Vida
15782 Santiago de Compostela
Tel. 881816242. Ext. 16242
E-mail: sxemasas@usc.es
Web:
https://www.usc.gal/es/servicios/unidades/unidad-espectrometria-masas-proteom

ESPECTRO APCI-FIA-Ion Trap

Analysis Info

Sample Name	8 APCI MS-24-0378-6-tb3 489_7_01_2211.d	Acquisition Date	16/05/2024 11:48:14
Method	2211.m	Instrument	amaZon ETD

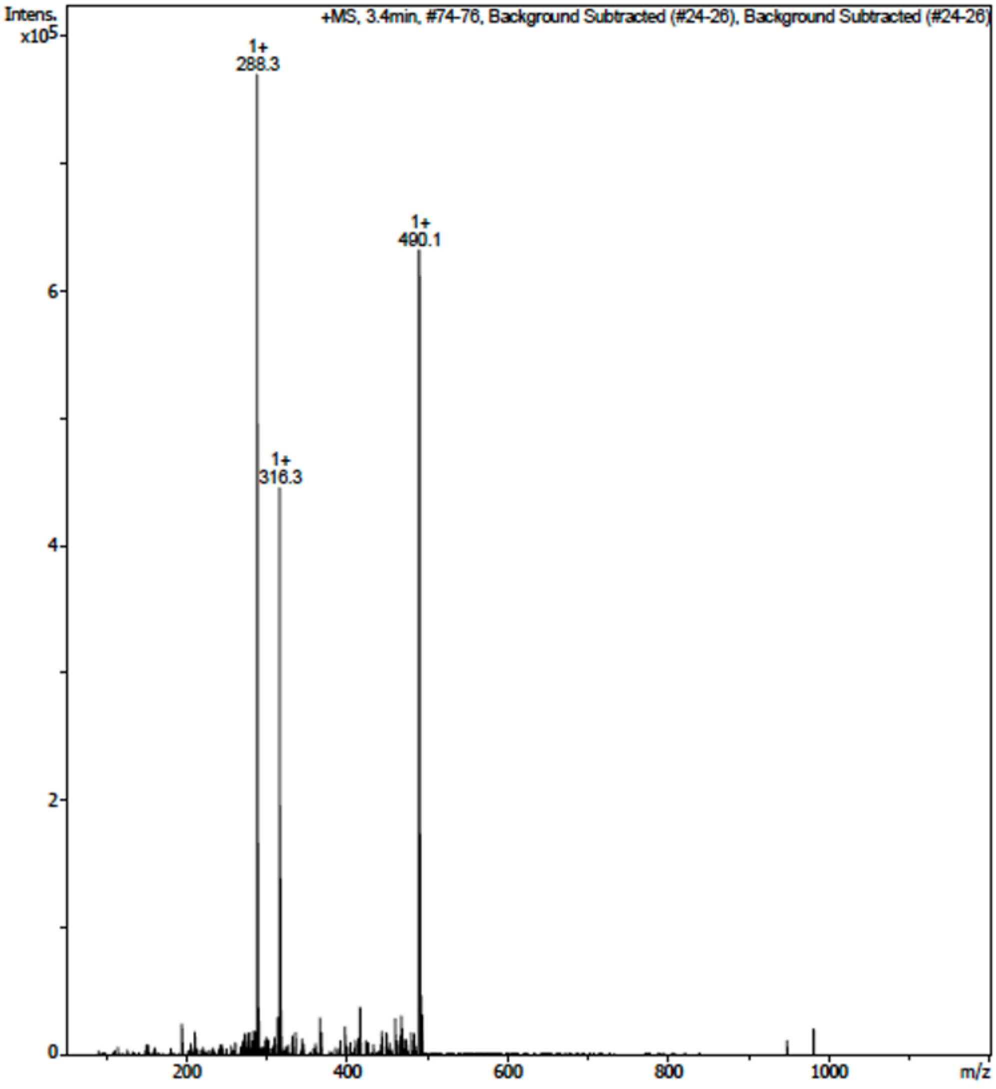

Medida De Masas Exactas

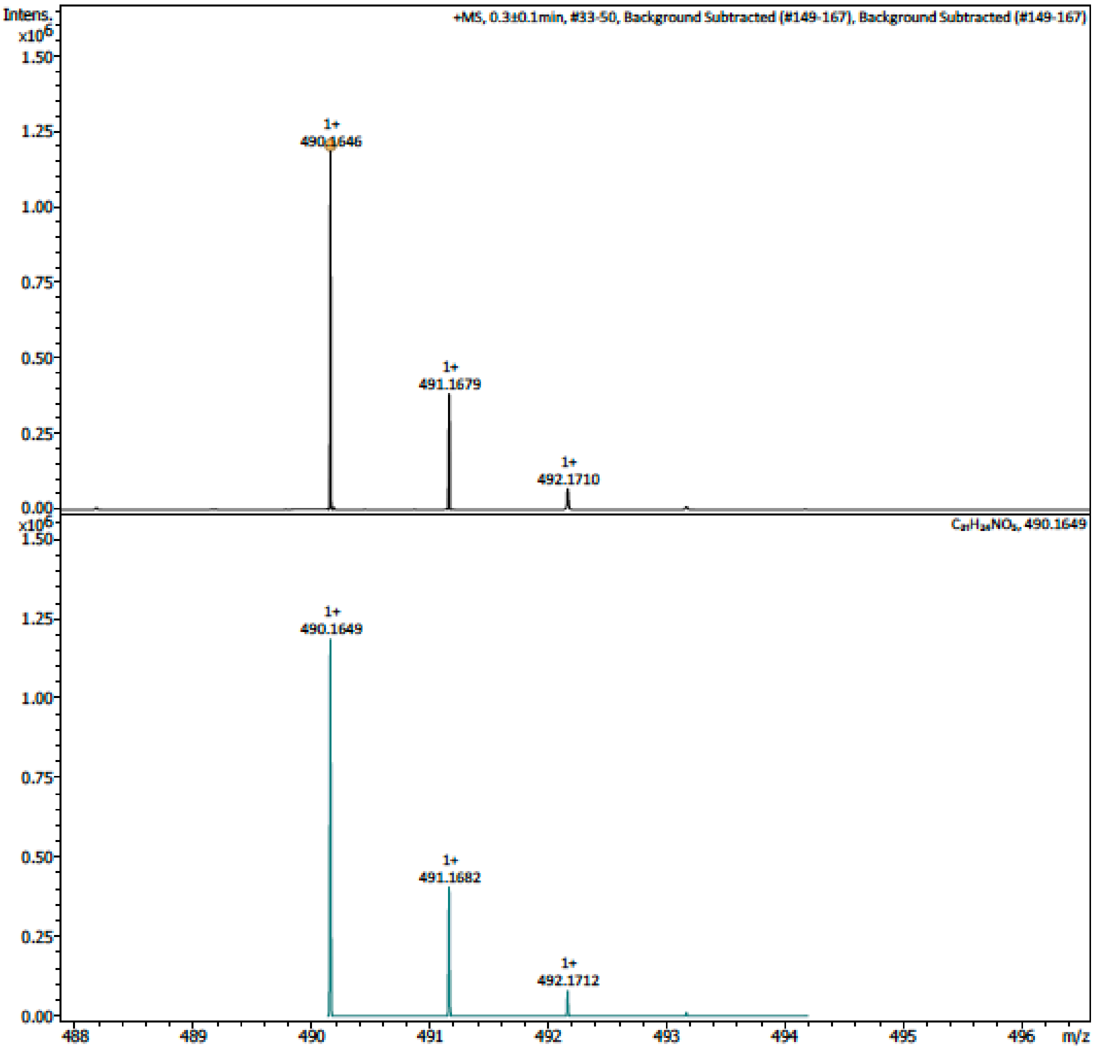

Meas. m/z	#	Ion Formula	Score	m/z	err [mDa]	err [ppm]	mSigma	rdb (neutral)	e⁻ Conf	N-Rule
490.1646	1	C29H22N4O4	78.70	490.1636	-1.0	-2.1	5.2	21.5	odd	ok
490.1646	2	C31H24NO5	100.00	490.1649	0.3	0.7	11.5	21.0	even	ok
490.1646	3	C30H18N8	90.41	490.1649	0.3	0.7	16.7	26.5	odd	ok
490.1646	4	C32H20N5O	36.98	490.1662	1.7	3.4	22.8	26.0	even	ok
490.1646	5	C15H14N20O	21.40	490.1654	0.8	1.7	52.0	19.5	odd	ok

Compound 34:

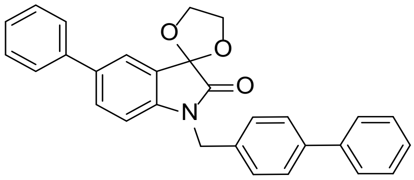
O
O
O
N

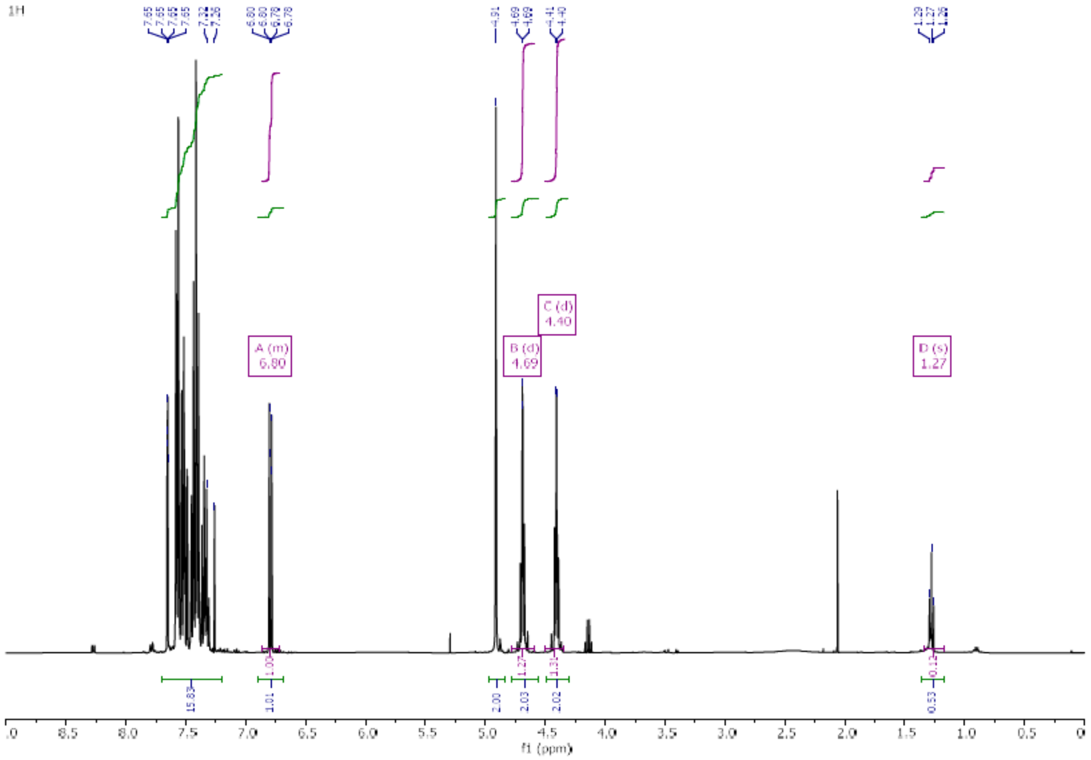
1H
A (m)
6.80
B (d)
4.69
C (d)
4.40
D (s)
1.27
f1 (ppm)

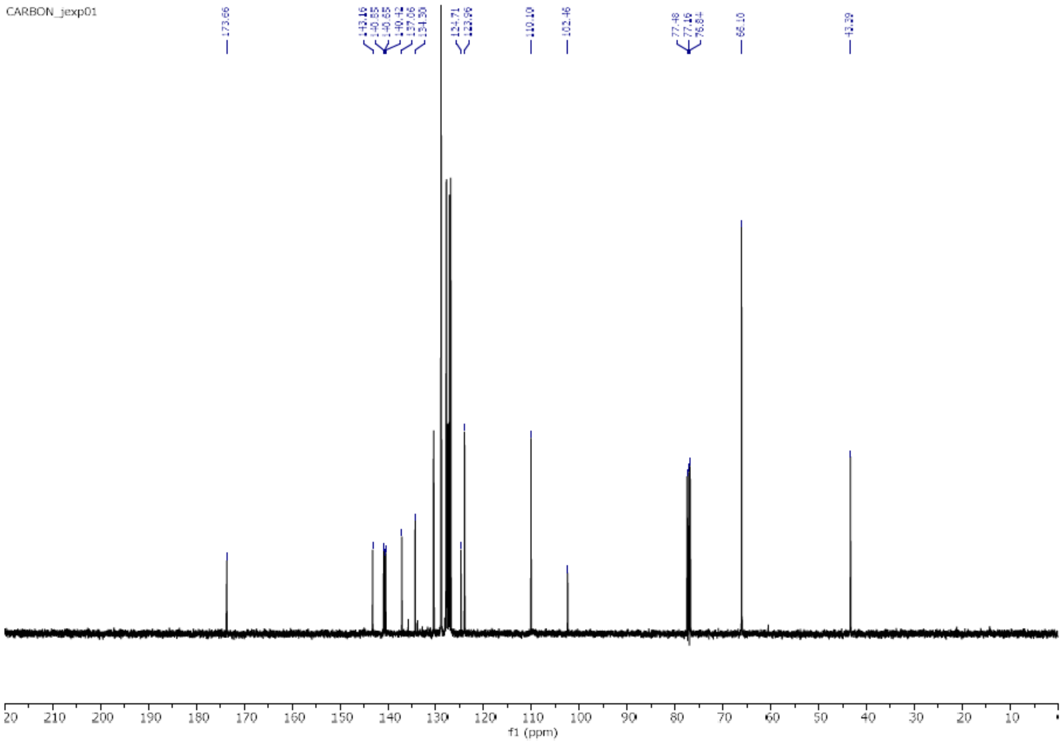
CARBON_jexp01
173.66
110.10
102.46
66.10
43.39
f1 (ppm)

UNIDADE DE ESPECTROMETRÍA DE MASAS E PROTEÓMICA

AREA DE INFRAESTRUCTURAS DE INVESTIGACION
Edificio CACTUS * Campus Vida
15782 Santiago de Compostela
Tel. 881816242. Ext. 16242
E-mail: sxemasas@usc.es
Web: https://www.usc.gal/es/servicios/unidades/unidad-espectrometria-masas-proteom

ESPECTRO APCI-FIA-Ion Trap

Analysis Info

Sample Name	12 APCI MS24-0272-8 433_9_01_2088.d	Acquisition Date	16/04/2024 19:25:53
Method	2088.m	Instrument	amaZon ETD

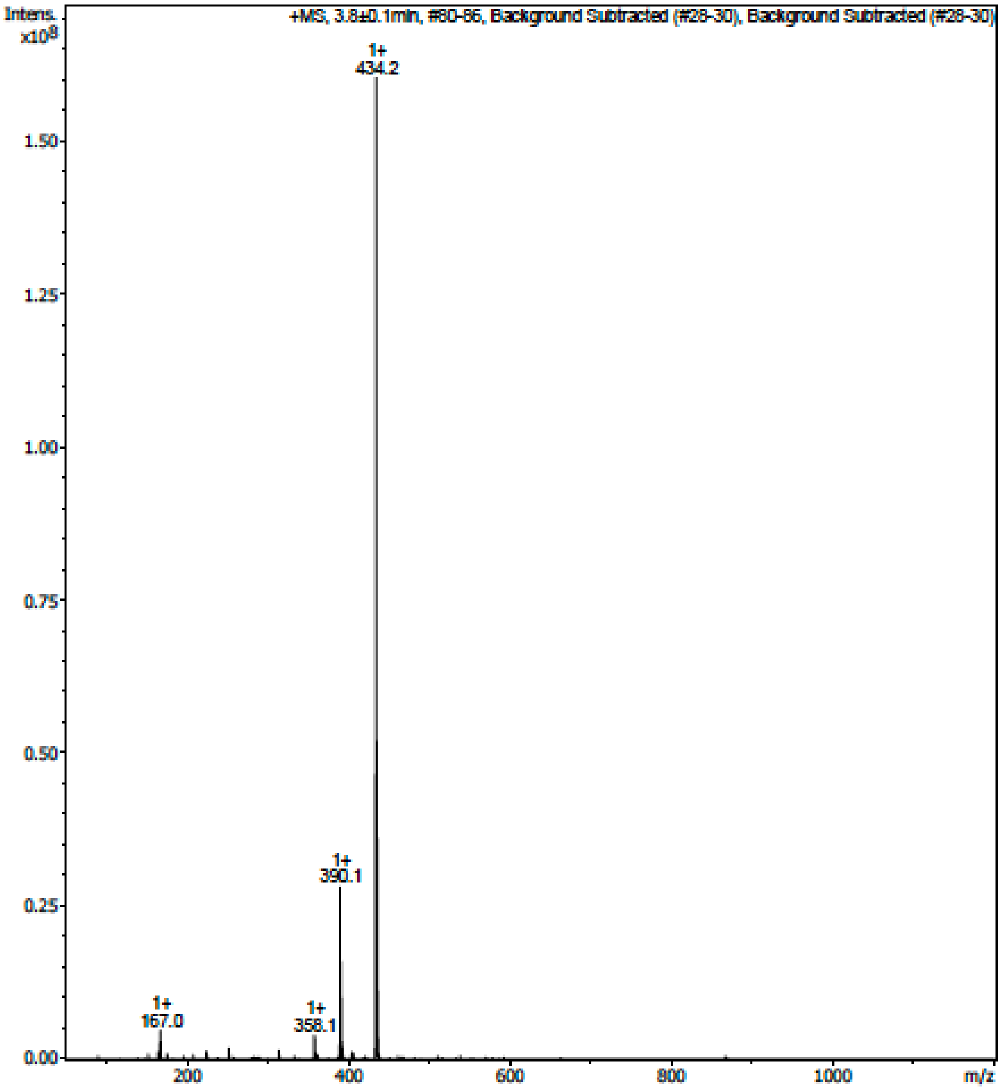

Medida De Masas Exactas

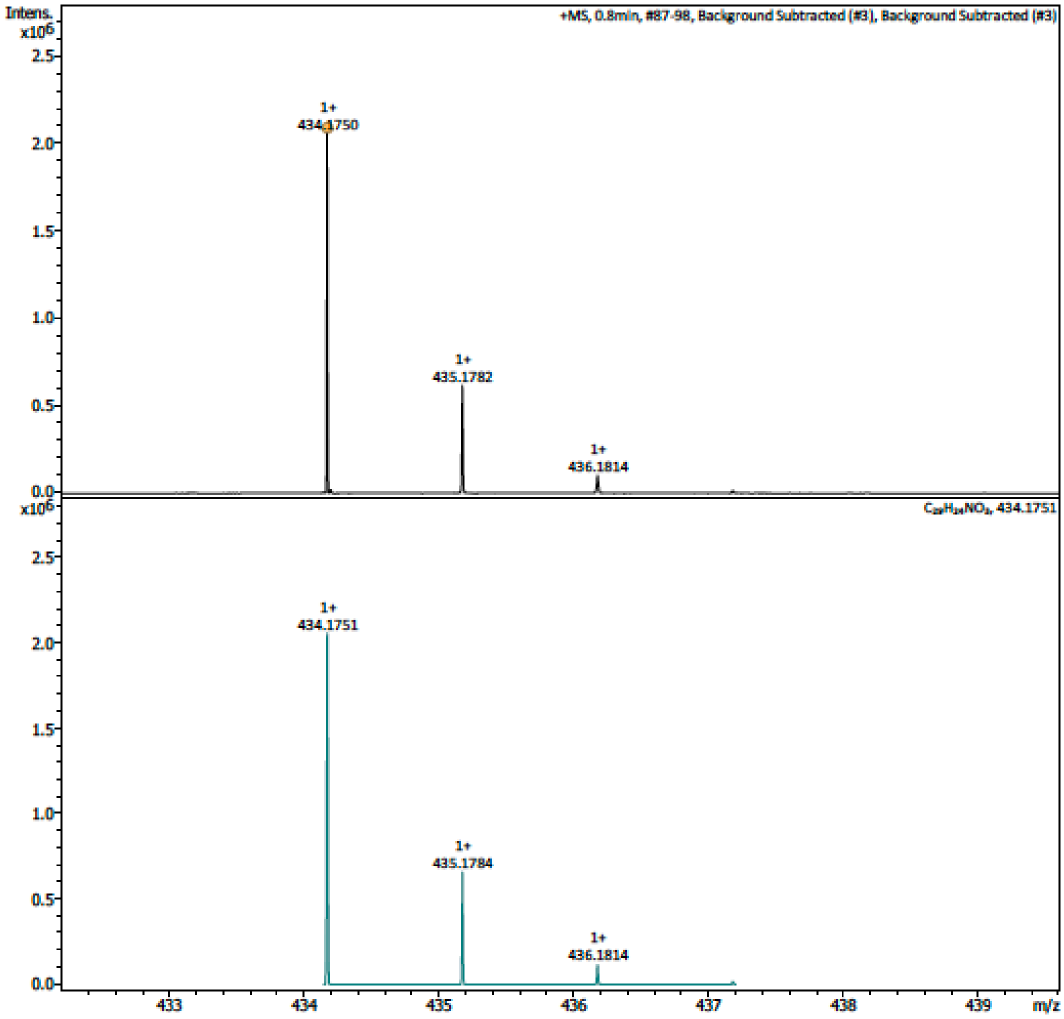

Meas. m/z	#	Ion Formula	Score	m/z	err [mDa]	err [ppm]	mSigma	rdb (neutral)	e⁻ Conf	N-Rule
434.1750	1	C27H22N4O2	45.45	434.1737	-1.3	-2.9	5.9	19.5	odd	ok
434.1750	2	C29H24NO3	100.00	434.1751	0.1	0.2	11.3	19.0	even	ok
434.1750	3	C14H20N13O4	18.26	434.1756	0.6	1.4	57.4	12.0	even	ok
434.1750	4	C15H26N6O9	14.78	434.1756	0.6	1.4	63.5	6.5	odd	ok
434.1750	5	C12H18N16O3	13.30	434.1742	-0.8	-1.7	64.2	12.5	odd	ok

Compound 35:

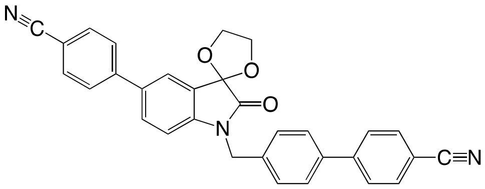
N≡C
O
O
O
N
C≡N

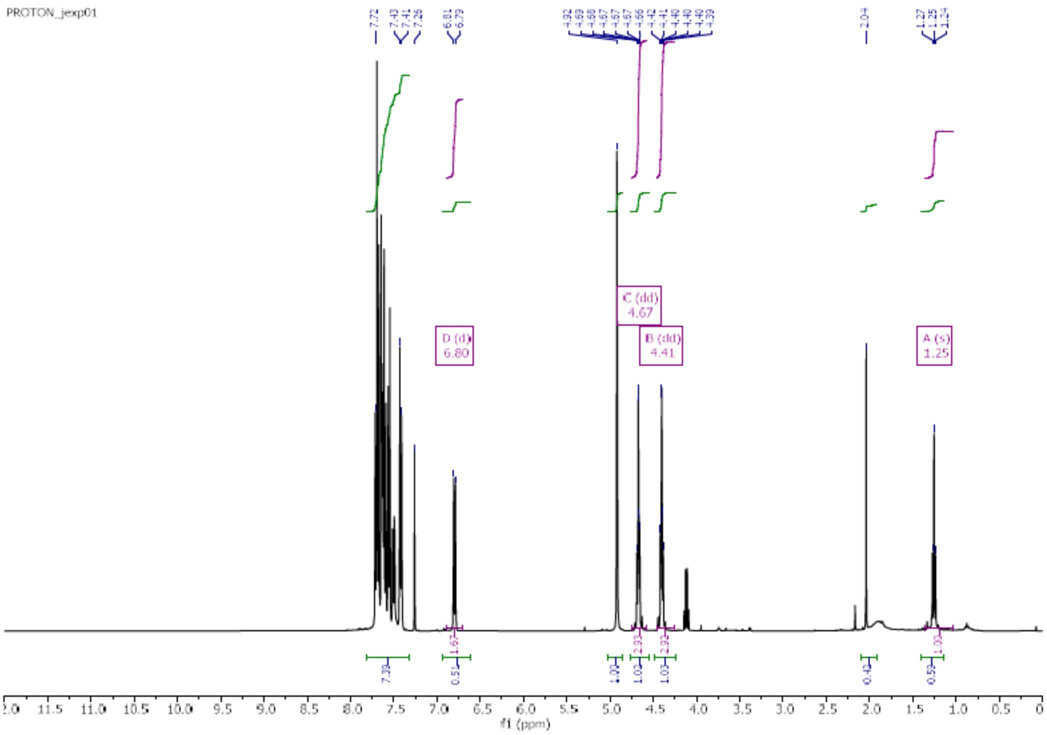
PROTON_jexp01
C (dd)
4.67
B (dd)
4.41
D (d)
6.80
A (s)
1.25
f1 (ppm)

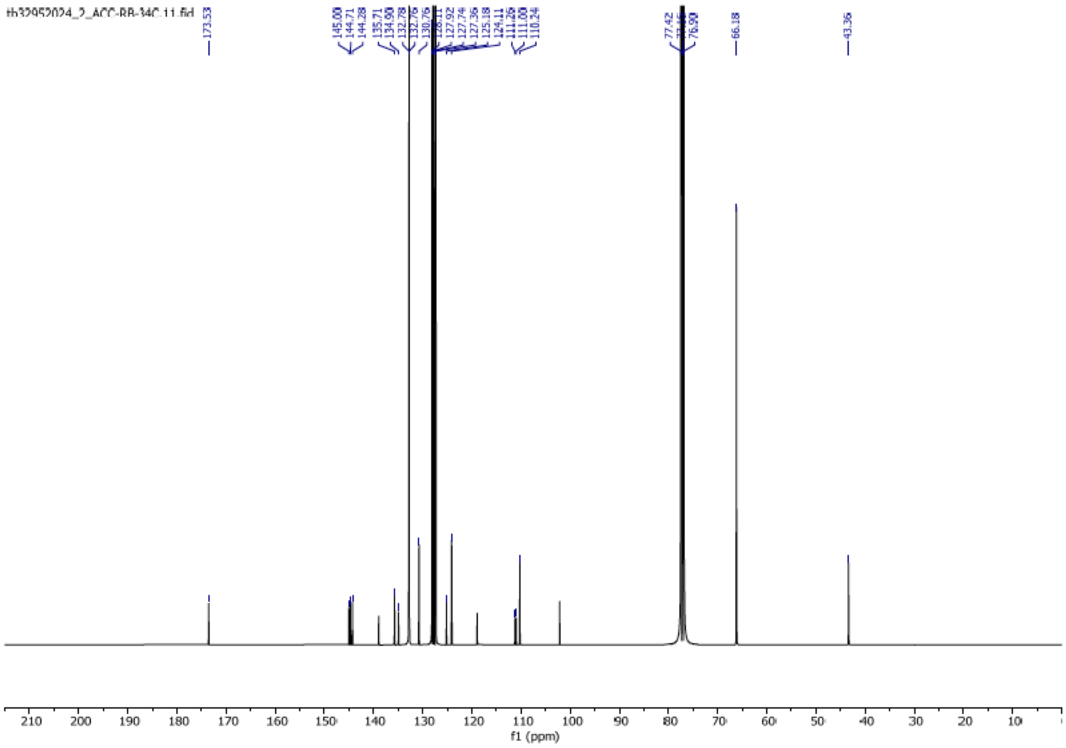
173.53
66.18
43.36
f1 (ppm)

UNIDADE DE ESPECTROMETRÍA DE MASAS E PROTEÓMICA

AREA DE INFRAESTRUCTURAS DE INVESTIGACION
Edificio CACTUS * Campus Vida
15782 Santiago de Compostela
Tel. 881816242. Ext. 16242
E-mail: sxemasas@usc.es
Web:
https://www.usc.gal/es/servicios/unidades/unidad-espectrometria-masas-proteom

ESPECTRO APCI-FIA-Ion Trap

Analysis Info

Sample Name	4 APCI MS-24-0378-2-tb3 483_3_01_2207.d	Acquisition Date	16/05/2024 11:04:10
Method	2207.m	Instrument	amaZon ETD

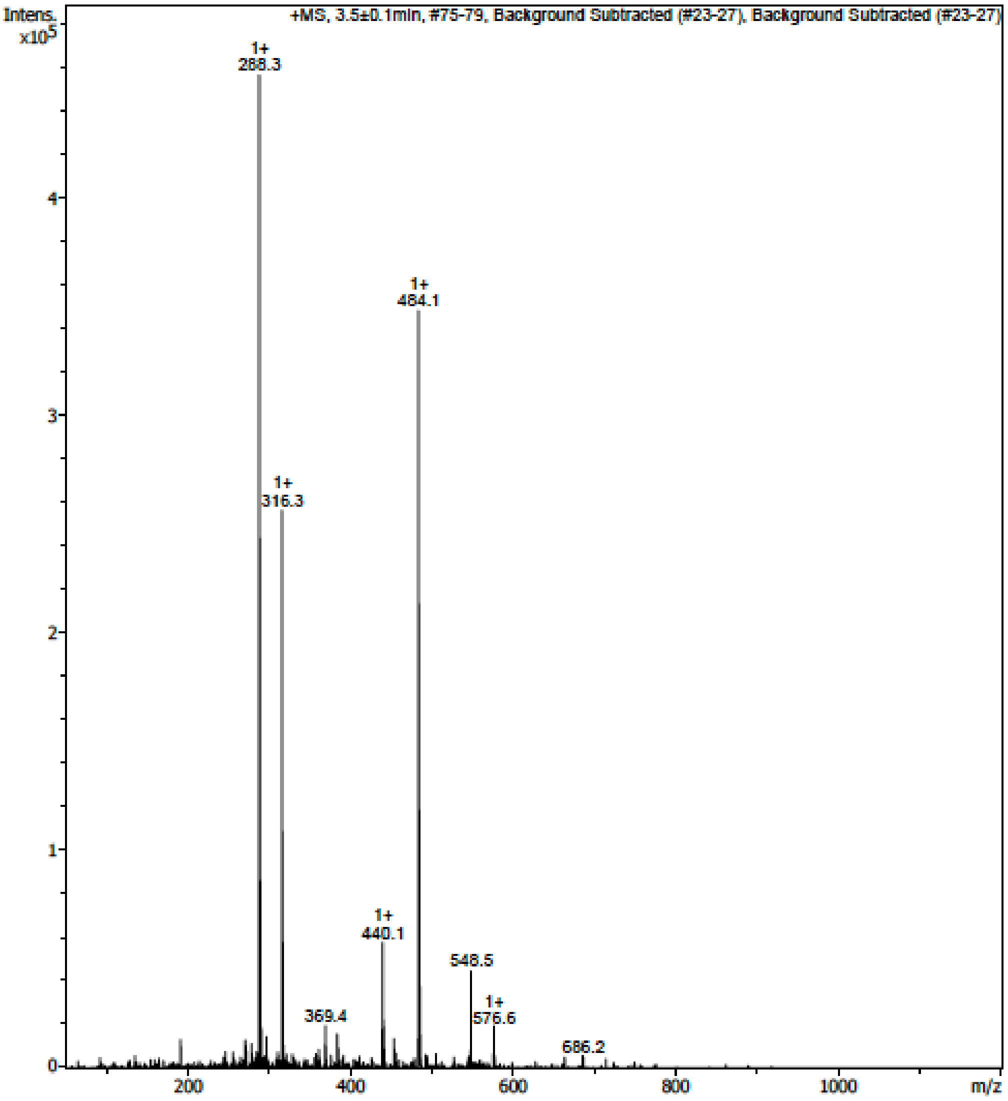

Medida De Masas Exactas

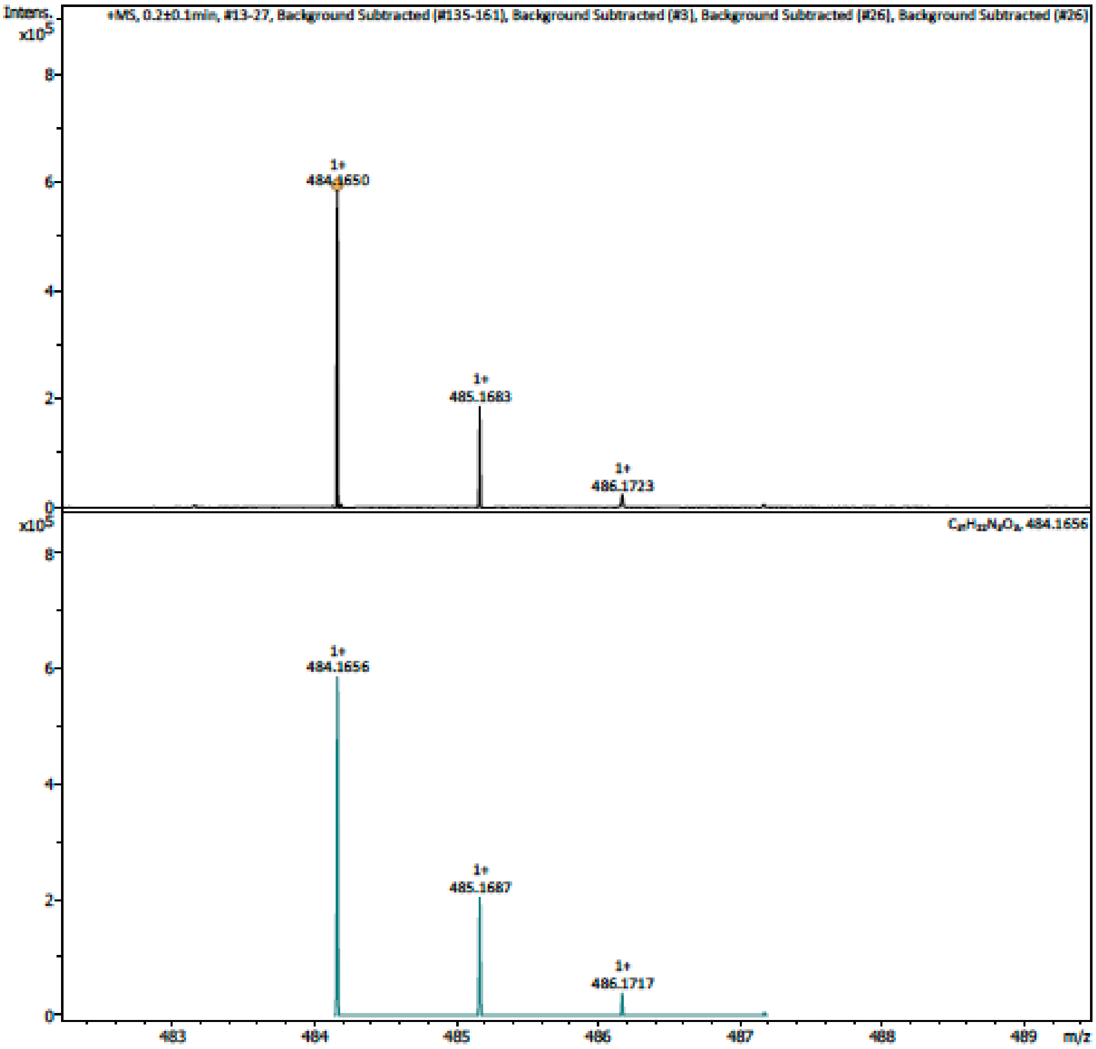

Meas. m/z	#	Ion Formula	Score	m/z	err [mDa]	err [ppm]	mSigma	rdb (neutral)	e⁻ Conf	N-Rule
484.1650	1	C29H20N6O2	99.08	484.1642	-0.8	-1.7	14.1	23.5	odd	ok
484.1650	2	C31H22N3O3	100.00	484.1656	0.5	1.1	20.2	23.0	even	ok
484.1650	3	C33H24O4	38.25	484.1669	1.9	3.9	26.5	22.5	odd	ok
484.1650	4	C16H18N15O4	26.31	484.1661	1.0	2.2	50.3	16.0	even	ok
484.1650	5	C18H30NO14	29.32	484.1661	1.1	2.2	55.8	5.0	even	ok

Table S1. Porosimetry of initial support and final monolithic catalyst.

Initial SiO_2 Sintered Monolith	3D-SiO_2@ Pd@SiK_2SiO_3 Monolith Catalyst
Average pore diameter: 0.28 mm	Average pore diameter: 9.84 mm
Median pore diameter: 0.27 mm	Median pore diameter: 1.48 mm
Total pore area: 0.08 m^2/g	Total pore area: 0.141 m^2/g
Porosity: 1.37%	Porosity: 9.12%

Table S2. Comparative performance of 3D-SiO_2@Pd@K_2SiO_3 with other catalysts.

Starting product	Final product	Catalyst	Method	Time (h)	Base /solvent	Temp. (ºC)	Yield (%)
**10**	**15**	$PdCl_2(PPh_3)_2$	Heck	12	TEA/DMF	90	10
**10**	**15**	Pd/C	Heck	12	TEA/DMF	90	30
**10**	**15**	$Pd(AcO)_2$	Heck	12	TEA/DMF	90	20
**10**	**15**	3D-SiO_2@ Pd@SiK_2SiO_3	Heck	12	TEA/DMF	85	78
**10**	**21**	$Pd(AcO)_2$	Suzuki	12	TEA/DMF	80	30
**10**	**21**	$Pd[(PPh)_3]_4$	Suzuki	12	TEA/DMF	80	20
**10**	**21**	Pd/C	Suzuki	12	TEA/DMF	80	30
**10**	**21**	3D-SiO_2@ Pd@SiK_2SiO_3	Suzuki	12	TEA/DMF	80	78
**10**	**23**	$Pd(AcO)_2$	Stille	12	-/ Toluene	80	35
**10**	**23**	Pd/C	Stille	12	-/ Toluene	80	35
**10**	**23**	3D-SiO_2@ Pd@SiK_2SiO_3	Stille	12	-/ Toluene	75	72
